# An approach to predict and inhibit Amyloid Beta dimerization pattern in Alzheimer’s disease

**DOI:** 10.1016/j.toxrep.2024.101879

**Published:** 2024-12-28

**Authors:** Sreekanya Roy, Sima Biswas, Anirban Nandy, Dipanjan Guha, Rakhi Dasgupta, Angshuman Bagchi, Parames Chandra Sil

**Affiliations:** a Department of Biochemistry and Biophysics, University of Kalyani, Kalyani, Nadia, West Bengal, India; b Bioinformatics Infrastructure Facility Center, University of Kalyani, Kalyani, Nadia, West Bengal, India; c Division of Molecular Medicine, Bose Institute, P-1/12, CIT Scheme VII M, Kolkata, West Bengal 700054, India

**Keywords:** Alzheimer’s Disease, Aβ molecule, mutation, drug toxicity, drug prediction, molecular docking

## Abstract

Alzheimer’s Disease (AD) is one of the leading neurodegenerative diseases that affect the human population. Several hypotheses are in the pipeline to establish the commencement of this disease; however, the amyloid hypothesis is one of the most widely accepted ones. Amyloid plaques are rich in Amyloid Beta (Aβ) proteins, which are found in the brains of Alzheimer’s patients. They are the spliced product of a transmembrane protein called Amyloid Precursor Protein (APP); when they enter into the amylogenic pathway, they get cleaved simultaneously by Beta and Gamma Secretase and produce Aβ protein. Appearances of Amyloid plaques are the significant clinical hallmarks of this disease. AD is mainly present in two genetically distinct forms; sporadic and familial AD. Sporadic Alzheimer’s Disease (sAD) is marked by a later clinical onset of the disease, whereas, familial Alzheimer’s Disease (fAD) is an early onset of the disease with mendelian inheritance. Several mutations have been clinically reported in the last decades that have shown a direct link with fAD. Many of those mutations are reported to be present in the APP. In this study, we selected a few significant mutations present in the Aβ stretch of the APP and tried to differentiate the wild-type Aβ dimers formed in sAD and the mutant dimers formed in fAD through molecular modelling as there are no structures available from wet-lab studies till date. We analysed the binding interactions leading to formations of the dimers. Our next aim was to come up with a solution to treat AD using the method of drug repurposing. For that we used virtual screening and molecular docking simulations of the already existing anti-inflammatory drugs and studied their potency in resisting the formation of Aβ dimers. This is the first such report of drug repurposing for the treatment of AD, which might pave new pathways in therapy.

## Highlights

• Functionalities of anti-inflammatory drugs.
• Pharmaco-kinetics study.
• Toxicity predictions of anti-inflammatory drugs.
• Identification of potential therapeutics against AD.

## Graphical Abstract

**Figure:**
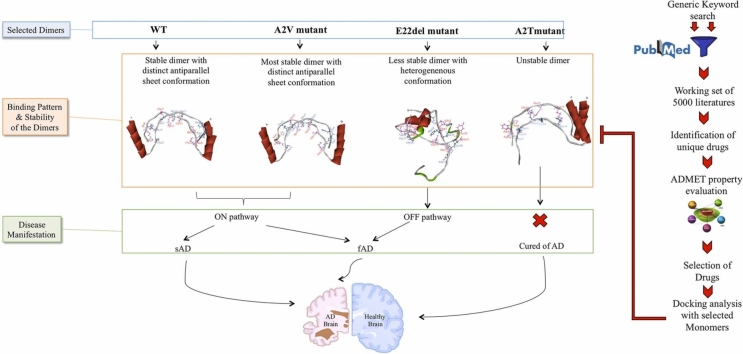

## 1 Introduction

Neurodegenerative disorders have impacted a large portion of the human population in the last ten years. Natural ageing can result in neurodegenerative illnesses, which are fairly common and concerning [1]. Alzheimer’s Disease (AD) is the most prevalent neurodegenerative disease, which was first reported on November 3, 1906 by clinical psychiatrist and neuroanatomist, Dr. Alois Alzheimer. He reported on a 50-year-old patient from the time of her admission for psychosis, progressive sleep and memory problems, violence, and disorientation until her death five years later. The patient was found to have distinctive plaques and neurofibrillary tangles in the brain histology. Alzheimer later reported three further instances in 1909 and a variety that was "plaque-only" in 1911. When the original specimens were examined again in 1993, it was discovered that the "plaque-only" variation was a different phase of the same process [2], [3].

The second decade of studying the disease paved the way for scientists to understand the pathology rather than the molecular mechanism of disease propagation [4]. Therefore, AD was distinguished into two forms: sporadic Alzheimer's Disease (sAD) which is marked by late onset of the disease and familial Alzheimer's Disease (fAD), on the contrary, is marked by early onset of the disease with mendelian inheritance [5]. Moreover, fAD has also been related to the presence of mutation(s) in Amyloid Precursor Protein (APP), which is the source of Amyloid Beta (Aβ) [6], [7], one of the clinical hallmarks of AD. One of the most widely accepted hypothesis regarding the formation of AD is the Amyloid plaque Hypothesis [8].

The amyloid plaque hypothesis states that the proteolytic cleavage of APP [9] with the help of Beta Secretase (BS) and Gamma Secretase (GS) in the amylogenic pathway [10], [11], [12] leads to the formation of Aβ, which is majorly a 40–42 amino acid residue long peptide [3], [13]. It is noteworthy that AD in recent times is also regarded as a prion disease as the amyloid plaques, the other clinical hallmark of AD, are essentially accumulations of miss-folded Aβ peptides [12], [13].

In this work, we are thus more concerned about the amyloid dimers formed in the disease. We selected some significant mutations which were clinically reported within the Aβ stretch of APP that are linked with fAD. Till date, no structure of the dimer is available from wet-lab studies. In this work, we used the amino acid sequence of the peptide to build the three-dimensional structure of the peptide through molecular modelling. The binding interactions in the dimers were analyzed further.

Our next aim was to conduct a drug-repurposing study with the already known anti-inflammatory drugs for the treatment of AD. For that we performed virtual screening and literature mining and came up with a list of candidate drugs. This is the first such report and we believe that our study may help in the development of future candidate drugs for the treatment of AD.

## 2 Materials and methods

### 2.1 Preparation of peptide molecule and selection of mutants

The 3D structure of the Aβ molecule was not available in the Protein Data Bank (PDB). The FASTA sequence of APP was downloaded from the NCBI database and then the Aβ stretch was taken to get its unique FASTA sequence. A model was built with the wild type (WT) Aβ using the SWISS MODEL tool (https://swissmodel.expasy.org/). The two missense mutants of Aβ, namely A2V and A2T, were built using the ‘build mutant’ module of Discovery Studio 2.5 (DS 2.5), whereas the one deletion mutant E22del was built using the HHpred webserver (https://toolkit.tuebingen.mpg.de/tools/hhpred).

### 2.2 Validation of the peptide structure

SAVESv6.0 server (https://saves.mbi.ucla.edu/) was used to evaluate the stereo-chemical properties of the structures, where Ramachandran plots were drawn with the PROCHECK tool [14]. It is likely that every time the subjected protein models would have all its residues in the allowed region of the Ramachandran Plot [15]. In our study, in the initial WT Aβ model, some residues present in the loop regions, were present in the disallowed regions. Therefore, we refined the model with ModLoop tool (https://modbase.compbio.ucsf.edu/modloop/). Similar steps were followed for the mutant Aβ monomers. The final refined structures of the peptides were used for further experimentations and the corresponding energy values are presented in the Section 3.1.

### 2.3 Energy minimization

We further refined the modelled structures by the method of energy minimizations with the help of ‘minimization’ module of Discovery Studio 2.5 (DS 2.5) where it was subjected to a 5000 cycle of Smart Minimizer run till the RMS gradient of energy derivative would reach 0.1 kcal/mol.

### 2.4 Peptide-Peptide docking

The aforementioned energy optimized structures of the monomeric Aβ peptides were then used to generate the corresponding dimers through peptide-peptide docking simulations. We used the tool Cluspro server (https://cluspro.bu.edu/). Cluspro implements FFT-based global sampling by using pairwise potentials implemented in PIPER. In PIPER, the ligands (here peptide) are free to move while the receptor's conformation remains fixed. In the algorithm for PIPER docking, based on interaction energy estimates, likely conformations were grouped together on a grid where the highly populated lowest energy conformations are clustered [16], [17], [18]. From this docking result the top ten docking poses were collected for further study.

### 2.5 Prediction of protein stability for the wild type and mutant Aβ peptides

We further predicted the protein stabilities for the wild type and mutant Aβ peptides. For that we used the tool Prodigy server [19], [20]. The corresponding values of the energy terms are presented in Section 3.2.1.

### 2.6 Calculation of binding free energy

To determine the best-docked complex, each of the retrieved docked poses from the docking studies, were typed with CHARMm force field in DS 2.5 platform where they were optimized and re-ranked based on their binding free energy (BFE) values. The best docking pose was defined as the docked pose with the lowest binding free energy.

### 2.7 Visualization of the peptides

The secondary structure of the monomers and dimers of the selected peptides were visualized by VMD software that is useful to visualize the distribution of secondary structures in the selected peptides.

### 2.8 Meta-analysis for anti-inflammatory drugs, preparation of ligand library and drug toxicity prediction

To determine the targeted drugs for our study we used PubMed database as the source of the literatures available and used for literature mining. We narrowed down our search by looking into specific published literatures particularly emphasizing on the anti-inflammatory drugs, which are being targeted for neurodegenerative diseases or those being used for treating dementia. After we curated our required dataset by simply searching through generic keywords, the literates were sorted on the basis of the different anti-inflammatory drugs.

The existing drugs have well-defined anti-inflammatory effects. However, for the sake of clarity, we checked their drug likeliness properties. Furthermore, our aim is to come up with a single inhibitor for the associated disorders. So, we conducted the experiments as a first step towards drug-repurposing.

We then moved towards further validations of the ligands (here anti-inflammatory drugs) as obtained for the aforementioned analyses. In order to do so, we used Autodock.4.2 tool. We made a library of the ligands from our previous analysis. From there, we chose one ligand at a time and subjected it to bind to the WT, A2V and E22del monomers as receptors. We performed this process for all the ligands present in our ligand library. We noted the resulting binding free energy values and presented them in the Supplementary Table 4.

### 2.9 Preparation of small molecule structures

The selected anti-inflammatory drugs in the prepared ligand library are regarded as the small molecule in this experiment. Their structures were downloaded from PubChem database (https://pubchem.ncbi.nlm.nih.gov/) and the post processing of the structures were done in Discovery Studio 2.5 (DS 2.5).

### 2.10 Peptide-Small molecule docking

Once all the required small molecules were prepared and verified. We performed peptide- small molecule docking simulation with the help of AutoDock v4.2 software [21], which is intended to forecast the binding patterns of small compounds and potential drugs to receptors (here Aβ monomers). Instead of using a deterministic approach, AutoDock v4.2 uses a Lamarckian genetic algorithm, which uses a variant of the genetic algorithm. As long as the objective function continues to improve, the local optimization begins by investigating random conformations of the small molecule with crossover, mutation, and recombination events. While continual failure narrows the search until the step size drops below the ultimate threshold, constant improvement increases the number of random search steps. The docked complexes’ fitness is assessed after every repetition, and the top 50 % advance to the next round where a force-field-based scoring mechanism is employed [22]. We thus get the output file where the binding free energies, RMSD etc. for each cluster are noted with their respective docked coordinates. From this docking result we selected top three for each set of anti-inflammatory drugs and Aβ monomer docked complexes for further analysis.

### 2.11 Determination and visualization of interacting residues

Once the best docked pose of each of the homo-dimers as well as the peptide-ligand complexes were retrieved we studied the interacting amino acid residues using the DIMPLOT program in LigPlot^+^ v.2.2 software [23], [24] and DS 2.5 visualization protocol respectively, further analyzed the bonding pattern in the residues.

### 2.12 Validation of docking site

**Fig. 1:**
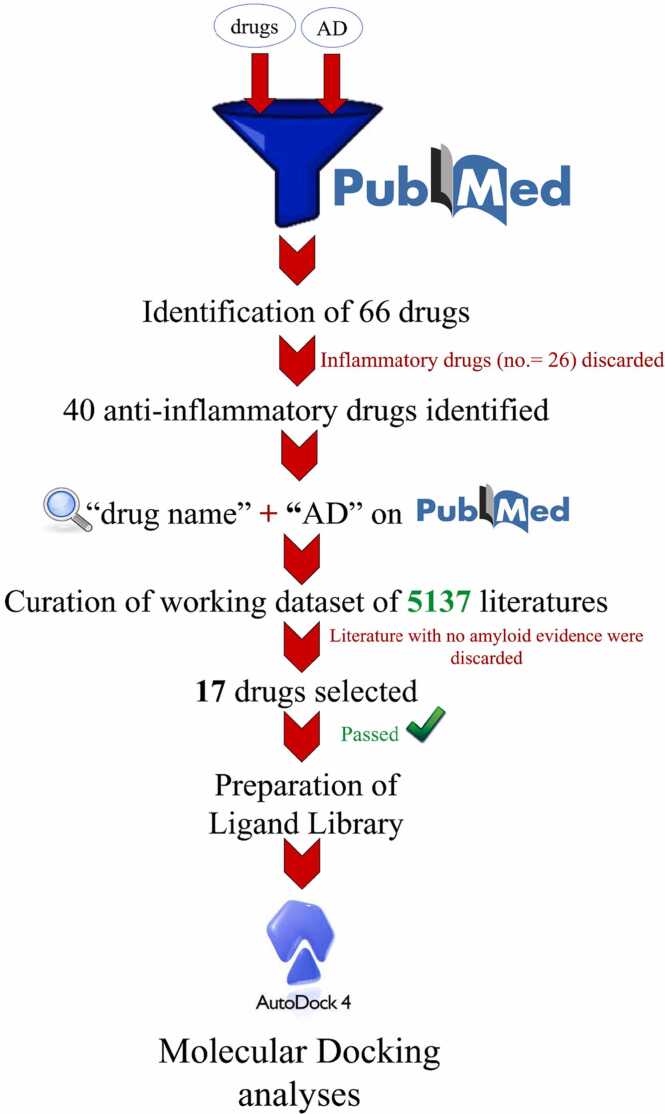
Workflow for selection of drugs. The schematic representation of fishing out the selected anti-inflammatory drugs through literature mining and toxicology evaluation.

The docking study of the peptide-ligand complexes was solely dependent on the blind docking and it was then cross-verified by the result of COACH server’s (https://zhanggroup.org/COACH/) binding site prediction for the WT monomer. It is a meta-server method for predicting peptide-ligand binding sites where, given the target protein's structure, COACH will use the two comparative methods, TM-SITE and S-SITE, to identify ligand-binding templates from the BioLiP protein function database by comparing binding-specific substructures and sequence profiles. This will result in predictions of complementary ligand binding sites. Hence, we selected the common residues obtained from the COACH server result and performed site directed docking with the same set of drugs and the Aβ monomers previously used. Fig. 1

## 3 Results

### 3.1 Structural analysis of the modeled peptides

**Fig. 2:**
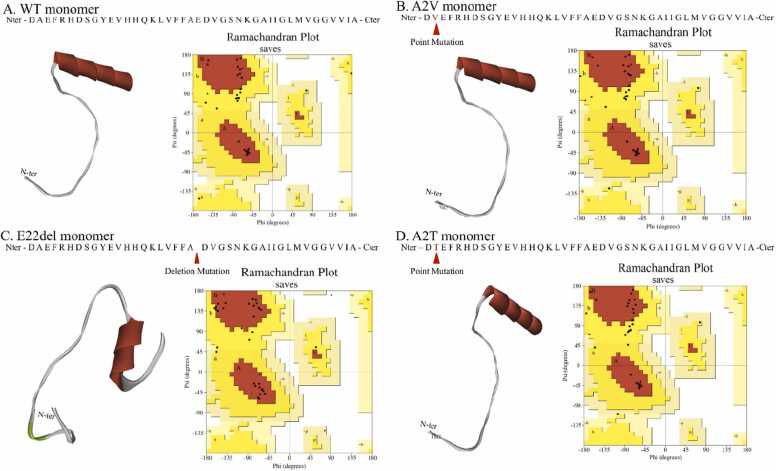
Sequences, 3D structures & respective Ramachandran plots of selected monomers. The sequences of the wild type monomer, A2V mutant monomer, E22del mutant monomer and A2T mutant monomer along with their secondary structural dispositions and Ramachandran Plots are represented in panel A, B, C and D respectively. The point mutation in the sequence is represented in red and the deletion mutation is represented as a space in the sequence.

The four peptides’ monomers as mentioned before were seen to have all the residues in the allowed region of the Ramachandran plot as shown in Fig. 2. It was further observed that the potential energy values of the monomeric peptides were:

wild type (WT): −2397.07 kcal/mol,

mutant A2V: −2402.04 kcal/mol,

mutant A2T: −2406.73 kcal/mol and

mutant E22del: −2273.59 kcal/mol.

The distributions of secondary structural elements in each peptide are elaborated in Table 1 along with the corresponding Supplementary Fig 1 upper panel as its reference.

**Table 1:** Secondary structure distribution of selected monomers.

**Secondary Structure Distribution**	**WT**	**A2V mutant**	**E22del mutant**	**A2T mutant**
**Coil**	1–8, 14–15,19,26, 41–42	1–8, 14–15,19,26, 41–42	1–2, 12–14, 18–22, 36–41	1–8, 14–15,19,26, 41–42
**Turn**	9–13, 16–18, 20–25	9–13, 16–18, 20–25	3–11, 15–17, 23–26	9–13, 16–18, 20–25
**Alpha Helix**	27–40	27–40	27–35	27–40

### 3.2 Analysis of the peptide complexes

Each peptide monomers were used to build homo-dimers respectively through peptide-protein docking simulation using the Cluspro server.

#### 3.2.1 Analysis of protein stability

The binding free energy of a protein is an important indicator of its stability. We computed the binding free energy values of the WT and mutant Aβ peptides to make a comparative estimate of the effects of the mutations on protein structure. The corresponding values were as follows:

wild type (WT): −10.2 kcal/mol,

mutant A2V: −10.5 kcal/mol,

mutant A2T: −7.3 kcal/mol and

mutant E22del: −8.2 kcal/mol.

Prodigy server (https://rascar.science.uu.nl/prodigy/) [19] was used to compute these values, where the cured variant A2T mutant dimer was least stable of the lot.

#### 3.2.2 Distribution of secondary structure in the selected homo-dimers

To gain an insight into the distributions of secondary structural elements of the selected dimers VMD was used and the detailed distributions are presented in Table 2 and shown in Supplementary Fig 1. It was observed that in comparison to the monomer peptides, described in Table 1 and shown in Supplementary Fig 1, the selected homo-dimers showed incorporation of isolated bridge structure except that in E22del mutant dimer.

**Table 2:** Secondary structure distribution of selected homodimers.

**Types**	**coil**		**turn**		**alpha helix**		**isolated bridge**		**extended conformation**	
	Chain A	Chain B	Chain A	Chain B	Chain A	Chain B	Chain A	Chain B	Chain A	Chain B
**WT**	1–8, 15,19–22, 26	1–11, 13–15, 19–25,42	9–13, 16–18, 23–25	16–18	27–42	26–41	14	12		
**A2V mutant**	1–8, 19	1–3, 8–10, 13–14, 16–18,24,42	9–10, 12–13, 16–18, 20–26	4–7, 19–22, 24–25	27–42	26–41	11	15	14,15	11,12
**E22del mutant**	1–2, 12–14, 19–20, 24–26, 37, 39–41	4, 12, 15–21, 39–41	3–11, 15–16, 21–23, 34–36	1–3, 5–11, 23–25, 37–38	27–33	26–36	38	22	17,18	13,14
**A2T mutant**	1–8, 19, 25–26	1–3, 8, 12–17, 21–23, 42	9–18, 20–24	4–7, 9–11, 18–20, 24–25	27–42	26–41				

#### 3.2.3 Determination of the binding interactions of the peptides

**Fig. 3:**
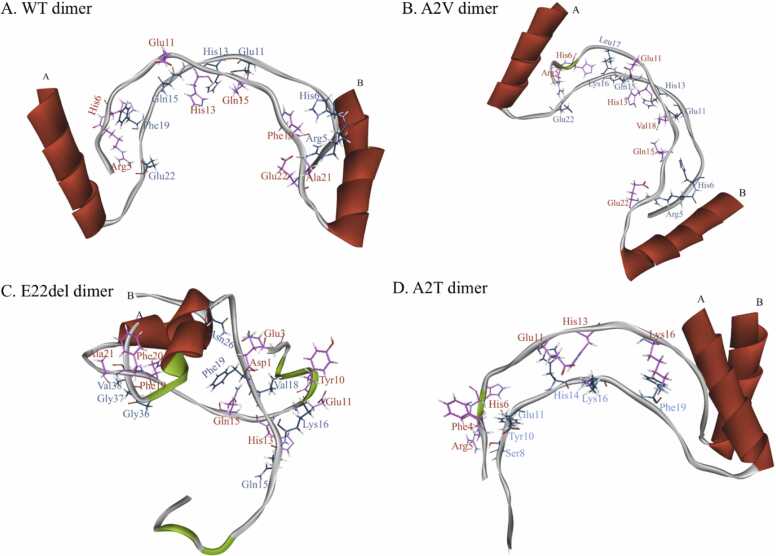
3D structures of selected dimers. The wild type dimer (A); A2V dimer (B); E22del dimer (C) and A2T dimer (D) are represented where the residues from the chains are represented in blue (Chain A)& red (Chain B).

We calculated the binding interactions of the docked poses using the MM-PBSA method and ranked them accordingly. The desirable docked poses of the WT dimer, A2V mutant dimer, A2T mutant dimer and E22del mutant dimer are shown in Fig. 3. The best binding interaction energy (BIE) during WT dimer, A2V mutant dimer, A2T mutant dimer and E22del mutant dimer formation were found to be −74.6 kcal/mol, −78.18 kcal/mol, −70.45 kcal/mol and −69.60 kcal/ mol respectively.

#### 3.2.4 Interacting residues in the selected homo-dimers

**Fig. 4:**
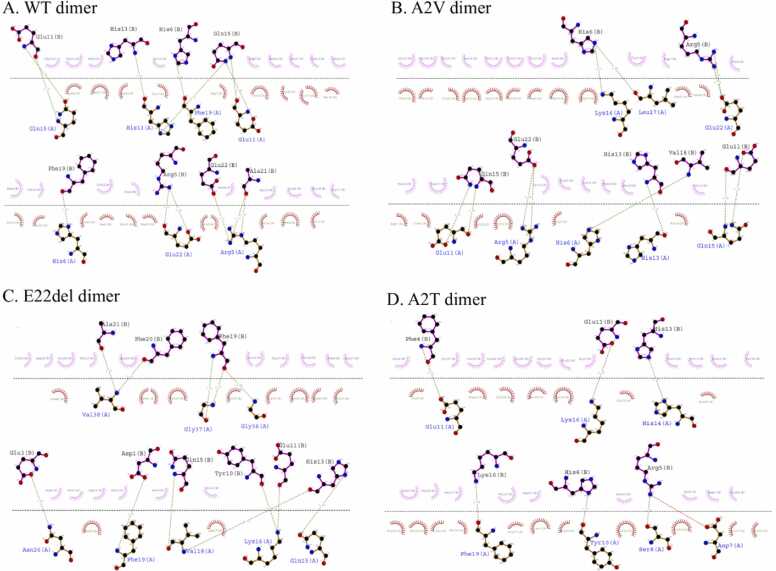
2D structures of selected dimers. The DimPlot of wild type dimer (A); A2V dimer (B); E22del dimer (C) and A2T dimer (D) are represented where the hydrogen bonds are shown in green. The amino acid residues which are involved in hydrogen bonding are shown in blue and black coloured text for Chain A and Chain B respectively.

The 2D Dimplot of each of the selected docked complexes gave us information on the non-covalent interactions (here hydrogen bond) holding the monomers together. Fig. 4 and Table 3 shows the amino acids, which are responsible to stabilize or interacting during the dimer formation. We observed that in WT dimer, there are thirteen H-bonds, the A2V mutant dimer forms eleven H-bonds, the A2T mutant dimer has six H-bonds and the E22del mutant dimer contains twelve H-bonds.

**Table 3:** The binding energy profile of the homodimers of the wild type (WT) and mutant Aβ42. The interacting amino acid residues of each chain are shown.

**Dimer**	**BFE** **(kcal/mol)**	**Interacting residues**		**No. of H-bonds**	**BIE (kcal/mol)**	**Type of AD**	**Special Characteristics**
		**Chain A**	**Chain B**				
**WT**	−10.2	Arg5	Ala21, Glu22	13	−74.6006	sAD	
		His6	Phe19				
		Glu11	Gln15				
		His13	His13, Gln15				
		Gln15	Glu11				
		Phe19	His6				
		Glu22	Arg5				
**A2V mutant**	−10.5	Arg5	Glu22	11	−78.1829	fAD	autosomal recessive mutation
		His6	Val18				
		Glu11	Gln15				
		His13	His13				
		Lys16	His6				
		Leu17	His6				
		Gln22	Arg5				
**E22del mutant**	−8.2	Gln15	His13	12	−69.601	fAD (Osaka Variant)	no senile plaque formation
		Lys16	Tyr10, Glu11				
		Val18	His13, Gln15				
		Phe19	Asp1				
		Asn26	Glu3				
		Gly36	Phe19				
		Gly37	Phe19				
		Val38	Ala21, Phe20				
**A2T mutant**	−7.3	Ser8	Arg5	6	−70.4468	NA	cured of AD
		Tyr10	His6				
		Glu11	Phe4				
		His14	His13				
		Lys16	Glu11				
		Phe19	Lys16				

### 3.3 Literature mining of the selected anti-inflammatory drugs & virtual screening for ligand library preparation and toxicity prediction

Literature mining is a powerful analysis method, which help us to identify the particular dataset of literatures appropriate for our specific problem from the enormous literature databases such as PubMed. We initiated our search by a generic keyword search by using “Alzheimer’s Disease” and “drugs” as the input. In result, we got literatures specific for 66 different drugs between the tenure 2013–2023 among which 40 were anti-inflammatory drugs, as we desire for this study described in Supplementary Table 1. Now, we took each of the specific 40 “drug name” as an input keyword along with “Alzheimer’s Disease” to again, search in PubMed in order to include all the literatures available in the working dataset. As a result, more than five thousand papers appeared in total, which is described in Supplementary Table 2. Thereafter, all the literatures were segregated according to the involved drugs; only those literatures were selected, which has evidence of “amyloid” in their work and it should also be noted that, only those research articles were considered for further studies and others were excluded, the details of which is mentioned in Supplementary Table 3. Using all these filters we successfully funneled-out seventeen anti-inflammatory drugs; these constituted our ligand library that was subjected to further experiment. Side by side, we performed virtual screening of the aforementioned drug molecules to check their efficacies as potent anti-AD therapeutics. From molecular docking simulations of the molecules with the monomeric mutant Aβ peptides, we selected the top 5 compounds in each case. The details of their binding interactions are presented in Supplementary Table 4. As an additional step, we checked their drug likeliness (Supplementary file).

#### 3.3.1 Binding free energy of the selected peptide-small molecule docked complexes

We noted the binding free energy of the complexes and ranked them in an ascending order. The one with lowest BFE was selected to study monomer-drug complexes as shown in Supplementary Table 4. Valdecoxib was seen to found in the group comprising of the five least BFE among the selected drugs for each monomer.

#### 3.3.2 Interacting residues in the selected peptide-small molecule docked complexes

**Fig. 5:**
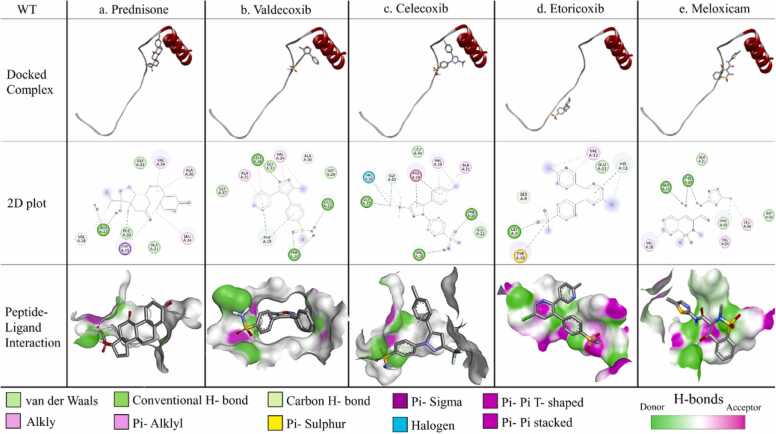
Protein-ligand interaction for WT monomer. The docked complex of individual ligand with WT monomer, their corresponding 2D plot and interaction are represented in the designated panels with all the corresponding colour coding.

**Fig. 6:**
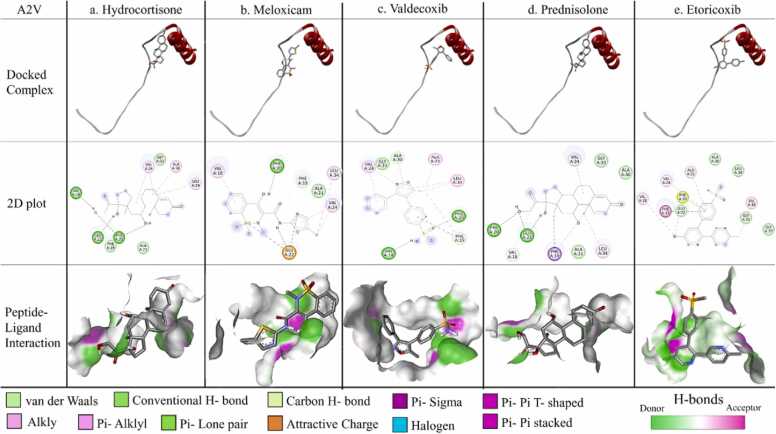
Protein-ligand interaction for A2V mutant monomer. The docked complex of individual ligand with A2V mutant monomer, their corresponding 2D plot and interaction are represented in the designated panels with all the corresponding colour coding.

**Fig. 7:**
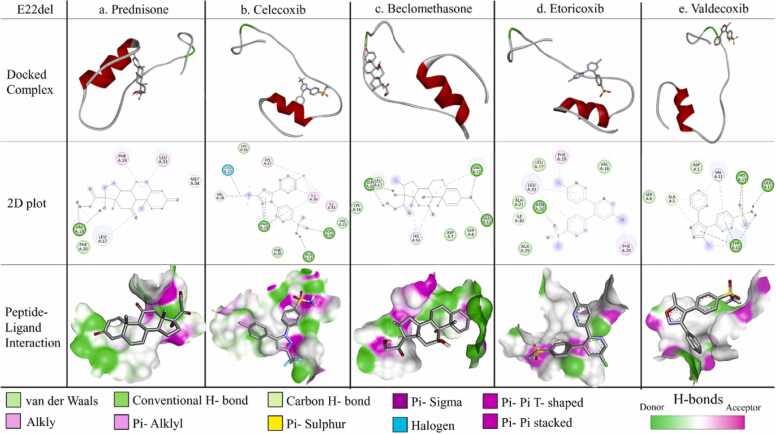
Protein-ligand interaction for E22del mutant monomer. The docked complex of individual ligand with E22del mutant monomer, their corresponding 2D plot and interaction are represented in the designated panels with all the corresponding colour coding.

To find out the interacting residues in the selected peptide-small molecule docked complexes, the interaction between Aβ monomers and anti-inflammatory drugs were thoroughly studied using DS 2.5 platform also shown in Fig. 5, Fig. 6, Fig. 7. It is to be noted that the residues in Table 4 are colour coded accordingly.

**Table 4:** The interacting amino acid residues of monomers with ligand molecules; where the colour code is indicated as: bold black for shared residue with dimer formation, bold red for residues responsible for both conventional H-bond (s) and dimer formation, finally blue indicates residues making only conventional H-bond (s).

black bolded residues are those participating in dimer formation, bold red are the residues which forms conventional H-bond with the particular ligand and were also responsible for dimer formation and lastly the blue marked residues are those which are forming only conventional H-bond with the specific ligand.

From the Table 4 the following results are worthy a view: a. In WT; GLU22 is interacting strongly with prednisone (two H-bonds), valdecoxib and meloxicam through formation of H-bonds.

b. In mutant A2V; VAL18 is interacting with hydrocortisone whereas GLU22 is making H-bond with hydrocortisone, meloxicam, valdecoxib and prednisolone each.

c. In mutant E22del; GLN15 is interacting with both prednisone and beclomethasone; ALA21 with celecoxib and finally TYR10, GLU11, HIS13 with valdecoxib.

Notably, all these amino acids, TYR10, GLU11, HIS13, GLN15, VAL18, ALA21 & GLU22, were found to play pivotal roles to stabilize the homo-dimers in the respective cases through H-bonding interactions. It was further observed that majority of the amino acid residues forming non-covalent interactions that stabilize the dimerization process were involved in binding with the aforementioned candidate molecules.

## 4 Discussion

Aβoligomers (AβOs) have been established as an important biomolecule that works in the juncture of amyloid pathway and tau phosphorylation [25], [26]; it is of utmost importance to study its structural constituent. Moreover, several studies reported, that Aβ oligomers were mostly made of Aβ* 2 (dimers), Aβ* 3 (trimers) and Aβ* 56 (dodecamers) [27]. Hence, in our study, we tried to analyze the initial step of oligomerization, i.e. dimer formation with the help of peptide-peptide docking simulations. But to do so we would require the monomeric structure of Aβ, which was not readily available; hence we used the available FASTA sequence of APP (Gene ID: 351) from NCBI, which contains the Aβ stretch within it. It must also be noted that, although various lengths of Aβ peptides are present, we chose the 42 amino acid residues long peptide Aβ42 due to its abundance in the sample of amyloid plaques of AD patients [28].

Now for this study, the missense mutations related to fAD were to be identified in the Aβ stretch of APP; 26 pathogenic missense mutations were readily available and reported in various literatures. Interestingly 25 of them were associated with autosomal dominant inheritance; but only one was reported as an autosomal recessive mutation [8]. Thus, to maintain the diversity in the study population, we selected four variants as follows: the Osaka Variant with E22del mutation (it was the first reported variant of all) [29] and no amyloid plaque deposition variant; and another is A2V [30], the only one in case of autosomal recessive variant of AD. The other two variants are - the wild-type variant with no mutation which is solely responsible for sAD and A2T mutation, the autosomal recessive mutation which happens to be a mutation that is cured of AD.

The three-dimensional structures of each monomer were generated by molecular modelling and studied for their stereo-chemical fitness. The dimeric forms of the peptides were built by docking simulations. It was observed that both the WT and the mutant A2V have similar BFEs indicating similar stabilities. The mutant A2T has the least BFE indicating that dimerization of the mutant is comparatively less likely to occur. Therefore, our results are in-agreement with the pathophysiology of AD. Again, as per the existing evidence [30] the mutant E22del does not form plaques but is able to form oligomers. The comparatively higher value of its binding free energy as compared to the cured variant A2T would indicate the same etiology.

Literature evidence shows that Aβ Oligomers obtain an ordered β sheet structure having stacks of parallel β sheets, elongated into amyloid fibrils which aggregate into amyloid plaques, the clinical hallmark of AD [27]; but in the contrary the Aβ monomer’s structure is distributed between some random coil and helix as depicted by the prepared WT monomer model in Fig. 2. However, through the process of dimerization, the WT and A2V mutant could attain β sheet conformation (Supplementary Fig 1). On the other hand, the A2T dimer shows the weakest binding interactions to stabilize itself as shown in Fig. 4. This again proves that indeed A2T mutant is cured of AD, as the dimers are the unstable and most unlikely to be able to oligomerize and therefore inhibit the formation of Aβ Oligomers, which is crucial for the disease pathology [31], [32]. Lastly, the E22del mutant shows an aggregation of the monomers into a cluster that is evident in the Off-pathway where AβOs never forms amyloid fibrils but rather induces neurotoxicity and thereby causes disease propagation [29], [33], [34], [35].

Now, while considering anti-inflammatory drugs we were meticulous in choosing our working dataset that consisted of 40 different drugs with over five thousand literatures related with them (Supplementary Table 1), from which we successfully curated a ligand library consisting of seventeen of those drugs for this study. Our working hypothesis was clear while studying the monomer-drug complexes; the drugs will only be successful if they interact with the residues responsible for the dimer formation thus, preventing the promotion of oligomerization of the Aβ peptides at the initiation step of dimerization [36]. The results could successfully mimic this hypothesis where the interacting residues of the monomers with the selected drugs coincided with those responsible for dimer formation. The binding pockets for the small molecules or ligands were validated using COACH server as well (Supplementary Table 6) which re-confirms the findings. Additionally, it was found, that the drugs tend to make hydrogen bond in the vicinity or with the exactly same amino acid residues responsible for the dimer formations. Our virtual screening experiments with the candidate drugs could affirm the same. Therefore, these well-established anti-inflammatory drugs may serve as the potential drug candidates for the treatment of AD.

## 5 Conclusion

This study gives valuable insight towards understanding of the structural assemblies of Aβ peptides, particularly dimers towards the formation of Aβ Oligomers. Here, a computational approach was employed to predict the interaction site of the dimers; both for WT and mutants. We were successful in identifying the differentiating factors for the formations of the dimers. For instance, we have predicted the individual dimer configurations that coincide with the requirement of disease propagation; the WT dimer and A2V mutant dimer showed structured β-sheet conformations leading to the on pathway of amyloid plaque formation; whereas the heterogeneous conformation of the E22del mutant dimer paved the conformation towards off pathway of neurotoxic AβOs. Our aim is to give a firsthand insight into the structures of Aβ peptide in its native and mutant forms as till date there are no such structures available from any wet-lab experiments. Therefore, we generated their structures through the technique of molecular modeling. Furthermore, we intended to predict the nature of a single inhibitor that might be used to treat AD along with other associated disorders as there is no such information present till date. This is predictive work and the results need verification in wet-lab. However, the results from our analyses might help in streamlining future wet-lab experiments in this matter.

## Ethics approval and consent to participate

Not applicable.

## Funding

The financial support from the Department of Biotechnology (DBT), Government of India is duly acknowledged. The infrastructural support from the DBT-funded Bioinformatics Infrastructure Facility Centre (Project Sanction no: BT/PR40162/BTIS/137/48/2022, dated 31.10.2022) and National Network Project (Project Sanction no: BT/PR40192/BTIS/137/69/2023, dated 19.12.2023), both of which have been sanctioned to Prof. Angshuman Bagchi, were utilized in this work.

## CRediT authorship contribution statement

**Sreekanya Roy:** Writing – original draft, Investigation, Formal analysis, Data curation. **Sima Biswas:** Writing – review & editing. **Anirban Nandy:** Writing – review & editing. **Dipanjan Guha:** Writing – review & editing, Formal analysis. **Angshuman Bagchi:** Writing – review & editing, Validation, Project administration, conceptualization. **Rakhi Dasgupta:** Writing – review & editing. **Parames Chandra Sil:** Writing – review & editing.

## Declaration of Competing Interest

The authors declare that they have no known competing financial interests or personal relationships that could have appeared to influence the work reported in this paper.

## Data Availability

Data will be made available on request.

## References

[1] Lamptey R.N., Chaulagain B., Trivedi R., Gothwal A., Layek B., Singh J. (2022). A review of the common neurodegenerative disorders: current therapeutic approaches and the potential role of nanotherapeutics. Int. J. Mol. Sci..

[2] Hippius H., Neundörfer G. (2003). The discovery of Alzheimer's disease. Dialog-. Clin. Neurosci..

[3] Glenner G.G., Wong C.W. (1984). Alzheimer's disease: initial report of the purification and characterization of a novel cerebrovascular amyloid protein. Biochem. Biophys. Res. Commun..

[4] Armstrong R.A. (2019 Jan 1). Risk factors for Alzheimer’s disease. Folia Neuropathol..

[5] Bekris L.M., Yu C.E., Bird T.D., Tsuang D.W. (2010). Genetics of Alzheimer disease. J. Geriatr. Psychiatry Neurol..

[6] Dorszewska J., Prendecki M., Oczkowska A., Dezor M., Kozubski W. (2016). Molecular basis of familial and sporadic Alzheimer's disease. Curr. Alzheimer Res..

[7] Hatami A., Monjazeb S., Milton S., Glabe C.G. (2017). Familial Alzheimer’s disease mutations within the amyloid precursor protein alter the aggregation and conformation of the amyloid-β peptide. J. Biol. Chem..

[8] Nasb M., Tao W., Chen N. (2024). Alzheimer's disease puzzle: delving into pathogenesis hypotheses. Aging Dis..

[9] O'brien R.J., Wong P.C. (2011). Amyloid precursor protein processing and Alzheimer's disease. Annu. Rev. Neurosci..

[10] Hur J.Y. (2022). γ-Secretase in Alzheimer’s disease. Exp. Mol. Med..

[11] Jurisch-Yaksi N., Sannerud R., Annaert W. (2013). A fast growing spectrum of biological functions of γ-secretase in development and disease. Biochim. Et. Biophys. Acta (BBA)-Biomembr..

[12] Xu T.H., Yan Y., Kang Y., Jiang Y., Melcher K., Xu H.E. (2016). Alzheimer’s disease-associated mutations increase amyloid precursor protein resistance to γ-secretase cleavage and the Aβ42/Aβ40 ratio. Cell Discov..

[13] Selkoe D.J., Hardy J. (2016). The amyloid hypothesis of Alzheimer's disease at 25 years. EMBO Mol. Med..

[14] Laskowski R.A., Rullmann J.A., MacArthur M.W., Kaptein R., Thornton J.M. (1996). AQUA and PROCHECK NMR: programs for checking the quality of protein structures solved by NMR. J. Biomol. NMR.

[15] Tam B., Qin Z., Zhao B., Wang S.M., Lei C.L. (2023). Integration of deep learning with Ramachandran plot molecular dynamics simulation for genetic variant classification. Iscience.

[16] IDesta I.T., Porter K.A., Xia B., Kozakov D., Vajda S. (2020). Performance and its limits in rigid body protein-protein docking. Structure.

[17] Vajda S., Yueh C., Beglov D., Bohnuud T., Mottarella S.E., Xia B., Hall D.R., Kozakov D. (2017). New additions to the C lus P ro server motivated by CAPRI. Protein.: Struct. Funct. Bioinforma..

[18] Kozakov D., Hall D.R., Xia B., Porter K.A., Padhorny D., Yueh C., Beglov D., Vajda S. (2017). The ClusPro web server for protein–protein docking. Nat. Protoc..

[19] Vangone A., Schaarschmidt J., Koukos P., Geng C., Citro N., Trellet M.E., Xue L.C., Bonvin A.M. (2019). Large-scale prediction of binding affinity in protein–small ligand complexes: the PRODIGY-LIG web server. Bioinformatics.

[20] Kurkcuoglu Z., Koukos P.I., Citro N., Trellet M.E., Rodrigues J.P., Moreira I.S., Roel-Touris J., Melquiond A.S., Geng C., Schaarschmidt J., Xue L.C. (2018). Performance of HADDOCK and a simple contact-based protein–ligand binding affinity predictor in the D3R grand challenge 2. J. Comput. -Aided Mol. Des..

[21] Norgan A.P., Coffman P.K., Kocher J.P., Katzmann D.J., Sosa C.P. (2011). Multilevel parallelization of AutoDock 4.2. J. Chemin.-..

[22] Tanchuk V.Y., Tanin V.O., Vovk A.I., Poda G. (2016). A new, improved hybrid scoring function for molecular docking and scoring based on AutoDock and AutoDock Vina. Chem. Biol. Drug Des..

[23] Wallace A.C., Laskowski R.A., Thornton J.M. (1995). LIGPLOT: a program to generate schematic diagrams of protein-ligand interactions. Protein Eng. Des. Sel..

[24] Laskowski R.A., Swindells M.B. LigPlot+: multiple ligand–protein interaction diagrams for drug discovery, 10.1021/ci200227u. 21919503

[25] Forloni G. (2023). Oligomers and neurodegeneration: new evidence. Aging Dis..

[26] Ghosh S., Ali R., Verma S. (2023). Aβ-oligomers: a potential therapeutic target for Alzheimer's disease. Int. J. Biol. Macromol..

[27] Lee S.J., Nam E., Lee H.J., Savelieff M.G., Lim M.H. (2017). Towards an understanding of amyloid-β oligomers: characterization, toxicity mechanisms, and inhibitors. Chem. Soc. Rev..

[28] Lesne S.E., Sherman M.A., Grant M., Kuskowski M., Schneider J.A., Bennett D.A., Ashe K.H. (2013). Brain amyloid-β oligomers in ageing and Alzheimer’s disease. Brain.

[29] Tomiyama T., Shimada H. (2020). APP Osaka mutation in familial Alzheimer’s disease—its discovery, phenotypes, and mechanism of recessive inheritance. Int. J. Mol. Sci..

[30] Li H., Nam Y., Salimi A., Lee J.Y. (2020). Impact of A2V mutation and histidine tautomerism on Aβ42 monomer structures from atomistic simulations. J. Chem. Inf. Model..

[31] Dear A.J., Thacker D., Wennmalm S., Ortigosa-Pascual L., Andrzejewska E.A., Meisl G., Linse S., Knowles T.P. (2024). Aβ oligomer dissociation is catalyzed by fibril surfaces. ACS Chem. Neurosci..

[32] Hampel H., Hardy J., Blennow K., Chen C., Perry G., Kim S.H., Villemagne V.L., Aisen P., Vendruscolo M., Iwatsubo T., Masters C.L. (2021). The amyloid-β pathway in Alzheimer’s disease. Mol. Psychiatry.

[33] Chiti F., Dobson C.M. (2017). Protein misfolding, amyloid formation, and human disease: a summary of progress over the last decade. Annu. Rev. Biochem..

[34] Tolar M., Hey J., Power A., Abushakra S. (2021). Neurotoxic soluble amyloid oligomers drive Alzheimer’s pathogenesis and represent a clinically validated target for slowing disease progression. Int. J. Mol. Sci..

[35] Abedin F., Kandel N., Tatulian S.A. (2021). Effects of Aβ-derived peptide fragments on fibrillogenesis of Aβ. Sci. Rep..

[36] Papadopoulos N., Suelves N., Perrin F., Vadukul D.M., Vrancx C., Constantinescu S.N., Kienlen-Campard P. (2022). Structural determinant of β-amyloid formation: from transmembrane protein dimerization to β-amyloid aggregates. Biomedicines.

