# Supplementary material for: An approach to predict and inhibit Amyloid Beta dimerization pattern in Alzheimer’s disease
Source: Toxicol Rep. 2024 Dec 28;14:101879. doi: 10.1016/j.toxrep.2024.101879 (PMC11762949; doi:10.1016/j.toxrep.2024.101879)
Supplement: Supplementary file 1 — Supplementary material [file mmc1.doc]

**Supplementary Figure 1.**

**
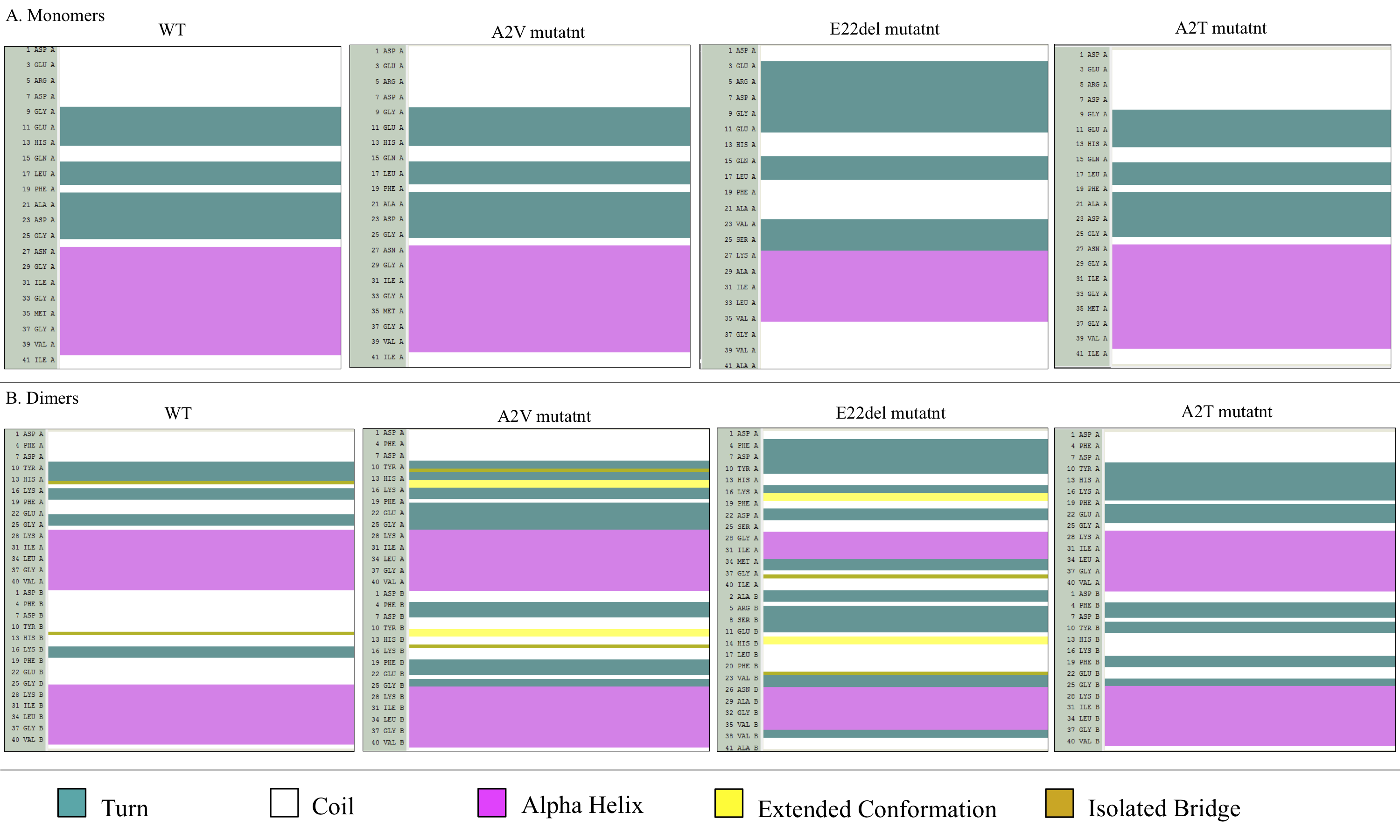
**

**Distribution of secondary structure**. The distribution of secondary structure of the selected monomers (upper panel) and dimmers (lower panel) are shown with the specific colour coding.
