# Supplementary material for: An approach to predict and inhibit Amyloid Beta dimerization pattern in Alzheimer’s disease
Source: Toxicol Rep. 2024 Dec 28;14:101879. doi: 10.1016/j.toxrep.2024.101879 (PMC11762949; doi:10.1016/j.toxrep.2024.101879)
Supplement: Supplementary file 2 — Supplementary material [file mmc2.docx]

**Supplementary Files**

| cid | cmpdname | mw | mf | polararea | complexity | xlogp | heavycnt | hbonddonor | hbondacc | rotbonds |
| --- | --- | --- | --- | --- | --- | --- | --- | --- | --- | --- |
| 3394 | Flurbiprofen | 244.26 | C15H13FO2 | 37.3 | 286 | 4.2 | 18 | 1 | 3 | 3 |
| 54676228 | Piroxicam | 331.3 | C15H13N3O4S | 108 | 611 | 3.1 | 23 | 2 | 6 | 2 |
| 3672 | Ibuprofen | 206.28 | C13H18O2 | 37.3 | 203 | 3.5 | 15 | 1 | 2 | 4 |
| 3826 | Ketorolac | 255.27 | C15H13NO3 | 59.3 | 376 | 1.9 | 19 | 1 | 3 | 3 |
| 5754 | Hydrocortisone | 362.5 | C21H30O5 | 94.8 | 684 | 1.6 | 26 | 3 | 5 | 2 |
| 5755 | Prednisolone | 360.4 | C21H28O5 | 94.8 | 724 | 1.6 | 26 | 3 | 5 | 2 |
| 5281004 | Budesonide | 430.5 | C25H34O6 | 93.1 | 862 | 2.5 | 31 | 2 | 6 | 4 |
| 4037 | Meclofenamic acid | 296.1 | C14H11Cl2NO2 | 49.3 | 327 | 5.2 | 19 | 2 | 3 | 3 |
| 4495 | Nimesulide | 308.31 | C13H12N2O5S | 110 | 450 | 2.6 | 21 | 1 | 6 | 4 |
| 68869 | Flunoxaprofen | 285.27 | C16H12FNO3 | 63.3 | 384 | 3.6 | 21 | 1 | 5 | 3 |
| 667550 | Dexketoprofen | 254.28 | C16H14O3 | 54.4 | 331 | 3.1 | 19 | 1 | 3 | 4 |
| 1981 | Acemetacin | 415.8 | C21H18ClNO6 | 94.8 | 620 | 4.2 | 29 | 1 | 6 | 7 |
| 2097 | Alminoprofen | 219.28 | C13H17NO2 | 49.3 | 255 | 3.2 | 16 | 2 | 3 | 5 |
| 2662 | Celecoxib | 381.4 | C17H14F3N3O2S | 86.4 | 577 | 3.4 | 26 | 1 | 7 | 3 |
| 3308 | Etodolac | 287.35 | C17H21NO3 | 62.3 | 400 | 2.8 | 21 | 2 | 3 | 4 |
| 3335 | Fenbufen | 254.28 | C16H14O3 | 54.4 | 310 | 3.2 | 19 | 1 | 3 | 5 |
| 3718 | Indoprofen | 281.3 | C17H15NO3 | 57.6 | 414 | 2.8 | 21 | 1 | 3 | 3 |
| 4075 | Mesalamine | 153.14 | C7H7NO3 | 83.6 | 160 | 1.3 | 11 | 3 | 4 | 1 |
| 4409 | Nabumetone | 228.29 | C15H16O2 | 26.3 | 262 | 3.1 | 17 | 0 | 2 | 4 |
| 4614 | Oxaprozin | 293.3 | C18H15NO3 | 63.3 | 361 | 4.2 | 22 | 1 | 4 | 5 |
| 4888 | Pranoprofen | 255.27 | C15H13NO3 | 59.4 | 346 | 2.7 | 19 | 1 | 4 | 2 |
| 4921 | Proglumetacin | 844.4 | C46H58ClN5O8 | 140 | 1360 | 7.3 | 60 | 1 | 10 | 23 |
| 5090 | Rofecoxib | 314.4 | C17H14O4S | 68.8 | 556 | 2.3 | 22 | 0 | 4 | 3 |
| 5339 | Sulfasalazine | 398.4 | C18H14N4O5S | 150 | 657 | -0.7 | 28 | 3 | 9 | 6 |
| 5359 | Suprofen | 260.31 | C14H12O3S | 82.6 | 321 | 3.3 | 18 | 1 | 4 | 4 |
| 5733 | Zomepirac | 291.73 | C15H14ClNO3 | 59.3 | 379 | 2.8 | 20 | 1 | 3 | 4 |
| 5743 | Dexamethasone | 392.5 | C22H29FO5 | 94.8 | 805 | 1.9 | 28 | 3 | 6 | 2 |
| 5865 | Prednisone | 358.4 | C21H26O5 | 91.7 | 764 | 1.5 | 26 | 2 | 5 | 2 |
| 22419 | Olsalazine | 302.24 | C14H10N2O6 | 140 | 415 | 3.1 | 22 | 4 | 8 | 4 |
| 26248 | Diacerein | 368.3 | C19H12O8 | 124 | 683 | 1.9 | 27 | 1 | 8 | 5 |
| 31508 | Proquazone | 278.3 | C18H18N2O | 32.7 | 423 | 3 | 21 | 0 | 1 | 2 |
| 35935 | Pirprofen | 251.71 | C13H14ClNO2 | 40.5 | 311 | 2.9 | 17 | 1 | 3 | 3 |
| 39912 | Dexibuprofen | 206.28 | C13H18O2 | 37.3 | 203 | 3.5 | 15 | 1 | 2 | 4 |
| 39941 | Benoxaprofen | 301.72 | C16H12ClNO3 | 63.3 | 384 | 4.1 | 21 | 1 | 4 | 3 |
| 54585 | Balsalazide | 357.32 | C17H15N3O6 | 149 | 545 | 2.2 | 26 | 4 | 8 | 7 |
| 60712 | Tenidap | 320.8 | C14H9ClN2O3S | 114 | 450 | 3.8 | 21 | 2 | 4 | 2 |
| 60726 | Bromfenac | 334.16 | C15H12BrNO3 | 80.4 | 366 | 3.3 | 20 | 2 | 4 | 4 |
| 65679 | Droxicam | 357.3 | C16H11N3O5S | 105 | 741 | 1.2 | 25 | 0 | 7 | 1 |
| 68704 | Ibuproxam | 221.29 | C13H19NO2 | 49.3 | 220 | 2.8 | 16 | 2 | 2 | 4 |
| 68706 | Lonazolac | 312.7 | C17H13ClN2O2 | 55.1 | 379 | 3.6 | 22 | 1 | 3 | 4 |
| 100472 | Difenpiramide | 288.3 | C19H16N2O | 42 | 343 | 3.8 | 22 | 1 | 2 | 4 |
| 119607 | Valdecoxib | 314.4 | C16H14N2O3S | 94.6 | 462 | 2.6 | 22 | 1 | 5 | 3 |
| 119828 | Parecoxib | 370.4 | C19H18N2O4S | 97.6 | 575 | 3.3 | 26 | 1 | 5 | 5 |
| 123619 | Etoricoxib | 358.8 | C18H15ClN2O2S | 68.3 | 514 | 3.3 | 24 | 0 | 4 | 3 |
| 151166 | Lumiracoxib | 293.72 | C15H13ClFNO2 | 49.3 | 342 | 4.2 | 20 | 2 | 4 | 4 |
| 9884642 | Naproxcinod | 347.4 | C18H21NO6 | 90.6 | 438 | 4.1 | 25 | 0 | 6 | 9 |
| 54677470 | Meloxicam | 351.4 | C14H13N3O4S2 | 136 | 628 | 3 | 23 | 2 | 7 | 2 |
| 54677971 | Tenoxicam | 337.4 | C13H11N3O4S2 | 136 | 599 | 1.1 | 22 | 2 | 7 | 2 |
| 54690031 | Lornoxicam | 371.8 | C13H10ClN3O4S2 | 136 | 634 | 2.1 | 23 | 2 | 7 | 2 |
| 3332 | Felbinac | 212.24 | C14H12O2 | 37.3 | 223 | 3 | 16 | 1 | 2 | 3 |
| 3965 | Loxoprofen | 246.3 | C15H18O3 | 54.4 | 316 | 2.4 | 18 | 1 | 3 | 4 |
| 656604 | Bucillamine | 223.3 | C7H13NO3S2 | 68.4 | 218 | 0.4 | 13 | 4 | 5 | 4 |
| 5280965 | Amphotericin B | 924.1 | C47H73NO17 | 320 | 1670 | 0 | 65 | 12 | 18 | 3 |
| 5311412 | Rimexolone | 370.5 | C24H34O3 | 54.4 | 749 | 3.5 | 27 | 1 | 3 | 2 |
| 6436173 | Rifaximin | 785.9 | C43H51N3O11 | 198 | 1590 | 6.9 | 57 | 5 | 12 | 3 |
| 6917992 | (1S,2R,18R,19R,22S,25R,28R,40R)-48-[(2S,3R,4S,5S,6R)-3-[(2S,4S,5S,6S)-4-amino-5-hydroxy-4,6-dimethyloxan-2-yl]oxy-4,5-dihydroxy-6-(hydroxymethyl)oxan-2-yl]oxy-22-(2-amino-2-oxoethyl)-5,15-dichloro-2,18,32,35,37-pentahydroxy-19-[[(2R)-4-methyl-2-(methylamino)pentanoyl]amino]-20,23,26,42,44-pentaoxo-7,13-dioxa-21,24,27,41,43-pentazaoctacyclo[26.14.2.23,6.214,17.18,12.129,33.010,25.034,39]pentaconta-3,5,8(48),9,11,14,16,29(45),30,32,34(39),35,37,46,49-pentadecaene-40-carboxylic acid | 1449.2 | C66H75Cl2N9O24 | 531 | 2960 | -2.6 | 101 | 19 | 26 | 13 |
| 6918453 | Ridauran | 678.5 | C20H34AuO9PS | 115 | 538 | NULL | 32 | 0 | 10 | 12 |
| 9571044 | Nifurzide | 336.28 | C12H8N4O6S | 175 | 529 | 2.7 | 23 | 1 | 8 | 4 |
| 10034073 | Fidaxomicin | 1058 | C52H74Cl2O18 | 267 | 1970 | 6.4 | 72 | 7 | 18 | 15 |
| 9782 | Betamethasone | 392.5 | C22H29FO5 | 94.8 | 805 | 1.9 | 28 | 3 | 6 | 2 |
| 2882 | Cromolyn | 468.4 | C23H16O11 | 166 | 835 | 1.9 | 34 | 3 | 11 | 8 |
| 20469 | Beclomethasone | 408.9 | C22H29ClO5 | 94.8 | 805 | 2.2 | 28 | 3 | 5 | 2 |
| 31307 | Triamcinolone | 394.4 | C21H27FO6 | 115 | 807 | 1.2 | 28 | 4 | 7 | 2 |
| 6324616 | Rifamycin | 697.8 | C37H47NO12 | 201 | 1330 | 4.9 | 50 | 6 | 12 | 3 |
| 71751 | Prifelone | 316.5 | C19H24O2S | 65.5 | 381 | 6.3 | 22 | 1 | 3 | 4 |
| 54677972 | Isoxicam | 335.34 | C14H13N3O5S | 121 | 627 | 3 | 23 | 2 | 7 | 2 |
|  |  |  |  |  |  |  |  |  |  |  |
| Red | Discarded |  |  |  |  |  |  |  |  |  |

**Supplementary File 1**

| Antiinflammatory Drugs | Paper Names |
| --- | --- |
| Flurbiprofen | Novel flurbiprofen derivatives with improved brain delivery: synthesis, in vitro and in vivo evaluations |
|  | Nanoparticulate flurbiprofen reduces amyloid-β42 generation in an in vitro blood-brain barrier model |
|  | Role of serum albumin as a nanoparticulate carrier for nose-to-brain delivery of R-flurbiprofen: implications for the treatment of Alzheimer's disease |
|  | Solubilization of flurbiprofen into aptamer-modified PEG-PLA micelles for targeted delivery to brain-derived endothelial cells in vitro |
|  | R-flurbiprofen attenuates experimental autoimmune encephalomyelitis in mice |
|  | Absorption kinetics of flurbiprofen axetil microspheres in cerebrospinal fluid: A pilot study |
|  | Inhibition of endocannabinoid metabolism by the metabolites of ibuprofen and flurbiprofen |
|  | Flurbiprofen axetil attenuates cerebral ischemia/reperfusion injury by reducing inflammation in a rat model of transient global cerebral ischemia/reperfusion |
|  | Lipid nanocarriers containing ester prodrugs of flurbiprofen preparation, physical-chemical characterization and biological studies |
|  | R-flurbiprofen improves tau, but not Aß pathology in a triple transgenic model of Alzheimer's disease |
|  | Nasal NSAIDs for Alzheimer's Disease |
|  | Design, synthesis and evaluation of 4'-OH-flurbiprofen-chalcone hybrids as potential multifunctional agents for Alzheimer's disease treatment |
|  | Intranasal delivery of nanoparticle encapsulated tarenflurbil: A potential brain targeting strategy for Alzheimer's disease |
|  | Design, synthesis and evaluation of tacrine-flurbiprofen-nitrate trihybrids as novel anti-Alzheimer's disease agents |
|  | New flurbiprofen derivatives: synthesis, membrane affinity and evaluation of in vitro effect on β-amyloid levels |
|  | [The Mechanisms and Pharmacological Strategy for Treatment of ER Stress-induced Metabolic Syndrome] |
|  | Efficacy of Nalbuphine with Flurbiprofen on Multimodal Analgesia with Transverse Abdominis Plane Block in Elderly Patients Undergoing Open Gastrointestinal Surgery: A Randomized, Controlled, Double-Blinded Trial |
|  | Inhibition of fatty acid amide hydrolase and cyclooxygenase by the N-(3-methylpyridin-2-yl)amide derivatives of flurbiprofen and naproxen |
|  | Interaction of the N-(3-Methylpyridin-2-yl)amide Derivatives of Flurbiprofen and Ibuprofen with FAAH: Enantiomeric Selectivity and Binding Mode |
|  | Combination therapy in a transgenic model of Alzheimer's disease |
|  | Aging Enables Ca2+ Overload and Apoptosis Induced by Amyloid-β Oligomers in Rat Hippocampal Neurons: Neuroprotection by Non-Steroidal Anti-Inflammatory Drugs and R-Flurbiprofen in Aging Neurons |
|  | Anandamide deficiency and heightened neuropathic pain in aged mice |
|  | Improved l-Type amino acid transporter 1 (LAT1)-mediated delivery of anti-inflammatory drugs into astrocytes and microglia with reduced prostaglandin production |
|  | ASIC1a activation enhances inhibition in the basolateral amygdala and reduces anxiety |
|  | Electrophysiological and metabolic effects of CHF5074 in the hippocampus: protection against in vitro ischemia |
|  | Gamma-Secretase Inhibitors Attenuate Neurotrauma and Neurogenic Acute Lung Injury in Rats by Rescuing the Accumulation of Hypertrophic Microglia |
|  | Characterisation of (R)-2-(2-Fluorobiphenyl-4-yl)-N-(3-Methylpyridin-2-yl)Propanamide as a Dual Fatty Acid Amide Hydrolase: Cyclooxygenase Inhibitor |
|  | Neuroprotective and Anti-Apoptotic Effects of CSP-1103 in Primary Cortical Neurons Exposed to Oxygen and Glucose Deprivation |
|  | Pharmacokinetics and pharmacodynamics of CHF5074 after short-term administration in healthy subjects |
|  | CHF5074 restores visual memory ability and pre-synaptic cortical acetylcholine release in pre-plaque Tg2576 mice |
|  | CHF5074 and LY450139 sub-acute treatments differently affect cortical extracellular glutamate levels in pre-plaque Tg2576 mice |
|  | CHF5074 protects SH-SY5Y human neuronal-like cells from amyloidbeta 25-35 and tumor necrosis factor related apoptosis inducing ligand toxicity in vitro |
|  | Multi-target action of the novel anti-Alzheimer compound CHF5074: in vivo study of long term treatment in Tg2576 mice |
| Piroxicam | Piroxicam attenuates 3-nitropropionic acid-induced brain oxidative stress and behavioral alteration in mice |
|  | Piroxicam: Source for Synthesis of Central Nervous System (CNS) Acting Drugs |
|  | Piroxicam-mediated modulatory action of 5-hydroxytryptamine serves as a "brake" on neuronal excitability in ischemic stroke |
|  | Aquaporin-4 inhibition mediates piroxicam-induced neuroprotection against focal cerebral ischemia/reperfusion injury in rodents |
|  | Genetics and genomics in postoperative pain and analgesia |
|  | Alleviation of glutamate mediated neuronal insult by piroxicam in rodent model of focal cerebral ischemia: a possible mechanism of GABA agonism |
|  | The effects of lornoxicam on brain edema and blood brain barrier following diffuse traumatic brain injury in rats |
|  | Piroxicam confer neuroprotection in Cerebral Ischemia by inhibiting Cyclooxygenases, Acid- Sensing Ion Channel-1a and Aquaporin-4: an in silico comparison with Aspirin and Nimesulide |
|  | Effects of piroxicam on tissue distribution of sulfadimidine in West African Dwarf male and female goats |
|  | Ibuprofen or piroxicam protects nigral neurons and delays the development of l-dopa induced dyskinesia in rats with experimental Parkinsonism: Influence on angiogenesis |
|  | Inhibition of matrix metalloproteinase-2 and 9 by Piroxicam confer neuroprotection in cerebral ischemia: an in silico evaluation of the hypothesis |
|  | Does Piroxicam really protect ischemic neurons and influence neuronal firing in cerebral ischemia? An exploration towards therapeutics |
|  | Transport rankings of non-steroidal antiinflammatory drugs across blood-brain barrier in vitro models |
|  | Intracerebroventricular injections of endotoxin (ET) reduces hippocampal neurogenesis |
|  | The effects of lornoxicam on neuroprotection following diffuse traumatic brain injury in rats |
|  | Increased generation of cyclopentenone prostaglandins after brain ischemia and their role in aggregation of ubiquitinated proteins in neurons |
|  | Molecular cloning and characterization of a new multispecific organic anion transporter from rat brain |
|  | Inflammation-induced functional connectivity of melanin-concentrating hormone and IL-10 |
|  | Influence of NSAIDs and methotrexate on CD73 expression and glioma cell growth |
|  | Antinociceptive tolerance to NSAIDs microinjected into dorsal hippocampus |
|  | Electrophysiological signatures of acute systemic lipopolysaccharide-induced inflammation: potential implications for delirium science |
|  | Antinociceptive tolerance to NSAIDs in the anterior cingulate cortex is mediated via endogenous opioid mechanism |
|  | Intra-hippocampal injection of lipopolysaccharide inhibits kindled seizures and retards kindling rate in adult rats |
|  | Determining the Molecular Pathways Underlying the Protective Effect of Non-Steroidal Anti-Inflammatory Drugs for Alzheimer's Disease: A Bioinformatics Approach |
|  | Risk of ischemic stroke and the use of individual non-steroidal anti-inflammatory drugs: A multi-country European database study within the SOS Project |
| Ibuprofen | The epidemiology of Parkinson's disease: risk factors and prevention |
|  | Cyclooxygenases and the cardiovascular system |
|  | Physical stability and in vivo brain delivery of polymeric ibuprofen nanoparticles fabricated by flash nanoprecipitation |
|  | Therapeutic potential to reduce brain injury in growth restricted newborns |
|  | Dual-targeting for brain-specific drug delivery: synthesis and biological evaluation |
|  | Design, synthesis and biological evaluation of brain targeting l-ascorbic acid prodrugs of ibuprofen with "lock-in" function |
|  | Paracetamol (acetaminophen) or non-steroidal anti-inflammatory drugs, alone or combined, for pain relief in acute otitis media in children |
|  | Altitude headache |
|  | Andrology: Ibuprofen and hypogonadism - bench to bedside to misinterpreted hype? |
|  | Reactive oxygen species-activated nanoprodrug of Ibuprofen for targeting traumatic brain injury in mice |
|  | Ibuprofen ameliorates fatigue- and depressive-like behavior in tumor-bearing mice |
|  | Ibuprofen does not reverse ventilatory acclimatization to chronic hypoxia |
|  | The Pharmacology of Indomethacin |
|  | Effect of Ibuprofen on BrainAGE: A Randomized, Placebo-Controlled, Dose-Response Exploratory Study |
|  | The effects of aging, housing and ibuprofen treatment on brain neurochemistry in a triple transgene Alzheimer's disease mouse model using magnetic resonance spectroscopy and imaging |
|  | Effect of ibuprofen exposure on blood, gill, liver, and brain on common carp (Cyprinus carpio) using oxidative stress biomarkers |
|  | Cerebroprotective effects of ibuprofen on diabetic encephalopathy in rats |
|  | Ibuprofen-based advanced therapeutics: breaking the inflammatory link in cancer, neurodegeneration, and diseases |
|  | Inhibition of endocannabinoid metabolism by the metabolites of ibuprofen and flurbiprofen |
|  | Ibuprofen Blunts Ventilatory Acclimatization to Sustained Hypoxia in Humans |
|  | Ibuprofen partially attenuates neurodegenerative symptoms in presenilin conditional double-knockout mice |
|  | Ibuprofen rescues abnormalities in periodontal tissues in conditional presenilin 1 and presenilin 2 double knockout mice |
|  | Rapid Ascent to High Altitude: Acetazolamide or Ibuprofen? |
|  | Effects of low concentrations of ibuprofen on freshwater fish Rhamdia quelen |
|  | Ibuprofen targets neuronal pentraxins expresion and improves cognitive function in mouse model of AlCl(3)-induced neurotoxicity |
|  | Effects of ibuprofen on cognition and NMDA receptor subunit expression across aging |
|  | Deep brain stimulation electrode insertion and depression: Patterns of activity and modulation by analgesics |
|  | Peripherally triggered and GSK-3β-driven brain inflammation differentially skew adult hippocampal neurogenesis, behavioral pattern separation and microglial activation in response to ibuprofen |
|  | Ibuprofen Protects from Cypermethrin-Induced Changes in the Striatal Dendritic Length and Spine Density |
|  | Mechanism of brain targeting by dexibuprofen prodrugs modified with ethanolamine-related structures |
|  | Posttraumatic headache in pediatrics: an update and review |
|  | Amidated and Ibuprofen-Conjugated Kyotorphins Promote Neuronal Rescue and Memory Recovery in Cerebral Hypoperfusion Dementia Model |
|  | Aneurysmal subarachnoid haemorrhage-cerebral vasospasm and prophylactic ibuprofen: a randomised controlled pilot trial protocol |
|  | The use of ibuprofen and acetaminophen for acute headache in the postconcussive youth: A pilot study |
|  | Impact of ibuprofen and peroxisome proliferator-activated receptor gamma on emotion-related neural activation: A randomized, placebo-controlled trial |
|  | Ibuprofen inhibits the synaptic failure induced by the amyloid-β peptide in hippocampal neurons |
|  | High-altitude headache and acute mountain sickness |
|  | Morphine-induced hearing loss |
|  | Pharmacological Potential of the Endogenous Dipeptide Kyotorphin and Selected Derivatives |
|  | Novel Treatments in Neuroprotection for Aneurysmal Subarachnoid Hemorrhage |
|  | Prophylactic Acetaminophen or Ibuprofen Results in Equivalent Acute Mountain Sickness Incidence at High Altitude: A Prospective Randomized Trial |
|  | Antidepressant and antioxidative effect of Ibuprofen in the rotenone model of Parkinson's disease |
|  | Ibuprofen suppresses depressive like behavior induced by BCG inoculation in mice: role of nitric oxide and prostaglandin |
|  | The effect of nC₆₀ on tissue distribution of ibuprofen in Cyprinus carpio |
|  | Long-term exposure to environmentally relevant concentrations of ibuprofen and aluminum alters oxidative stress status on Danio rerio |
|  | Paracetamol, Ibuprofen, and Recurrent Major Cardiovascular and Major Bleeding Events in 19 120 Patients With Recent Ischemic Stroke |
|  | Transport rankings of non-steroidal antiinflammatory drugs across blood-brain barrier in vitro models |
|  | Preparation, transportation mechanisms and brain-targeting evaluation in vivo of a chemical delivery system exploiting the blood-cerebrospinal fluid barrier |
|  | Stereoselective effects of ibuprofen in adult zebrafish (Danio rerio) using UPLC-TOF/MS-based metabolomics |
|  | Potential neuroprotective effect of ibuprofen, insights from the mice model of Parkinson's disease |
|  | Subacute ibuprofen treatment rescues the synaptic and cognitive deficits in advanced-aged mice |
|  | Ibuprofen or piroxicam protects nigral neurons and delays the development of l-dopa induced dyskinesia in rats with experimental Parkinsonism: Influence on angiogenesis |
|  | The novel agent phospho-glycerol-ibuprofen-amide (MDC-330) inhibits glioblastoma growth in mice: an effect mediated by cyclin D1 |
|  | Nasal NSAIDs for Alzheimer's Disease |
|  | Co-Administration of Gagam-Sipjeondaebo-Tang and Ibuprofen Alleviates the Inflammatory Response in MPTP-Induced Parkinson's Disease Mouse Model and RAW264.7 Macrophages |
|  | Enantioselective analysis of ibuprofen enantiomers in mice plasma and tissues by high-performance liquid chromatography with fluorescence detection: Application to a pharmacokinetic study |
|  | ROS-responsive and multifunctional anti-Alzheimer prodrugs: Tacrine-ibuprofen hybrids via a phenyl boronate linker |
|  | Acute over-the-counter pharmacological intervention does not adversely affect behavioral outcome following diffuse traumatic brain injury in the mouse |
|  | Pediatric migraine and episodic syndromes that may be associated with migraine |
|  | Design and evaluation of mucoadhesive microemulsion for neuroprotective effect of ibuprofen following intranasal route in the MPTP mice model |
|  | Why Fever Phobia Is Still Common? |
|  | Effects of Exposure to Acetaminophen and Ibuprofen on Fetal Germ Cell Development in Both Sexes in Rodent and Human Using Multiple Experimental Systems |
|  | Effects of prophylactic oral ibuprofen on the closure rate of patent ductus arteriosus in premature infants |
|  | Patent Ductus Arteriosus in the Preterm Infant: Diagnostic and Treatment Options |
|  | Sunitinib DDI with paracetamol, diclofenac, mefenamic acid and ibuprofen shows sex-divergent effects on the tissue uptake and distribution pattern of sunitinib in mice |
|  | Short-term exposure and long-term consequences of neonatal exposure to Δ(9)-tetrahydrocannabinol (THC) and ibuprofen in mice |
|  | Monoacylglycerol Lipase Regulates Fever Response |
|  | Increased Cytokines at High Altitude: Lack of Effect of Ibuprofen on Acute Mountain Sickness, Physiological Variables, or Cytokine Levels |
|  | Comparison of the Mortality and In-Hospital Outcomes of Preterm Infants Treated with Ibuprofen for Patent Ductus Arteriosus with or without Clinical Symptoms Attributable to the Patent Ductus Arteriosus at the Time of Ibuprofen Treatment |
|  | Reconsideration of the diagnosis and treatment of childhood migraine: A practical review of clinical experiences |
|  | Effects of systemic administration of ibuprofen on stress response in a rat model of post-traumatic stress disorder |
|  | Ibuprofen and lipoic acid conjugate neuroprotective activity is mediated by Ngb/Akt intracellular signaling pathway in Alzheimer's disease rat model |
|  | Longer analgesic effect with naproxen sodium than ibuprofen in post-surgical dental pain: a randomized, double-blind, placebo-controlled, single-dose trial |
|  | [Usefulness of brain natriuretic propeptide in the diagnosis and management of patent ductus arteriosus] |
|  | Pharmacological effects of Ibuprofen on learning and memory, muscarinic receptors gene expression and APP isoforms level in pre-frontal cortex of AlCl₃-induced toxicity mouse model |
|  | Predictive Value of Blood N-Terminal Pro-Brain Natriuretic Peptide Concentrations for Early Patent Ductus Closure in Very Preterm Infants |
|  | Zebrafish is a predictive model for identifying compounds that protect against brain toxicity in severe acute organophosphorus intoxication |
|  | Development of nitroxide-based theranostic compounds that act both as anti-inflammatory drugs and brain redox imaging probes in MRI |
|  | Alpha-synuclein oligomers impair memory through glial cell activation and via Toll-like receptor 2 |
|  | Letter by Iannuccelli et al Regarding Article, "Paracetamol, Ibuprofen, and Recurrent Major Cardiovascular and Major Bleeding Events in 19 120 Patients With Recent Ischemic Stroke" |
|  | Interaction of the N-(3-Methylpyridin-2-yl)amide Derivatives of Flurbiprofen and Ibuprofen with FAAH: Enantiomeric Selectivity and Binding Mode |
|  | Improved neuroprotection using miglustat, curcumin and ibuprofen as a triple combination therapy in Niemann-Pick disease type C1 mice |
|  | Evidence of the impact of systemic inflammation on neuroinflammation from a non-bacterial endotoxin animal model |
|  | Effect of acute intermittent hypoxia on motor function in individuals with chronic spinal cord injury following ibuprofen pretreatment: A pilot study |
|  | Immobilized Artificial Membrane HPLC Derived Parameters vs PAMPA-BBB Data in Estimating in Situ Measured Blood-Brain Barrier Permeation of Drugs |
|  | Bioactivity of albumins bound to silver nanoparticles |
|  | SCISSOR-Spinal Cord Injury Study on Small molecule-derived Rho inhibition: a clinical study protocol |
|  | Analgesia in Neurocritical Care: An International Survey and Practice Audit |
|  | Vulnerability of marsh frog Pelophylax ridibundus to the typical wastewater effluents ibuprofen, triclosan and estrone, detected by multi-biomarker approach |
|  | Electroencephalographic signatures of pain and analgesia in rats |
|  | Cromolyn Reduces Levels of the Alzheimer's Disease-Associated Amyloid β-Protein by Promoting Microglial Phagocytosis |
|  | Onset of analgesia and efficacy of ibuprofen sodium in postsurgical dental pain: a randomized, placebo-controlled study versus standard ibuprofen |
|  | A study on neuroinflammation and NMDA receptor function in STZ (ICV) induced memory impaired rats |
|  | Management of sport-related concussion: a review |
|  | Effect and reporting bias of RhoA/ROCK-blockade intervention on locomotor recovery after spinal cord injury: a systematic review and meta-analysis |
|  | New potential strategies for Alzheimer's disease prevention: pegylated biodegradable dexibuprofen nanospheres administration to APPswe/PS1dE9 |
|  | Impact of patent ductus arteriosus and subsequent therapy with ibuprofen on the release of S-100B and oxidative stress index in preterm infants |
|  | Identification and modification of amyloid-independent phenotypes of APOE4 mice |
|  | Dexibuprofen prevents neurodegeneration and cognitive decline in APPswe/PS1dE9 through multiple signaling pathways |
|  | Influence of NSAIDs and methotrexate on CD73 expression and glioma cell growth |
|  | Analgesia effect of baicalein against NTG-induced migraine in rats |
|  | Management of Pediatric Post-Concussion Headaches: National Survey of Abortive Therapies Used in the Emergency Department |
|  | Diclofenac enhances proinflammatory cytokine-induced aquaporin-4 expression in cultured astrocyte |
|  | Cyclooxygenase inhibition targets neurons to prevent early behavioural decline in Alzheimer's disease model mice |
|  | Evaluating the quality of online information about concussions |
|  | Pharmacological characterization of intraplantar Complete Freund's Adjuvant-induced burrowing deficits |
|  | (18)F-DPA-714 PET Imaging for Detecting Neuroinflammation in Rats with Chronic Hepatic Encephalopathy |
|  | Interventions for preventing high altitude illness: Part 1. Commonly-used classes of drugs |
|  | Mitigation of postnatal ethanol-induced neuroinflammation ameliorates trace fear memory deficits in juvenile rats |
|  | Insight into the Dissociation of Behavior from Histology in Synucleinopathies and in Related Neurodegenerative Diseases |
|  | Analgesic effects of 1,2,3,4,6-penta-O-galloyl-β-D-glucose in an animal model of lipopolysaccharide-induced pain |
|  | Peripheral surgical wounding and age-dependent neuroinflammation in mice |
|  | The mediation of central cyclooxygenase and lipoxygenase pathways in orexin-induced cardiovascular effects |
|  | Effects of non-steroidal anti-inflammatory drugs on hormones and genes of the hypothalamic-pituitary-gonad axis, and reproduction of zebrafish |
|  | Eyelid Edema: A Rare Cause of a Common Sign |
|  | Increased renal sodium absorption by inhibition of prostaglandin synthesis during fasting in healthy man. A possible role of the epithelial sodium channels |
|  | The kynurenine pathway: a missing piece in the puzzle of valproate action? |
|  | Postoperative analgesia for pediatric craniotomy patients: a randomized controlled trial |
|  | Alzheimer's Disease and Intranasal Fluticasone Propionate in the FDA MedWatch Adverse Events Database |
|  | Improved l-Type amino acid transporter 1 (LAT1)-mediated delivery of anti-inflammatory drugs into astrocytes and microglia with reduced prostaglandin production |
|  | A leptin-mediated central mechanism in analgesia-enhanced opioid reward in rats |
|  | Disambiguating Pharmacodynamic Efficacy from Behavior with Neuroimaging: Implications for Analgesic Drug Development |
|  | Indomethacin inhibits tetrodotoxin-resistant Na(+) channels at acidic pH in rat nociceptive neurons |
|  | Acute Agitation and Self-Injury in a 5-Year Old with Autism |
|  | Plant Natural Product Puerarin Ameliorates Depressive Behaviors and Chronic Pain in Mice with Spared Nerve Injury (SNI) |
|  | Acute Agitation and Self-Injury in a 5-Year Old with Autism |
|  | Effects of Bai-Hu decoction on fever induced by lipopolysaccharide |
|  | Synthesis and biological evaluation of novel 8- substituted sampangine derivatives as potent inhibitor of Zn(2+)-Aβ complex mediated toxicity, oxidative stress and inflammation |
|  | Nonsteroidal anti-inflammatory drug choice and adverse outcomes in clopidogrel users: A retrospective cohort study |
|  | The intermediary role of the central cyclooxygenase / lipoxygenase enzymes in intracerebroventricular injected nesfatin-1-evoked cardiovascular effects in rats |
|  | The screening of everyday life chemicals in validated assays targeting the pituitary-gonadal axis |
|  | Prostaglandin D2/J2 signaling pathway in a rat model of neuroinflammation displaying progressive parkinsonian-like pathology: potential novel therapeutic targets |
|  | Neuroprotective and Anti-Apoptotic Effects of CSP-1103 in Primary Cortical Neurons Exposed to Oxygen and Glucose Deprivation |
|  | A Multicriteria Decision Analysis Comparing Pharmacotherapy for Chronic Neuropathic Pain, Including Cannabinoids and Cannabis-Based Medical Products |
|  | Cyclooxygenase-1-dependent prostaglandins mediate susceptibility to systemic inflammation-induced acute cognitive dysfunction |
|  | The expression levels of prolyl oligopeptidase responds not only to neuroinflammation but also to systemic inflammation upon liver failure in rat models and cirrhotic patients |
|  | Environmental enrichment extends ocular dominance plasticity into adulthood and protects from stroke-induced impairments of plasticity |
|  | Bioaccumulation and trophic transfer of pharmaceuticals in food webs from a large freshwater lake |
|  | The effects of centrally injected arachidonic acid on respiratory system: Involvement of cyclooxygenase to thromboxane signaling pathway |
|  | Inoculation of Lewis lung carcinoma cells enhances formalin-induced pain behavior and spinal Fos expression in mice |
|  | Spontaneous In Vitro and In Vivo Interaction of (-)-Oleocanthal with Glycine in Biological Fluids: Novel Pharmacokinetic Markers |
|  | Particulate matter and risk of Parkinson disease in a large prospective study of women |
|  | Comparison of cannabinoids with known analgesics using a novel high throughput zebrafish larval model of nociception |
|  | R-flurbiprofen improves tau, but not Aß pathology in a triple transgenic model of Alzheimer's disease |
|  | The Impact of Central and Peripheral Cyclooxygenase Enzyme Inhibition on Exercise-Induced Elevations in Core Body Temperature |
|  | Determinants of Indices of Cerebral Volume in Former Very Premature Infants at Term Equivalent Age |
|  | Agraphia: Presenting Feature of Syndrome of Transient Headache and Neurological Deficits With Cerebrospinal Fluid Lymphocytosis (HaNDL) |
|  | Occurrence, bioaccumulation and risk assessment of lipophilic pharmaceutically active compounds in the downstream rivers of sewage treatment plants |
|  | Elevated urinary NT-proBNP after pharmacological closure of patent ductus arteriosus in very low birth weight infants |
|  | Effectiveness of conservative interventions for sickness and pain behaviors induced by a high repetition high force upper extremity task |
|  | Centrally administered CDP-choline induced cardiovascular responses are mediated by activation of the central phospholipase-prostaglandin signaling cascade |
|  | Characterization of aldo-keto reductase 1C subfamily members encoded in two rat genes (akr1c19 and RGD1564865). Relationship to 9-hydroxyprostaglandin dehydrogenase |
|  | Deficiency in either COX-1 or COX-2 genes does not affect amyloid beta protein burden in amyloid precursor protein transgenic mice |
|  | Enhancement of Endocannabinoid-dependent Depolarization-induced Suppression of Excitation in Glycinergic Neurons by Prolonged Exposure to High Doses of Salicylate |
|  | Effects of triclocarban, N,N-diethyl-meta-toluamide, and a mixture of pharmaceuticals and personal care products on fathead minnows (Pimephales promelas) |
|  | Why is mesial temporal lobe epilepsy with Ammon's horn sclerosis becoming less common? |
|  | [Migraine triggered by laughing as a form of presentation of a Chiari type I malformation] |
|  | Vanishing bile duct syndrome in the context of concurrent temozolomide for glioblastoma |
|  | P2X7 Cell Death Receptor Activation and Mitochondrial Impairment in Oxaliplatin-Induced Apoptosis and Neuronal Injury: Cellular Mechanisms and In Vivo Approach |
|  | Postmortem tissue distribution of MDPV following lethal intoxication by "bath salts" |
|  | Managing fever in children: a national survey of parents' knowledge and practices in France |
|  | Glial activation and post-synaptic neurotoxicity: the key events in Streptozotocin (ICV) induced memory impairment in rats |
|  | Risk of ischemic stroke and the use of individual non-steroidal anti-inflammatory drugs: A multi-country European database study within the SOS Project |
|  | Analgesic properties of a peripherally acting and GalR2 receptor-preferring galanin analog in inflammatory, neuropathic, and acute pain models |
|  | Inflammation and depression: combined use of selective serotonin reuptake inhibitors and NSAIDs or paracetamol and psychiatric outcomes |
|  | Optimization of a cisplatin model of chemotherapy-induced peripheral neuropathy in mice: use of vitamin C and sodium bicarbonate pretreatments to reduce nephrotoxicity and improve animal health status |
|  | Inhibitors of oxidative and hydrolytic endocannabinoid degradation do not enhance depolarization-induced suppression of excitation on dorsal cochlear nucleus glycinergic neurons |
| Ketorolac | Ketorolac Administration Attenuates Retinal Ganglion Cell Death After Axonal Injury |
|  | Cyclooxygenase inhibition attenuates brain angiogenesis and independently decreases mouse survival under hypoxia |
|  | The Interaction of Intramuscular Ketorolac (Toradol) and Concussion in a Rat Model |
|  | Intraoperative ketorolac dose of 15mg versus the standard 30mg on early postoperative pain after spine surgery: A randomized, blinded, non-inferiority trial |
|  | Molecular Mechanisms of the Blockage of Glioblastoma Motility |
|  | Effects of omega 3 polyunsaturated fatty acids, antioxidants, and/or non-steroidal inflammatory drugs in the brain of neonatal rats exposed to intermittent hypoxia |
|  | Selective anesthesia-induced neuroinflammation in developing mouse brain and cognitive impairment |
|  | Canadian Headache Society systematic review and recommendations on the treatment of migraine pain in emergency settings |
|  | The Effects of Two Non-Steroidal Anti-Inflammatory Drugs, Bromfenac 0.1% and Ketorolac 0.45%, on Cataract Surgery |
|  | Immunotherapy associated pain crisis and the haemophagocytic lymphohistiocytosis syndrome in advanced melanoma: Case report and review of the literature |
|  | Antinociceptive tolerance to NSAIDs microinjected into dorsal hippocampus |
|  | Characterizing Hospitalizations for Pediatric Concussion and Trends in Care |
|  | Intravenous migraine therapy in children with posttraumatic headache in the ED |
|  | Spinal activity of interleukin 6 mediates myelin basic protein-induced allodynia |
|  | Antinociceptive tolerance to NSAIDs in the anterior cingulate cortex is mediated via endogenous opioid mechanism |
|  | Antinociceptive tolerance to NSAIDs in the agranular insular cortex is mediated by opioid mechanism |
|  | Serous retinal detachment and cystoid macular edema in a patient with Wyburn-Mason syndrome |
|  | Clinical Reasoning: a 44-year-old woman with headache followed by sudden neurologic decline |
|  | Correlation Between Nasal Epithelial Injury and In Vitro Cytotoxicity Using a Series of Small Molecule Protein Tyrosine Phosphatase 1B Inhibitors Investigated for Reversal of Leptin Resistance in Obesity |
|  | The Effects of Perioperative Anesthesia and Analgesia on Immune Function in Patients Undergoing Breast Cancer Resection: A Prospective Randomized Study |
|  | Activation of mesocorticolimbic reward circuits for assessment of relief of ongoing pain: a potential biomarker of efficacy |
|  | Risk of ischemic stroke and the use of individual non-steroidal anti-inflammatory drugs: A multi-country European database study within the SOS Project |
|  | Effects of Postoperative Pain Management on Immune Function After Laparoscopic Resection of Colorectal Cancer: A Randomized Study |
| Hydrocortisone | The neurochemistry of music |
|  | Diurnal cortisol slopes and mental and physical health outcomes: A systematic review and meta-analysis |
|  | Prophylactic hydrocortisone in extremely preterm infants and brain MRI abnormality |
|  | Early life stress and cortisol: A meta-analysis |
|  | Stress and Health: A Review of Psychobiological Processes |
|  | Cortisol on Circadian Rhythm and Its Effect on Cardiovascular System |
|  | Human studies on hypothalamo-pituitary-adrenal (HPA) axis |
|  | HPA axis in major depression: cortisol, clinical symptomatology and genetic variation predict cognition |
|  | Chronic stress, cortisol dysfunction, and pain: a psychoneuroendocrine rationale for stress management in pain rehabilitation |
|  | Probiotic Lactobacillus casei strain Shirota relieves stress-associated symptoms by modulating the gut-brain interaction in human and animal models |
|  | Duration of mechanical ventilation is more critical for brain growth than postnatal hydrocortisone in extremely preterm infants |
|  | Investigation of the Hypothalamo-pituitary-adrenal (HPA) axis: a contemporary synthesis |
|  | Salivary cortisol and α-amylase: subclinical indicators of stress as cardiometabolic risk |
|  | Hydrocortisone treatment for bronchopulmonary dysplasia and brain volumes in preterm infants |
|  | Structural brain abnormalities in Cushing's syndrome |
|  | Cortisol stress reactivity across psychiatric disorders: A systematic review and meta-analysis |
|  | Modulation of the hypothalamic-pituitary-adrenal (HPA) axis by plants and phytonutrients: a systematic review of human trials |
|  | Pilot randomized trial of hydrocortisone in ventilator-dependent extremely preterm infants: effects on regional brain volumes |
|  | Cortisol promotes breast-to-brain metastasis through the blood-cerebrospinal fluid barrier |
|  | A Refill for the Brain Mineralocorticoid Receptor: The Benefit of Cortisol Add-On to Dexamethasone Therapy |
|  | Safety of Withholding Perioperative Hydrocortisone for Patients With Pituitary Adenomas With an Intact Hypothalamus-Pituitary-Adrenal Axis: A Randomized Clinical Trial |
|  | Stress and Salivary Glands |
|  | Is physiological glucocorticoid replacement important in children? |
|  | Contemporary relevance of occlusion and mastication |
|  | Stress-dose hydrocortisone reduces critical illness-related corticosteroid insufficiency associated with severe traumatic brain injury in rats |
|  | At-risk individuals display altered brain activity following stress |
|  | Psychosocial and psychoneuroendocrinal aspects of Takotsubo syndrome |
|  | Circulating cortisol and cognitive and structural brain measures: The Framingham Heart Study |
|  | No association between hair cortisol or cortisone and brain morphology in children |
|  | Brain Mechanisms of Social Threat Effects on Working Memory |
|  | Hippocampal volume in healthy controls given 3-day stress doses of hydrocortisone |
|  | Effects of cocaine on the hypothalamic-pituitary-adrenal axis |
|  | Infant diurnal cortisol predicts sleep |
|  | Sexual dimorphism in the neural impact of stress and alcohol |
|  | The impact of chronic stress on the rat brain lipidome |
|  | Stress Impact on Resting State Brain Networks |
|  | Hypothalamic-pituitary-adrenal axis hyperactivity and brain differences in healthy women |
|  | Cortisol alters reward processing in the human brain |
|  | Hair cortisol concentrations in mental disorders: A systematic review |
|  | Is Posthospital Syndrome a Result of Hospitalization-Induced Allostatic Overload? |
|  | Postnatal steroids in the preterm infant-the good, the ugly, and the unknown |
|  | Music, Spatial Task Performance, and Brain Plasticity in Elderly Adults |
|  | Stress hormones in obstructive sleep apnea complications: the role of cortisol |
|  | Salivary Cortisol and Regional Brain Volumes Among Veterans With and Without Posttraumatic Stress Disorder |
|  | Stress-induced brain activity, brain atrophy, and clinical disability in multiple sclerosis |
|  | Integrative Review of Early Life Adversity and Cortisol Regulation in Pregnancy |
|  | Cortisol administration induces global down-regulation of the brain's reward circuitry |
|  | Interaction of circadian and stress systems in the regulation of adipose physiology |
|  | The diagnosis and treatment of adrenal insufficiency during childhood and adolescence |
|  | Brain serotonin 4 receptor binding is associated with the cortisol awakening response |
|  | Structural changes of the brain in relation to occupational stress |
|  | Uncertainties in endocrine substitution therapy for central hypocortisolism |
|  | Novel insights in the HPA-axis during critical illness |
|  | A systematic review of physiological reactivity to stimuli in autism |
|  | Severe hypoxia exposure inhibits larval brain development but does not affect the capacity to mount a cortisol stress response in zebrafish |
|  | Brain white matter integrity and cortisol in older men: the Lothian Birth Cohort 1936 |
|  | Discrimination and the HPA axis: current evidence and future directions |
|  | Study protocol: imaging brain development in the Childhood to Adolescence Transition Study (iCATS) |
|  | Brain cortisol receptor expression differs in Arctic charr displaying opposite coping styles |
|  | Cortisol effects on brain functional connectivity during emotion processing in women with depression |
|  | Understanding the HPA response to critical illness: novel insights with clinical implications |
|  | Co-culture model consisting of human brain microvascular endothelial and peripheral blood mononuclear cells |
|  | Longitudinal relationships among depressive symptoms, cortisol, and brain atrophy in the neocortex and the hippocampus |
|  | A systematic review of the activity of the hypothalamic-pituitary-adrenal axis in first episode psychosis |
|  | Relative frontal brain asymmetry and cortisol release after social stress: The role of action orientation |
|  | Aging and the HPA axis: Stress and resilience in older adults |
|  | Human nail cortisol as a retrospective biomarker of chronic stress: A systematic review |
|  | Empathy networks in the parental brain and their long-term effects on children's stress reactivity and behavior adaptation |
|  | Voxel-based comparison of brain glucose metabolism between patients with Cushing's disease and healthy subjects |
|  | Cortisol awakening response in patients with psychosis: Systematic review and meta-analysis |
|  | Brain glucose metabolism is associated with hormone level in Cushing's disease: A voxel-based study using FDG-PET |
|  | Altered ultradian cortisol rhythmicity as a potential neurobiologic substrate for chronic insomnia |
|  | Glucocorticoids as an emerging pharmacologic agent for cardiopulmonary resuscitation |
|  | Salivary Cortisol: Is it Still the Canary in the Coal Mine? |
|  | Computerized Analysis of Brain MRI Parameter Dynamics in Young Patients With Cushing Syndrome-A Case-Control Study |
|  | Momentary emotions and salivary cortisol: A systematic review and meta-analysis of ecological momentary assessment studies |
|  | Hair Cortisol Analysis: A Promising Biomarker of HPA Activation in Older Adults |
|  | The differential calibration of the HPA axis as a function of trauma versus adversity: A systematic review and p-curve meta-analyses |
|  | Association between resting-state brain functional connectivity and cortisol levels in unmedicated major depressive disorder |
|  | Prenatal Maternal Cortisol Has Sex-Specific Associations with Child Brain Network Properties |
|  | Stress Physiology in Infancy and Early Childhood: Cortisol Flexibility, Attunement and Coordination |
|  | Neurobehavioral effects of acute low-dose whole-body irradiation |
|  | Gravity in the brain-how it may regulate skeletal muscle metabolism by balancing compressive ischemic changes in the weight-bearing pituitary and hypothalamus |
|  | Measuring Stress in Young Children Using Hair Cortisol: The State of the Science |
|  | Neither cortisol nor brain-derived neurotrophic factor is associated with serotonin transporter in bipolar disorder |
|  | Are attenuated positive symptoms and cortisol levels associated? |
|  | Maternal prenatal cortisol programs the infant hypothalamic-pituitary-adrenal axis |
|  | Basal cortisol levels and metabolic syndrome: A systematic review and meta-analysis of observational studies |
|  | The burden of conscientiousness? Examining brain activation and cortisol response during social evaluative stress |
|  | Time related effects on functional brain connectivity after serotonergic and cholinergic neuromodulation |
|  | The effects of acute stress and stress hormones on social cognition and behavior: Current state of research and future directions |
|  | A neurophysiological signature of motivational incongruence: EEG changes related to insufficient goal satisfaction |
|  | Genes and hormones of the hypothalamic-pituitary-adrenal axis in post-traumatic stress disorder. What is their role in symptom expression and treatment response? |
|  | Developmental and contextual considerations for adrenal and gonadal hormone functioning during adolescence: Implications for adolescent mental health |
|  | 11β-Hydroxysteroid dehydrogenase activity in the brain does not contribute to systemic interconversion of cortisol and cortisone in healthy men |
|  | Psychosocial Influences on Acceptability and Feasibility of Salivary Cortisol Collection From Community Samples of Children |
|  | Cortisol as a predictor of psychological therapy response in anxiety disorders-Systematic review and meta-analysis |
|  | Linking the hemodynamic consequences of adverse childhood experiences to an altered HPA axis and acute stress response |
|  | Influence of early trauma on features of schizophrenia |
|  | Dominance status alters restraint-induced neural activity in brain regions controlling stress vulnerability |
|  | Hypothalamic-Pituitary-Adrenal Axis Function in Children and Adults with Severe Antisocial Behavior and the Impact of Early Adversity |
|  | Integrated Inflammatory Stress (ITIS) Model |
|  | Serum cortisol mediates the relationship between fecal Ruminococcus and brain N-acetylaspartate in the young pig |
|  | [Stress and psychotic transition: A literature review] |
|  | [Understanding cortisol action in acute inflammation: A view from the adrenal gland to the target cell] |
|  | Septo-Optic Dysplasia: A Case Study |
|  | Pharmacological modulation of acute trauma memories to prevent PTSD: considerations from a developmental perspective |
|  | A combination of green tea, rhodiola, magnesium and B vitamins modulates brain activity and protects against the effects of induced social stress in healthy volunteers |
|  | Use of Salivary Diurnal Cortisol as an Outcome Measure in Randomised Controlled Trials: a Systematic Review |
|  | Quantification of 11β-hydroxysteroid dehydrogenase 1 kinetics and pharmacodynamic effects of inhibitors in brain using mass spectrometry imaging and stable-isotope tracers in mice |
|  | Research review: evaluating and reformulating the developmental taxonomic theory of antisocial behaviour |
|  | Expanding the actions of cortisol and corticosterone in wild vertebrates: A necessary step to overcome the emerging challenges |
|  | What we remember from a stressful episode |
|  | Acute net stressor increases whole-body cortisol levels without altering whole-brain monoamines in zebrafish |
|  | A dual-axis approach to understanding neuroendocrine development |
|  | Psychosocial functioning and the cortisol awakening response: Meta-analysis, P-curve analysis, and evaluation of the evidential value in existing studies |
|  | Dietary l-tryptophan leaves a lasting impression on the brain and the stress response |
|  | Sex differences in cognitive regulation of psychosocial achievement stress: brain and behavior |
|  | Effect of Intensive Training on Mood With No Effect on Brain-Derived Neurotrophic Factor |
|  | Artificial selection on relative brain size reveals a positive genetic correlation between brain size and proactive personality in the guppy |
|  | Sexual well-being and diurnal cortisol after prostate cancer treatment |
|  | Diurnal cortisol after early institutional care-Age matters |
|  | Early life low intensity stress experience modifies acute stress effects on juvenile brain cell proliferation of European sea bass (D. Labrax) |
|  | Positive upshots of cortisol in everyday life |
|  | A randomized, placebo-controlled proof-of-concept, crossover trial of phenytoin for hydrocortisone-induced declarative memory changes |
|  | Progesterone level predicts serotonin-1a receptor binding in the male human brain |
|  | Prenatal CRH: An integrating signal of fetal distress |
|  | Management of glucocorticoids following adrenalectomy for ACTH-independent Cushing's syndrome |
|  | Norepinephrine transporter blocker atomoxetine increases salivary alpha amylase |
|  | Hair Cortisol and Self-Injurious Behavior Among Children With Autism Spectrum Disorder |
|  | The response of brain serotonergic and dopaminergic systems to an acute stressor in rainbow trout: a time course study |
|  | The chronic effect of cortisol on orchestrating cerebral blood flow and brain functional connectivity: evidence from Cushing's disease |
|  | It still hurts: altered endogenous opioid activity in the brain during social rejection and acceptance in major depressive disorder |
|  | Oxytocin Modulates Semantic Integration in Speech Comprehension |
|  | Neural correlates of glucocorticoids effects on autobiographical memory retrieval in healthy women |
|  | Effects on pig immunophysiology, PBMC proteome and brain neurotransmitters caused by group mixing stress and human-animal relationship |
|  | Cortisol rapidly affects amplitudes of heartbeat-evoked brain potentials--implications for the contribution of stress to an altered perception of physical sensations? |
|  | Chronic stress exposure may affect the brain's response to high calorie food cues and predispose to obesogenic eating habits |
|  | Interactions of noradrenaline and cortisol and the induction of indelible memories |
|  | Neural correlates of parent-child HPA axis coregulation |
|  | Neural Underpinnings of Cortisol Effects on Fear Extinction |
|  | Mineralocorticoid receptor blockade prevents stress-induced modulation of multiple memory systems in the human brain |
|  | Clinical Reasoning: A 41-year-old man with thunderclap headache |
|  | Hypothalamic-pituitary-adrenal axis, childhood adversity and adolescent nonsuicidal self-injury |
|  | Childhood trauma affects autobiographical memory deficits through basal cortisol and prefrontal-extrastriate functional connectivity |
|  | Vasovagal reactions following venepuncture result in aberrant stress-induced cortisol levels |
|  | Day differences in the cortisol awakening response predict day differences in synaptic plasticity in the brain |
|  | Milk cortisol response to group relocation in lactating cows |
|  | The Price of Stress: High Bedtime Salivary Cortisol Levels Are Associated with Brain Atrophy and Cognitive Decline in Stroke Survivors. Results from the TABASCO Prospective Cohort Study |
|  | Role of testosterone: cortisol ratio in age- and sex-specific cortico-hippocampal development and cognitive performance |
|  | Childhood Adversity, Cortisol Levels, and Psychosis: A Retrospective Investigation |
|  | Socioeconomic Disparities Affect Children's Amygdala-Prefrontal Circuitry via Stress Hormone Response |
|  | Changes in neuroactive steroid concentrations after preterm delivery in the Guinea pig |
|  | Radiolabel validation of cortisol in the hair of rhesus monkeys |
|  | The relation between cortisol and functional connectivity in people with and without stress-sensitive epilepsy |
|  | Oxytocin facilitates the sensation of social stress |
|  | Virtual and real-life ostracism and its impact on a subsequent acute stressor |
|  | The relationship between personality and the response to acute psychological stress |
|  | Glucocorticoid ultradian rhythmicity differentially regulates mood and resting state networks in the human brain: A randomised controlled clinical trial |
|  | Prenatal maternal cortisol concentrations predict neurodevelopment in middle childhood |
|  | Blood lead levels and hypothalamic-pituitary-adrenal function in middle-aged individuals |
|  | Maternal Cortisol Mediates Hypothalamus-Pituitary-Interrenal Axis Development in Zebrafish |
|  | Collecting Hair Samples for Hair Cortisol Analysis in African Americans |
|  | Parenting predicts Strange Situation cortisol reactivity among children adopted internationally |
|  | Sleep and cortisol interact to support memory consolidation |
|  | Maternal separation in childhood and hair cortisol concentrations in late adulthood |
|  | Physiological responses to acute psychosocial stress in women with menopausal insomnia |
|  | Fecal glucocorticoid metabolites reflect hypothalamic-pituitary-adrenal axis activity in muskoxen (Ovibos moschatus) |
|  | Friendship network position and salivary cortisol levels |
|  | Intimate partner violence and diurnal cortisol patterns in couples |
|  | Dissociated neural effects of cortisol depending on threat escapability |
|  | Role of DHEA and cortisol in prefrontal-amygdalar development and working memory |
|  | The brain-gut axis of fish: Rainbow trout with low and high cortisol response show innate differences in intestinal integrity and brain gene expression |
|  | Stress, stress-induced cortisol responses, and eyewitness identification performance |
|  | Distinguishing shyness and sociability in adults: An event-related electrocortical-neuroendocrine study |
|  | Routine Magnetic Resonance Imaging at Term-Equivalent Age Detects Brain Injury in 25% of a Contemporary Cohort of Very Preterm Infants |
|  | Acute Psychological Stress Disrupts Attentional Bias to Threat-Related Stimuli |
|  | Longitudinal changes in amygdala, hippocampus and cortisol development following early caregiving adversity |
|  | Yawning, fatigue, and cortisol: expanding the Thompson Cortisol Hypothesis |
|  | High oxygen consumption rates and scale loss indicate elevated aggressive behaviour at low rearing density, while elevated brain serotonergic activity suggests chronic stress at high rearing densities in farmed rainbow trout |
|  | A Pharmacokinetic Model of a Tissue Implantable Cortisol Sensor |
|  | Low hair cortisol concentration and emerging attention-deficit/hyperactivity symptoms in preschool age |
|  | Functionally distinct smiles elicit different physiological responses in an evaluative context |
|  | Amygdala volume and hypothalamic-pituitary-adrenal axis reactivity to social stress |
|  | Concordant pattern of the HPA axis response to visceral stimulation and CRH administration |
|  | Childhood Adversity, Self-Esteem, and Diurnal Cortisol Profiles Across the Life Span |
|  | Limbic response to stress linking life trauma and hypothalamus-pituitary-adrenal axis function |
|  | Acute stress contributes to individual differences in pain and pain-related brain activity in healthy and chronic pain patients |
|  | Changes in the social environment induce neurogenic plasticity predominantly in niches residing in sensory structures of the zebrafish brain independently of cortisol levels |
|  | Inter-relation between autonomic and HPA axis activity in children and adolescents |
|  | Cortisol disrupts the neural correlates of extinction recall |
|  | Early adversity and internalizing symptoms in adolescence: Mediation by individual differences in latent trait cortisol |
|  | Incorporating cortisol into the NAPLS2 individualized risk calculator for prediction of psychosis |
|  | National German Audit of Diagnosis, Treatment, and Teaching in Secondary Adrenal Insufficiency |
|  | Hyper- and hypo-cortisol functioning in post-institutionalized adolescents: The role of severity of neglect and context |
|  | Glucocorticoid Administration Improves Aberrant Fear-Processing Networks in Spider Phobia |
|  | Alpha-1 Acid Glycoprotein (orosomucoid): An Overlooked Piece of Post Traumatic Stress Disorder Pathology? |
|  | Defensive freezing links Hypothalamic-Pituitary-Adrenal-axis activity and internalizing symptoms in humans |
|  | Depression and anxiety predict sex-specific cortisol responses to interpersonal stress |
|  | Glucocorticoid administration restores salience network activity in patients with spider phobia |
|  | Identifying diurnal cortisol profiles among young adults: Physiological signatures of mental health trajectories |
|  | Sex differences in the association between obsessive-compulsive symptom dimensions and diurnal cortisol patterns |
|  | Age moderates the association between social integration and diurnal cortisol measures |
|  | Le dosage salivaire du cortisol doit être reconnu comme utile en France |
|  | Neural Signaling of Cortisol, Childhood Emotional Abuse, and Depression-Related Memory Bias |
|  | Acute and delayed hormonal and blood cell count responses to high-intensity exercise before and after short-term high-intensity interval training |
|  | Does intensity or youth affect the neurobiological effect of exercise on major depressive disorder? |
|  | Elevated cortisol in healthy female adolescent offspring of mothers with posttraumatic stress disorder |
|  | Determinants of cortisol during pregnancy - The ABCD cohort |
|  | Associations between circadian and stress response cortisol in children |
|  | The role of hypocortisolism in chronic fatigue syndrome |
|  | Hair cortisol levels and mental health problems in children and adolescents exposed to victimization |
|  | Systematic review of the effects of acute stress in binge eating disorder |
|  | Neuroendocrine stress responses predict catecholamine-dependent working memory-related dorsolateral prefrontal cortex activity |
|  | Can the neural-cortisol association be moderated by experience-induced changes in awareness? |
|  | Sustained striatal activity predicts eudaimonic well-being and cortisol output |
|  | Developmental effects in physiological stress in early adolescents with and without autism spectrum disorder |
|  | Neural Correlates of the Cortisol Awakening Response in Humans |
|  | An integrated approach to understand biological stress system dysregulation across depressive and anxiety disorders |
|  | Correlations of hair level with salivary level in cortisol and cortisone |
|  | Insecure Attachment and Hypothalamus-Pituitary-Adrenal Axis Functioning in People With Eating Disorders |
|  | Stress and cortisol responses in men: differences according to facial symmetry |
|  | Loneliness in older adults is associated with diminished cortisol output |
|  | Children's diurnal cortisol responses to negative events at school and home |
|  | Externalizing behavior and stress system functioning in infants exposed to early adversity: A multi-system exploration |
|  | Longer-term increased cortisol levels in young people with mental health problems |
|  | The role of co-rumination and adrenocortical attunement in young women's close friendships |
|  | Regional microstructural organization of the cerebral cortex is affected by preterm birth |
|  | Daily family stress and HPA axis functioning during adolescence: The moderating role of sleep |
|  | Sex differences in the functional connectivity of the amygdalae in association with cortisol |
|  | Amygdala-centred functional connectivity affects daily cortisol concentrations: a putative link with anxiety |
|  | Stress in the zoo: Tracking the impact of stress on memory formation over time |
|  | Sex differences, hormones, and fMRI stress response circuitry deficits in psychoses |
|  | Is the hypothalamic-pituitary-adrenal axis disrupted in type 2 diabetes mellitus and is this relevant for bone health? |
|  | Early developmental emergence of human amygdala-prefrontal connectivity after maternal deprivation |
|  | Prenatal and childhood stress exposure and the sex specific response to psychosocial stress in adulthood |
|  | Externalizing and Internalizing Problems: Associations with Family Adversity and Young Children's Adrenocortical and Autonomic Functioning |
|  | Long-term cognitive effects of glucocorticoid excess in Cushing's syndrome |
|  | Late-Life Depression, Cortisol, and the Hippocampus: On the Need to Consider Depressive, Hippocampal, and Pharmacological Complexities |
|  | Hair cortisol and the relationship with chronic pain and quality of life in endometriosis patients |
|  | Cortisol, but not intranasal insulin, affects the central processing of visual food cues |
|  | Hypothalamic-pituitary-adrenal axis function and exposure to stress factors and cannabis use in recent-onset psychosis |
|  | Early-life manipulation of cortisol and its receptor alters stress axis programming and social competence |
|  | Endogenous testosterone and cortisol modulate neural responses during induced anger control |
|  | Effects of childhood trauma on cortisol levels in suicide attempters and ideators |
|  | Cortisol increases the return of fear by strengthening amygdala signaling in men |
|  | Blunted cortisol stress reactivity in low-income children relates to lower memory function |
|  | Acute and past subjective stress influence working memory and related neural substrates |
|  | Hair glucocorticoids and resting-state frontal lobe oxygenation: Findings from The Irish Longitudinal Study on Ageing |
|  | Posttraumatic stress disorder (PTSD) and depression severity in sexually assaulted women: hypothalamic-pituitary-adrenal (HPA) axis alterations |
|  | Differential associations between childhood trauma subtypes and adolescent HPA-axis functioning |
|  | Childhood poverty and stress reactivity are associated with aberrant functional connectivity in default mode network |
|  | Conscientiousness, hair cortisol concentration, and health behaviour in older men and women |
|  | Associations between psychiatric symptoms and cortisol levels in Nicaraguan young school-age children |
|  | Glucocorticoids regulate metallothionein-1/2 expression in rat choroid plexus: effects on apoptosis |
|  | Stress evokes stronger medial posterior cingulate deactivations during emotional distraction in slower paced aging |
|  | Sleep and Physiological Dysregulation: A Closer Look at Sleep Intraindividual Variability |
|  | Perceived stress and hair cortisol: Differences in bipolar disorder and schizophrenia |
|  | Early life stress sensitizes youth to the influence of stress-induced cortisol on memory for affective words |
|  | Cortisol reactivity and weight gain among adolescents who vary in prenatal drug exposure |
|  | Hair and salivary cortisol in a cohort of women with chronic fatigue syndrome |
|  | Working memory-related frontal theta activity is decreased under acute stress |
|  | Differences in Cortisol Awakening Response between Binge-Purging and Restrictive Patients with Anorexia Nervosa |
|  | Maternal sensitivity and adrenocortical functioning across infancy and toddlerhood: Physiological adaptation to context? |
|  | Individual differences in early adolescents' latent trait cortisol (LTC): Relation to early adversity |
|  | Family conflict and lower morning cortisol in adolescents and adults: modulation of puberty |
|  | Longitudinal pattern of early maturation on morning cortisol and depressive symptoms: Sex-specific effects |
|  | Does Psychosocial Stress Impact Cognitive Reappraisal? Behavioral and Neural Evidence |
|  | A Modified Trier Social Stress Test for Vulnerable Mexican American Adolescents |
|  | Stress eliminates retrieval-induced forgetting--does the oral application of cortisol? |
|  | Gaze behavior is associated with the cortisol response to acute psychosocial stress in the virtual TSST |
|  | Hair cortisol and cognitive performance in healthy older people |
|  | Reply to: Late-Life Depression, Cortisol, and the Hippocampus: On the Need to Consider Depressive, Hippocampal, and Pharmacological Complexities |
|  | Exploring longitudinal associations between neighborhood disadvantage and cortisol levels in early childhood |
|  | Associations between hippocampal morphology, diffusion characteristics, and salivary cortisol in older men |
|  | Impact of one HF-rTMS session over the DLPFC and motor cortex on acute hormone dynamics and emotional state in healthy adults: a sham-controlled pilot study |
|  | Reduced Slow-Wave Sleep and Altered Diurnal Cortisol Rhythms in Patients with Addison's Disease |
|  | Heart rate variability and salivary cortisol in very preterm children during school age |
|  | Social group dynamics predict stress variability among children in a New Zealand classroom |
|  | Amygdala Reward Reactivity Mediates the Association Between Preschool Stress Response and Depression Severity |
|  | HPA stability for children in foster care: mental health implications and moderation by early intervention |
|  | A polymorphism in the glucocorticoid receptor gene is associated with refractory hypotension in premature infants |
|  | Checking behavior in rhesus monkeys is related to anxiety and frontal activity |
|  | Coordination between frontolimbic resting state connectivity and hypothalamic-pituitary-adrenal axis functioning in adolescents with and without depression |
|  | Diurnal cortisol rhythm is associated with increased risky decision-making in older adults |
|  | HPA axis regulation and epigenetic programming of immune-related genes in chronically stressed and non-stressed mid-life women |
|  | Cortisol modifies extinction learning of recently acquired fear in men |
|  | Specific reduction in cortisol stress reactivity after social but not attention-based mental training |
|  | State-Independent and Dependent Neural Responses to Psychosocial Stress in Current and Remitted Depression |
|  | Effects of prenatal stress on fetal neurodevelopment and responses to maternal neurosteroid treatment in Guinea pigs |
|  | Childhood maltreatment, pituitary volume and adolescent hypothalamic-pituitary-adrenal axis - Evidence for a maltreatment-related attenuation |
|  | Does experimental cortisol elevation mediate risk-taking and antipredator behaviour in a wild teleost fish? |
|  | The Hepato-Hypothalamic-Pituitary-Adrenal-Renal Axis: Mathematical Modeling of Cortisol's Production, Metabolism, and Seasonal Variation |
|  | An integrative analysis of ethanol tolerance and withdrawal in zebrafish (Danio rerio) |
|  | PCLO rs2522833-mediated gray matter volume reduction in patients with drug-naive, first-episode major depressive disorder |
|  | Ultradian rhythmicity of plasma cortisol is necessary for normal emotional and cognitive responses in man |
|  | Neural and cortisol responses during play with human and computer partners in children with autism |
|  | Assessing the daily stability of the cortisol awakening response in a controlled environment |
|  | No acute suppression of cerebrospinal fluid corticotropin-releasing hormone in man by cortisol administration |
|  | Academic stress and personality interact to increase the neural response to high-calorie food cues |
|  | A cognitive stressor for event-related potential studies: the Portland arithmetic stress task |
|  | Early childhood adversity and HPA axis activity in adulthood:The importance of considering minimal age at exposure |
|  | A preliminary study of gut microbiome variation and HPA axis reactivity in healthy infants |
|  | A transgenic zebrafish model for monitoring glucocorticoid receptor activity |
|  | Temporal dynamics of stress-induced alternations of intrinsic amygdala connectivity and neuroendocrine levels |
|  | Pre-treatment cortisol awakening response predicts symptom reduction in posttraumatic stress disorder after treatment |
|  | The effect of a maternal history of childhood abuse on adrenocortical attunement in mothers and their toddlers |
|  | Ameliorating the biological impacts of childhood adversity: A review of intervention programs |
|  | HPA system activity in alexithymia: a cortisol awakening response study |
|  | HPA axis reactivity in early childhood: associations with symptoms and moderation by sex |
|  | Altered stress system reactivity after pediatric injury: Relation with post-traumatic stress symptoms |
|  | Evening salivary cortisol and alpha-amylase at 14months and neurodevelopment at 4years: Sex differences |
|  | Modulation of HPA axis response to social stress in schizophrenia by childhood trauma |
|  | Sexual orientation and diurnal cortisol patterns in a cohort of U.S. young adults |
|  | Neural circuitry of emotion regulation: Effects of appraisal, attention, and cortisol administration |
|  | The social buffering of the hypothalamic-pituitary-adrenocortical axis in humans: Developmental and experiential determinants |
|  | Early life stress modulates oxytocin effects on limbic system during acute psychosocial stress |
|  | Morning cortisol levels in schizophrenia and bipolar disorder: a meta-analysis |
|  | Commentary: Is there a there there in hair? A reflection on child maltreatment and hair cortisol concentrations in White et al. (2017) |
|  | Differential relations between youth internalizing/externalizing problems and cortisol responses to performance vs. interpersonal stress |
|  | Long-term stability of the cortisol awakening response over adolescence |
|  | Cortisol dysregulation is associated with daily diary-reported hot flashes among midlife women |
|  | Psychological, endocrine and neural responses to social evaluation in subclinical depression |
|  | Salivary cortisol levels as a biological marker of stress reaction |
|  | Disruption of the Diurnal Cortisol Hormone Pattern by Pesticide Use in a Longitudinal Study of Farmers in Thailand |
|  | Associations of work stress with hair cortisol concentrations - initial findings from a prospective study |
|  | Prenatal maternal mood is associated with altered diurnal cortisol in adolescence |
|  | Investigation into hippocampal nerve cell damage through the mineralocorticoid receptor in mice |
|  | Parental history of depression and higher basal salivary cortisol in unaffected child and adolescent offspring |
|  | Global variation in diurnal cortisol rhythms: evidence from Garisakang forager-horticulturalists of lowland Papua New Guinea |
|  | Increased anxiety-like behaviors, but blunted cortisol stress response after neonatal hippocampal lesions in monkeys |
|  | Stress differentially affects fear conditioning in men and women |
|  | Examining the concurrent and longitudinal relationship between diurnal cortisol rhythms and conduct problems during childhood |
|  | Short communication: hair cortisol concentrations in Holstein-Friesian and crossbreed F1 heifers |
|  | Prednisolone increases neural reactivity to negative socio-emotional stimuli in healthy young men |
|  | Perceived Stigmatization, Resilience, and Diurnal Cortisol Rhythm Among Children of Parents Living With HIV |
|  | How childhood trauma and recent adverse events are related to hair cortisol levels in a large adult cohort |
|  | A multi-dimensional characterization of anxiety in monozygotic twin pairs reveals susceptibility loci in humans |
|  | Cortisol Patterns for Young Children Displaying Disruptive Behavior: Links to a Teacher-Child, Relationship-Focused Intervention |
|  | Establishment of revised diagnostic cut-offs for adrenal laboratory investigation using the new Roche Diagnostics Elecsys(®) Cortisol II assay |
|  | Gut microbiota diversity but not composition is related to saliva cortisol stress response at the age of 2.5 months |
|  | Toxoplasma gondii infection in the peritoneal macrophages of rats treated with glucocorticoids |
|  | Intention retrieval and deactivation following an acute psychosocial stressor |
|  | What are the links between maternal social status, hippocampal function, and HPA axis function in children? |
|  | HPA axis linkage in parent-child dyads: Effects of parent sex, autism spectrum diagnosis, and dyadic relationship behavior |
|  | Caffeine neuroprotects against dexamethasone-induced anxiety-like behaviour in the Zebrafish (Danio rerio) |
|  | Nociceptive Cortical Activity Is Dissociated from Nociceptive Behavior in Newborn Human Infants under Stress |
|  | The Trend in Morning Levels of Salivary Cortisol in Children With ADHD During 6 Months of Methylphenidate Treatment |
|  | Physiological mechanisms that underlie the effects of interactional unfairness on deviant behavior: the role of cortisol activity |
|  | Advancing gestation does not attenuate biobehavioural coherence between psychological distress and cortisol |
|  | Longitudinal associations in adolescence between cortisol and persistent aggressive or rule-breaking behavior |
|  | Neural and neuroendocrine predictors of pharmacological treatment response in adolescents with depression: A preliminary study |
|  | Increased anxiety-like phenotype in female guinea pigs following reduced neurosteroid exposure in utero |
|  | Effortful control and parenting: associations with HPA axis reactivity in early childhood |
|  | The predictive value of cortisol levels on 2-year course of depression in older persons |
|  | Maternal Cortisol Concentrations During Pregnancy and Sex-Specific Associations With Neonatal Amygdala Connectivity and Emerging Internalizing Behaviors |
|  | Paediatric cyclical Cushing's disease due to corticotroph cell hyperplasia |
|  | Relationship of Hair Cortisol with History of Psychosis, Neuropsychological Performance and Functioning in Remitted Later-Life Major Depression |
|  | Cortisol cut-points for the glucagon stimulation test in the evaluation of hypothalamic pituitary adrenal axis |
|  | Flattened cortisol awakening response in chronic patients with schizophrenia onset after cannabis exposure |
|  | Stress as a mnemonic filter: Interactions between medial temporal lobe encoding processes and post-encoding stress |
|  | The slope of cortisol from awakening to 30 min post-wake in post-institutionalized children and early adolescents |
|  | Interactive effects of testosterone and cortisol on hippocampal volume and episodic memory in middle-aged men |
|  | Neural mechanisms underlying heterogeneity in the presentation of anxious temperament |
|  | Correlations between changes in the hypothalamic-pituitary-adrenal axis and neurochemistry of the anterior cingulate gyrus in postpartum depression |
|  | Transcranial electrical stimulation modifies the neuronal response to psychosocial stress exposure |
|  | Breast-Milk Cortisol and Cortisone Concentrations Follow the Diurnal Rhythm of Maternal Hypothalamus-Pituitary-Adrenal Axis Activity |
|  | Early psychosocial adversity and cortisol levels in children with attention-deficit/hyperactivity disorder |
|  | Analyses of hair and salivary cortisol for evaluating hypothalamic-pituitary-adrenal axis activation in patients with autoimmune disease |
|  | Maternal cortisol slope at 6 months predicts infant cortisol slope and EEG power at 12 months |
|  | Sleep quality but not sleep quantity effects on cortisol responses to acute psychosocial stress |
|  | Prefrontal serotonin transporter availability is positively associated with the cortisol awakening response |
|  | Pubertal transition with current life stress and support alters longitudinal diurnal cortisol patterns in adolescents exposed to early life adversity |
|  | Cognitive functioning and emotion processing in breast cancer survivors and controls: An ERP pilot study |
|  | Maternal negative affect during infancy is linked to disrupted patterns of diurnal cortisol and alpha asymmetry across contexts during childhood |
|  | High Cardiorespiratory Fitness Is Negatively Associated with Daily Cortisol Output in Healthy Aging Men |
|  | Neural - hormonal responses to negative affective stimuli: Impact of dysphoric mood and sex |
|  | Acute stress-induced cortisol elevations mediate reward system activity during subconscious processing of sexual stimuli |
|  | Does white matter structure or hippocampal volume mediate associations between cortisol and cognitive ageing? |
|  | Sex differences in salivary cortisol reactivity to the Trier Social Stress Test (TSST): A meta-analysis |
|  | Resting state functional MRI connectivity predicts hypothalamus-pituitary-axis status in healthy males |
|  | The Effects of Acute Physical Exercise on Memory, Peripheral BDNF, and Cortisol in Young Adults |
|  | Cortisol administration increases hippocampal activation to infant crying in males depending on childhood neglect |
|  | Alterations of autonomic nervous system and HPA axis basal activity and reactivity to acute stress: a comparison of traumatized adolescents and healthy controls |
|  | Cumulative exposure to socioeconomic and psychosocial adversity and hair cortisol concentration: A longitudinal study from 5 months to 17 years of age |
|  | The cortisol awakening response is blunted in patients with active Cushing's disease |
|  | Chronic stress exposure decreases the cortisol awakening response in healthy young men |
|  | Hair cortisol as a marker of hypothalamic-pituitary-adrenal Axis activity in female patients with major depressive disorder |
|  | Melatonin and cortisol secretion profile in patients with pineal cyst before and after pineal cyst resection |
|  | In vitro study of the long-term cortisol treatment effects on the growth rate and proliferation of the neural stem/precursor cells |
|  | Hypothalamic-pituitary-adrenal and cardiac autonomic responses to transrectal examination differ with behavioral reactivity in dairy cows |
|  | Longitudinal associations between low morning cortisol in infancy and anger dysregulation in early childhood in a CPS-referred sample |
|  | Evidence for increased immune mobilization in First Episode Psychosis compared with the prodromal stage in males |
|  | Long-term effects of preterm birth on behavior and neurosteroid sensitivity in the guinea pig |
|  | Characterization of the serum and salivary cortisol response to the intravenous 250 µg ACTH(1-24) stimulation test |
|  | Cortisol stress resonance in the laboratory is associated with inter-couple diurnal cortisol covariation in daily life |
|  | Modeling the Influence of Seasonal Differences in the HPA Axis on Synchronization of the Circadian Clock and Cell Cycle |
|  | The hypothalamo-pituitary-adrenal (HPA) axis in sheep is attenuated during lactation in response to psychosocial and predator stress |
|  | Hypothalamic-Pituitary-Adrenal Reactivity to Acute Stress: an Investigation into the Roles of Perceived Stress and Family Resources |
|  | How stable are diurnal cortisol activity indices in healthy individuals? Evidence from three multi-wave studies |
|  | Correlation of salivary cortisol level with obstructive sleep apnea syndrome in pediatric subjects |
|  | The relationship between early post-operative ACTH / cortisol following pituitary surgery and long-term glucocorticoid requirement - Do ultradian rhythms matter? |
|  | Multilevel assessment of the neurobiological threat system in depressed adolescents: interplay between the limbic system and hypothalamic-pituitary-adrenal axis |
|  | Effects of the pattern of glucocorticoid replacement on neural processing, emotional reactivity and well-being in healthy male individuals: study protocol for a randomised controlled trial |
|  | Comparison of aggressive behaviors between two wild populations of Japanese medaka, Oryzias latipes and O. sakaizumii |
|  | The effects of exercise training on hypothalamic-pituitary-adrenal axis reactivity and autonomic response to acute stress-a randomized controlled study |
|  | Effects of a pesticide and a parasite on neurological, endocrine, and behavioral responses of an estuarine fish |
|  | Intranasal insulin increases regional cerebral blood flow in the insular cortex in men independently of cortisol manipulation |
|  | The role of cortisol in chronic binge alcohol-induced cerebellar injury: Ovine model |
|  | Long-term cortisol stress response in depression and comorbid anxiety is linked with reduced N-acetylaspartate in the anterior cingulate cortex |
|  | Effects of late-night eating of easily-or slowly-digestible meals on sleep, hypothalamo-pituitary-adrenal axis, and autonomic nervous system in healthy young males |
|  | Memory performance is related to the cortisol awakening response in older people, but not to the diurnal cortisol slope |
|  | The cortisol awakening response and anterior cingulate cortex function in maltreated depressed versus non-maltreated depressed youth |
|  | Assessing significant (>30%) alopecia as a possible biomarker for stress in captive rhesus monkeys (Macaca mulatta) |
|  | Salivary latent trait cortisol (LTC): Relation to lipids, blood pressure, and body composition in middle childhood |
|  | Individual differences in early adolescents' latent trait cortisol (LTC): Relation to recent acute and chronic stress |
|  | How to disentangle psychobiological stress reactivity and recovery: A comparison of model-based and non-compartmental analyses of cortisol concentrations |
|  | Reduced hippocampal volume and hypothalamus-pituitary-adrenal axis function in first episode psychosis: evidence for sex differences |
|  | The relationship between cortisol and cognitive function in healthy older people: The moderating role of Apolipoprotein E polymorphism |
|  | A randomized controlled trial on the influence of two types of exercise training vs control on visuospatial processing and mathematical skills: The role of cortisol |
|  | Longitudinal changes in spouses' HPA responses: Convergence in cortisol patterns during the early years of marriage |
|  | An experimental evaluation of the role of the stress axis in mediating predator-prey interactions in wild marine fish |
|  | Higher diurnal salivary cortisol levels are related to smaller prefrontal cortex surface area in elderly men and women |
|  | Putting a finger on the problem: Finger stick blood draw and immunization at the well-child exam elicit a cortisol response to stress among one-year-old children |
|  | Towards understanding sex differences in visceral pain: enhanced reactivation of classically-conditioned fear in healthy women |
|  | Cortisol awakening response is decreased in patients with first-episode psychosis and increased in healthy controls with a history of severe childhood abuse |
|  | Hypercortisolemia and glucocorticoid receptor-signaling insufficiency in Alzheimer's disease initiation and development |
|  | Associations Between Hormonal Biomarkers and Cognitive, Motor, and Language Developmental Status in Very Low Birth Weight Infants |
|  | Afternoon cortisol provides a link between self-regulated anger and peer-reported aggression in typically developing children in the school context |
|  | Individual differences in the activity of the hypothalamic pituitary adrenal axis: Relations to age and cumulative risk in early childhood |
|  | Effects of nutritional history on stress response in gibel carp (Carassius auratus gibelio) and largemouth bass (Micropterus salmoides) |
|  | Ontogenesis of the HPI axis and molecular regulation of the cortisol stress response during early development in Dicentrarchus labrax |
|  | Accelerated DNA methylation age in adolescent girls: associations with elevated diurnal cortisol and reduced hippocampal volume |
|  | Elevated cortisol awakening response associated with early life stress and impaired executive function in healthy adult males |
|  | Examining the relationships between egg cortisol and oxidative stress in developing wild sockeye salmon (Oncorhynchus nerka) |
|  | Interaction between perceived maternal care, anxiety symptoms, and the neurobehavioral response to palatable foods in adolescents |
|  | In Obesity, HPA Axis Activity Does Not Increase with BMI, but Declines with Aging: A Meta-Analysis of Clinical Studies |
|  | Early life stress induces long-term changes in limbic areas of a teleost fish: the role of catecholamine systems in stress coping |
|  | Administration of Progesterone Throughout Pregnancy Increases Maternal Steroids Without Adverse Effect on Mature Oligodendrocyte Immunostaining in the Guinea Pig |
|  | The effects of left dorsolateral prefrontal transcranial direct current stimulation on episodic future thinking following acute psychosocial stress |
|  | Impaired cortisol awakening response in eating disorder women with childhood trauma exposure: evidence for a dose-dependent effect of the traumatic load |
|  | Relationship between white matter integrity and serum cortisol levels in drug-naive patients with major depressive disorder: diffusion tensor imaging study using tract-based spatial statistics |
|  | Mind-body interactions in the regulation of airway inflammation in asthma: A PET study of acute and chronic stress |
|  | The effect of exogenous cortisol during sleep on the behavioral and neural correlates of emotional memory consolidation in humans |
|  | Marital status as a predictor of diurnal salivary cortisol levels and slopes in a community sample of healthy adults |
|  | The psychology of HPA axis activation: Examining subjective emotional distress and control in a phobic fear exposure model |
|  | Neuroendocrine profiles associated with discrete behavioural variation in Symphodus ocellatus, a species with male alternative reproductive tactics |
|  | Early social deprivation and the social buffering of cortisol stress responses in late childhood: An experimental study |
|  | Circadian and homeostatic modulation of functional connectivity and regional cerebral blood flow in humans under normal entrained conditions |
|  | The mineralocorticoid receptor agonist, fludrocortisone, differentially inhibits pituitary-adrenal activity in humans with psychotic major depression |
|  | Interindividual differences in stress sensitivity: basal and stress-induced cortisol levels differentially predict neural vigilance processing under stress |
|  | Chronicity of depressive problems and the cortisol response to psychosocial stress in adolescents: the TRAILS study |
|  | Separating generalized anxiety disorder from major depression using clinical, hormonal, and structural MRI data: A multimodal machine learning study |
|  | Callosal abnormalities, altered cortisol levels, and neurocognitive deficits associated with early maltreatment among adolescents: A voxel-based diffusion-tensor imaging study |
|  | Blunted stress cortisol reactivity and failure to acclimate to familiar stress in depressed and sub-syndromal children |
|  | Sex differences in the ACTH and cortisol response to pharmacological probes are stressor-specific and occur regardless of alcohol dependence history |
|  | Anti-stress and nootropic activity of drugs affecting the renin-angiotensin system in rats based on indirect biochemical evidence |
|  | Cortisol-mediated downregulation of the serotonin 1A receptor subtype in the Gulf toadfish, Opsanus beta |
|  | [Effects of childhood trauma on the biochemical correlates of stress in men and women with nonpsychotic mental disorders] |
|  | Experience-Driven Differences in Childhood Cortisol Predict Affect-Relevant Brain Function and Coping in Adolescent Monozygotic Twins |
|  | Responses to the Human Intruder Test are related to hair cortisol phenotype and sex in rhesus macaques (Macaca mulatta) |
|  | Hyperthyroidism due to thyroid-stimulating hormone secretion after surgery for Cushing's syndrome: a novel cause of the syndrome of inappropriate secretion of thyroid-stimulating hormone |
|  | Stress and decision making: neural correlates of the interaction between stress, executive functions, and decision making under risk |
|  | Cortisol awakening response in infants during the first six postnatal months and its relation to birth outcome |
|  | A pilot study of depressed adolescents' cortisol patterns during parent-adolescent conflict and response to interpersonal psychotherapy (IPT-A) |
|  | Higher levels of physical activity are associated with lower hypothalamic-pituitary-adrenocortical axis reactivity to psychosocial stress in children |
|  | Reduced cingulate gyrus volume associated with enhanced cortisol awakening response in young healthy adults reporting childhood trauma |
|  | Interplay of hippocampal volume and hypothalamus-pituitary-adrenal axis function as markers of stress vulnerability in men at ultra-high risk for psychosis |
|  | Role of the NMDA receptor in cognitive deficits, anxiety and depressive-like behavior in juvenile and adult mice after neonatal dexamethasone exposure |
|  | Do psychosocial predictors affect the following days' cortisol awakening response? Expanding the temporal frame with which to explore morning cortisol |
|  | Early childhood cortisol reactivity moderates the effects of parent-child relationship quality on the development of children's temperament in early childhood |
|  | Behavioral reactivity to emotion challenge is associated with cortisol reactivity and regulation at 7, 15, and 24 months of age |
|  | Diurnal Cortisol Patterns and Dexamethasone Suppression Test Responses in Healthy Young Adults Born Preterm at Very Low Birth Weight |
|  | Polarity- and valence-dependent effects of prefrontal transcranial direct current stimulation on heart rate variability and salivary cortisol |
|  | Time of day does not modulate improvements in motor performance following a repetitive ballistic motor training task |
|  | Effects of cortisol on hippocampal subfields volumes and memory performance in healthy control subjects and patients with major depressive disorder |
|  | Noisy spit: parental noncompliance with child salivary cortisol sampling |
|  | Acute and chronic stress increase salivary cortisol: a study in the real-life setting of a national examination undertaken by medical graduates |
|  | Links between white matter microstructure and cortisol reactivity to stress in early childhood: evidence for moderation by parenting |
|  | Prenatal programming of emotion regulation: neonatal reactivity as a differential susceptibility factor moderating the outcome of prenatal cortisol levels |
|  | Maternal history of child abuse moderates the association between daily stress and diurnal cortisol in pregnancy: a pilot study |
|  | Suitability of saliva cortisol as a biomarker for hypothalamic-pituitary-adrenal axis activation assessment, effects of feeding actions, and immunostimulatory challenges in dairy cows |
|  | Comparison of salivary and calculated free cortisol levels during low and standard dose of ACTH stimulation tests in healthy volunteers |
|  | Placebo analgesia in patients with functional and organic abdominal pain: a fMRI study in IBS, UC and healthy volunteers |
|  | Differential expression of the corticosteroid receptors GR1, GR2 and MR in rainbow trout organs with slow release cortisol implants |
|  | Seasonal changes in CRF-I and urotensin I transcript levels in masu salmon: correlation with cortisol secretion during spawning |
|  | The DeStress for Success Program: effects of a stress education program on cortisol levels and depressive symptomatology in adolescents making the transition to high school |
|  | Diurnal patterns of salivary cortisol and DHEA using a novel collection device: electronic monitoring confirms accurate recording of collection time using this device |
|  | Distinct Trajectories of Cortisol Response to Prolonged Acute Stress Are Linked to Affective Responses and Hippocampal Gray Matter Volume in Healthy Females |
|  | Childhood interparental conflict and HPA axis activity in young adulthood: examining nonlinear relations |
|  | Analysis of glucose metabolism of (18)F-FDG in major depression patients using PET imaging: Correlation of salivary cortisol and α-amylase |
|  | Attenuated hypothalamic-pituitary-adrenal axis functioning predicts accelerated pubertal development in girls 1 year later |
|  | Periventricular white matter integrity and cortisol levels in healthy controls and in euthymic patients with bipolar disorder: an exploratory analysis |
|  | Neural mechanisms mediating positive and negative treatment expectations in visceral pain: a functional magnetic resonance imaging study on placebo and nocebo effects in healthy volunteers |
|  | Effortful control and context interact in shaping neuroendocrine stress responses during childhood |
|  | Influence of menarche on the relation between diurnal cortisol production and ventral striatum activity during reward anticipation |
|  | Cortisol responses to social evaluation in 10- to 15-year-old boys and girls |
|  | Commentary: The importance of exploring dose-dependent, subtype-specific, and age-related effects of maltreatment on the HPA axis and the mediating link to psychopathology. A response to Fisher (2017) |
|  | Noninvasive estimation of the hypothalamic-pituitary-adrenal system activity in the Far East leopard |
|  | In vitro effect of cortisol and urotensin I on arginine vasotocin and isotocin secretion from pituitary cells of gilthead sea bream Sparus aurata |
|  | Evaluation of the in vivo antioxidative activity of redox nanoparticles by using a developing chicken egg as an alternative animal model |
|  | Differential Responses of the HPA Axis to Mild Blast Traumatic Brain Injury in Male and Female Mice |
|  | Lower stress-reactive cortisol in female veterans associated with military status but not PTSD |
|  | Relocation and Hair Cortisol Concentrations in New Zealand White Rabbits |
|  | Misdiagnosis of hypocortisolemia based on early morning cortisol |
|  | Childhood adversity and cortisol habituation to repeated stress in adulthood |
|  | Adolescent Life Stress and the Cortisol Awakening Response: The Moderating Roles of Attachment and Sex |
|  | Social anxiety and the cortisol response to social evaluation in children and adolescents |
|  | Child anxiety symptoms related to longitudinal cortisol trajectories and acute stress responses: evidence of developmental stress sensitization |
|  | The behavioural, cognitive, and neural corollaries of blunted cardiovascular and cortisol reactions to acute psychological stress |
|  | Validation of autonomic and endocrine reactivity to a laboratory stressor in young children |
|  | The role of week(end)-day and awakening time on cortisol and alpha-amylase awakening responses |
|  | Further evidence of HPA-axis dysregulation and its correlation with depression in Autism Spectrum Disorders: Data from girls |
|  | Infants at familial risk for depression show a distinct pattern of cortisol response to experimental challenge |
|  | The time course of aggressive behaviour in juvenile matrinxã Brycon amazonicus fed with dietary L-tryptophan supplementation |
|  | Excessive Sugar Consumption May Be a Difficult Habit to Break: A View From the Brain and Body |
|  | Acute salivary cortisol response among Mexican American adolescents in immigrant families |
|  | Effects of a school readiness intervention on hypothalamus-pituitary-adrenal axis functioning and school adjustment for children in foster care |
|  | Cross-country differences in basal and stress-induced cortisol secretion in older adults |
|  | Using Actiwatch to monitor circadian rhythm disturbance in Huntington' disease: A cautionary note |
|  | Children's hair cortisol and hair testosterone concentrations in response to environmental changes: from summer holidays to school |
|  | Exploring the effects of daily hassles and uplifts on eating behaviour in young adults: The role of daily cortisol levels |
|  | Sleep deprivation potentiates HPA axis stress reactivity in healthy adults |
|  | Stress-related dysfunction of the right inferior frontal cortex in high ruminators: An fNIRS study |
|  | Salivary cortisol: a possible biomarker in evaluating stress and effects of interventions in young foster children? |
|  | Neuroimaging evidence for a role of neural social stress processing in ethnic minority-associated environmental risk |
|  | Relationship between the hippocampal shape abnormality and serum cortisol levels in first-episode and drug-naïve major depressive disorder patients |
|  | Cortisol response mediates the effect of post-reactivation stress exposure on contextualization of emotional memories |
|  | Cortisol responses to a group public speaking task for adolescents: variations by age, gender, and race |
|  | Disentangling the effects of genetic, prenatal and parenting influences on children's cortisol variability |
|  | Repeated stimulation of the HPA axis alters white blood cell count without increasing oxidative stress or inflammatory cytokines in fasting elephant seal pups |
|  | Lost in translation? The potential psychobiotic Lactobacillus rhamnosus (JB-1) fails to modulate stress or cognitive performance in healthy male subjects |
|  | Corticotropic axis drive of overnight cortisol secretion is suppressed in adolescents and young adults with type 1 diabetes mellitus |
|  | Effects of Late Gestational Fetal Exposure to Dexamethasone Administration on the Postnatal Hypothalamus-Pituitary-Adrenal Axis Response to Hypoglycemia in Pigs |
|  | Hearing loss is associated with hippocampal atrophy and high cortisol/dehydroepiandrosterone sulphate ratio in older adults |
|  | Psychosocial stress affects the acquisition of cerebellar-dependent sensorimotor adaptation |
|  | Insomnia symptoms, objective sleep duration and hypothalamic-pituitary-adrenal activity in children |
|  | Dysregulated diurnal cortisol pattern is associated with glucocorticoid resistance in women with major depressive disorder |
|  | Fighting experience alters brain androgen receptor expression dependent on testosterone status |
|  | Changes of Dietary Fat and Carbohydrate Content Alter Central and Peripheral Clock in Humans |
|  | Epigenetic Programming by Maternal Behavior in the Human Infant |
|  | Acute stress effects on GABA and glutamate levels in the prefrontal cortex: A 7T (1)H magnetic resonance spectroscopy study |
|  | Psychobiotic supplementation of HK-PS23 improves anxiety in highly stressed clinical nurses: a double-blind randomized placebo-controlled study |
|  | Decreased hypothalamic functional connectivity with subgenual cortex in psychotic major depression |
|  | The relationship between handling time and cortisol release rates changes as a function of brain parasite densities in California killifish Fundulus parvipinnis |
|  | A Randomised Controlled Trial of Neuronavigated Repetitive Transcranial Magnetic Stimulation (rTMS) in Anorexia Nervosa |
|  | Molecular cloning and expression analysis of a novel caspase recruitment domain protein (CARD) in common carp Cyprinus carpio L |
|  | Major depression in young girls is related to altered cortisol awakening response |
|  | Estradiol levels modulate brain activity and negative responses to psychosocial stress across the menstrual cycle |
|  | Bullying Victimization Heightens Cortisol Response to Psychosocial Stress in Chinese Children |
|  | Limited response to CRH stimulation tests at 2 weeks of age in preterm infants born at less than 30 weeks of gestational age |
|  | Effects of low and high protein:carbohydrate ratios in the diet of pregnant gilts on maternal cortisol concentrations and the adrenocortical and sympathoadrenal reactivity in their offspring |
|  | Obstructive sleep apnea and neurocognitive performance: the role of cortisol |
|  | Greater lifetime stress exposure predicts blunted cortisol but heightened DHEA responses to acute stress |
|  | Differential expression of corticotropin-releasing factor (CRF) and urotensin I precursor genes, and evidence of CRF gene expression regulated by cortisol in goldfish brain |
|  | Stress leads to aberrant hippocampal involvement when processing schema-related information |
|  | Daily parental knowledge of youth activities is linked to youth physical symptoms and HPA functioning |
|  | Enduring Association Between Parenting and Cortisol: A Meta-analysis |
|  | Mind your thoughts: associations between self-generated thoughts and stress-induced and baseline levels of cortisol and alpha-amylase |
|  | The impact of the severity of early life stress on diurnal cortisol: The role of puberty |
|  | HPA axis response to social stress is attenuated in schizophrenia but normal in depression: evidence from a meta-analysis of existing studies |
|  | State variation in the cortisol awakening response |
|  | Comparative study of 17 β-estradiol on endocrine disruption and biotransformation in fingerlings and juveniles of Japanese sea bass Lateolabrax japonicus |
|  | Cortisol awakening response and developmental outcomes at 6-7 years in children born extremely preterm |
|  | Coptidis Rhizoma Prevents Heat Stress-Induced Brain Damage and Cognitive Impairment in Mice |
|  | A vertebrate-conserved cis-regulatory module for targeted expression in the main hypothalamic regulatory region for the stress response |
|  | Correlates of cortisol in human hair: implications for epidemiologic studies on health effects of chronic stress |
|  | DHEA Moderates the Impact of Childhood Trauma on the HPA Axis in Adolescence |
|  | Influence of acute stress on response inhibition in healthy men: An ERP study |
|  | Affective alterations in patients with Cushing's syndrome in remission are associated with decreased BDNF and cortisone levels |
|  | The role of corticosteroid-binding globulin in the evaluation of adrenal insufficiency |
|  | Increased hair testosterone but unaltered hair cortisol in female patients with borderline personality disorder |
|  | Attachment dimensions and cortisol responses during the strange situation among young children adopted internationally |
|  | The calming effect of roasted coffee aroma in patients undergoing dental procedures |
|  | Noradrenergic Activity in the Human Brain: A Mechanism Supporting the Defense Against Hypoglycemia |
|  | Hypothalamic-pituitary-adrenal axis dysfunction in non-clinical psychosis |
|  | Is cortisol production in response to an acute stressor associated with diurnal cortisol production during adolescence? |
|  | Increased metabotropic glutamate receptor subtype 5 availability in human brain after one night without sleep |
|  | Tyrosine hydroxylase in the ventral tegmental area of rams with high or low libido-A role for dopamine |
|  | Dominance rank causally affects personality and glucocorticoid regulation in female rhesus macaques |
|  | The levonorgestrel-releasing intrauterine device potentiates stress reactivity |
|  | Effects of combined IUGR and prenatal stress on the development of the hippocampus in a fetal guinea pig model |
|  | Interleukin-17- and interleukin-22-secreting myelin-specific CD4(+) T cells resistant to corticoids are related with active brain lesions in multiple sclerosis patients |
|  | Sex-specific prediction of hypothalamic-pituitary-adrenal axis activity by pituitary volume during adolescence: a longitudinal study from 12 to 17 years of age |
|  | The relation between gaze aversion and cortisol reactivity in middle childhood |
|  | HPA-axis function and grey matter volume reductions: imaging the diathesis-stress model in individuals at ultra-high risk of psychosis |
|  | Hypothalamic-pituitary-adrenal axis activity in the comorbidity between obsessive-compulsive disorder and major depression |
|  | Allopregnanolone serum concentrations and diurnal cortisol secretion in women with premenstrual dysphoric disorder |
|  | Food-anticipatory activity in Syrian hamsters: behavioral and molecular responses in the hypothalamus according to photoperiodic conditions |
|  | Relative influences: patterns of HPA axis concordance during triadic family interaction |
|  | Metabolic changes in serum steroids for diagnosing and subtyping Cushing's syndrome |
|  | Salivary biomarkers of obstructive sleep apnea syndrome in children |
|  | Salivary cortisol and cognitive development in infants from low-income communities |
|  | Do different data analytic approaches generate discrepant findings when measuring mother-infant HPA axis attunement? |
|  | Measurement of cortisol concentration in the tears of horses and ponies with pituitary pars intermedia dysfunction |
|  | Cocaine- and amphetamine-regulated transcript peptide (CART) in the central nucleus of amygdala potentiates behavioral and hormonal responses of the rat exposed to its predator |
|  | Stress and strategic decision-making in the beauty contest game |
|  | Hair cortisol levels in pregnancy as a possible determinant of fetal sex: a longitudinal study |
|  | Difficulties with emotion regulation moderate the association between childhood history of maltreatment and cortisol reactivity to psychosocial challenge in postpartum women |
|  | Investigating the effect of acute sleep deprivation on hypothalamic-pituitary-adrenal-axis response to a psychosocial stressor |
|  | No association of cigarette smoking and depressive symptoms with cortisol concentration in adolescents. Results from a population-based Swedish cohort |
|  | Anxiety and depressive symptoms effects on cortisol trajectories from pregnancy to postpartum: Differences and similarities between women and men |
|  | Social context matters: Ethnicity, discrimination and stress reactivity |
|  | Adversity-driven changes in hypothalamic-pituitary-adrenal axis functioning during adolescence. The trails study |
|  | Stress/Immune Biomarkers in Saliva among Children with ADHD Status |
|  | Now you see it, now you don't: Testing environments modulate the association between hippocampal volume and cortisol levels in young and older adults |
|  | Childhood stress, grown-up brain networks: corticolimbic correlates of threat-related early life stress and adult stress response |
|  | Temporal patterns, heterogeneity, and stability of diurnal cortisol rhythms in children with autism spectrum disorder |
|  | One left dorsolateral prefrontal cortical HF-rTMS session attenuates HPA-system sensitivity to critical feedback in healthy females |
|  | Self- or parent report of (co-occurring) internalizing and externalizing problems, and basal or reactivity measures of HPA-axis functioning: a systematic evaluation of the internalizing-hyperresponsivity versus externalizing-hyporesponsivity HPA-axis hypothesis |
|  | Gut microbiota regulates mouse behaviors through glucocorticoid receptor pathway genes in the hippocampus |
|  | Interparental conflict and child HPA-axis responses to acute stress: Insights using intensive repeated measures |
|  | Individual and Day-to-Day Differences in Active Coping Predict Diurnal Cortisol Patterns among Early Adolescent Girls |
|  | Hypothalamic-pituitary-adrenal axis measures and cognitive abilities in early psychosis: Are there sex differences? |
|  | Overall cortisol, diurnal slope, and stress reactivity in psychosis: An experience sampling approach |
|  | Hypothalamus-Pituitary-Adrenal-axis activity and cognitive functioning in older adults |
|  | A test of maternal programming of offspring stress response to predation risk in threespine sticklebacks |
|  | Psychological and environmental correlates of HPA axis functioning in parentally bereaved children: preliminary findings |
|  | Perinatal hypothalamic-pituitary-adrenal axis regulation among women with eating disorders and their infants |
|  | Alopecia areata - hyperactivity of the hypothalamic-pituitary-adrenal axis is a myth? |
|  | Recent advances in cortisol sensing technologies for point-of-care application |
|  | Children's biological responsivity to acute stress predicts concurrent cognitive performance |
|  | Is there an association between work stress and diurnal cortisol patterns? Findings from the Whitehall II study |
|  | Probing the association between dexamethasone-induced cortisol suppression and serotonin transporter availability among drug-free patients with major depressive disorder--a small-sample SPECT study with [¹²³I]ADAM |
|  | Salivary cortisol reactivity in preschoolers is associated with hair cortisol and behavioral problems |
|  | Disparate plasma cortisol concentrations in sexually abused female children from Johannesburg, South Africa |
|  | Is there a viability-vulnerability tradeoff? Sex differences in fetal programming |
|  | Selective social buffering of behavioral and endocrine responses and Fos induction in the prelimbic cortex of infants exposed to a novel environment |
|  | Maternal hypothalamic-pituitary-adrenal axis response to foraging uncertainty: A model of individual vs. social allostasis and the "Superorganism Hypothesis" |
|  | Hair hormones in male youth with internet gaming disorder |
|  | Sex differences in morning cortisol in youth at ultra-high-risk for psychosis |
|  | Cortisol-dehydroepiandrosterone ratios are inversely associated with hippocampal and prefrontal brain volume in schizophrenia |
|  | Stress, Psychological Resources, and HPA and Inflammatory Reactivity During Late Adolescence |
|  | Modulatory mechanisms of cortisol effects on emotional learning and memory: novel perspectives |
|  | The 5-HT(2C) receptor agonist meta-chlorophenylpiperazine (mCPP) reduces palatable food consumption and BOLD fMRI responses to food images in healthy female volunteers |
|  | Higher post-encoding cortisol benefits the selective consolidation of emotional aspects of memory |
|  | Transcranial Stimulation of the Dorsolateral Prefrontal Cortex Prevents Stress-Induced Working Memory Deficits |
|  | Effects of acute psychological stress induced in laboratory on physiological responses in asthma populations: A systematic review |
|  | Working memory is differentially affected by stress in men and women |
|  | Neonatal amygdala lesions lead to increased activity of brain CRF systems and hypothalamic-pituitary-adrenal axis of juvenile rhesus monkeys |
|  | Diurnal cortisol rhythms in youth from risky families: effects of cumulative risk exposure and variation in the serotonin transporter gene-linked polymorphic region (5-HTTLPR) [corrected] |
|  | Salivary cortisol levels and the 2-year course of depressive and anxiety disorders |
|  | Mini-review of hair cortisol concentration for evaluation of Cushing syndrome |
|  | Intimate partner violence is associated with increased maternal hair cortisol in mother-child dyads |
|  | Feasibility of Hair Collection for Cortisol Measurement in Population Research on Adolescent Health |
|  | Idle behaviors of the hippocampus reflect endogenous cortisol levels in youth |
|  | The Involvement of the Hypothalamus-Pituitary-Adrenal Axis in the Development of Hyperalgesia during the Early Postoperative Period |
|  | In abstinent MDMA users the cortisol awakening response is off-set but associated with prefrontal serotonin transporter binding as in non-users |
|  | Hair cortisol in drug-naïve first-episode individuals with psychosis |
|  | Stress axis regulation during social ascension in a group-living cichlid fish |
|  | Variability of the cortisol awakening response and morning salivary oxytocin in late adolescence |
|  | Blunted endocrine response to a combined physical-cognitive stressor in adults with early life adversity |
|  | Dual-hormone stress reactivity predicts downstream war-zone stress-evoked PTSD |
|  | Cortisol reactivity and suicidal behavior: Investigating the role of hypothalamic-pituitary-adrenal axis responses to stress in suicide attempters and ideators |
|  | Change in parent-child conflict and the HPA-axis: Where should we be looking and for how long? |
|  | The role of cortisol and psychopathy in the cycle of violence |
|  | Glucagon, insulin, adrenocorticotropic hormone, and cortisol in response to carbohydrates and fasting in healthy neonatal foals |
|  | Adrenocorticotropic hormone in serial cerebrospinal fluid in man - Subject to acute regulation by the hypothalamic-pituitary-adrenocortical system? |
|  | Classroom social experiences in early elementary school relate to diurnal cortisol levels |
|  | COMT(val158met) polymorphism is associated with behavioral response and physiologic reactivity to socio-emotional stress in 4-month-old infants |
|  | No evidence of a longitudinal association between diurnal cortisol patterns and cognition |
|  | Pubertal recalibration of cortisol reactivity following early life parent-child separation |
|  | Subjective insomnia symptoms and sleep duration are not related to hypothalamic-pituitary-adrenal axis activity in older adults |
|  | Neonatal amygdala lesions alter basal cortisol levels in infant rhesus monkeys |
|  | Salivary and hair glucocorticoids and sleep in very preterm children during school age |
|  | A meta-analytic review of the impact of intranasal oxytocin administration on cortisol concentrations during laboratory tasks: moderation by method and mental health |
|  | Stress reactivity predicts symptom improvement in children with anxiety disorders |
|  | Increased cortisol awakening response was associated with time to recurrence of major depressive disorder |
|  | Prospective associations between the cortisol awakening response and first onsets of anxiety disorders over a six-year follow-up--2013 Curt Richter Award Winner |
|  | Maternal lipids in pregnancy are associated with increased offspring cortisol reactivity in childhood |
|  | Prenatal Stress and the Cortisol Awakening Response in African-American and Caucasian Women in the Third Trimester of Pregnancy |
|  | Gender roles are related to cortisol habituation to repeated social evaluative stressors in adults: secondary analyses from a randomized controlled trial |
|  | The perinatal effects of maternal caffeine intake on fetal and neonatal brain levels of testosterone, estradiol, and dihydrotestosterone in rats |
|  | Gonadotropin inhibitory hormone and RF9 stimulate hypothalamic-pituitary-adrenal axis in adult male rhesus monkeys |
|  | Maternal cortisol stimulates neurogenesis and affects larval behaviour in zebrafish |
|  | Modeling the influence of chronopharmacological administration of synthetic glucocorticoids on the hypothalamic-pituitary-adrenal axis |
|  | The longitudinal association of the diurnal cortisol rhythm with internalizing and externalizing problems in pre-schoolers. The Generation R Study |
|  | Social subordination stress and serotonin transporter polymorphisms: associations with brain white matter tract integrity and behavior in juvenile female macaques |
|  | Stress Induces a Shift Towards Striatum-Dependent Stimulus-Response Learning via the Mineralocorticoid Receptor |
|  | Acute stress enhances pupillary responses to erotic nudes: Evidence for differential effects of sympathetic activation and cortisol |
|  | Antenatal glucocorticoid treatment is associated with diurnal cortisol regulation in term-born children |
|  | A functional variant in the neuropeptide S receptor 1 gene moderates the influence of urban upbringing on stress processing in the amygdala |
|  | Neuroendocrine alterations in the exercising human: implications for energy homeostasis |
|  | Iron-induced neuronal damage in a rat model of post-traumatic stress disorder |
|  | Sympathetic and hypothalamic-pituitary-adrenal asymmetry in generalized anxiety disorder |
|  | Cortisol Response to Psychosocial Stress in Chinese Early Puberty Girls: Possible Role of Depressive Symptoms |
|  | Age-dependent and gender-dependent regulation of hypothalamic-adrenocorticotropic-adrenal axis |
|  | Twenty-four hour urinary cortisol excretion and the metabolic syndrome in prednisolone-treated renal transplant recipients |
|  | Examining habituation and sensitization across repetitive laboratory stress inductions using the MAST |
|  | Trait positive and negative emotionality differentially associate with diurnal cortisol activity |
|  | Effects of acute and chronic stress on telencephalic neurochemistry and gene expression in rainbow trout (Oncorhynchus mykiss) |
|  | Changes in salivary oxidative status, salivary cortisol, and clinical symptoms in female patients with temporomandibular disorders during occlusal splint therapy: a 3-month follow up |
|  | Dexamethasone facilitates fear extinction and safety discrimination in PTSD: A placebo-controlled, double-blind study |
|  | Diurnal salivary cortisol is associated with body mass index and waist circumference: the Multiethnic Study of Atherosclerosis |
|  | A traditional Korean multiple herbal formulae (Yuk-Mi-Jihwang-Tang) attenuates acute restraint stress-induced brain tissue oxidation |
|  | The immune responses and expression of metallothionein (MT) gene and heat shock protein 70 (HSP 70) in juvenile rockfish, Sebastes schlegelii, exposed to waterborne arsenic (As(3+)) |
|  | Hair cortisol as a hypothalamic-pituitary-adrenal axis biomarker in pregnant women with asthma: a retrospective observational study |
|  | Childhood maltreatment, pubertal development, HPA axis functioning, and psychosocial outcomes: An integrative biopsychosocial model |
|  | Prevention of pneumonia after severe traumatic brain injury |
|  | Associations among maternal socioeconomic status in childhood and pregnancy and hair cortisol in pregnancy |
|  | Does the cortisol awakening response link childhood adversity to adult BMI? |
|  | Intervening to enhance cortisol regulation among children at risk for neglect: Results of a randomized clinical trial |
|  | Corticotherapy for traumatic brain-injured patients--the Corti-TC trial: study protocol for a randomized controlled trial |
|  | Lack of significant association between type 2 diabetes mellitus with longitudinal change in diurnal salivary cortisol: the multiethnic study of atherosclerosis |
|  | Maternal sensitivity and infant autonomic and endocrine stress responses |
|  | Ambulatory Assessment Characteristics Predict the Clinical Course of Premenstrual Dysphoric Disorder |
|  | Stress system dysregulation in pediatric generalized anxiety disorder associated with comorbid depression |
|  | Longitudinal effects of the SSRI paroxetine on salivary cortisol in Major Depressive Disorder |
|  | The Hypothalamic-Pituitary-Adrenal Axis: A Brief History |
|  | Stress reactivity in maltreated and comparison male and female young adolescents |
|  | Maternal Prenatal Depression in Pregnancies With Female and Male Fetuses and Developmental Associations With C-reactive Protein and Cortisol |
|  | Age of Trauma Onset and HPA Axis Dysregulation Among Trauma-Exposed Youth |
|  | Bifidobacterium longum 1714 as a translational psychobiotic: modulation of stress, electrophysiology and neurocognition in healthy volunteers |
|  | Children's cortisol response to the transition from preschool to formal schooling: A review |
|  | Negative cognitive style and cortisol recovery accentuate the relationship between life stress and depressive symptoms |
|  | Physiological attunement in mother-infant dyads at clinical high risk: The influence of maternal depression and positive parenting |
|  | Refining the multisystem view of the stress response: coordination among cortisol, alpha-amylase, and subjective stress in response to relationship conflict |
|  | Associations between Sleep, Cortisol Regulation, and Diet: Possible Implications for the Risk of Alzheimer Disease |
|  | The HPA axis in bipolar disorder: Systematic review and meta-analysis |
|  | Salivary biomarkers of neural hypervigilance in trauma-exposed women |
|  | Trait mindfulness predicts the presence but not the magnitude of cortisol responses to acute stress |
|  | Adult males buffer the cortisol response of young guinea pigs: Changes with age, mediation by behavior, and comparison with prefrontal activity |
|  | The relationship between cannabis use and cortisol levels in youth at ultra high-risk for psychosis |
|  | Self-critical perfectionism predicts lower cortisol response to experimental stress in patients with chronic fatigue syndrome |
|  | Multiple measures of HPA axis function in ultra high risk and first-episode schizophrenia patients |
|  | Effect of dietary carbohydrates and time of year on ACTH and cortisol concentrations in adult and aged horses |
|  | Brief report: Neighborhood disadvantage and hair cortisol among older urban African Americans |
|  | Trait and state rumination interact to prolong cortisol activation to psychosocial stress in females |
|  | Are flatter diurnal cortisol rhythms associated with major depression and anxiety disorders in late adolescence? The role of life stress and daily negative emotion |
|  | Stress and the city: impact of urban upbringing on the (re)activity of the hypothalamus-pituitary-adrenal axis |
|  | HPA axis dysregulation in adult adoptees twenty years after severe institutional deprivation in childhood |
|  | The Levels of Cortisol, Oxidative Stress, and DNA Damage in the Victims of Childhood Sexual Abuse: A Preliminary Study |
|  | The Relationship between Cortisol Activity during Cognitive Task and Posttraumatic Stress Symptom Clusters |
|  | Acute cortisol reactivity attenuates engagement of fronto-parietal and striatal regions during emotion processing in negative mood disorders |
|  | Identification and replication of a combined epigenetic and genetic biomarker predicting suicide and suicidal behaviors |
|  | The cortisol awakening response predicts response inhibition in the afternoon of the same day |
|  | HPA and SAM axis responses as correlates of self- vs parental ratings of anxiety in boys with an Autistic Disorder |
|  | Endocannabinoid hydrolase and cannabinoid receptor 1 are involved in the regulation of hypothalamus-pituitary-adrenal axis in type 2 diabetes |
|  | Spatial patterns of the exposure-response relationship between mercury and cortisol in the fur of river otter (Lontra canadensis) |
|  | Diurnal Cortisol Interacts With Stressful Events to Prospectively Predict Depressive Symptoms in Adolescent Girls |
|  | ADRA2B genotype differentially modulates stress-induced neural activity in the amygdala and hippocampus during emotional memory retrieval |
|  | Biology, genes, and resilience: toward a multidisciplinary approach |
|  | Habitual sleep quality and diurnal rhythms of salivary cortisol and dehydroepiandrosterone in postmenopausal women |
|  | HPA regulation and dating couples' behaviors during conflict: gender-specific associations and cross-partner interactions |
|  | Peer victimization and diurnal cortisol rhythm among children affected by parental HIV: Mediating effects of emotional regulation and gender differences |
|  | Stressful life events, relationship stressors, and cortisol reactivity: The moderating role of suppression |
|  | The Pressure-Activation-Stress scale in relation to ADHD and cortisol |
|  | Primary (autoimmune) hypophysitis: a single centre experience |
|  | Predicting developmental changes in internalizing symptoms: examining the interplay between parenting and neuroendocrine stress reactivity |
|  | A new way of thinking: hydrocortisone in traumatic brain-injured patients |
|  | The concept of the immune-pineal axis tested in patients undergoing an abdominal hysterectomy |
|  | Free and total plasma cortisol measured by immunoassay and mass spectrometry following ACTH₁₋₂₄ stimulation in the assessment of pituitary patients |
|  | Circadian arrhythmia dysregulates emotional behaviors in aged Siberian hamsters |
|  | Cortisol Concentration as Predictor of Tobacco Initiation in Adolescents: Results From a Population-Based Swedish Cohort |
|  | Altered circadian patterns of salivary cortisol in low-functioning children and adolescents with autism |
|  | Social Buffering of Stress in Development: A Career Perspective |
|  | Use of Glucocorticoids for the Fetus and Preterm Infant |
|  | Non-thrombotic superior sagittal sinus occlusion with intracranial hypertension following metastatic Burkitt's lymphoma |
|  | Loneliness, HPA stress reactivity and social threat sensitivity: Analyzing naturalistic social challenges |
|  | The role of genetic variation in the glucocorticoid receptor (NR3C1) and mineralocorticoid receptor (NR3C2) in the association between cortisol response and cognition under acute stress |
|  | Prolonged fasting impairs neural reactivity to visual stimulation |
|  | The Impact of COVID-19 Viral Infection on the Hypothalamic-Pituitary-Adrenal Axis |
|  | Comparison of Hair Cortisol Levels and Perceived Stress in Mothers Who Deliver at Preterm and Term |
|  | The influence of childhood abuse on cortisol levels and the cortisol awakening response in depressed and nondepressed older adults |
|  | Prematurity and perinatal adversity effects hypothalamic-pituitary-adrenal axis reactivity to social evaluative threat in adulthood |
|  | Circadian clock control of endocrine factors |
|  | Association between changes in heart rate variability during the anticipation of a stressful situation and the stress-induced cortisol response |
|  | Altered functioning of the HPA axis in depressed postpartum women |
|  | Cortisol levels and cognitive profile in major depression: A comparison of currently and previously depressed patients |
|  | Hair Cortisol Concentrations in Adolescent Girls with Anorexia Nervosa are Lower Compared to Healthy and Psychiatric Controls |
|  | Cortisol response to stress in caregivers of offspring with autism spectrum disorder is associated with care recipient characteristics |
|  | Rumination and impaired cortisol recovery following a social stressor in adolescent depression |
|  | Auditory environmental enrichment prevents anxiety-like behavior, but not cortisol responses, evoked by 24-h social isolation in zebrafish |
|  | Pre-encoding stress induced changes in perceived stress, blood pressure and cortisol are differentially associated with recollection and familiarity |
|  | The effects of trait and state affect on diurnal cortisol slope among children affected by parental HIV/AIDS in rural China |
|  | Cortisol awakening response in adolescents with acute sexual abuse related posttraumatic stress disorder |
|  | Individual prolactin reactivity modulates response of nucleus accumbens to erotic stimuli during acute cannabis intoxication: an fMRI pilot study |
|  | Cortisol dysregulation: the bidirectional link between stress, depression, and type 2 diabetes mellitus |
|  | No effects of hydrocortisone and dexamethasone on pain sensitivity in healthy individuals |
|  | Cortisol and salivary alpha-amylase trajectories following a group social-evaluative stressor with adolescents |
|  | Intra-individual cortisol variability and low-grade inflammation over 10 years in older adults |
|  | Cortisol Stress Response Variability in Early Adolescence: Attachment, Affect and Sex |
|  | The validity of individual frontal alpha asymmetry EEG neurofeedback |
|  | The effect of lipopolysaccharide (LPS) on inflammatory markers in blood and brain and on behavior in individually-housed pigs |
|  | Pituitary size alteration and adverse effects of radiation therapy performed in 9 dogs with pituitary-dependent hypercortisolism |
|  | NMDA receptor modulation by dextromethorphan and acute stress selectively alters electroencephalographic indicators of partial report processing |
|  | Two-year stability of individual differences in (para)sympathetic and HPA-axis responses to public speaking in childhood and adolescence |
|  | Sustained action of developmental ethanol exposure on the cortisol response to stress in zebrafish larvae and adults |
|  | Multi-Level Risk Factors for Suicidal Ideation Among at-Risk Adolescent Females: The Role of Hypothalamic-Pituitary-Adrenal Axis Responses to Stress |
|  | Maternal influence on child HPA axis: a prospective study of cortisol levels in hair |
|  | Parental cannabis and tobacco use during pregnancy and childhood hair cortisol concentrations |
|  | Trauma exposure relates to heightened stress, altered amygdala morphology and deficient extinction learning: Implications for psychopathology |
|  | The association between cortisol, oxytocin, and immune cell mitochondrial oxygen consumption in postpartum women with childhood maltreatment |
|  | Systematic review and meta-analysis reveals acutely elevated plasma cortisol following fasting but not less severe calorie restriction |
|  | Transcriptome comparison in the pituitary-adrenal axis between Beagle and Chinese Field dogs after chronic stress exposure |
|  | Problematic drinking and physiological responses among female college students |
|  | Cancer-related fatigue shows a stable association with diurnal cortisol dysregulation in breast cancer patients |
|  | Adiposity and Cortisol Response to Stress in Indian Adolescents |
|  | Population density-dependent hair cortisol concentrations in rhesus monkeys (Macaca mulatta) |
|  | Co-variation of fatigue and psychobiological stress in couples' everyday life |
|  | Blunted diurnal cortisol pattern is associated with frailty: a cross-sectional study of 745 participants aged 65 to 90 years |
|  | Intraindividual variability in cortisol: Approaches, illustrations, and recommendations |
|  | Rapid cortisol enhancement of psychomotor and startle reactions to side-congruent stimuli in a focused cross-modal choice reaction time paradigm |
|  | Adrenal insufficiency in critical patients: New ethiopathogenic concepts and therapeutic implications |
|  | The behavioral and endocrinological development of stress response in dogs |
|  | Depressive symptom composites associated with cortisol stress reactivity in adolescents |
|  | Gestational cortisol and social play shape development of marmosets' HPA functioning and behavioral responses to stressors |
|  | Longitudinal associations between infections and atopic disorders across childhood and dysregulated adrenocortical functioning in early adolescence |
|  | Cortisol and Subjective Stress Responses to Acute Psychosocial Stress in Fibromyalgia Patients and Control Participants |
|  | Pain-related stress in the Neonatal Intensive Care Unit and salivary cortisol reactivity to socio-emotional stress in 3-month-old very preterm infants |
|  | The effects of birth timing and ambient temperature on the hypothalamic-pituitary-adrenal axis in 3-4 month old rhesus monkeys |
|  | Mechanisms underlying the effects of prenatal psychosocial stress on child outcomes: beyond the HPA axis |
|  | In search of the HPA axis activity in unipolar depression patients with childhood trauma: Combined cortisol awakening response and dexamethasone suppression test |
|  | The role of autosuggestion in geriatric patients' quality of life: a study on psycho-neuro-endocrine-immunology pathway |
|  | Pro-inflammatory and anti-inflammatory compounds exert similar effects on P-glycoprotein in blood-brain barrier endothelial cells |
|  | Course of ante- and postnatal depressive symptoms related to mothers' HPA axis regulation |
|  | Long term effects of childhood trauma on cortisol stress reactivity in adulthood and relationship to the occurrence of depression |
|  | Early Life Vitamin C Deficiency Does Not Alter Morphology of Hippocampal CA1 Pyramidal Neurons or Markers of Synaptic Plasticity in a Guinea Pig Model |
|  | Distinct activation of the sympathetic adreno-medullar system and hypothalamus pituitary adrenal axis following the caloric vestibular test in healthy subjects |
|  | The effect of hydrocortisone administration on intertemporal choice |
|  | Endogenous Glucocorticoid Response to Single-Dose Dexamethasone for Croup in Children: A Pharmacodynamic Study |
|  | Testosterone and cortisol responses to acute and prolonged stress during officer training school |
|  | Transcriptome sequencing of the choroid plexus in schizophrenia |
|  | Social context affects behavior, preoptic area gene expression, and response to D2 receptor manipulation during territorial defense in a cichlid fish |
|  | Childhood adversity moderates the influence of proximal episodic stress on the cortisol awakening response and depressive symptoms in adolescents |
|  | Response of the Hypothalamic-Pituitary-Adrenal System to Repeated Moderate Psychoemotional Stress Exposure Is Associated with Behavioral Parameters |
|  | Is salivary alpha-amylase an indicator of autonomic nervous system dysregulations in mental disorders?--a review of preliminary findings and the interactions with cortisol |
|  | Stronger hypothalamus-pituitary-adrenal axis habituation predicts lesser sensitization of inflammatory response to repeated acute stress exposures in healthy young adults |
|  | Is the Correlation between Salivary Cortisol and Serum Cortisol Reliable Enough to Enable Use of Salivary Cortisol Levels in Preterm Infants? |
|  | Evaluation of the hypothalamic-pituitary-adrenal axis and its relationship with central respiratory dysfunction in children with Prader-Willi syndrome |
|  | Relationship between function of hypothalamic-pituitary-adrenal axis and executive functions in chronic methamphetamine users: A cross-sectional study |
|  | Social change and access to a palatable diet produces differences in reward neurochemistry and appetite in female monkeys |
|  | Salivary cortisol reveals overt and hidden anxiety in survivors of childhood cancer attending clinic |
|  | Neurobiology: Rise of resilience |
|  | The influence of comorbid depression and overweight status on peripheral inflammation and cortisol levels |
|  | HPA-axis activity and the moderating effect of self-esteem in the context of intimate partner violence in Cameroon |
|  | Recent and long-term occupational noise exposure and salivary cortisol level |
|  | Maternal depression across the first years of life compromises child psychosocial adjustment; relations to child HPA-axis functioning |
|  | Salivary diurnal cortisol profiles in patients suffering from chronic breathlessness receiving supportive and palliative care services: A cross-sectional study |
|  | A pilot investigation of differential neuroendocrine associations with fronto-limbic activation during semantically-cued list learning in mood disorders |
|  | Cultural neurobiology and the family: Evidence from the daily lives of Latino adolescents |
|  | Sex differences in the association between internalizing symptoms and hair cortisol level among 10-12 year-old adolescents in China |
|  | Evidence for a normal HPA axis response to psychosocial stress in patients remitted from depression |
|  | Understanding the unfolding of stress regulation in infants |
|  | Post-dexamethasone cortisol, self-inflicted injury, and suicidal ideation among depressed adolescent girls |
|  | Social opportunity rapidly regulates expression of CRF and CRF receptors in the brain during social ascent of a teleost fish, Astatotilapia burtoni |
|  | Striatal Hypersensitivity During Stress in Remitted Individuals with Recurrent Depression |
|  | Assessment of tissue-specific cortisol activity with regard to degeneration of the suspensory ligaments in horses with pituitary pars intermedia dysfunction |
|  | Emotional attentional control predicts changes in diurnal cortisol secretion following exposure to a prolonged psychosocial stressor |
|  | Maternal stress, placental 11β-hydroxysteroid dehydrogenase type 2, and infant HPA axis development in humans: Psychosocial and physiological pathways |
|  | A multiparametric approach to discriminate the impacts of different degrees of invasiveness of surgical procedures in sheep |
|  | A functional polymorphism in the HTR2C gene associated with stress responses: a validation study |
|  | Sex-related differences in the association of salivary cortisol levels and type 2 diabetes. Findings from the cross-sectional population based KORA-age study |
|  | Increased hair cortisol and antecedent somatic complaints in children with a first epileptic seizure |
|  | HPA Axis Genes, and Their Interaction with Childhood Maltreatment, are Related to Cortisol Levels and Stress-Related Phenotypes |
|  | Socioeconomic status and the cerebellar grey matter volume. Data from a well-characterised population sample |
|  | Elevated brain cannabinoid CB1 receptor availability in post-traumatic stress disorder: a positron emission tomography study |
|  | Adrenocortical Stress Response during the Course of Critical Illness |
|  | Do depressive symptoms mediate the relationship between hopelessness and diurnal cortisol rhythm? |
|  | Noninvasive surrogate markers for plasma cortisol in newborn infants: utility of urine and saliva samples and caution for venipuncture blood samples |
|  | Analyses of fecal and hair glucocorticoids to evaluate short- and long-term stress and recovery of Asiatic black bears (Ursus thibetanus) removed from bile farms in China |
|  | The Interaction of Childhood Maltreatment, Sex, and Borderline Personality Features in the Prediction of the Cortisol Awakening Response in Adolescents |
|  | Endogenous cortisol reactivity moderates the relationship between fear inhibition to safety signals and posttraumatic stress disorder symptoms |
|  | Serum cortisol and adrenocorticotrophic hormone (ACTH) in infants receiving topical and subconjunctival corticosteroids following cataract surgery |
|  | MANAGEMENT OF ENDOCRINE DISEASE: Glucocorticoid-induced adrenal insufficiency: replace while we wait for evidence? |
|  | Hyper- and hypocortisolism in bipolar disorder - A beneficial influence of lithium on the HPA-axis? |
|  | Depressed suicidal adolescent males have an altered cortisol response to a pharmacological challenge |
|  | A Life Course Approach to the Relationship Between Fetal Growth and Hypothalamic-Pituitary-Adrenal Axis Function |
|  | Prolonged performance-related neuroendocrine activation and perseverative cognition in low- and high-anxious university music students |
|  | Cortisol levels in former preterm children at school age are predicted by neonatal procedural pain-related stress |
|  | Are cortisol and melatonin involved in the immune modulation by the light environment in pike perch Sander lucioperca? |
|  | Attachment security buffers the HPA axis of toddlers growing up in poverty or near poverty: Assessment during pediatric well-child exams with inoculations |
|  | Salivary alpha-amylase, secretory IgA and free cortisol as neurobiological components of the stress response in the acute phase of anorexia nervosa |
|  | A prospective study of the hypothalamic-pituitary-adrenal axis in children with acute lymphoblastic leukemia receiving chemotherapy |
|  | Relational victimization, friendship, and adolescents' hypothalamic-pituitary-adrenal axis responses to an in vivo social stressor |
|  | Parental support buffers the association of depressive symptoms with cortisol and C-reactive protein during adolescence |
|  | Genetic, molecular and clinical determinants for the involvement of aldosterone and its receptors in major depression |
|  | Interactive effects of early life stress and CACNA1C genotype on cortisol awakening response |
|  | The cortisol reactivity threshold model: Direction of trait rumination and cortisol reactivity association varies with stressor severity |
|  | Familial Risk for Insomnia Is Associated With Abnormal Cortisol Response to Stress |
|  | Daily diary reports of social connection, objective sleep, and the cortisol awakening response during adolescents' first year of college |
|  | Endocrine consequences of anorexia nervosa |
|  | Blunted cortisol awakening response in men with first episode psychosis: relationship to parental bonding |
|  | The influence of sleep on human hypothalamic-pituitary-adrenal (HPA) axis reactivity: A systematic review |
|  | Interaction of adrenocortical activity and autonomic arousal on children's externalizing and internalizing behavior problems |
|  | Social deprivation and the HPA axis in early development |
|  | A longitudinal study of several potential mediators of the relationship between child maltreatment and posttraumatic stress disorder symptoms |
|  | Acute stress impairs frontocingulate activation during error monitoring in remitted depression |
|  | Unhealthy lifestyle in early psychoses: the role of life stress and the hypothalamic-pituitary-adrenal axis |
|  | Hydrocortisone and fludrocortisone for prevention of hospital-acquired pneumonia in patients with severe traumatic brain injury (Corti-TC): a double-blind, multicentre phase 3, randomised placebo-controlled trial |
|  | Vasopressin, steroids, and epinephrine and neurologically favorable survival after in-hospital cardiac arrest: a randomized clinical trial |
|  | Toxic stress, health and nutrition among Brazilian children in shelters |
|  | Persistence of Cushing's disease symptoms and comorbidities after surgical cure: a long-term, integral evaluation |
|  | Reunion behavior after social separation is associated with enhanced HPA recovery in young marmoset monkeys |
|  | High hair cortisol concentrations predict worse cognitive outcome after stroke: Results from the TABASCO prospective cohort study |
|  | Mifepristone reduces insulin resistance in patient volunteers with adrenal incidentalomas that secrete low levels of cortisol: a pilot study |
|  | Lack of effect on adult and adolescent hypothalamic-pituitary-adrenal axis function with use of fluticasone furoate nasal spray |
|  | The stress model of chronic pain: evidence from basal cortisol and hippocampal structure and function in humans |
|  | Relationship between endogenous plasma adrenocorticotropic hormone concentration and reproductive performance in Thoroughbred broodmares |
|  | ABCC1 confers tissue-specific sensitivity to cortisol versus corticosterone: A rationale for safer glucocorticoid replacement therapy |
|  | Maternal supplementation with fishmeal protects against late gestation endotoxin-induced fetal programming of the ovine hypothalamic-pituitary-adrenal axis |
|  | Genetic parameters of hair cortisol as an indicator of chronic stress under different environments in Holstein cows |
|  | Intermittent pair-housing, pair relationship qualities, and HPA activity in adult female rhesus macaques |
|  | Impulsivity and Stress Response in Pathological Gamblers During the Trier Social Stress Test |
|  | Salivary cortisol and α-amylase responses to repeated bouts of downhill running |
|  | Hair cortisol, stress exposure, and mental health in humans: a systematic review |
|  | Endocrine and immunomodulatory effects of social isolation and loneliness across adulthood |
|  | Associations between self-reported discrimination and diurnal cortisol rhythms among young adults: The moderating role of racial-ethnic minority status |
|  | GR gene BclI polymorphysm changes the path, but not the level, of dexamethasone-induced cortisol suppression |
|  | Covariation between behaviour and physiology indicators of coping style in zebrafish (Danio rerio) |
|  | He said what? Physiological and cognitive responses to imagining and witnessing outgroup racism |
|  | Mathematical modeling of light-mediated HPA axis activity and downstream implications on the entrainment of peripheral clock genes |
|  | Parental life events cause behavioral difference among offspring: Adult pre-gestational restraint stress reduces anxiety across generations |
|  | Hypothalamic-pituitary-adrenal axis hyperactivity is associated with decreased brain-derived neurotrophic factor in female suicide attempters |
|  | Human immune deficiency virus (HIV) infection and the hypothalamic pituitary adrenal axis |
|  | Cortisol awakening response is linked to disease course and progression in multiple sclerosis |
|  | Effort-reward imbalance at work and pre-clinical biological indices of ill-health: the case for salivary immunoglobulin A |
|  | Effects of hydrocortisone and yohimbine on selective attention to emotional cues |
|  | Cortisol response to acute stress in asthma: Moderation by depressive mood |
|  | Neuroendocrine Response Following a Thoracic Spinal Manipulation in Healthy Men |
|  | Cortisol Awakening Response as a Prospective Risk Factor for Depressive Symptoms in Women After Treatment for Breast Cancer |
|  | Mothers' parenting stress is associated with salivary cortisol profiles in children with attention deficit hyperactivity disorder |
|  | A variant on the kappa opioid receptor gene (OPRK1) is associated with stress response and related drug craving, limbic brain activation and cocaine relapse risk |
|  | Sheehan's syndrome presenting as cardiac tamponade |
|  | Associations of weight stigma with cortisol and oxidative stress independent of adiposity |
|  | Parental sensitivity mediates the sustained effect of Attachment and Biobehavioral Catch-up on cortisol in middle childhood: A randomized clinical trial |
|  | Mediators of compassionate goal intervention effects on human neuroendocrine responses to the Trier Social Stress Test |
|  | Vasotocinergic and isotocinergic systems in the gilthead sea bream (Sparus aurata): an osmoregulatory story |
|  | Elevated Hair Cortisol Levels among Heroin Addicts on Current Methadone Maintenance Compared to Controls |
|  | Extensive clinical experience: Hypothalamic-pituitary-adrenal axis recovery after adrenalectomy for corticotropin-independent cortisol excess |
|  | Oxytocin receptor gene methylation: converging multilevel evidence for a role in social anxiety |
|  | Growth hormone and cortisol secretion in the elderly evaluated using the glucagon stimulation test |
|  | Blunted HPA Axis Activity in Suicide Attempters Compared to those at High Risk for Suicidal Behavior |
|  | Cortisol awakening response and subsequent depression: prospective longitudinal study |
|  | The lifetime experience of traumatic events is associated with hair cortisol concentrations in community-based children |
|  | Coping with having a depressed mother: the role of stress and coping in hypothalamic-pituitary-adrenal axis dysfunction in girls at familial risk for major depression |
|  | Chronic stress induces structural alterations in splenic lymphoid tissue that are associated with changes in corticosterone levels in wistar-kyoto rats |
|  | Primary granulomatous hypophysitis: an interesting entity |
|  | Effects of cortisol administration on craving in heroin addicts |
|  | Intranasal insulin decreases circulating cortisol concentrations during early sleep in elderly humans |
|  | Differences in HPA-axis and heart rate responsiveness to psychosocial stress in children with autism spectrum disorders with and without co-morbid anxiety |
|  | The Val66Met brain-derived neurotrophic factor gene variant interacts with early pain exposure to predict cortisol dysregulation in 7-year-old children born very preterm: Implications for cognition |
|  | Reduced corticosteroid-binding globulin cleavage in active rheumatoid arthritis |
|  | The gonadal response to social stress and its relationship to cortisol |
|  | Child mortality, hypothalamic-pituitary-adrenal axis activity and cellular aging in mothers |
|  | Analysis of baseline hypothalamic-pituitary-adrenal activity in late adolescence reveals gender specific sensitivity of the stress axis |
|  | Stress following extinction learning leads to a context-dependent return of fear |
|  | Determinants of hair cortisol concentration in children: A systematic review |
|  | Mother-child adrenocortical synchrony; Moderation by dyadic relational behavior |
|  | Stress axis programming generates long-term effects on cognitive abilities in a cooperative breeder |
|  | Something to talk about: Gossip increases oxytocin levels in a near real-life situation |
|  | High cortisol responses identify propensity for obesity that is linked to thermogenesis in skeletal muscle |
|  | Advances in the assessment of cortisol exposure and sensitivity |
|  | Long-Term Effects of Prematurity, Cumulative Medical Risk, and Proximal and Distal Social Forces on Individual Differences in Diurnal Cortisol at Young Adulthood |
|  | Psychobiology of cumulative trauma: hair cortisol as a risk marker for stress exposure in women |
|  | Ten years of research with the Socially Evaluated Cold Pressor Test: Data from the past and guidelines for the future |
|  | Patterns of Adolescent Regulatory Responses During Family Conflict and Mental Health Trajectories |
|  | The role of the hypothalamic-pituitary-adrenal axis in modulating seasonal changes in immunity |
|  | Effects of early adversity on young children's diurnal cortisol rhythms and externalizing behavior |
|  | Hypothalamic-pituitary-adrenal axis activity and cognition in major depression: The role of remission status |
|  | Impact of chronic maternal stress during early gestation on maternal-fetal stress transfer and fetal stress sensitivity in sheep |
|  | Age-dependent effect of high cholesterol diets on anxiety-like behavior in elevated plus maze test in rats |
|  | Association between child cortisol levels in saliva and neuropsychological development during the second year of life |
|  | Effort-reward imbalance in police work: associations with the cortisol awakening response |
|  | Glucocorticoids Protect Neonatal Rat Brain in Model of Hypoxic-Ischemic Encephalopathy (HIE) |
|  | The Effects of a Korean Ginseng, GINST15, on Hypo-Pituitary-Adrenal and Oxidative Activity Induced by Intense Work Stress |
|  | Long-Term Cortisol Concentration in Scalp Hair of Asthmatic Children Using Inhaled Corticosteroids: A Case-Control Study |
|  | Adrenocortical function during prolonged critical illness and beyond: a prospective observational study |
|  | Increased hypothalamic-pituitary-adrenal drive is associated with decreased appetite and hypoactivation of food-motivation neurocircuitry in anorexia nervosa |
|  | Cortisol and induced cognitive fatigue: effects on memory activation in healthy males |
|  | A Randomized Controlled Study of Low-Dose Hydrocortisone Versus Placebo in Dopamine-Treated Hypotensive Neonates Undergoing Hypothermia Treatment for Hypoxic-Ischemic Encephalopathy |
|  | Changes in brain arginine vasotocin, isotocin, plasma 11-ketotestosterone and cortisol in round goby, Neogobius melanostomus, males subjected to overcrowding stress during the breeding season |
|  | An exploratory analysis of the joint contribution of HPA axis activation and motivation to early adolescent depressive symptoms |
|  | Differences in cardiovascular and hypothalamic-pituitary-adrenal axis functions between high-altitude visitors and natives during a trek on the Annapurna circuit |
|  | Atypical hypocortisolism |
|  | Requirement for age-specific peak cortisol responses to insulin-induced hypoglycaemia in children |
|  | Reduced post-synaptic serotonin type 1A receptor binding in bipolar depression |
|  | Towards an Integrated View of Early Molecular Changes Underlying Vulnerability to Social Stress in Psychosis |
|  | Concurrent and prospective associations between HPA axis activity and depression symptoms in newlywed women |
|  | Enduring effect of childhood maltreatment on cortisol and heart rate responses to stress: The moderating role of severity of experiences |
|  | Increased HPA Axis Activity and Serum Tryptophan in Naswar (Dipping Tobacco) Users: A Case-Control Study |
|  | Hypothalamic-pituitary-adrenal axis functioning and dysfunctional attitude in depressed patients with and without childhood neglect |
|  | The association between the hypothalamic pituitary adrenal axis and tryptophan metabolism in persons with recurrent major depressive disorder and healthy controls |
|  | Cortisol awakening response and cognitive performance in hypertensive and normotensive older people |
|  | Seasonal variations of basal cortisol and high stress response to captivity in Octodon degus, a mammalian model species |
|  | Stress reactivity in war-exposed young children with and without posttraumatic stress disorder: relations to maternal stress hormones, parenting, and child emotionality and regulation |
|  | Repetitive transcranial magnetic stimulation reduces cortisol concentrations in bulimic disorders |
|  | Effects of gender and age on hypothalamic-pituitary-adrenal reactivity after pharmacological challenge with low-dose 1-μg ACTH test: a prospective study in healthy adults |
|  | Adipocyte glucocorticoid receptors mediate fat-to-brain signaling |
|  | The Effect of Nicotine on HPA Axis Activity in Females is Modulated by the FKBP5 Genotype |
|  | Longitudinal examination of infant baseline and reactivity cortisol from ages 7 to 16 months |
|  | Natural predator and a human stimulus differently affect the behavior, cortisol and cerebral hemisphere activity of marmoset monkeys |
|  | Relationship of childhood adversity and neighborhood violence to a proinflammatory phenotype in emerging adult African American men: An epigenetic link |
|  | The cortisol awakening response--applications and implications for sleep medicine |
|  | Stress hormone release is a key component of the metabolic response to lipopolysaccharide: studies in hypopituitary and healthy subjects |
|  | Tissue-specific daily variation in the oxidative status of sturgeon (Acipenser naccarii) and rainbow trout (Oncorhynchus mykiss): a comparative study |
|  | Breakfast high in whey protein or carbohydrates improves coping with workload in healthy subjects |
|  | The effect of exercise intensity on brain derived neurotrophic factor and memory in adolescents |
|  | Emotional stress regulation: The role of relative frontal alpha asymmetry in shaping the stress response |
|  | Assessment of Brain Derived Neurotrophic Factor in hair to study stress responses: A pilot investigation |
|  | Exploring the multidimensional complex systems structure of the stress response and its relation to health and sleep outcomes |
|  | Youth offspring of mothers with posttraumatic stress disorder have altered stress reactivity in response to a laboratory stressor |
|  | Activity mediates conscientiousness' relationship to diurnal cortisol slope in a national sample |
|  | A Pilot Study of Stress System Activation in Children Enrolled in a Targeted Prevention Program: Implications for Personalization |
|  | Quantification of hair cortisol concentration in common marmosets (Callithrix jacchus) and tufted capuchins (Cebus apella) |
|  | Salivary cortisol in early psychosis: New findings and meta-analysis |
|  | Stress and working memory in children and adolescents: Insights from a multisystem approach |
|  | Social setting, social rank and HPA axis response in cynomolgus monkeys |
|  | Patient With Severe Hyponatremia Caused by Adrenal Insufficiency Due to Ectopic Posterior Pituitary Lobe and Miscommunication Between Hypothalamus and Pituitary: A Case Report |
|  | The antidepressant venlafaxine disrupts brain monoamine levels and neuroendocrine responses to stress in rainbow trout |
|  | Featured Article: Community Crime Exposure and Risk for Obesity in Preschool Children: Moderation by the Hypothalamic-Pituitary-Adrenal-Axis Response |
|  | The buffering effect of social support on hypothalamic-pituitary-adrenal axis function during pregnancy |
|  | A 24-Hour Study of the Hypothalamo-Pituitary Axes in Huntington's Disease |
|  | The effects of long-term stress on neural dynamics of working memory processing: An investigation using ERP |
|  | Post-stress rumination predicts HPA axis responses to repeated acute stress |
|  | Neurobehavioral effects of 1,2-propanediol in zebrafish (Danio rerio) |
|  | Social support attenuates the adverse consequences of social deprivation stress in domestic piglets |
|  | Introduction: circadian rhythm and its disruption: impact on reproductive function |
|  | Microaggressions and Diurnal Cortisol: Examining Within-Person Associations Among African-American and Latino Young Adults |
|  | Reduced Cortical Excitability, Neuroplasticity, and Salivary Cortisol in 11-13-Year-Old Children Born to Women with Gestational Diabetes Mellitus |
|  | Does sleep deprivation increase the vulnerability to acute psychosocial stress in young and older adults? |
|  | Clinical correlates of hypothalamic-pituitary-adrenal axis measures in individuals at risk for psychosis and with first-episode psychosis |
|  | Acute Stress and Anxiety in Medical Residents on the Emergency Department Duty |
|  | During stress, heart rate variability moderates the impact of childhood adversity in women with breast cancer |
|  | Psychotherapeutic treatment and HPA axis regulation in posttraumatic stress disorder: A systematic review and meta-analysis |
|  | Resting cortisol level, self-concept, and putative familial environment in adolescents at ultra high-risk for psychotic disorders |
|  | Biological and symptom changes in posttraumatic stress disorder treatment: a randomized clinical trial |
|  | Cortisol covariation within parents of young children: Moderation by relationship aggression |
|  | Endotoxemia-induced inflammation and the effect on the human brain |
|  | The effect of mild acute stress during memory consolidation on emotional recognition memory |
|  | Maternal precarity and HPA axis functioning shape infant gut microbiota and HPA axis development in humans |
|  | Help or punishment: acute stress moderates basal testosterone's association with prosocial behavior |
|  | Peripheral and prefrontal stress system markers and risk of relapse in alcoholism |
|  | Plasma cortisol and faecal cortisol metabolites concentrations in stereotypic and non-stereotypic horses: do stereotypic horses cope better with poor environmental conditions? |
|  | Facebook behaviors associated with diurnal cortisol in adolescents: Is befriending stressful? |
|  | Prenatal maternal psychopathology and stress and offspring HPA axis function at 6 years |
|  | Diurnal coupling between testosterone and cortisol from adolescence to older adulthood |
|  | Evidence for central hypercortisolism and elevated blood pressure in adolescent offspring of mothers with pre-eclampsia |
|  | Physical activity and hypothalamic-pituitary-adrenocortical axis function in adolescents |
|  | Abnormal circadian rhythm and cortisol excretion in autistic children: a clinical study |
|  | The impact of PTSD treatment on the cortisol awakening response |
|  | [Adrenocorticotropic-secreting pheochromocytoma] |
|  | Effect of thyroid hormone on cardiac function following orthotopic heart transplantation in piglets |
|  | Hair cortisol concentration in preschoolers with attention-deficit/hyperactivity symptoms-Roles of gender and family adversity |
|  | Longitudinal associations between diurnal cortisol slope and alcohol use across adolescence: a seven-year prospective study |
|  | Effects of Horticultural Therapy on Asian Older Adults: A Randomized Controlled Trial |
|  | PREDICTING RECOVERY OF THE HYPOTHALAMIC-PITUITARY-ADRENAL AXIS AFTER PROLONGED GLUCOCORTICOID USE |
|  | Brief Report: Low-Dose Hydrocortisone Has Acute Enhancing Effects on Verbal Learning in HIV-Infected Men |
|  | Rapid acclimation of the cortisol stress response in adult turquoise killifish Nothobranchius furzeri |
|  | Alterations of hair cortisol and dehydroepiandrosterone in mother-infant-dyads with maternal childhood maltreatment |
|  | Boosting recovery rather than buffering reactivity: Higher stress-induced oxytocin secretion is associated with increased cortisol reactivity and faster vagal recovery after acute psychosocial stress |
|  | Impact of maternal steroids during pregnancy |
|  | Ewes With Divergent Cortisol Responses to ACTH Exhibit Functional Differences in the Hypothalamo-Pituitary-Adrenal (HPA) Axis |
|  | The fear-factor stress test: an ethical, non-invasive laboratory method that produces consistent and sustained cortisol responding in men and women |
|  | Polymorphisms of genes related to the hypothalamic-pituitary-adrenal axis influence the cortisol awakening response as well as self-perceived stress |
|  | A blunted diurnal cortisol response in the lower educated does not explain educational differences in coronary heart disease: findings from the AGES-Reykjavik study |
|  | Coupling of the HPA and HPG axes in the context of early life adversity in incarcerated male adolescents |
|  | Physiological and subjective responses after psychosocial stress in Chinese hepatitis B patients |
|  | Causal effects of the early caregiving environment on development of stress response systems in children |
|  | A community-based study on the association between insomnia and hypothalamic-pituitary-adrenal axis: sex and pubertal influences |
|  | Association between DNA methylation of the KITLG gene and cortisol levels under stress: a replication study |
|  | Acute psychosocial stress weakens the sense of agency in healthy adults |
|  | Salivary alpha-amylase and cortisol responsiveness following electrical stimulation stress in patients with the generalized type of social anxiety disorder |
|  | Effects of basal and acute cortisol on cognitive flexibility in an emotional task switching paradigm in men |
|  | A review of associated controversies surrounding glucocorticoid use in veterinary emergency and critical care |
|  | Glucocorticoid receptor gene methylation moderates the association of childhood trauma and cortisol stress reactivity |
|  | Free and bound cortisol in plasma and saliva during ACTH challenge in dairy cows and horses |
|  | Childhood trauma and HPA axis functionality in offspring of bipolar parents |
|  | Stress and sex: does cortisol mediate sex change in fish? |
|  | The effect of sex and irritable bowel syndrome on HPA axis response and peripheral glucocorticoid receptor expression |
|  | Fetal heart rate variability responsiveness to maternal stress, non-invasively detected from maternal transabdominal ECG |
|  | Atypical depression and non-atypical depression: Is HPA axis function a biomarker? A systematic review |
|  | Testosterone-cortisol dissociation in children exposed to prenatal maternal stress, and relationship with aggression: Project Ice Storm |
|  | Ecologically salient stressors and supports and the coordination of cortisol and salivary alpha-amylase in mothers and infants |
|  | Early calibration of the HPA axis by maternal psychopathology |
|  | Context influences the interplay of endocrine axes across the day |
|  | Remission in Cushing's disease is predicted by cortisol burden and its withdrawal following pituitary surgery |
|  | How children's anxiety symptoms impact the functioning of the hypothalamus-pituitary-adrenal axis over time: A cross-lagged panel approach using hierarchical linear modeling |
|  | A Natural Mutation in Helix 5 of the Ligand Binding Domain of Glucocorticoid Receptor Enhances Receptor-Ligand Interaction |
|  | Pilot study of adrenal steroid hormones in hair as an indicator of chronic mental and physical stress |
|  | Gender specific effect of psychological stress and cortisol reactivity on adolescent risk taking |
|  | Chronic ethanol exposure increases the non-dominant glucocorticoid, corticosterone, in the near-term pregnant guinea pig |
|  | Impact of early life adversity on the stress biobehavioral response during nicotine withdrawal |
|  | The Role of Biomarkers in Research on Caregivers for Cancer Patients: A Scoping Review |
|  | Food restriction alters salivary cortisol and α-amylase responses to a simulated weightlifting competition without significant performance modification |
|  | Posttraumatic growth and diurnal cortisol slope among women with metastatic breast cancer |
|  | Link between children's hair cortisol and psychopathology or quality of life moderated by childhood adversity risk |
|  | Relationship between sympathoadrenal and pituitary-adrenal response during colorectal distention in the presence of corticotropin-releasing hormone in patients with irritable bowel syndrome and healthy controls |
|  | Comparison of high- and low-dose corticosteroid regimens for organ donor management |
|  | Critical Illness-Related Corticosteroid Insufficiency (CIRCI): A Narrative Review from a Multispecialty Task Force of the Society of Critical Care Medicine (SCCM) and the European Society of Intensive Care Medicine (ESICM) |
|  | Ex vivo glucocorticoid receptor-mediated IL-10 response predicts the course of depression severity |
|  | Thermal imprinting modifies adult stress and innate immune responsiveness in the teleost sea bream |
|  | Stress reactivity and its effects on subsequent food intake in depressed and healthy women with and without adverse childhood experiences |
|  | Physiological linkage in couples and its implications for individual and interpersonal functioning: A literature review |
|  | Aluminum exposure impacts brain plasticity and behavior in Atlantic salmon (Salmo salar) |
|  | Diurnal salivary cortisol patterns prior to pregnancy predict infant birth weight |
|  | Effect of maternal smoking on stress physiology in healthy neonates |
|  | Effects of aircraft noise exposure on saliva cortisol near airports in France |
|  | MDMA and heightened cortisol: a neurohormonal perspective on the pregnancy outcomes of mothers used 'Ecstasy' during pregnancy |
|  | Glucocorticoid programming of the fetal male hippocampal epigenome |
|  | Social buffering of the maternal and infant HPA axes: Mediation and moderation in the intergenerational transmission of adverse childhood experiences |
|  | Five-year follow-up of effects of neonatal intensive care and morphine infusion during mechanical ventilation on diurnal cortisol rhythm |
|  | Posttraumatic stress disorder, smoking, and cortisol in a community sample of pregnant women |
|  | Psychophysiological responses to pain identify reproducible human clusters |
|  | Blocking the mineralocorticoid receptor in humans prevents the stress-induced enhancement of centromedial amygdala connectivity with the dorsal striatum |
|  | Social buffering of cortisol release and tympanic temperature asymmetries during novelty and isolation stress in marmoset monkeys |
|  | Glucocorticoids impair oocyte developmental potential by triggering apoptosis of ovarian cells via activating the Fas system |
|  | The effect of storage conditions on salivary cortisol concentrations using an enzyme immunoassay |
|  | The effect of atomoxetine on random and directed exploration in humans |
|  | Baseline morning cortisol level as a predictor of pituitary-adrenal reserve: a comparison across three assays |
|  | Burnout Is Associated with Reduced Parasympathetic Activity and Reduced HPA Axis Responsiveness, Predominantly in Males |
|  | A review of rhythm and responsiveness of cortisol in individuals with autism spectrum disorders |
|  | Brief, pre-retrieval stress differentially influences long-term memory depending on sex and corticosteroid response |
|  | The central CLOCK system and the stress axis in health and disease |
|  | Stress and immune biomarkers interact with parenting behavior to shape anxiety symptoms in trauma-exposed youth |
|  | Neurophysiological symptoms and aspartame: What is the connection? |
|  | Chronic stress, hair cortisol and depression: A prospective and longitudinal study of medical internship |
|  | How We Experience Being Alone: Age Differences in Affective and Biological Correlates of Momentary Solitude |
|  | Role of shame and body esteem in cortisol stress responses |
|  | The secretion, synthesis, and metabolism of cortisol and its downstream genes in the H-P-I axis of rare minnows (Gobiocypris rarus) are disrupted by acute waterborne cadmium exposure |
|  | Dietary Pyridoxine Protects against Stress and Maintains Immunohaematological Status in Chanos chanos Exposed to Endosulfan |
|  | Cortisol and testosterone increase financial risk taking and may destabilize markets |
|  | Acute shift in glutamate concentrations following experimentally induced panic with cholecystokinin tetrapeptide--a 3T-MRS study in healthy subjects |
|  | Acute psychophysiological stress impairs human associative learning |
|  | Cortisol profiles differentiated in adolescents and young adult males with fragile X syndrome versus autism spectrum disorder |
|  | Evidence of a unique and common genetic etiology between the CAR and the remaining part of the diurnal cycle: A study of 14 year-old twins |
|  | Child abuse, disruptive behavior disorders, depression, and salivary cortisol levels among institutionalized and community-residing boys in Mongolia |
|  | The Treatment of Cushing's Disease |
|  | Activation in the hypothalamic-pituitary-adrenocortical axis and sympathetic nervous system in women with carpal tunnel syndrome |
|  | Fatty acid metabolism and its longitudinal relationship with the hypothalamic-pituitary-adrenal axis in major depression: Associations with prospective antidepressant response |
|  | Evaluating the stress response as a bioindicator of sub-lethal effects of crude oil exposure in wild house sparrows (Passer domesticus) |
|  | Hydrocortisone reduces the beneficial effects of toll-like receptor 2 deficiency on survival in a mouse model of polymicrobial sepsis |
|  | Hair analysis reveals subtle HPA axis suppression associated with use of local corticosteroids: The Lifelines cohort study |
|  | Relaxin-related gene expression differs between anadromous and stream-resident stickleback (Gasterosteus aculeatus) following seawater transfer |
|  | Endogenous cortisol predicts decreased loss aversion in young men |
|  | Effects of melatonin and green-wavelength LED light on the physiological stress and immunity of goldfish, Carassius auratus, exposed to high water temperature |
|  | Changes in cortisol awakening responses (CAR) in menopausal women through short-term marine healing retreat program with specific factors affecting each CAR index |
|  | Defining adrenal status with salivary cortisol by gold-standard insulin hypoglycemia |
|  | Predicting first onset of depression in young girls: Interaction of diurnal cortisol and negative life events |
|  | Potentiation of Otoprotective Effect of Hydrocortisone Immobilized on Povidone Nanoparticles under Conditions of Intravenous Injection |
|  | Qiviut cortisol reflects hypothalamic-pituitary-adrenal axis activity in muskoxen (Ovibos moschatus) |
|  | Corticotropin (ACTH)-reactive immunoglobulins in adolescents in relation to antisocial behavior and stress-induced cortisol response. The TRAILS study |
|  | The postprandial rise in plasma cortisol in men is mediated by macronutrient-specific stimulation of adrenal and extra-adrenal cortisol production |
|  | Size at birth, morning cortisol and cardiometabolic risk markers in healthy Indian children |
|  | Prospective evaluation of a week one overnight metyrapone test with subsequent dynamic assessments of hypothalamic-pituitary-adrenal axis function after pituitary surgery |
|  | Positive and negative social support and HPA-axis hyperactivity: Evidence from glucocorticoids in human hair |
|  | Associations between maternal psychological distress and salivary cortisol during pregnancy: A mixed-models approach |
|  | Evaluation of stress response using psychological, biological, and electrophysiological markers during immersive simulation of life threatening events in multidisciplinary teams |
|  | Quality of maternal and paternal care predicts later stress reactivity in the cooperatively-breeding marmoset (Callithrix geoffroyi) |
|  | Impact of psychological health on peripheral endothelial function and the HPA-axis activity in healthy adolescents |
|  | A longitudinal study of hair cortisol concentrations in Macaca nemestrina mothers and infants |
|  | Blunted HPA axis activity prior to suicide attempt and increased inflammation in attempters |
|  | Associations between hypothalamic-pituitary-adrenal axis function and peak bone mass at 20years of age in a birth cohort |
|  | Borderline and cluster C personality disorders manifest distinct physiological responses to psychosocial stress |
|  | Validated assay for the simultaneous determination of cortisol and budesonide in human plasma using ultra high performance liquid chromatography-tandem mass spectrometry |
|  | Effect of cortisol on gonadotropin inhibitory hormone (GnIH) in the cinnamon clownfish, Amphiprion melanopus |
|  | Cortisol increases in response to brief social exchanges with opposite sex partners |
|  | MECHANISMS IN ENDOCRINOLOGY: New concepts to further unravel adrenal insufficiency during critical illness |
|  | Cortisol mediates the effects of stress on the contextual dependency of memories |
|  | Corticotroph tumor progression during long-term therapy with osilodrostat in a patient with persistent Cushing's disease |
|  | HPA-axis reactivity interacts with stage of pubertal development to predict the onset of depression |
|  | Cushing's Syndrome in a 6-month-old Boy: A Rare Side-effect due to Inadequate use of Topical Corticosteroids |
|  | Stress vulnerability in male youth with Internet Gaming Disorder |
|  | Central adrenal insufficiency in children and adolescents |
|  | Wives' and husbands' cortisol reactivity to proximal and distal dimensions of couple conflict |
|  | Inhibitory avoidance learning in zebrafish (Danio rerio): effects of shock intensity and unraveling differences in task performance |
|  | HPA-Axis Activation as a Key Moderator of Childhood Trauma Exposure and Adolescent Mental Health |
|  | DHEA and DHEA-S levels in posttraumatic stress disorder: A meta-analytic review |
|  | Curvilinear associations between family income in early childhood and the cortisol awakening response in adolescence |
|  | Tell me what to do: Stress facilitates stimulus-response learning by instruction |
|  | The combined propranolol/TSST paradigm--a new method for psychoneuroendocrinology |
|  | Toward subtyping of suicidality: Brief suicidal ideation is associated with greater stress response |
|  | Chronic administration of ellagic acid improved the cognition in middle-aged overweight men |
|  | Stress inhibition of melatonin synthesis in the pineal organ of rainbow trout (Oncorhynchus mykiss) is mediated by cortisol |
|  | Dissociation of ACTH and cortisol in septic and non-septic ICU patients |
|  | Cortisol reactivity, delay discounting and percent body fat in Chinese urban young adolescents |
|  | The social ecology of childhood and early life adversity |
|  | Oxytocin modulates behavioral and physiological responses to a stressor in marmoset monkeys |
|  | The roles of puberty and age in explaining the diminished effectiveness of parental buffering of HPA reactivity and recovery in adolescence |
|  | Functioning of the hypothalamic-pituitary-adrenal and growth hormone axes in frequently unexplained disorders: results of a population study |
|  | Weight Stigma and Hypothalamic-Pituitary-Adrenocortical Axis Reactivity in Individuals Who Are Overweight |
|  | Dietary fatty acids sex-specifically modulate guinea pig postnatal development via cortisol concentrations |
|  | Behavioral responses to social separation stressor change across development and are dynamically related to HPA activity in marmosets |
|  | Characterization of the Hypothalamic-Pituitary-Adrenal-Axis in Familial Longevity under Resting Conditions |
|  | Impact of psychosocial stress on gonadotrophins and sexual behaviour in females: role for cortisol? |
|  | Differential associations between behavioral and cortisol responses to a stressor in securely versus insecurely attached infants |
|  | The effect of cortisol in rat steatotic and non-steatotic liver transplantation from brain-dead donors |
|  | Social stress modulates the cortisol response to an acute stressor in rainbow trout (Oncorhynchus mykiss) |
|  | Hair cortisol levels in posttraumatic stress disorder and metabolic syndrome |
|  | Stress responses to repeated exposure to a combined physical and social evaluative laboratory stressor in young healthy males |
|  | Late-night salivary cortisol may be valuable for assessing treatment response in patients with Cushing's disease: 12-month, Phase III pasireotide study |
|  | Testosterone during Puberty Shifts Emotional Control from Pulvinar to Anterior Prefrontal Cortex |
|  | Inflammation and inflammatory control in interstitial cystitis/bladder pain syndrome: Associations with painful symptoms |
|  | Adolescent oxytocin response to stress and its behavioral and endocrine correlates |
|  | The socially evaluated handgrip test: Introduction of a novel, time-efficient stress protocol |
|  | Association between cortisol awakening response and memory function in major depression |
|  | Developmental histories of perceived racial discrimination and diurnal cortisol profiles in adulthood: A 20-year prospective study |
|  | Hippocampal gene expression, serum cortisol level, and spatial memory in rats exposed to hypergravity |
|  | Role of Aspirin and Dexamethasone against Experimentally Induced Depression in Rats |
|  | Associations between temperament and gene polymorphisms in the brain dopaminergic system and the adrenal gland of sheep |
|  | Association of HPA axis hormones with copeptin after psychological stress differs by sex |
|  | A randomized controlled trial on the efficacy of resistant dextrin, as functional food, in women with type 2 diabetes: Targeting the hypothalamic-pituitary-adrenal axis and immune system |
|  | The effectiveness of acupuncture on HPA functional in depressed patients under methadone maintenance treatment, a randomized double-blind sham-controlled trial |
|  | Cortisol and ACTH levels in drug-naive adolescents with first-episode early onset schizophrenia |
|  | Hormonal and Neuromuscular Responses to Breastfeeding: A Pilot Study |
|  | Social Transitions Cause Rapid Behavioral and Neuroendocrine Changes |
|  | What worries parents of a child with Autism? Evidence from a biomarker for chronic stress |
|  | Ambient PM(2.5) exposure and salivary cortisol output during pregnancy in a multi-ethnic urban sample |
|  | Neural plasticity and stress coping in teleost fishes |
|  | Diurnal Hypothalamic-Pituitary-Adrenal Axis Measures and Inflammatory Marker Correlates in Major Depressive Disorder |
|  | Chronic stress in the mother-infant dyad: Maternal hair cortisol, infant salivary cortisol and interactional synchrony |
|  | Stress and genetics influence hair cortisol in FMR1 premutation carrier mothers of children with fragile X syndrome |
|  | HPA axis and aging in depression: systematic review and meta-analysis |
|  | Salivary cortisol and cold pain sensitivity in female twins |
|  | Catechol-O-Methyltransferase gene (val158met) polymorphisms and anxious symptoms in early childhood: The roles of hypothalamus-pituitary-adrenal axis reactivity and life stress |
|  | Oxytocin, cortisol and 3,4-methylenedioxymethamphetamine: neurohormonal aspects of recreational 'ecstasy' |
|  | Diurnal cortisol and survival in epithelial ovarian cancer |
|  | Hair cortisol concentration (HCC) as a measure for prenatal psychological distress - A systematic review |
|  | Hydrocortisone infusion exerts dose- and sex-dependent effects on attention to emotional stimuli |
|  | Hypothalamic-pituitary-adrenal axis suppression by inhaled or nasal corticosteroids in HIV-infected patients |
|  | Glucocorticoid receptor density and binding affinity in healthy horses and horses with systemic inflammatory response syndrome |
|  | Chronic stress induces a hyporeactivity of the autonomic nervous system in response to acute mental stressor and impairs cognitive performance in business executives |
|  | High-throughput profiling of the circulating proteome suggests sexually dimorphic corticosteroid signaling following ischemic stroke |
|  | Cold-water immersion decreases cerebral oxygenation but improves recovery after intermittent-sprint exercise in the heat |
|  | Diurnal salivary cortisol, glycemia and insulin resistance: The multi-ethnic study of atherosclerosis |
|  | Hippocampal glucocorticoid receptor expression in the tree shrew: regulation by psychosocial conflict |
|  | Promoter haplotypes of the corticotropin-releasing hormone encoding gene modulate the physiological stress response in vitro and in vivo |
|  | Safety of long-term high-volume sinonasal budesonide irrigations for chronic rhinosinusitis |
|  | Autonomic nervous system and hypothalamic-pituitary-adrenal axis response to experimentally induced cold pain in adolescent non-suicidal self-injury--study protocol |
|  | Impact of physical fitness on salivary stress markers in sedentary to low-active young to middle-aged men |
|  | Immune dysregulation and glucocorticoid resistance in minority and low income pregnant women |
|  | Cortisol stress response in post-traumatic stress disorder, panic disorder, and major depressive disorder patients |
|  | Hormonal underpinnings of status conflict: Testosterone and cortisol are related to decisions and satisfaction in the hawk-dove game |
|  | Hair cortisol levels, psychological stress and psychopathological symptoms as predictors of postpartum depression |
|  | Acclimation of zebrafish to transport stress |
|  | Hypothalamic-pituitary-adrenal axis activity and vascular function in healthy adults |
|  | Long-term alteration of the hypothalamic-pituitary-adrenal axis in children undergoing cardiac surgery in the first 6 months of life |
|  | Subcutaneous pulsatile glucocorticoid replacement therapy |
|  | Genetics of neuroendocrine factors in rheumatoid arthritis |
|  | Inducing physiological stress recovery with sounds of nature in a virtual reality forest--results from a pilot study |
|  | A Deletion Variant of the α2b-Adrenoceptor Modulates the Stress-Induced Shift from "Cognitive" to "Habit" Memory |
|  | Ferredoxin 1b (Fdx1b) Is the Essential Mitochondrial Redox Partner for Cortisol Biosynthesis in Zebrafish |
|  | Psoriasis severity and hypothalamic-pituitary-adrenal axis function: results from the CALIPSO study |
|  | Hair cortisol concentration and glycated hemoglobin in African American adults |
|  | Can hair steroids predict pregnancy longevity? |
|  | Drug-induced HPA axis alterations during acute critical illness: a multivariable association study |
|  | Effects of naproxen on immune responses in a colchicine-induced rat model of Alzheimer's disease |
|  | Characterization of membrane receptor binding activity for cortisol in the liver and kidney of the euryhaline teleost, Mozambique tilapia (Oreochromis mossambicus) |
|  | The interaction of BDNF Val66Met, PTSD, and child abuse on psychophysiological reactivity and HPA axis function in a sample of Gulf War Veterans |
|  | Exposure to high fat during early development impairs adaptations in dopamine and neuroendocrine responses to repeated stress |
|  | Measures of adiposity predict interleukin-6 responses to repeated psychosocial stress |
|  | Glucocorticoid receptor exon 1(F) methylation and the cortisol stress response in health and disease |
|  | Cranial electrotherapy stimulation affects mood state but not levels of peripheral neurotrophic factors or hypothalamic- pituitary-adrenal axis regulation |
|  | Matrilineal Behavioral and Physiological Changes following the Death of a Non-Alpha Matriarch in Rhesus Macaques (Macaca mulatta) |
|  | Socioeconomic status in children is associated with hair cortisol levels as a biological measure of chronic stress |
|  | Copeptin as a marker of an altered CRH axis in pituitary disease |
|  | Hydrocortisone-induced parkin prevents dopaminergic cell death via CREB pathway in Parkinson's disease model |
|  | Underweight subjects with anorexia nervosa have an enhanced salivary cortisol response not seen in weight restored subjects with anorexia nervosa |
|  | Cortisol awakening response in PTSD treatment: Predictor or mechanism of change |
|  | Hidden hypercortisolism: a too frequently neglected clinical condition |
|  | Effects of acute stress provocation on cortisol levels, zonulin and inflammatory markers in low- and high-stressed men |
|  | Examining stress: an investigation of stress, mood and exercise in medical students |
|  | Evaluating tertiary adrenal insufficiency in rheumatology patients on long-term systemic glucocorticoid treatment |
|  | Commentary: Connecting cytokines to distress via cortisol concentrations |
|  | Physiologic and cortical response to acute psychosocial stress in left temporal lobe epilepsy - a pilot cross-sectional fMRI study |
|  | Salivary cortisol and cortisone responses to short-term psychological stress challenge in late adolescent and young women with different hyperandrogenic states |
|  | Salivary cortisol is associated with cognitive changes in patients with fibromyalgia |
|  | Lead exposure is related to hypercortisolemic profiles and allostatic load in Brazilian older adults |
|  | Social evaluative threat with verbal performance feedback alters neuroendocrine response to stress |
|  | Salivary nerve growth factor reactivity to acute psychosocial stress: a new frontier for stress research |
|  | Prednisolone markedly reduced serum IgG4 levels along with the improvement of pituitary mass and anterior pituitary function in a patient with IgG4-related infundibulo-hypophysitis |
|  | Diurnal Cortisol Concentrations and Growth Indexes of 12- to 48-Month-Old Children From Mexico City |
|  | Disrupted-in-Schizophrenia-1 is essential for normal hypothalamic-pituitary-interrenal (HPI) axis function |
|  | Social correlates of the dominance rank and long-term cortisol levels in adolescent and adult male rhesus macaques (Macaca mulatta) |
|  | Gonadotropin-releasing hormone agonist in premenopausal women does not alter hypothalamic-pituitary-adrenal axis response to corticotropin-releasing hormone |
|  | Infant adrenocortical reactivity and behavioral functioning: relation to early exposure to maternal intimate partner violence |
|  | Cell number and neuropil alterations in subregions of the anterior hippocampus in a female monkey model of depression |
|  | Association between hair cortisol, hair cortisone, and fatigue in people living with HIV |
|  | Individual differences in early adolescents' latent trait cortisol: Interaction of early adversity and 5-HTTLPR |
|  | Psychopathy's influence on the coupling between hypothalamic-pituitary-adrenal and -gonadal axes among incarcerated adolescents |
|  | Childhood family adversity and adult cortisol response: The role of observed marital conflict behavior |
|  | Salivary alpha-amylase and cortisol responsiveness to stress in first episode, drug-naïve patients with panic disorder |
|  | Update on adrenal insufficiency in patients with liver cirrhosis |
|  | Stress-induced reliance on habitual behavior is moderated by cortisol reactivity |
|  | Age-related differences in stress responsiveness of the hypothalamic-pituitary-adrenal axis of nonhuman primates with various types of adaptive behavior |
|  | Effects of freely accessible computerized test systems on the spontaneous behaviors and stress level of Guinea baboons (Papio papio) |
|  | Special features of neuroendocrine interactions between stress and reproduction in teleosts |
|  | Cushing's disease: the burden of illness |
|  | An integrated mechanism of pediatric pseudotumor cerebri syndrome: evidence of bioenergetic and hormonal regulation of cerebrospinal fluid dynamics |
|  | Maternal variables associated with physiologic stress and perinatal complications in preterm infants |
|  | Corticotropin-releasing hormone system polymorphisms are associated with children's cortisol reactivity |
|  | Role of the dorsomedial hypothalamus in glucocorticoid-mediated feedback inhibition of the hypothalamic-pituitary-adrenal axis |
|  | Glucocorticoid receptor positively regulates transcription of FNDC5 in the liver |
|  | Toxic stress history and hypothalamic-pituitary-adrenal axis function in a social stress task: Genetic and epigenetic factors |
|  | A decline in female baboon hypothalamo-pituitary-adrenal axis activity anticipates aging |
|  | Learning to resist the urge: a double-blind, randomized controlled trial investigating alcohol-specific inhibition training in abstinent patients with alcohol use disorder |
|  | Dominant men are faster in decision-making situations and exhibit a distinct neural signal for promptness |
|  | Sleep duration moderates the association between insula activation and risky decisions under stress in adolescents and adults |
|  | Maternal obesity programs reduced leptin signaling in the pituitary and altered GH/IGF1 axis function leading to increased adiposity in adult sheep offspring |
|  | Early social experience has life-long effects on baseline but not stress-induced cortisol levels in a cooperatively breeding fish |
|  | HPA axis in psychotic major depression and schizophrenia spectrum disorders: Cortisol, clinical symptomatology, and cognition |
|  | Hypothalamus-pituitary-adrenal axis function in patients with rheumatoid arthritis treated with nighttime-release prednisone |
|  | Stress-induced decrease of uterine blood flow in sheep is mediated by alpha 1-adrenergic receptors |
|  | DHEAS and cortisol/DHEAS-ratio in recurrent depression: State, or trait predicting 10-year recurrence? |
|  | Psychosocial stress sensitizes neuroendocrine and inflammatory responses to Escherichia coli challenge in domestic piglets |
|  | Stress-system genes and life stress predict cortisol levels and amygdala and hippocampal volumes in children |
|  | Increased symptoms of anxiety and depression in prepubertal girls, but not boys, with premature adrenarche: associations with serum DHEAS and daily salivary cortisol concentrations |
|  | Activation of the hypothalamic-pituitary-adrenal axis in adults with mineralocorticoid receptor haploinsufficiency |
|  | Stress enhances reconsolidation of declarative memory |
|  | Selective impact of early parental responsivity on adolescent stress reactivity |
|  | Suppressing the endocrine and autonomic stress systems does not impact the emotional stress experience after psychosocial stress |
|  | A Comparison of Salivary Steroid Levels during Diagnostic Tests for Adrenal Insufficiency |
|  | Limitations in the inverse association between psychological resilience and depression in prostate cancer patients experiencing chronic physiological stress |
|  | [A case of stiff-person syndrome due to secondary adrenal insufficiency] |
|  | Benefit finding and diurnal cortisol after prostate cancer: The mediating role of positive affect |
|  | Effects of short-term varenicline administration on cortisol in healthy, non-smoking adults: a randomized, double-blind, study |
|  | Clinical application of DEX/CRH test and multi-channel NIRS in patients with depression |
|  | Dynamics of salivary cortisol in chronic kidney disease patients at stages 1 through 4 |
|  | Victimization and Biological Stress Responses in Urban Adolescents: Emotion Regulation as a Moderator |
|  | Adolescent alcohol exposure alters the rat adult hypothalamic-pituitary-adrenal axis responsiveness in a sex-specific manner |
|  | Biomarkers of stress in behavioural medicine |
|  | Delayed effects of cortisol enhance fear memory of trace conditioning |
|  | Depressogenic vulnerability and gender-specific patterns of neuro-immune dysregulation: What the ratio of cortisol to C-reactive protein can tell us about loss of normal regulatory control |
|  | Error consciousness predicts physiological response to an acute psychosocial stressor in men |
|  | Total and free cortisol levels during 1 μg, 25 μg, and 250 μg cosyntropin stimulation tests compared to insulin tolerance test: results of a randomized, prospective, pilot study |
|  | Dynamics of electrocardiographic changes, brain-natriuretic peptide and cortisol levels in a patient with stress (takotsubo) cardiomyopathy--a case report |
|  | Examining multiple sleep behaviors and diurnal salivary cortisol and alpha-amylase: Within- and between-person associations |
|  | How should we interrogate the hypothalamic-pituitary-adrenal axis in patients with suspected hypopituitarism? |
|  | Maternal experiences of trauma and hair cortisol in early childhood in a prospective cohort |
|  | Childhood trauma dependent anxious depression sensitizes HPA axis function |
|  | Exposure of Porphyromonas gingivalis to cortisol increases bacterial growth |
|  | Hypothalamic-Pituitary-Adrenocortical Dysfunction in Elderly, Male Marathon Runners: Feedback Sensitivity, Stress Response, and Effects on Verbal Memory |
|  | DNA methylation differences at the glucocorticoid receptor gene in depression are related to functional alterations in hypothalamic-pituitary-adrenal axis activity and to early life emotional abuse |
|  | Effects of prenatal stress on behavioural and neurodevelopmental outcomes are altered by maternal separation in the neonatal period |
|  | Between Temperament and Psychopathology: Examples from Neuropharmacological Challenge Tests in Healthy Humans |
|  | Prenatal and postnatal inflammation in relation to cortisol levels in preterm infants at 18 months corrected age |
|  | Cortisol-dependent stress effects on cell distribution in healthy individuals and individuals suffering from chronic adrenal insufficiency |
|  | Salivary cortisol response to infant distress in pregnant women with depressive symptoms |
|  | [Opioid Induced Pituitary Dysfunction] |
|  | Associations between dehydroepiandrosterone (DHEA) levels, pituitary volume, and social anxiety in children |
|  | Examining HPA-axis functioning as a mediator of the relationship between depression and cognition across the adult lifespan |
|  | Effects of interpersonal violence-related post-traumatic stress disorder (PTSD) on mother and child diurnal cortisol rhythm and cortisol reactivity to a laboratory stressor involving separation |
|  | Receptors rather than signals change in expression in four physiological regulatory networks during evolutionary divergence in threespine stickleback |
|  | Social stress buffering by friends in childhood and adolescence: Effects on HPA and oxytocin activity |
|  | The Modulating Role of Stress in the Onset and Course of Tourette's Syndrome: A Review |
|  | Caffeine alters emotion and emotional responses in low habitual caffeine consumers |
|  | Beyond the social stereotypes of hormones |
|  | Biologic effects of stress and bonding in mother-infant pairs |
|  | Intimacy as Related to Cortisol Reactivity and Recovery in Couples Undergoing Psychosocial Stress |
|  | Cardio-metabolic consequences of glucocorticoid replacement: relevance of ultradian signalling |
|  | Hypothalamic-pituitary-adrenal axis responses of horses to therapeutic riding program: effects of different riders |
|  | Daily regulation of hormone profiles |
|  | The Short Synacthen (Corticotropin) Test Can Be Used to Predict Recovery of Hypothalamo-Pituitary-Adrenal Axis Function |
|  | Co-expression of c-Fos with oestradiol receptor α or somatostatin in the arcuate nucleus, ventromedial nucleus and medial preoptic area in the follicular phase of intact ewes: alteration after insulin-induced hypoglycaemia |
|  | The correlation between perceived social support, cortisol and brain derived neurotrophic factor levels in healthy women |
|  | Comparison of clear and narrow outcomes on testosterone levels in social competition |
|  | Is stress affecting our ability to tune into others? Evidence for gender differences in the effects of stress on self-other distinction |
|  | Stress-Dose Corticosteroid Versus Placebo in Neonatal Cardiac Operations: A Randomized Controlled Trial |
|  | Blunted HPA axis response to stress is related to a persistent Dysregulation Profile in youth |
|  | Hair Cortisol in Twins: Heritability and Genetic Overlap with Psychological Variables and Stress-System Genes |
|  | Adrenal hormones and circulating leukocyte subtypes in stroke patients treated with reperfusion therapy |
|  | Mindful parenting predicts mothers' and infants' hypothalamic-pituitary-adrenal activity during a dyadic stressor |
|  | Adrenal insufficiency in patients on long-term opioid analgesia |
|  | C-reactive protein, pre- and postdexamethasone cortisol levels in post-traumatic stress disorder |
|  | Posterior reversible encephalopathy syndrome due to ectopic Cushing's syndrome: an uncommon presentation of a rare disease |
|  | Effects of acute handling stress on short-term central expression of orexigenic/anorexigenic genes in zebrafish |
|  | Coping with stress before and after mild traumatic brain injury: a pilot hair cortisol study |
|  | Performance of salivary cortisol in the diagnosis of Cushing's syndrome, adrenal incidentaloma, and adrenal insufficiency |
|  | Objective Evaluation of Performance Stress in Musicians With Focal Hand Dystonia: A Case Series |
|  | Hair cortisol concentrations in a Spanish sample of healthy adults |
|  | Reduced glucocorticoid receptor protein expression in children with critical illness |
|  | Avocado oil (Persea americana) protects SH-SY5Y cells against cytotoxicity triggered by cortisol by the modulation of BDNF, oxidative stress, and apoptosis molecules |
|  | Prolonged hypothalamic-pituitary-adrenal axis activation after acute coronary syndrome in the GENESIS-PRAXY cohort |
|  | Adverse effects of two nights of sleep restriction on the hypothalamic-pituitary-adrenal axis in healthy men |
|  | Serotonin directly stimulates cortisol secretion from the interrenals in goldfish |
|  | Acute Elevations in Cortisol Increase the In Vivo Binding of [(11)C]NOP-1A to Nociceptin Receptors: A Novel Imaging Paradigm to Study the Interaction Between Stress- and Antistress-Regulating Neuropeptides |
|  | Influence of stress systems and physical activity on different dimensions of fatigue in female fibromyalgia patients |
|  | Early adversity, hypocortisolism, and behavior problems at school entry: A study of internationally adopted children |
|  | Icariin inhibits inflammation via immunomodulation of the cutaneous hypothalamus-pituitary-adrenal axis in vitro |
|  | Global stress response during a social stress test: impact of alexithymia and its subfactors |
|  | Basal functioning of the hypothalamic-pituitary-adrenal (HPA) axis and psychological distress in recreational ecstasy polydrug users |
|  | Effects of mindfulness training on levels of cortisol in cancer patients |
|  | Hypothalamic dopaminergic stimulation in cluster headache |
|  | Mice selected for extremes in stress reactivity reveal key endophenotypes of major depression: a translational approach |
|  | Health-related quality of life of patients with hypothalamic-pituitary-adrenal axis dysregulations: a cohort study |
|  | Peripheral blood mononuclear cell proliferation and cytokine production in sheep as affected by cortisol level and duration of stress |
|  | Angiotensin-converting enzyme gene variants are associated with both cortisol secretion and late-life depression |
|  | The effect of a primary sexual reward manipulation on cortisol responses to psychosocial stress in men |
|  | Children's fingernail cortisol among BaYaka foragers of the Congo Basin: associations with fathers' roles |
|  | Cortisol increase in empathic stress is modulated by emotional closeness and observation modality |
|  | Cortisol/DHEA ratio and hippocampal volume: A pilot study in major depression and healthy controls |
|  | [Contributions of cortisol suppression tests to understanding of psychiatric disorders: a narrative review of literature] |
|  | The attachment system and physiology in adulthood: normative processes, individual differences, and implications for health |
|  | High cortisol response to adrenocorticotrophic hormone identifies ewes with reduced melanocortin signalling and increased propensity to obesity |
|  | The interface of hypothalamic-pituitary-adrenocortical axis and circulating brain natriuretic peptide in prediction of cardiopulmonary performance during physical stress |
|  | Fructose-induced inflammation and increased cortisol: A new mechanism for how sugar induces visceral adiposity |
|  | Nebulized dexamethasone sodium phosphate in the treatment of horses with severe asthma |
|  | Communication and social interaction anxiety enhance interleukin-1 beta and cortisol reactivity during high-stakes public speaking |
|  | Clinical, hormonal and radiological features of partial Sheehan's syndrome: an Indian experience |
|  | Human milk cortisol is associated with infant temperament |
|  | Genetics of glucocorticoid regulation and posttraumatic stress disorder--What do we know? |
|  | Basal and stress-activated hypothalamic pituitary adrenal axis function in postmenopausal women with overactive bladder |
|  | Interactive influence of sex, stressor timing, and the BclI glucocorticoid receptor polymorphism on stress-induced alterations of long-term memory |
|  | Different regulation of cortisol and corticosterone in the subterranean rodent Ctenomys talarum: Responses to dexamethasone, angiotensin II, potassium, and diet |
|  | Hyperactivity of the Sympatho-Adrenomedullary System Without Any Modification of the Hypothalamic-Pituitary-Adrenal Axis After Food Restriction Among High-Level Weightlifters |
|  | Cortisol and α-Amylase Secretion Patterns between and within Depressed and Non-Depressed Individuals |
|  | Antenatal depression in a multi-ethnic, community sample of Canadian immigrants: psychosocial correlates and hypothalamic-pituitary-adrenal axis function |
|  | Locally elevated cortisol in lymphoid organs of the developing zebra finch but not Japanese quail or chicken |
|  | Salivary cortisol and alpha-amylase diurnal profiles and stress reactivity in children with Attention Deficit Hyperactivity Disorder |
|  | Coordination of cortisol response to social evaluative threat with autonomic and inflammatory responses is moderated by stress appraisals and affect |
|  | Sleep and hormonal changes in aging |
|  | The influence of foetal prednisone exposure on the cortisol levels in the offspring |
|  | Effects of dietary nucleotides on acute stress response and cannabinoid receptor 1 mRNAs in sole, Solea solea |
|  | Sex and the housing: Effects on behavior, cortisol levels and weight in zebrafish |
|  | Obstructive sleep apnoea syndrome is associated with relative hypocortisolemia and decreased hypothalamo-pituitary-adrenal axis response to 1 and 250μg ACTH and glucagon stimulation tests |
|  | Identification of a miRNAs signature associated with exposure to stress early in life and enhanced vulnerability for schizophrenia: New insights for the key role of miR-125b-1-3p in neurodevelopmental processes |
|  | Effects of environmental enrichment on cognitive performance of pigs in a spatial holeboard discrimination task |
|  | Effects of psychosocial stress on the goal-directed and habit memory systems during learning and later execution |
|  | MDMA, cortisol, and heightened stress in recreational ecstasy users |
|  | Combined use of spatial restraint stress and middle cerebral artery occlusion is a novel model of post-stroke depression in mice |
|  | The cortisol awakening response and cognition across the adult lifespan |
|  | A comparative study of the response to repeated chasing stress in Atlantic salmon (Salmo salar L.) parr and post-smolts |
|  | Differences in HPA axis reactivity to intimacy in women with and without histories of sexual trauma |
|  | The associations between adolescent sleep, diurnal cortisol patterns and cortisol reactivity to dexamethasone suppression test |
|  | Classification criteria for distinguishing cortisol responders from nonresponders to psychosocial stress: evaluation of salivary cortisol pulse detection in panel designs |
|  | Up-regulation of the fetal baboon hypothalamo-pituitary-adrenal axis in intrauterine growth restriction: coincidence with hypothalamic glucocorticoid receptor insensitivity and leptin receptor down-regulation |
|  | Subclinical Cushing's syndrome: current concepts and trends |
|  | Stressing over anxiety: A novel interaction of 5-HTTPLR genotype and anxiety-related phenotypes in older adults |
|  | Sustained Effects of Developmental Exposure to Ethanol on Zebrafish Anxiety-Like Behaviour |
|  | Acute effects of heroin on negative emotional processing: relation of amygdala activity and stress-related responses |
|  | Diurnal salivary cortisol measurement in the neurosurgical-surgical intensive care unit in critically ill acute trauma patients |
|  | Microcystin-LR affects the hypothalamic-pituitary-inter-renal (HPI) axis in early life stages (embryos and larvae) of zebrafish |
|  | Cortisol levels in hair are altered in irritable bowel syndrome - A case control study in primary care |
|  | Different stressors induce differential responses of the CRH-stress system in the gilthead sea bream (Sparus aurata) |
|  | A Systematic Review of Mechanisms of Change in Body-Oriented Yoga in Major Depressive Disorders |
|  | Programming of the hypothalamic-pituitary-interrenal axis by maternal social status in zebrafish (Danio rerio) |
|  | Impact of a purported nootropic supplementation on measures of mood, stress, and marksmanship performance in U.S. active duty soldiers |
|  | Polygonatum sibiricum polysaccharide prevents depression-like behaviors by reducing oxidative stress, inflammation, and cellular and synaptic damage |
|  | Chronic grouped social restriction triggers long-lasting immune system adaptations |
|  | Characterization and novel analyses of acute stress response patterns in a population-based cohort of young adults: influence of gender, smoking, and BMI |
|  | Longitudinal patterns of cortisol regulation differ in maltreated and nonmaltreated children |
|  | Associations between cognitive performance and cortisol reaction to the DEX/CRH test in patients recovered from depression |
|  | Cholestasis Reveals Severe Cortisol Deficiency in Neonatal Pituitary Stalk Interruption Syndrome |
|  | Prenatal and Postpartum Evening Salivary Cortisol Levels in Association with Peripartum Depressive Symptoms |
|  | Carbenoxolone Disodium Treatment for Canine Pituitary-Dependent Hyperadrenocorticism |
|  | Effects of chronic treatment with methylphenidate on oxidative stress and inflammation in hippocampus of adult rats |
|  | Stress and glucocorticoid receptor transcriptional programming in time and space: Implications for the brain-gut axis |
|  | Intrauterine Wachstumsretardierung als lebenslanges Risiko für das Kind |
|  | Association of Vitamin C, Thiamine, and Hydrocortisone Infusion With Long-term Cognitive, Psychological, and Functional Outcomes in Sepsis Survivors: A Secondary Analysis of the Vitamin C, Thiamine, and Steroids in Sepsis Randomized Clinical Trial |
|  | Time-dependent effects of dexamethasone plasma concentrations on glucocorticoid receptor challenge tests |
|  | HPA-axis hormone modulation of stress response circuitry activity in women with remitted major depression |
|  | Cortisol awakening and stress response, personality and psychiatric profiles in patients with takotsubo cardiomyopathy |
|  | Effects of antipsychotics on cortisol, interleukin-6 and hippocampal perfusion in healthy volunteers |
|  | DNA methylation and genetic variation of the angiotensin converting enzyme (ACE) in depression |
|  | Coordinated Action of Corticotropin-Releasing Hormone and Cortisol Shapes the Acute Stress-Induced Behavioural Response in Zebrafish |
|  | Evidence for disruption of normal circadian cortisol rhythm in women with obesity |
|  | Pituitary-adrenal function in patients with acute subarachnoid haemorrhage: a prospective cohort study |
|  | Modulatory effects of aromatherapy massage intervention on electroencephalogram, psychological assessments, salivary cortisol and plasma brain-derived neurotrophic factor |
|  | Human Placenta Buffers the Fetus from Adverse Effects of Perceived Maternal Stress |
|  | Impact on cortisol and antidepressant efficacy of quetiapine and escitalopram in depression |
|  | Sex-specific interaction between cortisol and striato-limbic responses to psychosocial stress |
|  | The effects of intranasal oxytocin on smoothie intake, cortisol and attentional bias in anorexia nervosa |
|  | Developmental origins of infant stress reactivity profiles: A multi-system approach |
|  | Physiological Effects of Nucleotide Supplementation on Resistance Exercise Stress in Men and Women |
|  | Acute glucocorticoid deficiency and diabetes insipidus are common after acute traumatic brain injury and predict mortality |
|  | Mother-child language style matching predicts children's and mothers' emotion reactivity |
|  | Salivary neuropeptides, stress, and periodontitis |
|  | Hair cortisol in the evaluation of Cushing syndrome |
|  | Stress-induced pro- and anti-inflammatory cytokine concentrations in panic disorder patients |
|  | IL-6 and TNF-α in unmedicated adults with ADHD: Relationship to cortisol awakening response |
|  | Dose timing of D-cycloserine to augment cognitive behavioral therapy for social anxiety: Study design and rationale |
|  | Sex determines cortisol and alpha-amylase responses to acute physical and psychosocial stress in patients with avoidant personality disorder |
|  | Hippocampal and cerebellar histological changes and their behavioural repercussions caused by brain ischaemic hypoxia experimentally induced by sodium nitrite |
|  | Neurochemical Effects of 4-(2Chloro-4-Fluorobenzyl)-3-(2-Thienyl)-1,2,4-Oxadiazol-5(4H)-One in the Pentylenetetrazole (PTZ)-Induced Epileptic Seizure Zebrafish Model |
|  | Oral corticosteroids for asthma exacerbations might be associated with adrenal suppression: Are physicians aware of that? |
|  | Restoring the salivary cortisol awakening response through nasal continuous positive airway pressure therapy in obstructive sleep apnea |
|  | Stress attenuates the flexible updating of aversive value |
|  | Cortisol modulates men's affiliative responses to acute social stress |
|  | The impact of an exercise training intervention on cortisol levels and post-traumatic stress disorder in juveniles from an Ugandan refugee settlement: study protocol for a randomized control trial |
|  | HPA-axis and inflammatory reactivity to acute stress is related with basal HPA-axis activity |
|  | Age-Adapted Stress Task in Preschoolers Does not Lead to Uniform Stress Responses |
|  | Advancing the science of organ donor management |
|  | The social transmission of risk: Maternal stress physiology, synchronous parenting, and well-being mediate the effects of war exposure on child psychopathology |
|  | Corticotropin-releasing hormone improves survival in pneumococcal pneumonia by reducing pulmonary inflammation |
|  | Saliva oxytocin, cortisol, and testosterone levels in adolescent boys with autism spectrum disorder, oppositional defiant disorder/conduct disorder and typically developing individuals |
|  | Delayed peak response of cortisol to insulin tolerance test in patients with Prader-Willi syndrome |
|  | Maternal stress and effects of prenatal air pollution on offspring mental health outcomes in mice |
|  | Prolonged secretion of cortisol as a possible mechanism underlying stress and depressive behaviour |
|  | Seasonal changes in gene expression of corticoid receptors in anadromous and non-anadromous strains of rainbow trout Oncorhynchus mykiss |
|  | Bright light treatment in elderly patients with nonseasonal major depressive disorder: a randomized placebo-controlled trial |
|  | Treadmill walking during vocabulary encoding improves verbal long-term memory |
|  | Genetic variants in serotonin and corticosteroid systems modulate neuroendocrine and cardiovascular responses to intense stress |
|  | Regulation of hypothalamic-pituitary-interrenal axis function in male smallmouth bass (Micropterus dolomieu) during parental care |
|  | Less immune activation following social stress in rural vs. urban participants raised with regular or no animal contact, respectively |
|  | Functional TSPO polymorphism predicts variance in the diurnal cortisol rhythm in bipolar disorder |
|  | Interaction of HPA axis genetics and early life stress shapes emotion recognition in healthy adults |
|  | Endocrine and hematological responses of beef heifers divergently ranked for residual feed intake following a bovine corticotropin-releasing hormone challenge |
|  | Combined dexamethasone suppression-corticotrophin-releasing hormone stimulation test in medication-free major depression and healthy volunteers |
|  | Cortisol fluctuations relate to interictal epileptiform discharges in stress sensitive epilepsy |
|  | Cortisol modulates vasotocinergic and isotocinergic pathways in the gilthead sea bream |
|  | Stress intensifies demands on response selection during action cascading processes |
|  | Competing targets of microRNA-608 affect anxiety and hypertension |
|  | Salivary markers of stress system activation and social withdrawal in humans |
|  | Hypothalamic-pituitary-adrenal axis attenuation and obesity risk in sexually abused females |
|  | Fine particulate matter constituents and stress hormones in the hypothalamus-pituitary-adrenal axis |
|  | Childhood abuse is associated with increased hair cortisol levels among urban pregnant women |
|  | Maternal programming of offspring hypothalamic-pituitary-interrenal axis in wild sockeye salmon (Oncorhynchus nerka) |
|  | Linking plasma cortisol levels to phenotypic heterogeneity of posttraumatic stress symptomatology |
|  | HPA axis dysregulation, NR3C1 polymorphisms and glucocorticoid receptor isoforms imbalance in metabolic syndrome |
|  | Saliva cortisol levels and physiological parameter fluctuations in mild traumatic brain injury patients compared to controls |
|  | Effects of diets high in unsaturated Fatty acids on socially induced stress responses in Guinea pigs |
|  | Cross-talk between adipose tissue and the HPA axis in obesity and overt hypercortisolemic states |
|  | Effects of subchronic exposure to waterborne cadmium on H-P-I axis hormones and related genes in rare minnows (Gobiocypris rarus) |
|  | Neural, Hormonal, and Cognitive Correlates of Metabolic Dysfunction and Emotional Reactivity |
|  | Characterizing the psychophysiological signature of boredom |
|  | Exploring patterns in cortisol synchrony among anxious and nonanxious mother and child dyads: a preliminary study |
|  | Omega-3 fatty acids and anxiety: A systematic review of the possible mechanisms at play |
|  | Mineralocorticoid receptor stimulation improves cognitive function and decreases cortisol secretion in depressed patients and healthy individuals |
|  | "More than skin deep": stress neurobiology and mental health consequences of racial discrimination |
|  | In pregnancy increased maternal STAI trait stress score shows decreased insulin sensitivity and increased stress hormones |
|  | Longitudinal changes in serum catecholamines, dopamine, serotonin, ACTH and cortisol in pregnant Spanish mares |
|  | Cortisol-induced immune suppression by a blockade of lymphocyte egress in traumatic brain injury |
|  | Short communication: Ovine leukocyte telomere length is associated with variation in the cortisol response to systemic bacterial endotoxin challenge |
|  | Cerebrospinal fluid cortisol and progesterone profiles and outcomes prognostication after severe traumatic brain injury |
|  | Association of pain intensity, pain-related disability, and depression with hypothalamus-pituitary-adrenal axis function in female patients with chronic temporomandibular disorders |
|  | [Pathophysiological aspects of neuro-endocrine regulation system in patients with obstructive sleep apnea syndrome] |
|  | Is afternoon cortisol more reliable than waking cortisol in association studies of children with an ASD? |
|  | An inverted U-shaped relationship between cortisol awakening response and same-day error monitoring function in healthy males |
|  | Transdermal neuromodulation of noradrenergic activity suppresses psychophysiological and biochemical stress responses in humans |
|  | Hypothalamic-Pituitary-Adrenocortical Axis Activity in Alcohol-Dependent Patients During Treatment with High-Dose Baclofen |
|  | Diet matters: Glucocorticoid-related neuroadaptations associated with calorie intake in female rhesus monkeys |
|  | High dose hydrocortisone immediately after trauma may alter the trajectory of PTSD: interplay between clinical and animal studies |
|  | No hypothalamic-pituitary-adrenal function effect with beclomethasone dipropionate nasal aerosol, based on 24-hour serum cortisol in pediatric allergic rhinitis |
|  | Perinatal determinants of neonatal hair glucocorticoid concentrations |
|  | Adrenal response to ACTH challenge alters thyroid and immune function and varies with body reserves in molting adult female northern elephant seals |
|  | Hypothalamic-pituitary-adrenal axis recovery following prolonged prednisolone therapy in infants |
|  | A low cortisol response to stress is associated with musculoskeletal pain combined with increased pain sensitivity in young adults: a longitudinal cohort study |
|  | Dexamethasone-suppressed Salivary Cortisol and Pain Sensitivity in Female Twins |
|  | Sex-specific association between functional neuropeptide S receptor gene (NPSR1) variants and cortisol and central stress responses |
|  | Circadian rhythms in rheumatology--a glucocorticoid perspective |
|  | Cortisol, cortisone, and BDNF in amniotic fluid in the second trimester of pregnancy: Effect of early life and current maternal stress and socioeconomic status |
|  | Early life adversity reduces stress reactivity and enhances impulsive behavior: implications for health behaviors |
|  | Glucocorticoid treatment earlier in childhood and adolescence show dose-response associations with diurnal cortisol levels |
|  | Time matters - acute stress response and glucocorticoid sensitivity in early multiple sclerosis |
|  | Suckling and salsolinol attenuate responsiveness of the hypothalamic-pituitary-adrenal axis to stress: focus on catecholamines, corticotrophin-releasing hormone, adrenocorticotrophic hormone, cortisol and prolactin secretion in lactating sheep |
|  | Posttraumatic stress symptoms and cortisol regulation in mothers of very preterm infants |
|  | Not just sticks and stones: Indirect ethnic discrimination leads to greater physiological reactivity |
|  | The Hypothalamic-Pituitary-Adrenal Axis and the Fetus |
|  | Sex differences in Alzheimer's disease risk: are we looking at the wrong hormones? |
|  | Kisspeptin, c-Fos and CRFR type 2 co-expression in the hypothalamus after insulin-induced hypoglycaemia |
|  | Cognitive control moderates parenting stress effects on children's diurnal cortisol |
|  | Disruption of Rich-Club Connectivity in Cushing Disease |
|  | Childhood Trauma Is Associated With Poorer Cognitive Performance in Older Adults |
|  | The Effects of Stress on Cognitive Aging, Physiology and Emotion (ESCAPE) Project |
|  | Exploring the use of thermal infrared imaging in human stress research |
|  | Increased HPA axis response to psychosocial stress in remitted depression: the influence of coping style |
|  | An ecologically relevant guinea pig model of fetal behavior |
|  | The role of psychopathic traits, social anxiety and cortisol in social approach avoidance tendencies |
|  | The relationship between dehydroepiandrosterone (DHEA), working memory and distraction--a behavioral and electrophysiological approach |
|  | Infant HPA axis as a potential mechanism linking maternal mental health and infant telomere length |
|  | Sex and stress: Men and women show different cortisol responses to psychological stress induced by the Trier social stress test and the Iowa singing social stress test |
|  | Massive attack of honeybee on macaws (Ara ararauna and Ara chloropterus) in Brazil - A case report |
|  | Obstructive sleep apnea syndrome causes a pseudo-Cushing's state in Japanese obese patients with type 2 diabetes mellitus |
|  | Stress exacerbates pain in the everyday lives of women with fibromyalgia syndrome--The role of cortisol and alpha-amylase |
|  | Diurnal cortisol rhythm as a predictor of lung cancer survival |
|  | Dysregulation of the hypothalamic pituitary adrenal (HPA) axis and physical performance at older ages: an individual participant meta-analysis |
|  | Influence of vegetable diets on physiological and immune responses to thermal stress in Senegalese sole (Solea senegalensis) |
|  | Antenatal depression programs cortisol stress reactivity in offspring through increased maternal inflammation and cortisol in pregnancy: The Psychiatry Research and Motherhood - Depression (PRAM-D) Study |
|  | A translational investigation targeting stress-reactivity and prefrontal cognitive control with guanfacine for smoking cessation |
|  | Prognostic value of plasma neuroendocrine biomarkers in patients with acute ischaemic stroke |
|  | Short-Term d-Aspartic Acid Supplementation Does Not Affect Serum Biomarkers Associated With the Hypothalamic-Pituitary-Gonadal Axis in Male Climbers |
|  | Exaggerated activity of HPA axis in obese rats fed normocaloric liquid nutrition |
|  | Dissociation of endocrine responses to the Trier Social Stress Test in Virtual Reality (VR-TSST) by the benzodiazepine alprazolam and the translocator protein 18 kDa (TSPO) ligand etifoxine |
|  | Blunted cortisol response to psychosocial stress in atopic patients is associated with decrease in salivary alpha-amylase and aldosterone: Focus on sex and menstrual cycle phase |
|  | Stress reactivity in childhood functional abdominal pain or irritable bowel syndrome |
|  | [Psychological and biological background of the correlation between psoriasis and stress] |
|  | Effects of acute social stress on emotion processing in children |
|  | Melatonin and cortisol profiles in the absence of light perception |
|  | Autonomic and Adrenocortical Interactions Predict Mental Health in Late Adolescence: The TRAILS Study |
|  | Early exposure to parental depression and parenting: associations with young offspring's stress physiology and oppositional behavior |
|  | Functional Correlates and Impact of Dietary Lactoferrin Intervention and its Concentration-dependence on Neurodevelopment and Cognition in Neonatal Piglets |
|  | Acute effects of intravenous heroin on the hypothalamic-pituitary-adrenal axis response: a controlled trial |
|  | Higher cortisol levels at diurnal trough predict greater attentional bias towards threat in healthy young adults |
|  | Cortisol response to stress in schizophrenia: Associations with oxytocin, social support and social functioning |
|  | Reward anticipation modulates the effect of stress-related increases in cortisol on episodic memory |
|  | Involvement of cortisol and sirtuin1 during the response to stress of hypothalamic circadian system and food intake-related peptides in rainbow trout, Oncorhynchus mykiss |
|  | Victims of war-Psychoendocrine evidence for the impact of traumatic stress on psychological well-being of adolescents growing up during the Israeli-Palestinian conflict |
|  | Effects of oxytocin on cortisol reactivity and conflict resolution behaviors among couples with substance misuse |
|  | In response: Letter on update to the Vitamin C, Thiamine and Steroids in Sepsis (VICTAS) protocol |
|  | Cortisol Co-Secretion and Clinical Usefulness of ACTH Stimulation Test in Primary Aldosteronism: A Systematic Review and Biases in Epidemiological Studies |
|  | Recent life stress predicts blunted acute stress response and the role of executive control |
|  | Infant hair cortisol: associations with salivary cortisol and environmental context |
|  | Heterogeneity in HPA axis dysregulation and serotonergic vulnerability to depression |
|  | Association of Sleep Duration and Quality With Alterations in the Hypothalamic-Pituitary Adrenocortical Axis: The Multi-Ethnic Study of Atherosclerosis (MESA) |
|  | Hair cortisol in captive corral-housed baboons |
|  | How to measure glucocorticoid receptor's sensitivity in patients with stress-related psychiatric disorders |
|  | Enhanced cortisol secretion in acute transient global amnesia |
|  | Hypocortisolism and preterm birth |
|  | Effects of early life adversity on cortisol/salivary alpha-amylase symmetry in free-ranging juvenile rhesus macaques |
|  | Endocannabinoid-related lipids are increased during an episode of cyclic vomiting syndrome |
|  | Hypothalamic-pituitary-adrenal axis in lethal canine Staphylococcus aureus pneumonia |
|  | Role of BDNF val66met polymorphism in modulating exercised-induced emotional memories |
|  | Preoperative and Postoperative Pituitary Function in Patients with Tuberculum Sellae Meningioma -Based on Pituitary Provocation Tests |
|  | A meta-analytic review of the association between cortisol reactivity in response to a stressor and attention-deficit hyperactivity disorder |
|  | Smaller grey matter volumes in the anterior cingulate cortex and greater cerebellar volumes in patients with long-term remission of Cushing's disease: a case-control study |
|  | The effects of early trauma and the FKBP5 gene on PTSD and the HPA axis in a clinical sample of Gulf War veterans |
|  | Changes in Salivary Cortisol During Psychotherapy for Posttraumatic Stress Disorder: A Pilot Study in 30 Veterans |
|  | Dysregulated physiological stress systems and accelerated cellular aging |
|  | The effect of movement-focused and breath-focused yoga practice on stress parameters and sustained attention: A randomized controlled pilot study |
|  | Yawning and cortisol levels in multiple sclerosis: Potential new diagnostic tool |
|  | Cortisol response to an experimental stress paradigm prospectively predicts long-term distress and resilience trajectories in response to active police service |
|  | Prenatal maternal depression is associated with offspring inflammation at 25 years: a prospective longitudinal cohort study |
|  | The association between perceived emotional support, maternal mood, salivary cortisol, salivary cortisone, and the ratio between the two compounds in response to acute stress in second trimester pregnant women |
|  | Acute stress alters individual risk taking in a time-dependent manner and leads to anti-social risk |
|  | Acute Stress and Perceptual Load Consume the Same Attentional Resources: A Behavioral-ERP Study |
|  | Effects of adverse childhood experiences on the association between intranasal oxytocin and social stress reactivity among individuals with cocaine dependence |
|  | Brief cognitive intervention can modulate neuroendocrine stress responses to the Trier Social Stress Test: buffering effects of a compassionate goal orientation |
|  | High activity of the stress promoter contributes to susceptibility to stress in the tree shrew |
|  | Association Between Interleukin-6 and Striatal Prediction-Error Signals Following Acute Stress in Healthy Female Participants |
|  | Analysis of serum cortisol to predict recovery in paediatric sport-related concussion |
|  | Human milk cortisol concentration predicts experimentally induced infant fear reactivity: moderation by infant sex |
|  | Macrophage migration inhibitory factor (MIF) gene is associated with adolescents' cortisol reactivity and anxiety |
|  | Diurnal Cortisol Rhythm Is Associated With Adverse Cardiac Events and Mortality in Coronary Artery Bypass Patients |
|  | Response to Xuerong Luo et al., Letter to the Editor |
|  | Maternal hypothalamus-pituitary-adrenal (HPA) system activity and stress during pregnancy: Effects on gestational age and infant's anthropometric measures at birth |
|  | Care Transitions and Adult Day Services Moderate the Longitudinal Links between Stress Biomarkers and Family Caregivers' Functional Health |
|  | Salivary stress biomarkers of recent nicotine use and dependence |
|  | The relationship between self-reported childhood adversities, adulthood psychopathology and psychological stress markers in patients with schizophrenia |
|  | Perinatal Depression, Adverse Life Events, and Hypothalamic-Adrenal-Pituitary Axis Response to Cold Pressor Stress in Latinas: An Exploratory Study |
|  | Dietary fat and corticosterone levels are contributing factors to meal anticipation |
|  | The effects of two different doses of hydrocortisone on cognition in patients with secondary adrenal insufficiency--results from a randomized controlled trial |
|  | A randomised controlled trial of expressive arts-based intervention for young stroke survivors |
|  | Endogenous Cortisol Exposure and Declarative Verbal Memory: A Longitudinal Study of Healthy Older Adults |
|  | Pituitary ACTH-secreting adenoma in Addison's disease: a case report |
|  | Effects of cognitive-behavioural therapy for stress management on stress and hair cortisol levels in pregnant women: A randomised controlled trial |
|  | Newborn infants' hair cortisol levels reflect chronic maternal stress during pregnancy |
|  | Effects of Positive Psychology Interventions on Risk Biomarkers in Coronary Patients: A Randomized, Wait-List Controlled Pilot Trial |
|  | Unpredictable chronic stress decreases inhibitory avoidance learning in Tuebingen long-fin zebrafish: stronger effects in the resting phase than in the active phase |
|  | Blunted neuroactive steroid and HPA axis responses to stress are associated with reduced sleep quality and negative affect in pregnancy: a pilot study |
|  | Cortisol evaluation during the acute phase of traumatic brain injury-A prospective study |
|  | Obstructive Sleep Apnea in Gestational Diabetes: A Pilot Study of the Role of the Hypothalamic-Pituitary-Adrenal Axis |
|  | A soy-based phosphatidylserine/ phosphatidic acid complex (PAS) normalizes the stress reactivity of hypothalamus-pituitary-adrenal-axis in chronically stressed male subjects: a randomized, placebo-controlled study |
|  | Immunoassay interference complicating management of Cushing's disease: the onus is on the clinician and the laboratory |
|  | Role of sampling times and serum cortisol cut-off concentrations on the routine assessment of adrenal function using the standard cosyntropin test in an academic hospital from Spain: a retrospective chart review |
|  | Adrenocortical sensitivity, moderated by ongoing stress, predicts drinking intensity in alcohol-dependent men |
|  | Cerebral perfusion in sepsis-associated delirium |
|  | Effects of tail docking and castration on stress responses in lambs and the influence of prenatal glucocorticoid treatment |
|  | Stress in childhood, adolescence and early adulthood, and cortisol levels in older age |
|  | Longitudinal sex and stress hormone profiles among reproductive age and post-menopausal women after severe TBI: A case series analysis |
|  | All-trans retinoic acid-induced hypothalamus-pituitary-adrenal hyperactivity involves glucocorticoid receptor dysregulation |
|  | Salivary biomarkers of stress and inflammation in first graders in Côte d'Ivoire: Effects of a probiotic food intervention |
|  | Evaluation of acute tryptophan depletion and sham depletion with a gelatin-based collagen peptide protein mixture |
|  | Sleep and hypothalamic pituitary adrenal axis responses to metyrapone in posttraumatic stress disorder |
|  | Stress modulates reinforcement learning in younger and older adults |
|  | Increased Total Urinary Cortisol (tUC) and Serum Brain-derived Neurotrophic Factor (BDNF) Ratio in Alzheimer Disease (AD)-affected Patients |
|  | Pituitary abscess |
|  | Dehydroepiandrosterone and dehydroepiandrosterone sulfate: anabolic, neuroprotective, and neuroexcitatory properties in military men |
|  | Dilated cardiomyopathy as a presenting feature of Cushing's syndrome |
|  | Deep brain stimulation for obsessive-compulsive disorder is associated with cortisol changes |
|  | Hypothalamic-pituitary-adrenal axis response to acute psychosocial stress: Effects of biological sex and circulating sex hormones |
|  | Greater inflammatory activity and blunted glucocorticoid signaling in monocytes of chronically stressed caregivers |
|  | Attenuated DHEA and DHEA-S response to acute psychosocial stress in individuals with depressive disorders |
|  | Behavioral Assessment of Stress Compensation in Minipigs Transgenic for the Huntington Gene Using Cortisol Levels: A Proof-of-Concept Study |
|  | Oxytocin administration alters HPA reactivity in the context of parent-infant interaction |
|  | Prenatal programming in an obese swine model: sex-related effects of maternal energy restriction on morphology, metabolism and hypothalamic gene expression |
|  | Hormonal Status and Cognitivo-Emotional Profile in Real-Life Patients With Neuropathic Pain: A Case Control Study |
|  | Longitudinal association between psychosocial stress and retinal microvasculature in children and adolescents |
|  | Prenatal intimate partner violence exposure predicts infant biobehavioral regulation: Moderation by the brain-derived neurotrophic factor (BDNF) gene |
|  | Immediate and delayed neuroendocrine responses to social exclusion in males and females |
|  | Changes in behavior and salivary cortisol after targeted cognitive training in typical 12-month-old infants |
|  | The Early Endocrine Stress Response in Experimental Subarachnoid Hemorrhage |
|  | Glucocorticoid synthesis inhibitor metyrapone blocks stress-induced suppression along luteinizing hormone secreting cells–ovary axis in the fish Oreochromis mossambicus |
|  | Brain-derived neurotrophic factor (BDNF) Val66Met polymorphism interacts with gender to influence cortisol responses to mental stress |
|  | Cortisol awakening response is blunted and pain perception is increased during menses in cyclic women |
|  | An in vivo explorative study to observe the protective effects of Puerariae flos extract on chronic ethanol exposure and withdrawal male mice |
|  | Biological predictors of insulin resistance associated with posttraumatic stress disorder in young military veterans |
|  | Temporomandibular Disorders Related to Stress and HPA-Axis Regulation |
|  | Dexamethasone PONV prophylaxis alters the hypothalamic-pituitary-adrenal axis after transsphenoidal pituitary surgery |
|  | Pituitary control of branchial NCC, NKCC and Na(+), K (+)-ATPase α-subunit gene expression in Nile tilapia, Oreochromis niloticus |
|  | Maternal depression and cortisol in pregnancy predict offspring emotional reactivity in the preschool period |
|  | Variability and reliability of diurnal cortisol in younger and older adults: implications for design decisions |
|  | Contemplative Mental Training Reduces Hair Glucocorticoid Levels in a Randomized Clinical Trial |
|  | The Severity of Acute Stress Is Represented by Increased Synchronous Activity and Recruitment of Hypothalamic CRH Neurons |
|  | A Comprehensive Evaluation of Steroid Metabolism in Women with Intrahepatic Cholestasis of Pregnancy |
|  | Counter-regulatory response to a fall in circulating fatty acid levels in rainbow trout. Possible involvement of the hypothalamus-pituitary-interrenal axis |
|  | Maternal stimulation in infancy predicts hypothalamic-pituitary-adrenal axis reactivity in young men |
|  | Identification of environmental stressors and validation of light preference as a measure of anxiety in larval zebrafish |
|  | Full-term deliveries without antecedent labor reveal sex differences in umbilical cord glucocorticoid concentrations |
|  | Markers of stress and inflammation as potential mediators of the relationship between exercise and depressive symptoms: findings from the TRAILS study |
|  | Moderate dose cranial radiotherapy causes central adrenal insufficiency in long-term survivors of childhood leukaemia |
|  | Hardness does not affect the physiological responses of wild and domestic strains of diploid and triploid rainbow trout Oncorhynchus mykiss to short-term exposure to pH 9.5 |
|  | Integrative psycho-biophysiological markers in predicting psychological resilience |
|  | Mild cognitive deficits in patients with primary adrenal insufficiency |
|  | Apparent Hypothalamic-Pituitary-Adrenal Axis Suppression via Reduction of Interleukin-6 by Glucocorticoid Therapy in Systemic Autoimmune Diseases |
|  | History of stress-related health changes: a cue to pursue a diagnosis of latent primary adrenal insufficiency |
|  | C3435T polymorphism of the MDR1 gene is not associated with blood levels of hypothalamus-pituitary-adrenal axis hormones in healthy male subjects |
|  | FKBP5 polymorphisms and hypothalamic-pituitary-adrenal axis negative feedback in major depression and obsessive-compulsive disorder |
|  | Shortening sow restraint period during lactation improves production and decreases hair cortisol concentrations in sows and their piglets |
|  | Hydrogen sulfide measurement using sulfide dibimane: critical evaluation with electrospray ion trap mass spectrometry |
|  | Childhood maltreatment increases the risk for visceral obesity |
|  | Resetting the Stress System with a Mifepristone Challenge |
|  | Psychosocial stress differentially affects emotional empathy in women with borderline personality disorder and healthy controls |
|  | Sex differences in the associations between maternal prenatal distress and infant cortisol reactivity and recovery |
|  | Adrenal function in cats with cholestatic liver disease |
|  | Increased left ventricular mass in hypercortisolemic depressed patients: a hypothesis based on a case series |
|  | Acclimation to different environmental salinities induces molecular endocrine changes in the GH/IGF-I axis of juvenile gilthead sea bream (Sparus aurata L.) |
|  | Acute physiological stress down-regulates mRNA expressions of growth-related genes in coho salmon |
|  | Resuscitation from hemorrhagic shock after traumatic brain injury with polymerized hemoglobin |
|  | Urinary bisphenol A is associated with dysregulation of HPA-axis function in pregnant women: Findings from the APrON cohort study |
|  | Anti-Inflammatory Modulation of Microglia via CD163-Targeted Glucocorticoids Protects Dopaminergic Neurons in the 6-OHDA Parkinson's Disease Model |
|  | Salivary cortisol and behavioral response to social evaluative threat in adolescents with autism spectrum disorder |
|  | Maternal postnatal depression predicts altered offspring biological stress reactivity in adulthood |
|  | Long-term remission and recurrence rates after first and second transsphenoidal surgery for Cushing's disease: care reality in the Munich Metropolitan Region |
|  | Relation among HPA and HPG neuroendocrine systems, transmissible risk and neighborhood quality on development of substance use disorder: results of a 10-year prospective study |
|  | Associations between Parity, Hair Hormone Profiles during Pregnancy and Lactation, and Infant Development in Rhesus Monkeys (Macaca mulatta) |
|  | Changes Induced by Mind-Body Intervention Including Epigenetic Marks and Its Effects on Diabetes |
|  | Cortisol response patterns in depressed women and their healthy daughters at risk: Comparison with healthy women and their daughters |
|  | Imbalance in the diurnal salivary testosterone/cortisol ratio in men with severe obstructive sleep apnea: an observational study |
|  | HPA axis dysregulation in men with hypersexual disorder |
|  | Corticosteroid Fludrocortisone Acetate Targets Multiple End Points in Zebrafish (Danio rerio) at Low Concentrations |
|  | Early life adversity influences stress response association with smoking relapse |
|  | Survival risk of salivary cortisol and serum N-terminal pro-hormone B-type natriuretic peptide in patients with systolic heart failure |
|  | Problematic Internet use, excessive alcohol consumption, their comorbidity and cardiovascular and cortisol reactions to acute psychological stress in a student population |
|  | Comparison of methods for evaluation of the suppressive effects of prednisolone on the HPA axis and bone turnover: changes in s-DHEAS are as sensitive as the ACTH test |
|  | Cognitive impairment and cortisol levels in first-episode schizophrenia patients |
|  | Effects of adverse life events on heart rate variability, cortisol, and C-reactive protein |
|  | The relationship between the menstrual cycle and cortisol secretion: Daily and stress-invoked cortisol patterns |
|  | Investigation of genetic variants, birthweight and hypothalamic-pituitary-adrenal axis function suggests a genetic variant in the SERPINA6 gene is associated with corticosteroid binding globulin in the western Australia pregnancy cohort (Raine) study |
|  | HPA-axis stress reactivity in youth depression: evidence of impaired regulatory processes in depressed boys |
|  | Effect of artificial dawn light on cardiovascular function, alertness, and balance in middle-aged and older adults |
|  | Principal components derived from CSF inflammatory profiles predict outcome in survivors after severe traumatic brain injury |
|  | In the face of threat: neural and endocrine correlates of impaired facial emotion recognition in cocaine dependence |
|  | Blunted glucocorticoid and mineralocorticoid sensitivity to stress in people with diabetes |
|  | SERUM FREE CORTISOL DURING GLUCAGON STIMULATION TEST IN HEALTHY SHORT-STATURED CHILDREN AND ADOLESCENTS |
|  | Men and women differ in inflammatory and neuroendocrine responses to endotoxin but not in the severity of sickness symptoms |
|  | Yoga practice improves executive function by attenuating stress levels |
|  | Socioeconomic status, hair cortisol and internalizing symptoms in parents and children |
|  | Increased cortisol awakening response after completing the summer treatment program in children with ADHD |
|  | Autoantibodies reactive to adrenocorticotropic hormone can alter cortisol secretion in both aggressive and nonaggressive humans |
|  | The Vitamin C, Thiamine and Steroids in Sepsis (VICTAS) Protocol: a prospective, multi-center, double-blind, adaptive sample size, randomized, placebo-controlled, clinical trial |
|  | Associations of SLC6A4 methylation with salivary cortisol, salivary alpha-amylase, and subjective stress in everyday life |
|  | Blunted hypothalamic-pituitary-adrenal axis and insulin response to psychosocial stress in young adults born preterm at very low birth weight |
|  | Elevated fetal steroidogenic activity in autism |
|  | Stress and decision making: a few minutes make all the difference |
|  | Open and Calm--a randomized controlled trial evaluating a public stress reduction program in Denmark |
|  | Secretory IgA reactivity to social threat in youth: Relations with HPA, ANS, and behavior |
|  | Resilience is decreased in irritable bowel syndrome and associated with symptoms and cortisol response |
|  | Disturbances in Hypothalamic-Pituitary-Adrenal Axis and Immunological Activity Differentiating between Unipolar and Bipolar Depressive Episodes |
|  | Bradykinin B2 receptor in the adrenal medulla of male rats and mice: glucocorticoid-dependent increase with immobilization stress |
|  | HPA axis activity in multiple sclerosis correlates with disease severity, lesion type and gene expression in normal-appearing white matter |
|  | Children's altruism following acute stress: The role of autonomic nervous system activity and social support |
|  | Sexual Dimorphism in Glucocorticoid Stress Response |
|  | Effects of childhood trauma exposure and cortisol levels on cognitive functioning among breast cancer survivors |
|  | Effects of IL-6 and cortisol fluctuations in post-stroke depression |
|  | Increased release of dopamine in the striata of young adults with hearing impairment and its relevance for the social defeat hypothesis of schizophrenia |
|  | Lactoferrin Promotes Early Neurodevelopment and Cognition in Postnatal Piglets by Upregulating the BDNF Signaling Pathway and Polysialylation |
|  | The imaging Maastricht Acute Stress Test (iMAST): a neuroimaging compatible psychophysiological stressor |
|  | Safety, Pharmacokinetics and Pharmacodynamics of the Selective Glucocorticoid Receptor Modulator AZD5423 after Inhalation in Healthy Volunteers |
|  | Multifactorial analyses revealed optimal aquaculture modalities improving husbandry fitness without clear effect on stress and immune status of pikeperch Sander lucioperca |
|  | Oxytocin and HPA stress axis reactivity in postpartum women |
|  | Post-dexamethasone serum copeptin corresponds to HPA axis responsiveness in human obesity |
|  | The submariners' sleep study: a field investigation of sleep and circadian hormones during a 67-day submarine mission with a strict 6-h-on/6-h-off watch routine |
|  | Effects of overshadowing on conditioned and unconditioned nausea in a rotation paradigm with humans |
|  | Efficacy of Souroubea-Platanus Dietary Supplement Containing Triterpenes in Beagle Dogs Using a Thunderstorm Noise-Induced Model of Fear and Anxiety |
|  | A randomized controlled study of power posing before public speaking exposure for social anxiety disorder: No evidence for augmentative effects |
|  | Serum cortisol and BDNF in patients with major depression-effect of yoga |
|  | Reduction of Glucocorticoid Receptor Function in Chronic Fatigue Syndrome |
|  | Physiological, psychosocial, and environmental factors in depression among autistic girls |
|  | HPA axis response and psychosocial stress as interactive predictors of suicidal ideation and behavior in adolescent females: a multilevel diathesis-stress framework |
|  | Lifetime exposure to traumatic and other stressful life events and hair cortisol in a multi-racial/ethnic sample of pregnant women |
|  | Effects of acute cortisol administration on response inhibition in patients with major depression and healthy controls |
|  | Aberrant G-protein coupled hormone receptor in adrenal diseases |
|  | Daytime light exposure: effects on biomarkers, measures of alertness, and performance |
|  | Low-Dose Testosterone Augmentation for Antidepressant-Resistant Major Depressive Disorder in Women: An 8-Week Randomized Placebo-Controlled Study |
|  | Differential effects of eating and drinking on wellbeing-An ecological ambulatory assessment study |
|  | Getting better, but not well: A 1.5 year follow-up of cognitive performance and cortisol levels in clinical and non-Clinical burnout |
|  | Maternal intimate partner violence exposure, child cortisol reactivity and child asthma |
|  | Endocrine stress response in pregnancy and 12 weeks postpartum - Exploring risk factors for postpartum depression |
|  | Stem cell-based interventions for the prevention of morbidity and mortality following hypoxic-ischaemic encephalopathy in newborn infants |
|  | Intervention effects on diurnal cortisol rhythms of Child Protective Services-referred infants in early childhood: preschool follow-up results of a randomized clinical trial |
|  | Reduced stress and inflammatory responsiveness in experienced meditators compared to a matched healthy control group |
|  | Effect of levodopa/carbidopa on stress response in zebrafish |
|  | Neuropsychiatric and cardiometabolic comorbidities in patients with previously diagnosed Cushing's disease: a longitudinal observational study |
|  | The interactive Physical and Cognitive Exercise System (iPACES™): effects of a 3-month in-home pilot clinical trial for mild cognitive impairment and caregivers |
|  | Cortisol reactivity and emotional memory after psychosocial stress in oral contraceptive users |
|  | Enhanced orienting of attention in response to emotional gaze cues after oxytocin administration in healthy young men |
|  | Hypothalamic-pituitary-adrenal axis response to oral naltrexone in alcoholics during early withdrawal |
|  | How do individuals cope with stress? Behavioural, physiological and neuronal differences between proactive and reactive coping styles in fish |
|  | Differential neuroendocrine and immune responses to acute psychosocial stress in women with type 1 bipolar disorder |
|  | Further characterisation of differences between TL and AB zebrafish (Danio rerio): Gene expression, physiology and behaviour at day 5 of the larval stage |
|  | Different levels of brain-derived neurotrophic factor and cortisol in healthy heavy smokers |
|  | Affective and inflammatory responses among orchestra musicians in performance situation |
|  | The response of C19- and some C21-steroids during Synacthen and insulin tolerance test |
|  | Selection and early clinical evaluation of the brain-penetrant 11β-hydroxysteroid dehydrogenase type 1 (11β-HSD1) inhibitor UE2343 (Xanamem™) |
|  | Lactobacillus rhamnosus CNCM I-3690 decreases subjective academic stress in healthy adults: a randomized placebo-controlled trial |
|  | Clinical and Biochemical Outcomes Following EEG Neurofeedback Training in Traumatic Brain Injury in the Context of Spontaneous Recovery |
|  | NCB5OR Deficiency in the Cerebellum and Midbrain Leads to Dehydration and Alterations in Thirst Response, Fasted Feeding Behavior, and Voluntary Exercise in Mice |
|  | Medical Treatment for Acromegaly does not Increase the Risk of Central Adrenal Insufficiency: A Long-Term Follow-Up Study |
|  | INVITED REVIEW: The usefulness of measuring glucocorticoids for assessing animal welfare |
|  | Maternal psychological distress during gestation is associated with infant food allergy |
|  | Home alone-The effects of isolation on uptake of a pharmaceutical contaminant in a social fish |
|  | Cardiovascular Instability Preceded by Orolingual Angioedema after Alteplase Treatment |
|  | Effect of Arginine on the Hypothalamic-Pituitary-Adrenal Axis in Individuals With and Without Vasopressin Deficiency |
|  | Hypothalamic-pituitary-adrenal-axis dysregulation and double product increases potentiate ischemic heart disease risk in a Black male cohort: the SABPA study |
|  | Why are depressed patients inflamed? A reflection on 20 years of research on depression, glucocorticoid resistance and inflammation |
|  | The Tutsi genocide and transgenerational transmission of maternal stress: epigenetics and biology of the HPA axis |
|  | Transcription elements and functional expression of proopiomelanocortin genes in the pituitary gland of the barfin flounder |
|  | Autologous serum collected 1 h post-exercise enhances natural killer cell cytotoxicity |
|  | Myrcia sylvatica essential oil mitigates molecular, biochemical and physiological alterations in Rhamdia quelen under different stress events associated to transport |
|  | Cortisol as a prognostic marker of short-term outcome in chinese patients with acute ischemic stroke |
|  | Functional neuroanatomy and neural oscillations during social eavesdropping in male golden hamsters |
|  | Self-reported mindfulness and cortisol during a Shamatha meditation retreat |
|  | Sex-dependent effects of stress on brain correlates to empathy for pain |
|  | Involvement of PKCα and ERK1/2 signaling pathways in EGCG's protection against stress-induced neural injuries in Wistar rats |
|  | Moderate alcohol consumption after a mental stressor attenuates the endocrine stress response |
|  | Psychological Stress Deteriorates Skin Barrier Function by Activating 11β-Hydroxysteroid Dehydrogenase 1 and the HPA Axis |
|  | Hypothalamo-pituitary-adrenal axis after a single epidural triamcinolone injection |
|  | Effect of Hydrocortisone on Mortality and Organ Support in Patients With Severe COVID-19: The REMAP-CAP COVID-19 Corticosteroid Domain Randomized Clinical Trial |
|  | Relations between feeding intolerance and stress biomarkers in preterm infants |
|  | 520-d Isolation and confinement simulating a flight to Mars reveals heightened immune responses and alterations of leukocyte phenotype |
|  | Fear of pain and cortisol reactivity predict the strength of stress-induced hypoalgesia |
|  | Sexual orientation and disclosure in relation to psychiatric symptoms, diurnal cortisol, and allostatic load |
|  | Effects of ZnSO(4)-induced peripheral anosmia on zebrafish behavior and physiology |
|  | Hyperdehydroepiandrosterone in neonates with hypoxic ischemic encephalopathy and circulatory collapse |
|  | Cortisol and Brain-Derived Neurotrophic Factor Levels Prior to Treatment in Children With Obsessive-Compulsive Disorder |
|  | Associations between CSF cortisol and CSF norepinephrine in cognitively normal controls and patients with amnestic MCI and AD dementia |
|  | Involuntary swimming exercise in pregnant rats disturbs ERK1/2 signaling in embryonic neurons through increased cortisol in the amniotic fluid |
|  | Effects of adolescent sociocognitive development on the cortisol response to social evaluation |
|  | Prebiotic intake reduces the waking cortisol response and alters emotional bias in healthy volunteers |
|  | Associations of prenatal depressive symptoms with DNA methylation of HPA axis-related genes and diurnal cortisol profiles in primary school-aged children |
|  | Mifepristone as a therapeutic agent in psychiatry |
|  | Bipolar affective disorder and borderline personality disorder: Differentiation based on the history of early life stress and psychoneuroendocrine measures |
|  | Early adversity contributes to chronic stress induced depression-like behavior in adolescent male rhesus monkeys |
|  | A biopsychosocial perspective on maternal parenting in the first two years of infant life |
|  | Anna-Monika Award Lecture, DGPPN Kongress, 2013: the role of the hypothalamic-pituitary-adrenal (HPA) axis in the pathogenesis of psychotic major depression |
|  | Effect of primary empty sella syndrome on pituitary surgery for Cushing's disease |
|  | Cortisol reactivity to stress among youth: stability over time and genetic variants for stress sensitivity |
|  | Ontogeny of the adrenal gland in the spiny mouse, with particular reference to production of the steroids cortisol and dehydroepiandrosterone |
|  | [Importance of the 11β-hydroxysteroid dehydrogenase enzyme in clinical disorders] |
|  | Isolation and sequence of a cDNA clone which contains the complete coding region of rat phenylalanine hydroxylase. Structural homology with tyrosine hydroxylase, glucocorticoid regulation, and use of alternate polyadenylation sites |
|  | Understanding oral stereotypies in calves: alternative strategies, hypothalamic-pituitary-adrenal axis (re)activity and gene by environment interactions |
|  | Effects of resistance exercise on the HPA axis response to psychological stress during short-term smoking abstinence in men |
|  | Hair cortisol and work stress: Importance of workload and stress model (JDCS or ERI) |
|  | Efficacy of therapeutic play for pediatric brain tumor patients during external beam radiotherapy |
|  | Characterizing emotional dysfunction in borderline personality, major depression, and their co-occurrence |
|  | Biomarkers of brain injury following an American football game: A pilot study |
|  | Short-term effects of 17β-estradiol on physiological responses in stellate sturgeon Acipenser stellatus |
|  | Are Cushing's disease patients curable? |
|  | Multidimensional assessment of neuroendocrine and psychopathological profiles in maltreated youth |
|  | Interest of low-dose hydrocortisone therapy during brain-dead organ donor resuscitation: the CORTICOME study |
|  | Sex differences in neurosteroid and hormonal responses to metyrapone in posttraumatic stress disorder |
|  | The influence of Hatha yoga as an add-on treatment in major depression on hypothalamic-pituitary-adrenal-axis activity: a randomized trial |
|  | Depressive symptoms and cortisol variability prior to surgery for suspected endometrial cancer |
|  | Basal blood DHEA-S/cortisol levels predicts EMDR treatment response in adolescents with PTSD |
|  | Acute hormonal findings after aneurysmal subarachnoid hemorrhage - report from a single center |
|  | Exposure to environmental levels of waterborne cadmium impacts corticosteroidogenic and metabolic capacities, and compromises secondary stressor performance in rainbow trout |
|  | Factors influencing alopecia and hair cortisol in rhesus macaques (Macaca mulatta) |
|  | Long-term outcomes of tissue-based ACTH-antibody assay-guided transsphenoidal resection of pituitary adenomas in Cushing disease |
|  | Diurnal cortisol profile in Williams syndrome in novel and familiar settings |
|  | Human Metabolome Changes after a Single Dose of 3,4-Methylenedioxymethamphetamine (MDMA) with Special Focus on Steroid Metabolism and Inflammation Processes |
|  | Circumcision does not alter long-term glucocorticoids accumulation or psychological effects associated with trauma- and stressor-related disorders |
|  | The association between three major physiological stress systems and oxidative DNA and lipid damage |
|  | Does stress affect the joints? Daily stressors, stress vulnerability, immune and HPA axis activity, and short-term disease and symptom fluctuations in rheumatoid arthritis |
|  | A mechanism for rapid neurosteroidal regulation of parenting behaviour |
|  | Metabolite-inactive etomidate analogues alleviating suppression on adrenal function in Beagle dogs |
|  | Maternal prenatal depressive symptoms predict infant NR3C1 1F and BDNF IV DNA methylation |
|  | Gender-specific co-activation of arginine vasopressin and the hypothalamic-pituitary-adrenal axis during stress |
|  | The effect of allostatic load on hypothalamic-pituitary-interrenal (HPI) axis before and after secondary vaccination in Atlantic salmon postsmolts (Salmo salar L.) |
|  | A placebo-controlled trial to investigate the safety and efficacy of Penicillin G/Hydrocortisone in patients with ALS (PHALS trial) |
|  | Stress-induced enhancement of response inhibition depends on mineralocorticoid receptor activation |
|  | Adipokines and cardiovascular disease: A comprehensive review |
|  | Response of the hypothalamic-pituitary-adrenal axis to stimulation tests before and after exercise training in old and young Standardbred mares |
|  | Polygenic risk score of SERPINA6/SERPINA1 associates with diurnal and stress-induced HPA axis activity in children |
|  | Mindfulness-Based Stress Reduction for Older Adults With Stress Disorders and Neurocognitive Difficulties: A Randomized Controlled Trial |
|  | Biomarker response and hypothalamus-pituitary-interrenal axis functioning in Arctic charr from Bjørnøya (74°30' N), Norway, with high levels of organohalogenated compounds |
|  | Early Parenting Intervention - Biobehavioral Outcomes in infants with Neurodevelopmental Disabilities (EPI-BOND): study protocol for an Italian multicentre randomised controlled trial |
|  | A reliable global cognitive decline and cortisol as an associated risk factor for patients with late-life depression in the short term: A 1-year prospective study |
|  | Unique and potent effects of acute ibogaine on zebrafish: the developing utility of novel aquatic models for hallucinogenic drug research |
|  | The role of substance use, smoking, and inflammation in risk for suicidal behavior |
|  | Stress hormones and verbal memory in young people over the first 12 weeks of treatment for psychosis |
|  | Differences in anticipatory versus reactive stress to social evaluative threat in adults versus adolescents with autism |
|  | Effects of thermal stress on the expression of glucocorticoid receptor complex linked genes in Senegalese sole (Solea senegalensis): Acute and adaptive stress responses |
|  | Acute toxicity, biochemical and histopathological responses of endosulfan in Chanos chanos |
|  | Association of in vivo κ-opioid receptor availability and the transdiagnostic dimensional expression of trauma-related psychopathology |
|  | Possible ACTH-independent, cortisol-secreting and DHEA-secreting metastatic hepatocellular carcinoma causing Cushing's syndrome |
|  | Acute effects of coffee consumption on self-reported gastrointestinal symptoms, blood pressure and stress indices in healthy individuals |
|  | Light at night alters daily patterns of cortisol and clock proteins in female Siberian hamsters |
|  | Blunted IL-6 and IL-10 response to maximal aerobic exercise in patients with traumatic brain injury |
|  | Advanced Circadian Phase in Mania and Delayed Circadian Phase in Mixed Mania and Depression Returned to Normal after Treatment of Bipolar Disorder |
|  | Trauma Severity in Early Childhood Correlates with Stress and Satiety Hormone Levels in a Pilot Cohort Receiving Diamorphine Maintenance Treatment |
|  | A comparison of age, cognitive, hormonal, symptomatic and mood correlates of Aggression towards Others in boys with ASD |
|  | Prenatal bisphenol a exposure and dysregulation of infant hypothalamic-pituitary-adrenal axis function: findings from the APrON cohort study |
|  | Associations between maternal socioeconomic, psychosocial and seasonal factors, infant characteristics and human milk cortisol concentrations |
|  | Effect and mechanism of hydrocortisone on organ function in patients with severe burns |
|  | Preoperative normalization of cortisol levels in Cushing's disease after medical treatment: consequences for somatostatin and dopamine receptor subtype expression and in vitro response to somatostatin analogs and dopamine agonists |
|  | A mu-opioid receptor single nucleotide polymorphism in rhesus monkey: association with stress response and aggression |
|  | Ketamine Attenuates the ACTH Response to Hypoxia in Late-Gestation Ovine Fetus |
|  | Glucocorticoids enhance the in vivo migratory response of human monocytes |
|  | Update to the Vitamin C, Thiamine and Steroids in Sepsis (VICTAS) protocol: statistical analysis plan for a prospective, multicenter, double-blind, adaptive sample size, randomized, placebo-controlled, clinical trial |
|  | Sexual selection on male vocal fundamental frequency in humans and other anthropoids |
|  | Assessment of the effect of continuous sedation with mechanical ventilation on adrenal insufficiency in patients with traumatic brain injury |
|  | Interaction between serum BDNF and aerobic fitness predicts recognition memory in healthy young adults |
|  | Omega-3 polyunsaturated fatty acid levels and dysregulations in biological stress systems |
|  | Prospective, randomized, double-blind, placebo-controlled phase IIa clinical trial on the effects of an estrogen-progestin combination as add-on to inpatient psychotherapy in adult female patients suffering from anorexia nervosa |
|  | The dexamethasone corticotropin releasing hormone test in healthy and depressed women with and without childhood adversity |
|  | Cortisol and depression in pre-diagnosed and early stage Huntington's disease |
|  | Repeated intranasal oxytocin administration in early life dysregulates the HPA axis and alters social behavior |
|  | Stress and Depression: a Crucial Role of the Mineralocorticoid Receptor |
|  | Sympathetic activity and hypothalamo-pituitary-adrenal axis activity during sleep in post-traumatic stress disorder: a study assessing polysomnography with simultaneous blood sampling |
|  | Perceived Discrimination, Racial Identity, and Multisystem Stress Response to Social Evaluative Threat Among African American Men and Women |
|  | Short-term effect of ovariectomy on measures of insulin sensitivity and response to dexamethasone administration in horses |
|  | The influence of combined oral contraceptives containing drospirenone on hypothalamic-pituitary-adrenocortical axis activity and glucocorticoid receptor expression and function in women with polycystic ovary syndrome |
|  | Common marmoset (Callithrix jacchus) personality, subjective well-being, hair cortisol level and AVPR1a, OPRM1, and DAT genotypes |
|  | Exhaled nitric oxide and vascular endothelial growth factor as predictors of cold symptoms after stress |
|  | Increased maternal BMI is associated with infant wheezing in early life: a prospective cohort study |
|  | Assessment of Insulin Resistance Among Drug-Naive Patients With First-Episode Schizophrenia in the Context of Hormonal Stress Axis Activation |
|  | Effect of dexamethasone intravitreal implant on blood glucose, hypothalamic-pituitary-adrenal axis function and vascular endothelial growth factor serum levels in patients with diabetic macular oedema |
|  | Relationships between affiliative social behavior and hair cortisol concentrations in semi-free ranging rhesus monkeys |
|  | Neuroendocrine markers and psychological features in patients with irritable bowel syndrome |
|  | Enhanced cholinergic-tone during the stress induce a depressive-like state in mice |
|  | Oral administration of melatonin counteracts several of the effects of chronic stress in rainbow trout |
|  | Neurobiological mechanisms of exercise and psychotherapy in depression: The SPeED study-Rationale, design, and methodological issues |
|  | Nineteen and Up study (19Up): understanding pathways to mental health disorders in young Australian twins |
|  | Coping with unpredictability: dopaminergic and neurotrophic responses to omission of expected reward in Atlantic salmon (Salmo salar L.) |
|  | ACTH, alpha-MSH, and control of cortisol release: cloning, sequencing, and functional expression of the melanocortin-2 and melanocortin-5 receptor in Cyprinus carpio |
|  | The effect of tryptophan on the cortisol response to social stress is modulated by the 5-HTTLPR genotype |
|  | Perceived stress is linked to heightened biomarkers of inflammation via diurnal cortisol in a national sample of adults |
|  | Prevalence, structure and correlates of anxiety-depression in boys with an autism spectrum disorder |
|  | Total and free cortisol responses and their relation to outcomes after cardiopulmonary bypass in infants |
|  | Effects of Mental Fatigue on Endurance Performance in the Heat |
|  | BDNF val(66)met genotype shows distinct associations with the acoustic startle reflex and the cortisol stress response in young adults and children |
|  | Hypothalamic-pituitary-adrenal axis dysfunction and illness progression in bipolar disorder |
|  | Age Effects on Cognitive and Physiological Parameters in Familial Caregivers of Alzheimer's Disease Patients |
|  | Alterations in hypothalamic-pituitary-adrenal function immediately after resection of adrenal adenomas in patients with Cushing's syndrome and others with incidentalomas and subclinical hypercortisolism |
|  | The role of anxiety in cortisol stress response and cortisol recovery in boys with oppositional defiant disorder/conduct disorder |
|  | Maternal distress and hair cortisol in pregnancy among women with elevated adverse childhood experiences |
|  | Maternal prenatal cortisol predicts infant negative emotionality in a sex-dependent manner |
|  | A mindfulness-based stress management program for caregivers of allogeneic hematopoietic stem cell transplant (HCT) patients: Protocol for a randomized controlled trial |
|  | Intergenerational gene × environment interaction of FKBP5 and childhood maltreatment on hair steroids |
|  | An open-label adrenal suppression study of 0.1% fluocinonide cream in pediatric patients with atopic dermatitis |
|  | Potential Biomarkers with Plasma Cortisol, Brain-derived Neurotrophic Factor and Nitrites in Patients with Acute Ischemic Stroke |
|  | The role of suboptimal mitochondrial function in vulnerability to post-traumatic stress disorder |
|  | Primate HPT axis response to the peripheral kisspeptin challenge under different time periods of food restriction in monkeys |
|  | The effect of local injection of methylprednisolone acetate on the hypothalamic-pituitary-adrenal axis among patients with greater trochanteric pain syndrome |
|  | Dose-response inhibitory effects of purified cathinone from khat (Catha edulis) on cortisol and prolactin release in vervet monkeys (Chlorocebus aethiops) |
|  | Chronic systemic administration of serotonergic ligands flibanserin and 8-OH-DPAT enhance HPA axis responses to restraint in female marmosets |
|  | Effects of Carbenoxolone on the Canine Pituitary-Adrenal Axis |
|  | Cortisol response to cosyntropin administration in military veterans with or without posttraumatic stress disorder |
|  | Assessment of the relationship between melatonin, hormones of the pituitary-ovarian, -thyroid and -adrenocortical axes, and osteoprotegerin and its ligand sRANKL in girls with anorexia nervosa |
|  | SKA2 Methylation is Involved in Cortisol Stress Reactivity and Predicts the Development of Post-Traumatic Stress Disorder (PTSD) After Military Deployment |
|  | Lead exposure and fear-potentiated startle in the VA Normative Aging Study: a pilot study of a novel physiological approach to investigating neurotoxicant effects |
|  | Hair Cortisol and Its Association With Psychological Risk Factors for Psychiatric Disorders: A Pilot Study in Adolescent Twins |
|  | Inhibitory effects of β-endorphin on cortisol release from goldfish (Carassius auratus) head kidney: an in vitro study |
|  | Evaluation of Adrenal Function in Nonhospitalized Patients with Cirrhosis |
|  | Seasonal changes in plasma testosterone and cortisol suggest an androgen mediated regulation of the pituitary adrenal axis in the Tarabul's gerbil Gerbillus tarabuli (Thomas, 1902) |
|  | Relationship between vagal tone, cortisol, TNF-alpha, epinephrine and negative affects in Crohn's disease and irritable bowel syndrome |
|  | Adrenocortical Function in Children With Brain Tumors and Pediatric Hematopoietic Cell Transplantation Recipients |
|  | Diurnal cortisol rhythms, fatigue and psychosocial factors in five-year survivors of ovarian cancer |
|  | Effect of Vasopressin on the Hypothalamic-Pituitary-Adrenal Axis in ADPKD Patients during V2 Receptor Antagonism |
|  | Diurnal variation of hypothalamic function and chronic subthalamic nucleus stimulation in Parkinson's disease |
|  | Urinary cortisol and psychopathology in obese binge eating subjects |
|  | Aldosterone synthase inhibition for the treatment of hypertension and the derived mechanistic requirements for a new therapeutic strategy |
|  | Recurrent hypoglycaemia: a delayed presentation of Sheehan syndrome |
|  | Effects of an exercise and hypocaloric healthy eating intervention on indices of psychological health status, hypothalamic-pituitary-adrenal axis regulation and immune function after early-stage breast cancer: a randomised controlled trial |
|  | Sex-Related Characteristics of Systemic Hormonal Homeostasis in Rats with Sarcoma C-45 Cells Transplanted to the Lung |
|  | The association of openness personality trait with stress-related salivary biomarkers in burning mouth syndrome |
|  | Neurobiological and psychological evidence of chronic stress in prostate cancer patients |
|  | A 3-arm randomized controlled trial on the effects of dance movement intervention and exercises on elderly with early dementia |
|  | Acute fasting-induced repression of the hypothalamic-pituitary-gonadal axis is reversed by RF-9 administration in the adult male macaque |
|  | Unusual case of Hashimoto's encephalopathy and pseudo-obstruction in a patient with undiagnosed hypothyroidism: a case report |
|  | Association of testosterone and BDNF serum levels with craving during alcohol withdrawal |
|  | Microbial modulation of behavior and stress responses in zebrafish larvae |
|  | Stress improves task processing efficiency in dual-tasks |
|  | Proteomic changes in serum of first onset, antidepressant drug-naïve major depression patients |
|  | Voluntary running influences the efficacy of fluoxetine in a model of postpartum depression |
|  | The effect of epidural methylprednisolone acetate injection on the hypothalamic-pituitary-adrenal axis |
|  | Mortality data from the European Adrenal Insufficiency Registry-Patient characterization and associations |
|  | Regulation of the corticosteroid signalling system in rainbow trout HPI axis during confinement stress |
|  | Acute effect of HIIT on testosterone and cortisol levels in healthy individuals: A systematic review and meta-analysis |
|  | Telmisartan attenuates diabetes induced depression in rats |
|  | Anti-Stress, Behavioural and Magnetoencephalography Effects of an L-Theanine-Based Nutrient Drink: A Randomised, Double-Blind, Placebo-Controlled, Crossover Trial |
|  | Salivary neurosteroid levels and behavioural profiles of children with attention-deficit/hyperactivity disorder during six months of methylphenidate treatment |
|  | Thyroid axis activity and dopamine function in depression |
|  | Primary Empty Sella Syndrome and the Prevalence of Hormonal Dysregulation |
|  | The prevalence of impaired glucose regulation in anxiety disorder patients and the relationship with hypothalamic-pituitary-adrenal axis and hypothalamic-pituitary-thyroid axis activity |
|  | The presence of symptoms of testosterone deficiency in the exercise-hypogonadal male condition and the role of nutrition |
|  | Evidence for Immune Activation and Resistance to Glucocorticoids Following Childhood Maltreatment in Adolescents Without Psychopathology |
|  | Deconvolution of serum cortisol levels by using compressed sensing |
|  | Intra-articular methylprednisolone acetate injection at the knee joint and the hypothalamic-pituitary-adrenal axis: a randomized controlled study |
|  | Effects of seasonal differences in testosterone and cortisol levels on pain responses under resting and anxiety conditions |
|  | Basal salivary cortisol secretion and susceptibility to upper respiratory infection |
|  | Attention-deficit/hyperactivity disorder symptoms and stress-related biomarkers |
|  | Effect of alpine grazing on plasma and hair cortisol, serotonin, and DHEA in dairy cows and its welfare impact |
|  | The safety and efficacy of short-term budesonide delivered via mucosal atomization device for chronic rhinosinusitis without nasal polyposis |
|  | Daily rhythms in the hypothalamus-pituitary-interrenal axis and acute stress responses in a teleost flatfish, Solea senegalensis |
|  | The association between alcohol abuse and neuroendocrine system dysregulation: Race differences in a National sample |
|  | Clinicopathological predictive factors in the early remission of corticotroph pituitary macroadenomas in a tertiary referral centre |
|  | Associations between cytokines, endocrine stress response, and gastrointestinal symptoms in autism spectrum disorder |
|  | Morning Serum Cortisol Is Uniquely Associated with Cardiometabolic Risk Independent of Body Composition in Latino Adolescents |
|  | Catechol-O-methyltransferase Val158Met polymorphism associates with affect and cortisol levels in women |
|  | Neonatal gut and immune maturation is determined more by postnatal age than by postconceptional age in moderately preterm pigs |
|  | Stress matters! Psychophysiological and emotional loadings of pregnant women undergoing fetal magnetic resonance imaging |
|  | Markers of physiological stress during exercise under conditions of normoxia, normobaric hypoxia, hypobaric hypoxia, and genuine high altitude |
|  | Probiotic Lactobacillus plantarum P8 alleviated stress and anxiety while enhancing memory and cognition in stressed adults: A randomised, double-blind, placebo-controlled study |
|  | Response and habituation of pro- and anti-inflammatory gene expression to repeated acute stress |
|  | Subclinical Cushing syndrome associated with an empty sella turcica |
|  | Cortisol and inflammatory processes in ovarian cancer patients following primary treatment: relationships with depression, fatigue, and disability |
|  | Effects of mineralocorticoid-receptor stimulation on risk taking behavior in young healthy men and women |
|  | Management of hyperglycaemia in Cushing's disease: experts' proposals on the use of pasireotide |
|  | HPA axis genetic variation, cortisol and psychosis in major depression |
|  | Adiposity moderates links from early adversity and depressive symptoms to inflammatory reactivity to acute stress during late adolescence |
|  | The misleading nature of in vitro and ex vivo findings in studying the impact of stress hormones on NK cell cytotoxicity |
|  | Workplace based mindfulness practice and inflammation: a randomized trial |
|  | Steroid 21-hydroxylase deficiency in congenital adrenal hyperplasia |
|  | BDNF, interleukin-6, and salivary cortisol levels in depressed patients treated with desvenlafaxine |
|  | Corticosteroids for managing tuberculous meningitis |
|  | Plantar stimulation in parkinsonians: From biomarkers to mobility - randomized-controlled trial |
|  | MMP9 mRNA is a potential diagnostic and treatment monitoring marker for PTSD: Evidence from mice and humans |
|  | Monocytes from depressed patients display an altered pattern of response to endotoxin challenge |
|  | Low-dose oxytocin delivered intranasally with Breath Powered device affects social-cognitive behavior: a randomized four-way crossover trial with nasal cavity dimension assessment |
|  | Duress without stress: Cryptobia infection results in HPI axis dysfunction in rainbow trout |
|  | Plasma lipoproteins in posttraumatic stress disorder patients compared to healthy controls and their associations with the HPA- and HPT-axis |
|  | Relationship of cortisol levels and genetic polymorphisms to antidepressant response to placebo and fluoxetine in patients with major depressive disorder: a prospective study |
|  | Delayed retinal vein recovery responses indicate both non-adaptation to stress as well as increased risk for stroke: the SABPA study |
|  | The chronic effects of a combination of herbal extracts (Euphytose(®)) on psychological mood state and response to a laboratory stressor: A randomised, placebo-controlled, double blind study in healthy humans |
|  | Effects of pre-experience of social exclusion on hypothalamus-pituitary-adrenal axis and catecholaminergic responsiveness to public speaking stress |
|  | Exercise capacity is not impaired after acute alcohol ingestion: a pilot study |
|  | Glutamate receptors and the regulation of steroidogenesis in the human adrenal gland: the metabotropic pathway |
|  | Effect of constant, predictable, and unpredictable motor tasks on motor performance and blood markers of stress |
|  | Airway inflammation and hypothalamic-pituitary-adrenal axis activity in asthmatic adults with depression |
|  | Dairy food consumption and meal-induced cortisol response interacted to influence weight loss in overweight women undergoing a 12-week, meal-controlled, weight loss intervention |
|  | Safety analysis of long-term budesonide nasal irrigations in patients with chronic rhinosinusitis post endoscopic sinus surgery |
|  | Association between hyperinsulinaemia and laminitis severity at the time of pituitary pars intermedia dysfunction diagnosis |
|  | Targeting hypothalamic-pituitary-adrenal axis hormones and sex steroids for improving cognition in major mood disorders and schizophrenia: a systematic review and narrative synthesis |
|  | Effects of bimagrumab, an activin receptor type II inhibitor, on pituitary neurohormonal axes |
|  | Effects of the Synthetic Neurosteroid: 3β-Methoxypregnenolone (MAP4343) on Behavioral and Physiological Alterations Provoked by Chronic Psychosocial Stress in Tree Shrews |
|  | Reduced hypothalamic-pituitary-adrenal axis activity in chronic multi-site musculoskeletal pain: partly masked by depressive and anxiety disorders |
|  | The biological impact of listening to music in clinical and nonclinical settings: A systematic review |
|  | Sleep deprivation attenuates endotoxin-induced cytokine gene expression independent of day length and circulating cortisol in male Siberian hamsters (Phodopus sungorus) |
|  | Hypothalamo-pituitary-adrenal axis, glucose metabolism and TNF-α in narcolepsy |
|  | Biological markers in noninvasive brain stimulation trials in major depressive disorder: a systematic review |
|  | Effects of web-based cognitive behavioral stress management and health promotion interventions on neuroendocrine and inflammatory markers in men with advanced prostate cancer: A randomized controlled trial |
|  | Changes in energy metabolism, and levels of stress-related hormones and electrolytes in horses after intravenous administration of romifidine and the peripheral α-2 adrenoceptor antagonist vatinoxan |
|  | Aldosterone secretion in patients with septic shock: a prospective study |
|  | Effect of Vitamin C, Thiamine, and Hydrocortisone on Ventilator- and Vasopressor-Free Days in Patients With Sepsis: The VICTAS Randomized Clinical Trial |
|  | Lasting Adaptations in Social Behavior Produced by Social Disruption and Inhibition of Adult Neurogenesis |
|  | Evaluation of the influence of prenatal transportation stress on GnRH-stimulated luteinizing hormone and testosterone secretion in sexually mature Brahman bulls |
|  | Copeptin is associated with mortality in elderly people |
|  | Direction of post-prandial ghrelin response associated with cortisol response, perceived stress and anxiety, and self-reported coping and hunger in obese women |
|  | Acute stress alters autonomic modulation during sleep in women approaching menopause |
|  | Change in urinary cortisol excretion mediates the effect of angry/hostile mood on 9 month diastolic blood pressure in HIV+ adults |
|  | Sex steroid modulation of cortisol secretion in sheep |
|  | The prognostic value of perioperative profiles of ACTH and cortisol for recurrence after transsphenoidal hypophysectomy in dogs with corticotroph adenomas |
|  | Acute neuro-endocrine profile and prediction of outcome after severe brain injury |
|  | Neuroactive Steroids in Acute Ischemic Stroke: Association with Cognitive, Functional, and Neurological Outcomes |
|  | Rise of ketone bodies with psychosocial stress in normal weight men |
|  | Inhaled fluticasone furoate/vilanterol does not affect hypothalamic-pituitary-adrenal axis function in adolescent and adult asthma: randomised, double-blind, placebo-controlled study |
|  | Influence of the hypothalamic-pituitary-adrenal axis dysregulation on the metabolic profile of patients affected by diabetes mellitus-associated late onset hypogonadism |
|  | Altered Acoustic Startle Reflex, Prepulse Inhibition, and Peripheral Brain-Derived Neurotrophic Factor in Morphine Self-Administered Rats |
|  | Toll-like receptor 4 and comorbid pain in Interstitial Cystitis/Bladder Pain Syndrome: a multidisciplinary approach to the study of chronic pelvic pain research network study |
|  | Hypothalamic-pituitary-adrenal axis activity is associated with the prevalence of chronic kidney disease in diabetic patients |
|  | A variant of the neuronal amino acid transporter SLC6A15 is associated with ACTH and cortisol responses and cognitive performance in unipolar depression |
|  | Genome-wide DNA methylation levels and altered cortisol stress reactivity following childhood trauma in humans |
|  | Stress hormones at rest and following exercise testing predict coronary artery disease severity and outcome |
|  | Arginine vasotocin treatment induces a stress response and exerts a potent anorexigenic effect in rainbow trout, Oncorhynchus mykiss |
|  | The stress response to surgery and postoperative delirium: evidence of hypothalamic-pituitary-adrenal axis hyperresponsiveness and decreased suppression of the GH/IGF-1 Axis |
|  | Role for the kinase SGK1 in stress, depression, and glucocorticoid effects on hippocampal neurogenesis |
|  | The prevalence of impaired glucose regulation in psychiatric patients with sleep disorders and its relationship with altered hypothalamopituitary-adrenal and hypothalamopituitary-thyroid axis activity |
|  | Short intense psychological stress induced by skydiving does not impair intestinal barrier function |
|  | Evaluation of activity of hypothalamo-pituitary-gonadal axis in postmenopausal women suffering from severe acute illness |
|  | A method for conducting functional MRI studies in alert nonhuman primates: initial results with opioid agonists in male cynomolgus monkeys |
|  | Moderate aerobic training modulates cytokines and cortisol profiles in older adults with cognitive abilities |
|  | Congenital Hypopituitarism |
|  | Revised GH and cortisol cut-points for the glucagon stimulation test in the evaluation of GH and hypothalamic-pituitary-adrenal axes in adults: results from a prospective randomized multicenter study |
|  | Pituitary dysfunction after traumatic brain injury in children: is there a need for ongoing endocrine assessment? |
|  | Endoscopic transsphenoidal pituitary surgery: a good and safe primary treatment option for Cushing's disease, even in case of macroadenomas or invasive adenomas |
|  | Catecholamines and Paroxysmal Sympathetic Hyperactivity after Traumatic Brain Injury |
|  | Effects of leukemia inhibitory receptor gene mutations on human hypothalamo-pituitary-adrenal function |
|  | Dynamic changes of central thyroid functions in the management of Cushing's syndrome |
|  | Cognitive Performance and the Alteration of Neuroendocrine Hormones in Chronic Tension-Type Headache |
|  | Endocrine disorders in women with complex regional pain syndrome type I |
|  | Rosemary Tea Consumption Alters Peripheral Anxiety and Depression Biomarkers: A Pilot Study in Limited Healthy Volunteers |
|  | Increased cortisol relative to adrenocorticotropic hormone predicts improvement during anti-tumor necrosis factor therapy in rheumatoid arthritis |
|  | Selective use of the insulin tolerance test to diagnose hypopituitarism |
|  | Bidirectional psychoneuroimmune interactions in the early postpartum period influence risk of postpartum depression |
|  | Changes in pain perception and hormones pre- and post-kumdo competition |
|  | The Cortisol and ACTH Response to Dex/CRH Testing in Women With and Without Perimenopausal Depression |
|  | Relative hypocortisolism is associated with obesity and the metabolic syndrome in recurrent affective disorders |
|  | The role of mineralocorticoid receptor function in treatment-resistant depression |
|  | Dynamics of postoperative serum cortisol after transsphenoidal surgery for Cushing's disease: implications for immediate reoperation and remission |
|  | Abuse, nocturnal stress hormones, and coronary heart disease risk among women with HIV |
|  | Associations between immunological function and memory recall in healthy adults |
|  | Relationship between plasma analytes and SPARE-AD defined brain atrophy patterns in ADNI |
|  | Relation of progesterone and DHEAS serum levels to 5-HT1A receptor binding potential in pre- and postmenopausal women |
|  | Trait reflection predicts interleukin-6 response to a social-evaluative stressor |
|  | Alterations in hypothalamus-pituitary-adrenal/thyroid axes and gonadotropin-releasing hormone in the patients with primary insomnia: a clinical research |
|  | A Single Bout of High-Intensity Interval Training Reduces Awareness of Subsequent Hypoglycemia in Patients With Type 1 Diabetes |
|  | A naturally hypersensitive glucocorticoid receptor elicits a compensatory reduction of hypothalamus-pituitary-adrenal axis activity early in ontogeny |
|  | Effects of a 6-week, whole-body vibration strength-training on depression symptoms, endocrinological and neurobiological parameters in adolescent inpatients experiencing a major depressive episode (the "Balancing Vibrations Study"): study protocol for a randomized placebo-controlled trial |
|  | Sex-specific associations of basal steroid hormones and neuropeptides with Conduct Disorder and neuroendocrine mediation of environmental risk |
|  | Higher serum DHEA concentrations before and after SSRI treatment are associated with remission of major depression |
|  | Stress and Obesity: Are There More Susceptible Individuals? |
|  | Repeating patterns of sleep restriction and recovery: Do we get used to it? |
|  | Impaired leukocyte trafficking and skin inflammatory responses in hamsters lacking a functional circadian system |
|  | Pathophysiology of major depressive disorder: mechanisms involved in etiology are not associated with clinical progression |
|  | Renin-Angiotensin-aldosterone system and hypothalamic-pituitary-adrenal axis in hospitalized newborn foals |
|  | Stress biomarkers in minimally invasive and conventional colorectal resections |
|  | Evaluation of the association between placental corticotrophin-releasing hormone and postpartum depressive symptoms |
|  | Steroids, steroid precursors, and neuroactive steroids in critically ill equine neonates |
|  | Mineralocorticoid receptor stimulation effects on spatial memory in healthy young adults: A study using the virtual Morris Water Maze task |
|  | Psychological and Physiological Markers of Stress in Concussed Athletes Across Recovery Milestones |
|  | Effect of sub chronic tryptophan supplementation on stress-induced cortisol and appetite in subjects differing in 5-HTTLPR genotype and trait neuroticism |
|  | Changes in plasma ACTH levels and corticotroph tumor size in patients with Cushing's disease during long-term treatment with the glucocorticoid receptor antagonist mifepristone |
|  | Estradiol, but not testosterone, heightens cortisol-mediated negative feedback on pulsatile ACTH secretion and ACTH approximate entropy in unstressed older men and women |
|  | Partial cloning of CB1 cDNA and CB1 mRNA changes in stress responses in the Solea solea |
|  | Prognostic significance of hypothalamic-pituitary-adrenal axis hormones in early sepsis: a study performed in the emergency department |
|  | Immune and endocrine function in patients with burning mouth syndrome |
|  | Sleep apnoea and the hypothalamic-pituitary-adrenal axis in men and women: effects of continuous positive airway pressure |
|  | Control of sleep and wakefulness in health and disease |
|  | The impact of peri-operative dexamethasone administration on the normal hypothalamic pituitary adrenal response to major surgical procedures |
|  | Placebo-controlled dietary intervention of stress-induced neurovegetative disorders with a specific amino acid composition: a pilot-study |
|  | The effects of repetitive transcranial magnetic stimulation in obese females with binge eating disorder: a protocol for a double-blinded, randomized, sham-controlled trial |
|  | Examination of Org 26576, an AMPA receptor positive allosteric modulator, in patients diagnosed with major depressive disorder: an exploratory, randomized, double-blind, placebo-controlled trial |
|  | Acute aseptic meningitis as the initial presentation of a macroprolactinoma |
|  | Variable neuroendocrine-immune dysfunction in individuals with unfavorable outcome after severe traumatic brain injury |
|  | Prenatal maternal anxiety predicts reduced adaptive immunity in infants |
|  | Prognostic value of dehydroepiandrosterone-sulfate and other parameters of adrenal function in acute ischemic stroke |
|  | Mutual influences between partners' hormones shape conflict dialog and relationship duration at the initiation of romantic love |
|  | Alexithymia is linked to neurocognitive, psychological, neuroendocrine, and immune dysfunction in persons living with HIV |
|  | Effects of fludrocortisone on water and sodium intake of C57BL/6 mice |
|  | Sleep characteristics as predictor variables of stress systems markers in insomnia disorder |
|  | Blocking metabotropic glutamate receptor subtype 5 relieves maladaptive chronic stress consequences |
|  | Serum levels of brain-derived neurotrophic factor and cortisol to sulfate of dehydroepiandrosterone molar ratio associated with clinical response to L-theanine as augmentation of antipsychotic therapy in schizophrenia and schizoaffective disorder patients |
|  | ApoE2 Exaggerates PTSD-Related Behavioral, Cognitive, and Neuroendocrine Alterations |
|  | Inhibitory effects of Aconiti Lateralis Radix Preparata on chronic intermittent cold-induced inflammation in the mouse hypothalamus |
|  | Decreased maternal hypothalamic-pituitary-adrenal axis activity in very severely obese pregnancy: Associations with birthweight and gestation at delivery |
|  | Severe loss-of-function mutations in the adrenocorticotropin receptor (ACTHR, MC2R) can be found in patients diagnosed with salt-losing adrenal hypoplasia |
|  | Allele-specific FKBP5 DNA demethylation mediates gene-childhood trauma interactions |
|  | Crowding stress inhibits serotonin 1A receptor-mediated increases in corticotropin-releasing factor mRNA expression and adrenocorticotropin hormone secretion in the Gulf toadfish |
|  | Influence of sleep deprivation and circadian misalignment on cortisol, inflammatory markers, and cytokine balance |
|  | Effect of SOM230 (pasireotide) on corticotropic cells: action in dogs with Cushing's disease |
|  | Glucose homeostasis in major depression and schizophrenia: a comparison among drug-naïve first-episode patients |
|  | Transferring the blues: Depression-associated gut microbiota induces neurobehavioural changes in the rat |
|  | Nocturnal Gamma-Hydroxybutyrate Reduces Cortisol-Awakening Response and Morning Kynurenine Pathway Metabolites in Healthy Volunteers |
|  | Increased irritability, anxiety, and immune reactivity in transgenic Huntington's disease monkeys |
|  | Oxytocin reduces reward-driven food intake in humans |
|  | Black porgy (Acanthopagrus schlegeli) prolactin cDNA sequence: mRNA expression and blood physiological responses during freshwater acclimation |
|  | Short-term treatment with the calcineurin inhibitor cyclosporine A decreases HPA axis activity and plasma noradrenaline levels in healthy male volunteers |
|  | A novel biomarker associated with distress in humans: calcium-binding protein, spermatid-specific 1 (CABS1) |
|  | The influence of FKBP5 genotype on expression of FKBP5 and other glucocorticoid-regulated genes, dependent on trauma exposure |
|  | Neuroendocrine function and associated mental health outcomes following mild traumatic brain injury in OEF-deployed service members |
|  | Assessment of low-dose cisplatin as a model of nausea and emesis in beagle dogs, potential for repeated administration |
|  | Effects of Insufficient Sleep on Pituitary-Adrenocortical Response to CRH Stimulation in Healthy Men |
|  | Genetic variation in FKBP5 associated with the extent of stress hormone dysregulation in major depression |
|  | Placental FKBP5 genetic and epigenetic variation is associated with infant neurobehavioral outcomes in the RICHS cohort |
|  | Improved Method for the Establishment of an In Vitro Blood-Brain Barrier Model Based on Porcine Brain Endothelial Cells |
|  | Intranasal angiotensin II in humans reduces blood pressure when angiotensin II type 1 receptors are blocked |
|  | Effect of Etomidate vs Propofol for Total Intravenous Anesthesia on Major Postoperative Complications in Older Patients: A Randomized Clinical Trial |
|  | B-type natriuretic peptide increases cortisol and catecholamine concentrations in healthy subjects |
|  | Glucocorticoid inhibition of leptin- and lipopolysaccharide-induced interleukin-6 production in obesity |
|  | The Role of Nutrients in Protecting Mitochondrial Function and Neurotransmitter Signaling: Implications for the Treatment of Depression, PTSD, and Suicidal Behaviors |
|  | A comparison of mindfulness-based stress reduction and an active control in modulation of neurogenic inflammation |
|  | Changes in stress hormones and metabolism during a 105-day simulated Mars mission |
|  | Oral contraceptives may alter the detection of emotions in facial expressions |
|  | The CRF1 Antagonist Verucerfont in Anxious Alcohol-Dependent Women: Translation of Neuroendocrine, But not of Anti-Craving Effects |
|  | Effects of inhibition of gonadotropin releasing hormone secretion on the response to novel objects in young male and female sheep |
|  | Life stress as a risk factor for sustained anxiety and cortisol dysregulation during the first year of survivorship in ovarian cancer |
|  | Early development of endocrine and metabolic consequences after treatment of central nervous system tumors in children |
|  | Screening for hypopituitarism in 509 patients with traumatic brain injury or subarachnoid hemorrhage |
|  | Pineal hypoplasia, reduced melatonin and sleep disturbance in patients with PAX6 haploinsufficiency |
|  | The effects of exogenous progesterone on drug craving and stress arousal in cocaine dependence: impact of gender and cue type |
|  | Spironolactone for poorly controlled hypertension in type 2 diabetes: conflicting effects on blood pressure, endothelial function, glycaemic control and hormonal profiles |
|  | Covariation between plasma phosphate and daytime cortisol in early Parkinson's disease |
|  | Reductions in circulating endocannabinoid levels in individuals with post-traumatic stress disorder following exposure to the World Trade Center attacks |
|  | Association between obesity-related biomarkers and cognitive and motor development in infants |
|  | Acute psychological stress induces short-term variable immune response |
|  | Agomelatine in the tree shrew model of depression: effects on stress-induced nocturnal hyperthermia and hormonal status |
|  | Relapsing, remitting hypercortisolism in Cushing's disease due to intratumoral hemorrhages in pituitary microadenoma |
|  | Assessment of cognitive function across pregnancy using CANTAB: a longitudinal study |
|  | Utilization of machine learning for prediction of post-traumatic stress: a re-examination of cortisol in the prediction and pathways to non-remitting PTSD |
|  | Hypothalamic-pituitary-adrenal axis effects of mometasone furoate/formoterol fumarate vs fluticasone propionate/salmeterol administered through metered-dose inhaler |
|  | Supplementation with macular carotenoids reduces psychological stress, serum cortisol, and sub-optimal symptoms of physical and emotional health in young adults |
|  | Cortisol is an associated-risk factor of brain dysfunction in patients with severe sepsis and septic shock |
|  | Aldosterone and cortisol predict medium-term left ventricular remodelling following myocardial infarction |
|  | Prospects for the pharmacological prevention of post-traumatic stress in vulnerable individuals |
|  | Ectopic ACTH-secreting syndrome: a single-center experience |
|  | Stroke-induced chronic systolic dysfunction driven by sympathetic overactivity |
|  | Pituitary tumor apoplexy associated with extrapontine myelinolysis during pregnancy: A case report |
|  | Altered Stress-Induced Regulation of Genes in Monocytes in Adults with a History of Childhood Adversity |
|  | A case of ectopic ACTH syndrome due to DDAVP-sensitive but V1b receptor-negative bronchial typical carcinoid with lymphatic metastasis and plasma ProGRP elevation |
|  | Hypopituitarism |
|  | Individual responses in biomarkers of health after marathon and half-marathon running: is age a factor in troponin changes? |
|  | Endocrine biomarkers and symptom clusters during the menopausal transition and early postmenopause: observations from the Seattle Midlife Women's Health Study |
|  | A Metabolic Study of Huntington's Disease |
|  | Immune suppression of IgG response against dairy proteins in major depression |
|  | Adrenocorticotropic hormone elicits gonadotropin secretion in premenopausal women |
|  | Extrapontine Myelinolysis and Reversible Parkinsonism After Hyponatremia Correction in a Case of Pituitary Adenoma: Hypopituitarism as a Predisposition for Osmotic Demyelination |
|  | A novel karyopherin-beta homolog is developmentally and hormonally regulated in fetal lung |
|  | Identifying blood-brain-barrier selective single-chain antibody fragments |
|  | Deaths among adult patients with hypopituitarism: hypocortisolism during acute stress, and de novo malignant brain tumors contribute to an increased mortality |
|  | Dark chocolate attenuates intracellular pro-inflammatory reactivity to acute psychosocial stress in men: A randomized controlled trial |
|  | Serum brain-derived neurotrophic factor (BDNF) across pregnancy and postpartum: Associations with race, depressive symptoms, and low birth weight |
|  | Role of hormonal levels on hospital mortality for male patients with severe traumatic brain injury |
|  | Altered DNA methylation of glucose transporter 1 and glucose transporter 4 in patients with major depressive disorder |
|  | Cortisol:brain-derived neurotrophic factor ratio associated with silent ischaemia in a black male cohort: the SA BPA study |
|  | Neuroendocrine Inflammatory Responses in Overweight/Obese Infants |
|  | Synthesis and deposition of basement membrane proteins by primary brain capillary endothelial cells in a murine model of the blood-brain barrier |
|  | Increased serum level of high sensitivity troponin T even prior to surgery can predict adverse events during carotid endarterectomy |
|  | Cytokine inhibition in chronic fatigue syndrome patients: study protocol for a randomized controlled trial |
|  | Hyponatremia following mild/moderate subarachnoid hemorrhage is due to SIAD and glucocorticoid deficiency and not cerebral salt wasting |
|  | Expression of miR-18a and miR-34c in circulating monocytes associated with vulnerability to type 2 diabetes mellitus and insulin resistance |
|  | Correlation of baseline hormonal disorders with immunological failure and mortality in male HIV patients during follow-up |
|  | Appetite changes reveal depression subgroups with distinct endocrine, metabolic, and immune states |
|  | Rapid disease progression in a patient with mismatch repair-deficient and cortisol secreting adrenocortical carcinoma treated with pembrolizumab |
|  | Selective attention to emotional cues and emotion recognition in healthy subjects: the role of mineralocorticoid receptor stimulation |
|  | The impact of acute stress on hormones and cytokines, and how their recovery is affected by music-evoked positive mood |
|  | Multiple latent viruses reactivate in astronauts during Space Shuttle missions |
|  | Hypothalamic pituitary dysfunction amongst nasopharyngeal cancer survivors |
|  | Cerebral salt-wasting syndrome in a child with Wernicke encephalopathy treated with fludrocortisone therapy: A case report |
|  | Blood-brain barrier models and their relevance for a successful development of CNS drug delivery systems: a review |
|  | Perceived discrimination is associated with the inflammatory response to acute laboratory stress in women at risk for cardiovascular disease |
|  | Inflammation-induced hyperalgesia: effects of timing, dosage, and negative affect on somatic pain sensitivity in human experimental endotoxemia |
|  | Early morning cortisol levels as predictors of short-term and long-term adrenal function after endonasal transsphenoidal surgery for pituitary adenomas and Rathke's cleft cysts |
|  | Effects of acute glucocorticoid blockade on metabolic dysfunction in patients with Type 2 diabetes with and without fatty liver |
|  | Sex steroid hormones and sex hormone binding globulin levels, CYP17 MSP AI (-34T:C) and CYP19 codon 39 (Trp:Arg) variants in children with developmental stuttering |
|  | Infection of a Rathke cleft cyst: a rare cause of pituitary abscess |
|  | Cerebral Salt Wasting Is the Most Common Cause of Hyponatremia in Stroke |
|  | Glycocalyx degradation leads to blood-brain barrier dysfunction and brain edema after asphyxia cardiac arrest in rats |
|  | Primary hypothalamic lymphoma with clinical findings mimicking pituitary apoplexy: a case report |
|  | Fluctuation in Serum Sodium Levels Related to Ipragliflozin Administration in a Patient with Diabetic Nephropathy and Sequela of Traumatic Brain Injury |
|  | Longitudinal behavior of autoimmune GH deficiency: from childhood to transition age |
|  | Hypothalamic germinoma masquerading as superior mesenteric artery (SMA) syndrome |
|  | Discrimination exposure and DNA methylation of stress-related genes in Latina mothers |
|  | Stress, memory, and the hippocampus |
|  | A rare association between Rathke's cyst and hypophysitis in a patient with delayed sex development and growth failure |
|  | Reset osmostat: a rare cause of hyponatraemia |
|  | Reversible Non-parkinsonian Bradykinesia with Impaired Frontal Lobe Function as the Predominant Manifestation of Adrenal Insufficiency |
|  | Selective attention to emotional stimuli and emotion recognition in patients with major depression: The role of mineralocorticoid and glutamatergic NMDA receptors |
|  | Hormonal changes following a low-salt diet in patients with Ménière's disease |
|  | Adrenal Insufficiency Caused by Chronic Corticosteroid Use, Identified through Medication Therapy Management |
|  | A novel hook-shaped enhancement on contrast-enhanced sagittal magnetic resonance image in acute Sheehan's syndrome: a case report |
|  | EVALUATION AND MANAGEMENT OF ADRENAL INSUFFICIENCY IN CRITICALLY ILL PATIENTS: DISEASE STATE REVIEW |
|  | Diagnosis and treatment of hyponatraemia in neurosurgical patients |
|  | Glucose metabolism, gut-brain hormones, and acromegaly treatment: an explorative single centre descriptive analysis |
|  | Naringin ameliorates memory deficits and exerts neuroprotective effects in a mouse model of Alzheimer's disease by regulating multiple metabolic pathways |
|  | Evidence for adverse effect of perinatal glucocorticoid use on the developing brain |
|  | Addison's disease concomitant with corticotropin deficiency and pituitary CRH resistance - a case report |
|  | Low-dose corticosteroid therapy for cardiogenic shock in adults (COCCA): study protocol for a randomized controlled trial |
|  | Gonadotroph adenoma with secondary hypersecretion of testosterone |
|  | Anterior hypopituitarism in a patient with amyloidosis secondary to Crohn's disease: a case report |
|  | Hyponatremia in Traumatic Brain Injury: A Practical Management Protocol |
|  | Effect of Noni on Memory Impairment Induced by Hydrocortisone in Mice |
|  | Clinically Mild Encephalitis/Encephalopathy With a Reversible Splenial Lesion Accompanied by Epstein-Barr Virus Hemophagocytic Lymphohistiocytosis: A Case Report and Review of the Literature |
|  | Neuroendocrine Dysfunction in the Acute Setting of Penetrating Brain Injury: A Systematic Review |
|  | Evaluation of the diagnostic criteria for Cushing's disease in Japan |
|  | Myxedema Coma Secondary to Central Hypothyroidism: A Rare but Real Cause of Altered Mental Status in Pediatrics |
|  | Hydrocortisone 21-hemisuccinate did not prevent exogenous GAPDH-induced apoptosis in human neuroblastoma cells |
|  | Hydrocortisone enhances the barrier properties of HBMEC/ciβ, a brain microvascular endothelial cell line, through mesenchymal-to-endothelial transition-like effects |
|  | Role of Endogenous and Exogenous Corticosterone on Behavioral and Cognitive Responses to Low-Pressure Blast Wave Exposure |
|  | Healthy baby delivered vaginally from a brain-dead mother |
|  | Postnatal Corticosteroids to Prevent or Treat Bronchopulmonary Dysplasia |
|  | Central adrenal insufficiency following traumatic brain injury: a missed diagnosis in the critically injured |
|  | Brain white matter abnormality in a newborn infant with congenital adrenal hyperplasia |
|  | Operative care and surveillance in severe trauma patients. Interference between resuscitation treatments and anaesthesiology, and consequence on immunity |
|  | Varicella causing remission of Cushing's disease |
|  | Synchronous pituitary and pineal gland lesions presenting with panhypopituitarism in a patient with widespread colorectal cancer: a case report |
|  | Primary pituitary abscess in an adolescent female patient: case report, literature review, and operative video |
|  | Dexamethasone Downregulates Endothelin Receptors and Reduces Endothelin-Induced Production of Matrix Metalloproteinases in Cultured Rat Astrocytes |
|  | Pituitary Abscess with Unusual Clinical Course |
|  | Context modulates outcome of perinatal glucocorticoid action in the brain |
|  | GAPDH-targeted therapy - A new approach for secondary damage after traumatic brain injury on rats |
|  | Association between ABCB1 Polymorphisms and Antidepressant Treatment Response in Taiwanese Major Depressive Patients |
|  | Pituitary function within the first year after traumatic brain injury or subarachnoid haemorrhage |
|  | Use of endocrinological and neurological medication among 5-year survivors of young onset brain tumors |
|  | Successful treatment of recurrent CNS disease post-bone marrow transplant in children with familial hemophagocytic lymphohistiocytosis |
|  | Glucocorticoids promote neural progenitor cell proliferation derived from human induced pluripotent stem cells |
|  | Simultaneous occurrence of diabetic ketoacidosis, thyroid storm, and multiple cerebral infarctions due to Moyamoya disease |
|  | Effects of glucocorticoid and noradrenergic activity on implicit and explicit facial emotion recognition in healthy young men |
|  | Dexamethasone-related adrenal insufficiency in patients with brain and skull base tumours |
|  | Septo-optic dysplasia/de Morsier's syndrome |
|  | Cerebral vasoconstriction triggered by sympathomimetic drugs during intra-atrerial chemotherapy |
|  | Septooptic Dysplasia with an Associated Arachnoid Cyst |
|  | Comparison of intraoperative cortisol levels after preoperative hydrocortisone administration versus placebo in patients without adrenal insufficiency undergoing endoscopic transsphenoidal removal of nonfunctioning pituitary adenomas: a double-blind randomized trial |
|  | HORMONE SUBSTITUTION AFTER GASTRIC BYPASS SURGERY IN PATIENTS WITH HYPOPITUITARISM SECONDARY TO CRANIOPHARYNGIOMA |
|  | Enhanced brain expression of genes related to cell proliferation and neural differentiation is associated with cortisol receptor expression in fishes |
|  | Perioperative management of Fontan operation for the child with panhypopituitarism: a case report |
|  | Severe hyponatremia caused by secondary adrenal insufficiency in a patient with giant pituitary prolactinoma |
|  | A newborn with combined pituitary hormone deficiency developing shock and sludge |
|  | Bevacizumab for symptomatic radiation-induced tumor enlargement in pediatric low grade gliomas |
|  | Application of the amniotic fluid metabolome to the study of fetal malformations, using Down syndrome as a specific model |
|  | Case report of neonatal ductus venosus atresia |
|  | Glucocorticoid-induced hyperglycemia is prevalent and unpredictable for patients undergoing cancer therapy: an observational cohort study |
|  | Natural product HTP screening for antibacterial (E.coli 0157:H7) and anti-inflammatory agents in (LPS from E. coli O111:B4) activated macrophages and microglial cells; focus on sepsis |
|  | Management of Cushing syndrome in children and adolescents: experience of a single tertiary centre |
|  | Intrathecal methotrexate prophylaxis and central nervous system relapse in patients with diffuse large B-cell lymphoma following rituximab plus cyclophosphamide, doxorubicin, vincristine and prednisone |
|  | Cancerous leptomeningitis and familial congenital hypopituitarism |
|  | Social role conflict predicts stimulated cytokine production among men, not women |
|  | Socioeconomic status, financial stress, and glucocorticoid resistance among youth with asthma: Testing the moderation effects of maternal involvement and warmth |
|  | Should patients with Phosphomannomutase 2-CDG (PMM2-CDG) be screened for adrenal insufficiency? |
| Prednisolone | Corticosteroids for managing tuberculous meningitis |
|  | Prednisolone-responsive Postpartum IgG4-related Hypophysitis |
|  | A brain mass in a patient with Behcet's disease: a case report |
|  | Prednisolone has a positive effect on the kidney but not on the liver of brain dead rats: a potencial role in complement activation |
|  | Effects of prednisolone on the dystrophin-associated proteins in the blood-brain barrier and skeletal muscle of dystrophic mdx mice |
|  | Brain abscess caused by Nocardia asiatica |
|  | [Neuromyelitis optica] |
|  | CLIPPERS: Eine seltene Diagnose mit charakteristischem MRT-Erscheinungsbild |
|  | Neuro-Sweet's disease |
|  | [Clinically isolated syndrome] |
|  | Prednisolone does not improve olfactory function after COVID-19: a randomized, double-blind, placebo-controlled trial |
|  | RETROSPECTIVE COMPARISON OF THREE-DIMENSIONAL CONFORMAL RADIATION THERAPY VS. PREDNISOLONE ALONE IN 30 CASES OF CANINE INFRATENTORIAL BRAIN TUMORS |
|  | Diagnosis of Guillain-Barré syndrome and validation of Brighton criteria |
|  | Steroid-responsive depression |
|  | Prednisolone increases neural reactivity to negative socio-emotional stimuli in healthy young men |
|  | Management of the brain-dead organ donor: a systematic review and meta-analysis |
|  | Muscle disease |
|  | Corticosteroids in the management of brain-dead potential organ donors: a systematic review |
|  | A Clinical Study of Miliary Brain Tuberculomas in China |
|  | [Hashimoto encephalopathy] |
|  | The corticosteroid prednisolone increases amygdala and insula reactivity to food approach signals in healthy young men |
|  | Macrophage enzyme and reduced inflammation drive brain correction of mucopolysaccharidosis IIIB by stem cell gene therapy |
|  | Steroid Anti-Inflammatory Effects Did Not Improve Organ Quality in Brain-Dead Rats |
|  | Prednisolone induces microglial activation in the subnucleus caudalis of the rat trigeminal sensory complex |
|  | Effects of prednisolone on behavioral and inflammatory profile in animal model of PTZ-induced seizure |
|  | A rational pharmacologic approach toward a biologically meaningful subtype of autism spectrum disorder |
|  | Rosai-Dorfman disease with extranodal involvement |
|  | COVID-19-related encephalopathy responsive to high-dose glucocorticoids |
|  | [New treatments for cluster headache] |
|  | Confusing presentation of chaetomium brain abscess |
|  | Hemodynamic Effects of High-dose Levothyroxine and Methylprednisolone in Brain-dead Potential Organ Donors |
|  | Prednisolone markedly reduced serum IgG4 levels along with the improvement of pituitary mass and anterior pituitary function in a patient with IgG4-related infundibulo-hypophysitis |
|  | Prednisolone versus placebo addition in the treatment of patients with recent-onset psychotic disorder: a trial design |
|  | Neuromyelitis optica spectrum disorder presenting in an octogenarian |
|  | What's in a name? |
|  | The effect of prednisolone on symptom severity in schizophrenia: A placebo-controlled, randomized controlled trial |
|  | Suspected acquired narcolepsy in 8 dogs |
|  | Low dose prednisolone and insulin sensitivity differentially affect arterial stiffness and endothelial function: An open interventional and cross-sectional study |
|  | The possible protective effects of vitamin E and selenium administration in oxidative stress caused by high doses of glucocorticoid administration in the brain of rats |
|  | Pediatric Parainfectious Encephalitis Associated With COVID-19 |
|  | The Protective Effect of Omeprazole Against Traumatic Brain Injury: An Experimental Study |
|  | Uncertainties in endocrine substitution therapy for central hypocortisolism |
|  | Headache in a young woman: do not forget Susac's syndrome |
|  | Solid lipid nanoparticles surface modified with anti-Contactin-2 or anti-Neurofascin for brain-targeted delivery of medicines |
|  | Risk of vigabatrin-associated brain abnormalities on MRI in the treatment of infantile spasms is dose-dependent |
|  | Vascular brain-derived neurotrophic factor pathway in rats with adjuvant-induced arthritis: Effect of anti-rheumatic drugs |
|  | Maternal use of prednisolone is unlikely to be associated with neonatal adrenal suppression-a single-center study of 16 cases |
|  | Angiostrongylus cantonensis: Agent of a Sometimes Fatal Globally Emerging Infectious Disease (Rat Lungworm Disease) |
|  | Hypothalamic-pituitary-adrenal axis recovery following prolonged prednisolone therapy in infants |
|  | Effects of methylprednisolone on inflammatory activity and oxidative stress in the lungs of brain-dead rats |
|  | Childhood acute disseminated encephalomyelitis: an Egyptian pilot study |
|  | A case report of eosinophilic myocarditis and a review of the relevant literature |
|  | Isolated IgG4-related hypertrophic pachymeningitis |
|  | A new way of thinking: hydrocortisone in traumatic brain-injured patients |
|  | A case of IgG4-related hypophysitis without pituitary insufficiency |
|  | Cerebellar degeneration in primary Sjӧgren syndrome |
|  | Effects of methylprednisolone on blood-brain barrier and cerebral inflammation in cardiac surgery-a randomized trial |
|  | A woman with intractable nausea and vomiting |
|  | Bevacizumab Monotherapy Reduces Radiation-induced Brain Necrosis in Nasopharyngeal Carcinoma Patients: A Randomized Controlled Trial |
|  | Epileptic spasms - 175 years on: Trying to teach an old dog new tricks |
|  | Miller Fisher Syndrome Mimicking Tolosa-Hunt Syndrome |
|  | Neutrophilic dermatosis associated with an NFKB2 mutation |
|  | Evaluation of cerebral blood flow in the hippocampus, thalamus, and basal ganglia and the volume of the hippocampus in dogs before and during treatment with prednisolone |
|  | Twenty-four hour urinary cortisol excretion and the metabolic syndrome in prednisolone-treated renal transplant recipients |
|  | Atypical presentation of multiple sclerosis |
|  | Effect of the systemic administration of methylprednisolone on the lungs of brain-dead donor rats undergoing pulmonary transplantation |
|  | Faciobrachial dystonic seizures arise from cortico-subcortical abnormal brain areas |
|  | Intractable Neurosarcoidosis Effectively Treated with Infliximab |
|  | Prenatal diagnosis of hemimegalencephaly |
|  | Neurotrichinosis in a pediatric patient |
|  | Balo concentric sclerosis in a 23-year-old man |
|  | Procarbazine, lomustine and vincristine for recurrent high-grade glioma |
|  | Henoch-Schönlein purpura from vasculitis to intestinal perforation: A case report and literature review |
|  | Corticosteroid therapy for duchenne muscular dystrophy: improvement of psychomotor function |
|  | Strategies to prevent the neuropsychiatric side-effects of corticosteroids: a case report and review of the literature |
|  | Unsuccessful cyclosporine plus prednisolone therapy for autoimmune meningoencephalitis in three dogs |
|  | Malaysian outcome of acute necrotising encephalopathy of childhood |
|  | CLIPPERS and the need for long-term immunosuppression |
|  | Wegener granulomatosis-associated optic perineuritis |
|  | Optic neuritis and the evaluation of visual impairment in multiple sclerosis |
|  | Steroid responsive encephalopathy associated with autoimmune thyroiditis (SREAT) in childhood |
|  | [Guillain-Barré syndrome associated with acute hepatitis A-A case report and literature review] |
|  | Fulminant tumefactive multiple sclerosis in pregnancy |
|  | Case of steroid-responsive encephalopathy from hypoglycaemia |
|  | Innate inflammatory gene expression profiling in potential brain-dead donors: detailed investigation of the effect of common corticosteroid therapy |
|  | Rheumatoid Meningitis Occurring during Etanercept Treatment |
|  | Acute Disseminated Encephalomyelitis in an Incarcerated Adolescent Presents as Acute Psychosis: Case Report and Literature Review |
|  | Horizontal eyeball akinesia as an initial manifestation of CLIPPERS: Case report and review of literature |
|  | Acute hemicerebellitis in a young adult: a case report and literature review |
|  | Comparison of methods for evaluation of the suppressive effects of prednisolone on the HPA axis and bone turnover: changes in s-DHEAS are as sensitive as the ACTH test |
|  | Hashimoto's encephalopathy mimicking presenile dementia |
|  | Intraoperative Methylprednisolone and Neurodevelopmental Outcomes in Infants After Cardiac Surgery |
|  | Focal Cerebral Arteriopathy: Do Steroids Improve Outcome? |
|  | [A case of optic perineuritis-A literature review of Japanese cases and clinical problems] |
|  | IV steroids during long episodes of Kleine-Levin syndrome |
|  | Clinical, radiographic characteristics and immunomodulating changes in neuromyelitis optica with extensive brain lesions |
|  | [Human herpesvirus 6 encephalitis followed by acute disseminated encephalomyelitis in an immunocompetent adult] |
|  | Case Report: Long-Term Chemotherapy With Hydroxyurea and Prednisolone in a Cat With a Meningioma: Correlation of FDG Uptake and Tumor Grade Assessed by Histopathology and Expression of Ki-67 and p53 |
|  | Spontaneous thrombosis of the main draining vein revealing an unruptured brain arteriovenous malformation |
|  | [A case of inflammatory cerebral amyloid angiopathy with white matter lesions appearing after brain biopsy] |
|  | Progressive meningoencephalitis due to neurosarcoidosis |
|  | Glucocorticoid-associated worsening in reversible cerebral vasoconstriction syndrome |
|  | Stress-dose hydrocortisone reduces critical illness-related corticosteroid insufficiency associated with severe traumatic brain injury in rats |
|  | Rituximab treatment for relapsed opsoclonus-myoclonus syndrome |
|  | Clinically Mild Encephalitis/Encephalopathy With a Reversible Splenial Lesion Accompanied by Epstein-Barr Virus Hemophagocytic Lymphohistiocytosis: A Case Report and Review of the Literature |
|  | Variant of multiple sclerosis with dementia and tumefactive demyelinating brain lesions |
|  | Comparison of Gefitinib versus VMP in the combination with radiotherapy for multiple brain metastases from non-small cell lung cancer |
|  | Progressive Multifocal Leukoencephalopathy Following Treatment with Rituximab in an HIV-Negative Patient with Non-Hodgkin Lymphoma. A Case Report and Literature Review |
|  | A cerebral phenotype of chronic lymphocytic inflammation with pontine perivascular enhancement responsive to steroids: A case report and review of the literature |
|  | Efficacy of pulse intravenous methylprednisolone in epileptic encephalopathy: a randomised controlled trial |
|  | The role of mineralocorticoid receptor function in treatment-resistant depression |
|  | [Multiple cerebral infarctions in the deep perforator regions in a case of idiopathic hypereosinophilic syndrome] |
|  | Hypertrophic pachymeningoencephalitis associated with temporal giant cell arteritis |
|  | Eosinophilic pneumonia induced by ceftaroline |
|  | Behçet's disease aggravated after tooth extraction |
|  | Longstanding spastic paraparesis in a patient infected with hepatitis C virus and seropositive for aquaporin-4 antibody - Case report and review of the literature |
|  | Cerebral venous sinus thrombosis and subdural hematoma in a female patient with systemic lupus erythematosus: a case report and literature review |
|  | Isolated Third Cranial Nerve Palsy Leading to the Diagnosis of Disseminated Burkitt Lymphoma: A Case Report and Literature Review |
|  | Hashimoto's encephalopathy: report of three cases |
|  | Dramatic response of diffuse osteosclerosis secondary to multiple myeloma using thalidomide with melphalan and prednisolone |
|  | Role of Angiography in Systemic Lupus Erythematosus-Induced Choroiditis |
|  | [Cerebral venous sinus thrombosis in the patient with multiple sclerosis associated with congenital antithrombin deficiency] |
|  | [Low signal intensity lesions on brain susceptibility-weighted MRI in a patient with intravascular large B-cell lymphoma] |
|  | Cardiotoxicity with rituximab, cyclophosphamide, non-pegylated liposomal doxorubicin, vincristine and prednisolone compared to rituximab, cyclophosphamide, doxorubicin, vincristine, and prednisolone in frontline treatment of patients with diffuse large B-cell lymphoma: A randomised phase-III study from the Austrian Cancer Drug Therapy Working Group [Arbeitsgemeinschaft Medikamentöse Tumortherapie AGMT](NHL-14) |
|  | [Diagnostic value of brain biopsy in intravascular large B-cell lymphoma mimicking progressive multifocal leukoencephalopathy: case report] |
|  | Oral rather than intravenous corticosteroids should be used to treat MS relapses - Commentary |
|  | Sonic Hedgehog Agonist Protects Against Complex Neonatal Cerebellar Injury |
|  | Hypothalamic-pituitary-adrenal (HPA) axis suppression after treatment with glucocorticoid therapy for childhood acute lymphoblastic leukaemia |
|  | Rheumatoid pannus compressing the medulla oblongata |
|  | Magnetic resonance imaging and magnetic resonance spectroscopy in a young male patient with anti-N-methyl-D-aspartate receptor encephalitis and uncommon cerebellar involvement: A case report with review of the literature |
|  | Drug-induced thrombocytopenia secondary to natalizumab treatment |
|  | Corticosteroid induced hyperosmolar hyperglycaemic state and hemiballismus |
|  | Striatal TH-immunopositive fibers recover after an intrastriatal injection of 6-hydroxydopamine in golden hamsters treated with prednisolone: roles of tumor necrosis factor-α and inducible nitric oxide synthase in neurodegeneration |
|  | Neuro-Behcet disease presenting as a solitary cerebellar hemorrhagic lesion: a case report and review of the literature |
|  | Reduced neurotoxicity with combined treatment of high-dose methotrexate, cyclophosphamide, doxorubicin, vincristine and prednisolone (M-CHOP) and deferred radiotherapy for primary central nervous system lymphoma |
|  | Neuroprotective Effects of C-terminal Domain of Tetanus Toxin on Rat Brain Against Motorneuron Damages After Experimental Spinal Cord Injury |
|  | Anti-N-methyl-D-aspartate receptor encephalitis concomitant with multifocal subcortical white matter lesions on magnetic resonance imaging: a case report and review of the literature |
|  | Positron emission tomography/computed tomography scan of Vogt-Koyanagi-Harada syndrome with associated autoimmune thyroid disease: A case report and literature review |
|  | Methylprednisolone sodium succinate reduces BBB disruption and inflammation in a model mouse of intracranial haemorrhage |
|  | Scalp-recorded high-frequency oscillations in atypical benign partial epilepsy |
|  | Rheumatoid disease: an unusual cause of relapsing meningoencephalitis |
|  | Idiopathic hypertrophic pachymeningitis with anticardiolipin antibody: A case report |
|  | Can pulse steroid therapy increase the risk of infection by COVID-19 in patients with multiple sclerosis? |
|  | Frosted branch angiitis and cerebral venous sinus thrombosis as an initial onset of neuro-Behçet's disease: a case report and review of the literature |
|  | Effects of erythropoietin and methylprednisolone on AQP4 expression in astrocytes |
|  | Neuromyelitis Optica Spectrum Disorder with Recurrent Intracranial Hemorrhage |
|  | Balo concentric sclerosis: A presentation mimicking ischaemic stroke |
|  | Acute reversible Marchiafava-Bignami disease with hypernatremia: a "callosal myelinolysis"? |
|  | Cotard syndrome in Tumefactive Multiple Sclerosis- A case report |
|  | Clinical diagnosis of LGI1 antibody encephalitis in an 83-year-old woman |
|  | Physiological pituitary hyperplasia misinterpreted and treated as lymphocytic hypophysitis |
|  | Guillain-Barré syndrome and optic neuritis after Mycoplasma pneumoniae infection |
|  | Characteristics of (18)F-FDG and (18)F-FDOPA PET in an 8-year-old neutered male Yorkshire Terrier dog with glioma: long-term chemotherapy using hydroxyurea plus imatinib with prednisolone and immunoreactivity for PDGFR-β and LAT1 |
|  | [Obstructive pneumonia and brain abscess due to Nocardia elegans in a patient with systemic lupus erythematosus] |
|  | Primary CNS lymphoma in a patient treated with azathioprine |
|  | Steroid treatment as anti-inflammatory and neuroprotective agent following out-of-hospital cardiac arrest: a randomized clinical trial |
|  | Auditory processing following infantile spasms: An event-related potential study |
|  | Delayed leucoencephalopathy after coil embolisation of unruptured cerebral aneurysm |
|  | Liver injury after pulsed methylprednisolone therapy in multiple sclerosis patients |
|  | Pediatric opsoclonus-myoclonus-ataxia syndrome: Experience from a tertiary care university hospital |
|  | Effects of immunotherapies and clinical outcomes in neurosarcoidosis: a retrospective cohort study |
|  | Orbital mass secondary to infantile acute lymphoblastic leukaemia |
|  | Clinical analysis of neuromyelitis optica presenting as intractable nausea, vomiting and hiccups |
|  | Late-Onset Post-transplantation Central Nervous System Lymphoproliferative Disorder: Case Report |
|  | Dual diagnosis: rheumatoid arthritis and multiple sclerosis |
|  | Idiopathic generalised tremor syndrome in two cats |
|  | Measurable outcomes for pediatric epileptic encephalopathy: a single-center experience with corticosteroid therapy |
|  | Pediatric anti-NMDA (N-methyl D-aspartate) receptor encephalitis |
|  | Differential effects of sympathetic nervous system and hypothalamic-pituitary-adrenal axis on systemic immune cells after severe experimental stroke |
|  | Autoimmune glial fibrillary acidic protein astrocytopathy in Chinese patients: a retrospective study |
|  | [Cerebral infarction related to varicella zoster virus vasculopathy] |
|  | Post-operative quadriplegia as the initial manifestation of tumefactive multiple sclerosis |
|  | Immunotherapy-responsive childhood neurodegeneration with systemic and central nervous system inflammation |
|  | Artemisinin therapeutic efficacy in the experimental model of multiple sclerosis |
|  | Susac syndrome: clinical characteristics, clinical classification, and long-term prognosis |
|  | Magnetic resonance imaging findings in giant cell arteritis |
|  | Visual disturbance with systemic symptoms: old lessons revisited |
|  | Acute flaccid myelitis-Clustering of polio-like illness in the tertiary care centre in Southern India |
|  | [Routine hormonal therapy in the heart transplant donor] |
|  | Rhomb- and bickerstaff encephalitis: two clinical phenotypes? |
|  | Autoimmune encephalitis with GABA(A) receptor antibodies in a 10-year-old girl |
|  | Evaluating tertiary adrenal insufficiency in rheumatology patients on long-term systemic glucocorticoid treatment |
|  | Metaphyseal osteopathy in three Australian Kelpie siblings |
|  | Progressive micrographia without parkinsonism caused by autoimmune brainstem encephalitis: A case report |
|  | Secondary CNS involvement of ALK-negative anaplastic large cell lymphoma |
|  | Perforation in an intestinal malignant lymphoma case |
|  | Reversible splenial lesion syndrome associated with SARS-CoV-2 infection in two children |
|  | A severe neurological complication of influenza in a previously well child |
|  | A pediatric patient of hemorrhagic acute transverse myelitis |
|  | [Successful combination immunotherapy of anti-gamma aminobutyric acid (GABA)(A) receptor antibody-positive encephalitis with extensive multifocal brain lesions] |
|  | Etiologic spectrum and functional outcome of the acute inflammatory myelitis |
|  | A proteomic investigation into mechanisms underpinning corticosteroid effects on neural stem cells |
|  | Granulomatosis with polyangiitis presenting with unilateral facial nerve palsy and nasal septum perforation |
|  | A rare case of SPG11 mutation with multiple sclerosis |
|  | Pediatric Acute Flaccid Paralysis: Enterovirus D68-Associated Anterior Myelitis |
|  | Long-term outcomes of steroid therapy for Duchenne muscular dystrophy in Japan |
|  | A Rare and Fatal Case of Viral Encephalitis in an Immunocompetent Host |
|  | Progressive Susac syndrome with bilateral visual loss and disability |
|  | Persistently Gadolinium-Enhancing Lesion Is a Predictor of Poor Prognosis in NMOSD Attack: a Clinical Trial |
|  | The onset location of neuromyelitis optica spectrum disorder predicts the location of subsequent relapses |
|  | Liver injury, rash, and encephalopathy in a 23-year-old Asian man prescribed dapsone |
|  | [A case of cysticercosis with multiple lesions in the brain and femoral muscles] |
|  | Hypertrophic pachymeningitis: significance of myeloperoxidase anti-neutrophil cytoplasmic antibody |
|  | Jisuikang, a Chinese herbal formula, increases neurotrophic factor expression and promotes the recovery of neurological function after spinal cord injury |
|  | Acute exacerbation of idiopathic pulmonary fibrosis after total hip replacement |
|  | Primary dural diffuse large B cell lymphoma mimicking parafalcine meningioma |
|  | Encephalopathy Associated with Influenza B in a Healthy Young Man |
|  | Pharmacokinetics of Budesonide Administered with Surfactant in Premature Lambs: Implications for Neonatal Clinical Trials |
|  | Antibodies to myelin oligodendrocyte glycoprotein in idiopathic optic neuritis |
|  | Effect evaluation of methylprednisolone plus mitochondrial division inhibitor-1 on spinal cord injury rats |
|  | The Effect of Preoperative Methylprednisolone on Postoperative Delirium in Older Patients Undergoing Gastrointestinal Surgery: A Randomized, Double-Blind, Placebo-Controlled Trial |
|  | [Intracranial germinoma masquerading as a granulomatous inflammation, diagnostic failure after brain biopsy] |
|  | Glycine receptor antibody-associated epilepsy in a boy aged 4 years |
|  | Short-Term Outcome of Intravenous Methylprednisolone Pulse Therapy in Patients With Infantile Spasms |
|  | Anterior horn syndrome: A rare manifestation of primary Sjögren's syndrome |
|  | Isolated CNS involvement in eosinophilic granulomatosis with polyangiitis treated with mepolizumab: A case report |
|  | Methylprednisolone as a memory enhancer in rats: Effects on aversive memory, long-term potentiation and calcium influx |
|  | Blunted glucocorticoid and mineralocorticoid sensitivity to stress in people with diabetes |
|  | [A case of neuromyelitis optica spectrum disorder (NMOSD) with Sjögren's syndrome manifested only brain involvement by preceding parotitis] |
|  | [Brain stem infarction, temporal headache, and elevated inflammatory parameters in a 74-year-old man] |
|  | CNS vasculitis in a patient with MS on daclizumab monotherapy |
|  | Recurrent hypoglycaemia: a delayed presentation of Sheehan syndrome |
|  | [Case report of a 28-year-old male with the rapid progression of steroid-resistant central nervous system vasculitis diagnosed by a brain biopsy] |
|  | A study of methylprednisolone neuroprotection against acute injury to the rat spinal cord in vitro |
|  | Early-Onset Multiple Sclerosis With Frequent Relapses: A Challenging Diagnosis With a Less Favorable Prognosis |
|  | Brainstem encephalitis and acute polyneuropathy associated with hepatitis E infection |
|  | Stress-Dose Corticosteroid Versus Placebo in Neonatal Cardiac Operations: A Randomized Controlled Trial |
|  | Corticosteroid therapy and severity of vasogenic edema in posterior reversible encephalopathy syndrome |
|  | Dynamic changes of central thyroid functions in the management of Cushing's syndrome |
|  | Neuromyelitis optica accompanied by nephrotic syndrome and autoimmune-related pancytopenia |
|  | Recurrent Bell's palsy following ventriculoperitoneal shunt insertion: an unusual case to face |
|  | Vegetarian diet and excessive tea consumption: a dangerous association? |
|  | Demyelinating encephalopathy in adult onset Still's disease: case report and review of the literatures |
|  | Behcet's disease presenting as intracranial hypertension due to cerebral venous thrombosis |
|  | Outcome of limbic encephalitis with VGKC-complex antibodies: relation to antigenic specificity |
|  | Severe neurological manifestations of influenza during 2018-2019 influenza season: Case series of 13 pediatric patients |
|  | Varicella zoster virus cerebral aneurysmal vasculopathy presenting in a newly-diagnosed HIV-positive patient |
|  | Prognostic factors for profound sudden idiopathic sensorineural hearing loss: a multicenter retrospective study |
|  | Modeling non-syndromic autism and the impact of TRPC6 disruption in human neurons |
|  | Paraneoplastic encephalopathy: an unusual presenting feature of bladder cancer metastasis |
|  | Monocular Oculomotor Nerve Disorder Manifesting as Cranial Neuropathy in Systemic Lupus Erythematosus |
|  | Delayed recurrent enhancing white matter lesions complicating coiling of intracranial aneurysm |
|  | Immunotherapy-responsive limbic encephalitis with antibodies to glutamic acid decarboxylase |
|  | Human herpes virus 8-unrelated primary effusion lymphoma-like lymphoma presenting with cardiac tamponade: A case report |
|  | Acute Leukoencephalopathy with Restricted Diffusion in an Infant with Severe COVID-19 and Dengue Coinfection Progressing to West Syndrome |
|  | Toxoplasma gondii infection in the peritoneal macrophages of rats treated with glucocorticoids |
|  | Relapses in multiple sclerosis: effects of high-dose steroids on cortical excitability |
|  | Chronic periodic lateralised epileptic discharges and anti-N-methyl-D-aspartate receptor antibodies |
|  | An unusual case of encephalitis with starry sky pattern in MRI |
|  | Ramsay Hunt Syndrome Complicated by Brainstem Encephalitis in Varicella-zoster Virus Infection |
|  | Anti-NMDA Receptor Encephalitis in a Young Girl with Altered Behaviour and Abnormal Movements |
|  | Clinical Features, Therapeutic Response, and Follow-Up in Pediatric Anti-N-Methyl-D-Aspartate Receptor Encephalitis: Experience from a Tertiary Care University Hospital in India |
|  | Myelin Oligodendrocyte Glycoprotein Antibody Persistency in a Steroid-Dependent ADEM Case |
|  | Bilateral frontal cortex encephalitis and paraparesis in a patient with anti-MOG antibodies |
|  | A case report of Sjögren syndrome manifesting bilateral basal ganglia lesions |
|  | The influence of immunosuppressive drugs on neural stem/progenitor cell fate in vitro |
|  | Cerebral demyelination in children with collagenous colitis |
|  | Steroid-responsive encephalopathy: an under recognised aspect of Hashimoto's thyroiditis |
|  | Efficacy of intravenous methylprednisolone pulse therapy in patients with multiple sclerosis and neuromyelitis optica |
|  | Hypertrophic Pachymeningitis as a Potential Cause of Headache Associated with Temporal Arteritis |
|  | Comparison of systemic and localized carrier-mediated delivery of methylprednisolone succinate for treatment of acute spinal cord injury |
|  | CD8 transverse myelitis in a patient with HIV-1 infection |
|  | MS disease activity in RESTORE: a randomized 24-week natalizumab treatment interruption study |
|  | Adult-onset Opsoclonus-Myoclonus Syndrome Associated With Ganglionic Acetylcholine Receptor Autoantibody |
|  | Multiple intracranial nodules associated with rheumatoid arthritis: case report |
|  | Letter: The Impact of Guidelines on Clinical Practice: Survey of the Use of Methylprednisolone for Acute Spinal Cord Injury |
|  | Multisystem Inflammatory Syndrome in a Child with Scrub Typhus and Macrophage Activation Syndrome |
|  | Chronic lymphocytic inflammation with pontine perivascular enhancement responsive to steroids and human leukocyte antigen |
|  | Effects of anti-inflammatory drugs on the expression of tryptophan-metabolism genes by human macrophages |
|  | CD8-Positive T-Cell Leukoencephalitis With Astrocytopathy Clinically Presenting as Neuromyelitis Optica |
|  | Infectious causes of acute meningitis among Thai adults in a university hospital |
|  | Biopsy-proven case of Epstein-Barr virus (EBV)-associated vasculitis of the central nervous system |
|  | Targeted temperature management for acute encephalopathy in a Japanese secondary emergency medical care hospital |
|  | Comparison of high- and low-dose corticosteroid regimens for organ donor management |
|  | Disseminated juvenile xanthogranuloma occurring after treatment of Langerhans cell histiocytosis: a case report |
|  | CNS-targeted glucocorticoid reduces pathology in mouse model of amyotrophic lateral sclerosis |
|  | Glucocorticoid treatment increases density of serotonin 5-HT2A receptors in humans |
|  | Juvenile systemic lupus erythematosus with primary neuropsychiatric presentation |
|  | CNS intravascular lymphoma: an underappreciated cause of rapidly progressive dementia |
|  | Prophylactic and therapeutic functions of drug combinations against noise-induced hearing loss |
|  | Hashimoto encephalopathy presenting as progressive myoclonus epilepsy syndrome |
|  | Treatment with methotrexate and low-dose corticosteroids in sarcoidosis patients with cardiac lesions |
|  | Polyarteritis nodosa with central nervous system involvement mimicking relapsing-remitting multiple sclerosis |
|  | Cranial Nerve-VI Palsy as the Main Clinical Manifestation of Neurosarcoidosis |
|  | Mikulicz's Disease with hypophysitis - a new IgG4-mediated disorder |
|  | Simultaneous quantification of seven hippocampal neurotransmitters in depression mice by LC-MS/MS |
|  | Scalp-recorded high-frequency oscillations in childhood epileptic encephalopathy with continuous spike-and-wave during sleep with different etiologies |
|  | Apparent Hypothalamic-Pituitary-Adrenal Axis Suppression via Reduction of Interleukin-6 by Glucocorticoid Therapy in Systemic Autoimmune Diseases |
|  | Atorvastatin calcium in combination with methylprednisolone for the treatment of multiple sclerosis relapse |
|  | Methylprednisolone inhibits the proliferation of endogenous neural stem cells in nonhuman primates with spinal cord injury |
|  | Myelitis in systemic lupus erythematosus: clinical characteristics and effect in accrual damage. A single-center experience |
|  | Recurrent headaches: a case of neurological Behçet's disease |
|  | Recurrent steroid-responsive cerebral vasogenic edema in status migrainosus and persistent aura |
|  | Radiomics Analysis of DTI Data to Assess Vision Outcome After Intravenous Methylprednisolone Therapy in Neuromyelitis Optic Neuritis |
|  | Early predictors of rapidly evolving multiple sclerosis: A case report |
|  | Management of paradoxical response in pediatric tubercular meningitis with methylprednisolone |
|  | [A case of Bickerstaff brainstem encephalitis with transient reflex myoclonus] |
|  | Acute disseminated encephalomyelitis in children and adolescents: a single center experience |
|  | [Subacute anti-N-methyl-D-aspartate receptor encephalitis. A serie of 13 paediatric cases] |
|  | Methylprednisolone for the Treatment of Patients with Acute Spinal Cord Injuries: A Propensity Score-Matched Cohort Study from a Canadian Multi-Center Spinal Cord Injury Registry |
|  | A patient with systemic lupus erythematosus complicated by neurological symptoms of toluene poisoning |
|  | A randomized clinical trial of oral versus intravenous methylprednisolone for relapse of MS |
|  | Isolated dysphagia unmasking bulbar neurosarcoidosis and pulmonary sarcoidosis |
|  | Occipital nerve block for the short-term preventive treatment of migraine: A randomized, double-blinded, placebo-controlled study |
|  | The effect of epidural methylprednisolone acetate injection on the hypothalamic-pituitary-adrenal axis |
|  | [Two pediatric cases of anti-NMDA receptor antibody encephalitis] |
|  | Acute penile pain and swelling in a 4-year-old child with Henoch-Schönlein purpura |
|  | Myelin Oligodendrocyte Glycoprotein (MOG)-IgG Associated Demyelinating Disease: Our Experience with this Distinct Syndrome |
|  | Acute Urinary Retention Induced by Chemical Meningitis Which Occurred Due to a Ruptured Dermoid Cyst |
|  | A case report of obsessive-compulsive disorder following acute disseminated encephalomyelitis |
|  | Glutathione pegylated liposomal methylprednisolone administration after the early phase of status epilepticus did not modify epileptogenesis in the rat |
|  | Diffusion weighted MR imaging of 5-fluorouracil and oxaliplatin-induced leukoencephalopathy |
|  | Detection of branch retinal artery occlusions in Susac's syndrome |
|  | Serous retinal detachment and cystoid macular edema in a patient with Wyburn-Mason syndrome |
|  | Retrospective follow up of gross motor development in children using propranolol for treatment of infantile haemangioma at Sydney Children's Hospital |
|  | Bilateral optic neuritis related to chronic inflammatory demyelinating polyneuropathy |
|  | Bell's palsy in a pediatric patient with hyper IgM syndrome and severe acute respiratory syndrome coronavirus 2 (SARS-CoV-2) |
|  | Steroid pulse therapy in patients with encephalopathy associated with severe fever with thrombocytopenia syndrome |
|  | Changes in Blood B Cell-Activating Factor (BAFF) Levels in Multiple Sclerosis: A Sign of Treatment Outcome |
|  | Lethal high: acute disseminated encephalomyelitis (ADEM) triggered by toxic effect of synthetic cannabinoid black mamba |
|  | Longitudinal Extensive Transverse Myelitis and Central Diabetes Insipidus: A Severe Flare of Systemic Lupus Erythematosus |
|  | Treatment of multiple sclerosis relapses with high-dose methylprednisolone reduces the evolution of contrast-enhancing lesions into persistent black holes |
|  | Punctate lesion pattern suggestive of perivascular inflammation in acute natalizumab-associated progressive multifocal leukoencephalopathy: productive JC virus infection or preclinical PML-IRIS manifestation? |
|  | Multiple intracranial lesions as the unusual imaging features of Hashimoto's encephalopathy: A case report |
|  | Extra-axial primary non-Hodgkin's CNS lymphoma mimicking meningioma, in a 5-year-old immunocompetent child: a rare entity |
|  | Intratympanic methylprednisolone versus gentamicin in patients with unilateral Ménière's disease: a randomised, double-blind, comparative effectiveness trial |
|  | Disease Activity and Conversion into Multiple Sclerosis after Optic Neuritis Is Treated with Erythropoietin |
|  | Clinical Outcomes of Myocarditis after Moderate-Dose Steroid Therapy in Systemic Sclerosis: A Pilot Study |
|  | First use of alemtuzumab in Balo's concentric sclerosis: a case report |
|  | Using genome-wide CRISPR library screening with library resistant DCK to find new sources of Ara-C drug resistance in AML |
|  | Acute disseminated encephalomyelitis associated with acute Toxoplasma gondii Infection |
|  | A teenage girl with new-onset diplopia and sharp, shooting leg pain |
|  | Idiopathic Non-traumatic Facial Nerve Palsy (Bell's Palsy) in Neonates; An Atypical Age and Management Dilemma |
|  | Infantile Hemangioma of the Posterior Fossa in a Newborn: Early Management and Long-Term Follow-up |
|  | Bilateral diffuse scleritis as a first manifestation of immunoglobulin G4-related sclerosing pachymeningitis |
|  | A benzothiadiazine derivative and methylprednisolone are novel and selective activators of transient receptor potential canonical 5 (TRPC5) channels |
|  | Susac's syndrome: an immune mediated endotheliopathy laden with challenges and controversies |
|  | Embolic Stroke due to Carotidynia Potentially Associated with Moving Carotid Artery Caused by Swallowing |
|  | Apolipoproteins are associated with new MRI lesions and deep grey matter atrophy in clinically isolated syndromes |
|  | Adaptive changes in basal and stress-induced HPA activity in lactating and post-lactating female rats |
|  | Sjögren syndrome presenting with encephalopathy mimicking Creutzfeldt-Jakob disease |
|  | FNDC5 expression in Purkinje neurons of adult male rats with acute spinal cord injury following treatment with methylprednisolone |
|  | Paraneoplastic Demyelinating Sensorimotor Neuropathy Delaying the Diagnosis of an Underlying Acute Lymphoblastic Leukemia in a Child |
|  | Opercular myoclonic-anarthric status epilepticus due to glutamic acid decarboxylase antibody-associated encephalitis |
|  | Simultaneous bilateral knee injection of methylprednisolone acetate and the hypothalamic-pituitary adrenal axis: a single-blind case-control study |
|  | Incomplete peripheral facial nerve palsy and ulnar neuropathy due to leprosy mistaken as faciobrachial stroke |
|  | Acute transverse myelitis (ascending myelitis) as the initial manifestation of Japanese encephalitis: a rare presentation |
|  | Anti-inflammatory and antioxidant activities of Costus afer Ker Gawl. hexane leaf fraction in arthritic rat models |
|  | Exserohilum infections associated with contaminated steroid injections: a clinicopathologic review of 40 cases |
|  | Clinical spectrum and treatment outcome of West Syndrome in children from Northern India |
|  | Transient adrenal insufficiency in diffuse large B cell lymphoma patients after chemotherapy with short-course, high-dose corticosteroids |
|  | A Young Woman with Ischemic Stroke: Should We Pay More Attention to Varicella Zoster Infection? |
|  | Adhesion molecules, chemokines and matrix metallo-proteinases response after albendazole and albendazole plus steroid therapy in swine neurocysticercosis |
|  | Choroid plexus papilloma in a dog surviving for 15 months after diagnosis with symptomatic therapy |
|  | Use of MRI signal intensity of extraocular muscles to evaluate methylprednisolone pulse therapy in thyroid-associated ophthalmopathy |
|  | Intra-articular methylprednisolone acetate injection at the knee joint and the hypothalamic-pituitary-adrenal axis: a randomized controlled study |
|  | Voxel-wise magnetization transfer imaging study of effects of natalizumab and IFNβ-1a in multiple sclerosis |
|  | Neuromyelitis optica and neuromyelitis optica spectrum disorder: Natural history and long-term outcome, an Indian experience |
|  | Steroid responsive encephalopathy associated with autoimmune thyroiditis (SREAT) presenting as major depression |
|  | Moyamoya syndrome as an unusual presenting manifestation of systemic lupus erythematosus in a young woman |
|  | Imatinib mesylate plus hydroxyurea chemotherapy for cerebellar meningioma in a Belgian Malinois dog |
|  | Extensive intracranial involvement with multiple dissections in a case of giant cell arteritis |
|  | Neurocysticercosis presenting as acute psychosis: a rare case report from rural India |
|  | Non-thrombotic superior sagittal sinus occlusion with intracranial hypertension following metastatic Burkitt's lymphoma |
|  | Successful treatment of neurological malignant atrophic papulosis in child by corticosteroid combined with intravenous immunoglobulin |
|  | Quantitative magnetic resonance imaging evidence for altered structural remodeling of the temporal lobe in West syndrome |
|  | A case report of life-threatening acute dysphagia in dermatomyositis: Challenges in diagnosis and treatment |
|  | Autonomic alterations as a clinical manifestation of encephalopathy associated with autoimmune thyroid disease |
|  | A 67-year-old woman with asthma, word finding difficulty, and an abnormal chest radiograph |
|  | Meningeal inflammation and demyelination in a patient clinically diagnosed with acute disseminated encephalomyelitis |
|  | The alterations of matrix metalloproteinase-9 in mouse brainstem during herpes simplex virus type 1-induced facial palsy |
|  | Glucocorticoid treatment of MCMV infected newborn mice attenuates CNS inflammation and limits deficits in cerebellar development |
|  | Extensive acute disseminated encephalomyelitis in a young girl responding to intravenous methylprednisolone |
|  | Teaching NeuroImages: Ma2 encephalitis presenting as acute panhypopituitarism in a young man |
|  | An unusual case of inflammatory meningitis in a young man with systemic lupus erythematosus |
|  | Gait Disturbance as the Presenting Symptom in Young Children With Anti-NMDA Receptor Encephalitis |
|  | Effect of a constant rate infusion of cytosine arabinoside on mortality in dogs with meningoencephalitis of unknown origin |
|  | [Reversible posterior leukoencephalopathy syndrome in a patient presenting granulomatosis with polyangiitis] |
|  | Blood Pressure Profile and N-Terminal-proBNP Dynamics in Response to Intravenous Methylprednisolone Pulse Therapy of Severe Graves' Orbitopathy |
|  | Transverse myelitis extended to disseminated encephalitis in systemic lupus erythematosus: Histological evidence for vasculitis |
|  | Steroid-responsive intracranial germinoma presenting as Holmes' tremor: importance of a tissue diagnosis |
|  | The First Hand Allotransplantation in Taiwan: A Report at 9 Months |
|  | Need for prolonged immunosupressive therapy in CLIPPERS--a case report |
|  | Vasopressin, steroids, and epinephrine and neurologically favorable survival after in-hospital cardiac arrest: a randomized clinical trial |
|  | Effect of Methylprednisolone on Pain Management in Total Knee or Hip Arthroplasty: A Systematic Review and Meta-Analysis of Randomized Controlled Trials |
|  | The effect of local injection of methylprednisolone acetate on the hypothalamic-pituitary-adrenal axis among patients with greater trochanteric pain syndrome |
|  | Etiologic spectrum and prognosis in noncompressive acute transverse myelopathies: An experience of 80 patients at a tertiary care facility |
|  | Disseminated nocardiosis caused by Nocardia otitidiscaviarum in an immunocompetent host: A case report and literature review |
|  | Improvement in insulin resistance is greater when infliximab is added to methotrexate during intensive treatment of early rheumatoid arthritis-results from the IDEA study |
|  | Randomized, double-blind, comparative-effectiveness study comparing pulsed radiofrequency to steroid injections for occipital neuralgia or migraine with occipital nerve tenderness |
|  | A Patient with Limbic Encephalitis Associated with Anti-leucine-rich Glioma-inactivated 1 (LGI1) Antibody Presenting with Slowly Progressive Cognitive Impairment and Fluctuating Striatal Lesions |
|  | [An adult case of group A streptococcus meningitis associated with steroid-responsive meningoencephalitis] |
|  | Disseminated nocardiosis during systemic steroid therapy for the prevention of esophageal stricture after endoscopic submucosal dissection |
|  | [Concentric Sclerosis Baló: A rare Variant of Multiple Sclerosis] |
|  | Syndrome of inappropriate antidiuretic hormone accompanied by bilateral hypothalamic and anterior thalamic lesions with serum antiaquaporin 4 antibody |
|  | Histologically confirmed case of cerebral vasculitis associated with Crohn's disease--a case report |
|  | Pulsed corticosteroid treatment in MS patients stabilizes disease activity following natalizumab withdrawal prior to switching to fingolimod |
|  | Diffuse large B-cell lymphoma presenting with central pontine myelinolysis: a case report |
|  | Relapses Requiring Intravenous Steroid Use and Multiple-Sclerosis-related Hospitalizations: Integrated Analysis of the Delayed-release Dimethyl Fumarate Phase III Studies |
|  | Rhino-orbitocerebral mucormycosis in a patient with idiopathic crescentic glomerulonephritis |
|  | Glutathione PEGylated liposomal methylprednisolone (2B3-201) attenuates CNS inflammation and degeneration in murine myelin oligodendrocyte glycoprotein induced experimental autoimmune encephalomyelitis |
|  | Phase I-II Clinical Trial Assessing Safety and Efficacy of Umbilical Cord Blood Mononuclear Cell Transplant Therapy of Chronic Complete Spinal Cord Injury |
|  | A case of neuropsychiatric lupus Erythematosus characterized by the Owl's eye sign: a case report |
|  | Management of optic neuritis in Ireland: a survey comparing the management practices of acute demyelinating optic neuritis amongst ophthalmologists and neurologists in Ireland |
|  | Atypical sino-orbital inflammatory myofibroblastic tumor with bone and cerebral invasion extending to the orbit |
|  | Case of granulomatosis with polyangiitis (Wegener's granulomatosis) manifested with asymptomatic intracerebral hemorrhage |
|  | Is chronic lymphocytic inflammation with pontine perivascular enhancement responsive to steroids (CLIPPERS) in children the same condition as in adults? |
|  | Limbic Encephalitis Associated with Human Herpesvirus-7 (HHV-7) in an Immunocompetent Adult: The First Reported Case in Japan |
|  | Central nervous system relapse in patients with diffuse large B cell lymphoma: analysis of the risk factors and proposal of a new prognostic model |
|  | Intractable Nausea Due to the Area Postrema Syndrome of Neuromyelitis Optica: An Uncommon Cause of a Common Symptom |
|  | Lack of effect on adult and adolescent hypothalamic-pituitary-adrenal axis function with use of fluticasone furoate nasal spray |
|  | [Case of non-convulsive status epilepticus after influenza virus B infection] |
|  | [An adult-onset multiphasic disseminated encephalomyelitis (MDEM) presenting favorable response to steroid therapy] |
|  | Steroid responsive encephalopathy associated with autoimmune thyroiditis following ipilimumab therapy: a case report |
|  | Successful Treatment of Refractory Langerhans Cell Histiocytosis of the Choroid Plexus in a Child With Pulse Dexamethasone and Lenalidomide |
|  | Kikuchi-Fujimoto disease (histiocytic necrotizing lymphadenitis) with atypical encephalitis and painful testitis: a case report |
|  | [Steroid responsive anti-Hu-associated paraneoplastic encephalitis with bilateral frontal lobe lesions] |
|  | Oxidative stress induced by lipid peroxidation is related with inflammation of demyelination and neurodegeneration in multiple sclerosis |
|  | [Case of CNS-limited ANCA-associated vasculitis presenting as recurrent ischemic stroke] |
|  | [Anti-NMDA receptor antibody-positive meningoencephalitis with SIADH and CNS demyelination: A case report] |
|  | Sudden onset of sleep due to hypothalamic lesions in neuromyelitis optica spectrum disorder positive for anti-aquaporin-4 antibody |
|  | Hypertensive emergency preceding the progression of periaortitis and retroperitoneal fibrosis: case report and review of the literature |
|  | Sixth nerve palsy associated with obstruction in Dorello's canal, accompanied by nodular type muscular sarcoidosis |
|  | Progressive multifocal leukoencephalopathy and black fungus in a patient with rheumatoid arthritis without severe lymphocytopenia |
|  | Cerebral vascular findings in PAPA syndrome: cerebral arterial vasculopathy or vasculitis and a posterior cerebral artery dissecting aneurysm |
|  | Medial medullary infarction caused by antineutrophil cytoplasmic antibody-related vasculitis: Case report and review of the literature |
|  | Mycophenolate mofetil and deflazacort combination in neuropsychiatric lupus: a decade of experience from a tertiary care teaching hospital in southern India |
|  | [Giant cell arteritis: Genetic and epigenetic aspects] |
|  | Subacute cognitive deterioration with high serum anti-thyroid peroxidase antibodies: two cases and a plea for pragmatism |
|  | [Orbital apex syndrome without MRI lesion caused by ANCA-associated vasculitis] |
|  | Meningoencephalitis with secondary obstructive hydrocephalus caused by probable coccidioides species in a buff-cheeked gibbon (Nomascus gabriellae) |
|  | Inhaled fluticasone furoate/vilanterol does not affect hypothalamic-pituitary-adrenal axis function in adolescent and adult asthma: randomised, double-blind, placebo-controlled study |
|  | Mn (III) tetrakis (4-benzoic acid) porphyrin scavenges reactive species, reduces oxidative stress, and improves functional recovery after experimental spinal cord injury in rats: comparison with methylprednisolone |
|  | [A Case of Severe Peripheral Polyneuropathy Occurring after Entecavir Treatment in a Hepatitis B Patient] |
|  | Putative paraneoplastic pemphigus and myasthenia gravis in a cat with a lymphocytic thymoma |
|  | Posterior reversible encephalopathy syndrome in a patient with mixed connective tissue disease: a case report |
|  | Corticosteroid treatment buys time in case of a newly diagnosed hypophysitis with visual deterioration |
|  | Vascular complications of fungal meningitis attributed to injections of contaminated methylprednisolone acetate |
|  | IgG4-related hypophysitis presenting as a pituitary adenoma with systemic disease |
|  | Use of a clinicoradiological score to determine the presurgical diagnosis of autoimmune hypophysitis in a teenage girl |
|  | Paraneoplastic brainstem encephalitis in a patient with exceptionally long course of a metastasized neuroendocrine rectum neoplasm |
|  | [An usual case of vasculo- and neuro-Behçet's disease with MEFV mutations] |
|  | Clinically mild encephalitis/encephalopathy with a reversible splenial lesion associated with febrile urinary tract infection |
|  | Successful treatment of subdural hemorrhage and retinal hemorrhage in childhood-onset systemic lupus erythematosus associated with thrombocytopenia: Case report |
|  | Acute disseminated encephalomyelitis (ADEM) following a H3N3 parainfluenza virus infection in a pregnant asthmatic woman with respiratory failure |
|  | Everolimus in immunosuppressive treatment after kidney transplantation in a patient with tuberous sclerosis: case report |
|  | Gastric perforation and critical illness polyneuropathy after steroid treatment in a patient with encephalitis/encephalopathy with transient splenial lesion |
|  | Myelin Oligodendrocyte Glycoprotein-Associated Pediatric Central Nervous System Demyelination: Clinical Course, Neuroimaging Findings, and Response to Therapy |
|  | Simultaneous presentation of acute disseminated encephalomyelitis (ADEM) and systemic lupus erythematosus (SLE) after enteroviral infection: can ADEM present as the first manifestation of SLE? |
|  | Restored vision in a young dog following corticosteroid treatment of presumptive hypophysitis |
|  | Infective endocarditis following tumor necrosis factor-α antagonist therapy for management of psoriatic erythroderma: a case report |
|  | [Two patients with progressive multifocal leukoencephalopathy with immune response against JC virus showing good long-term outcome by combination therapy of mefloquine, mirtazapine, and risperidone] |
|  | Hypophysitis due to IgG4-related disease responding to treatment with azathioprine: an alternative to corticosteroid therapy |
|  | Fatal neurological side-effects with necrosis of spinal cord following nelarabine treatment in a child with relapsed T-cell acute lymphoblastic leukemia |
|  | Autoimmune hypophysitis presenting with intracranial multi-organ involvement: three case reports and review of the literature |
|  | Hypoglutamatergic state is associated with reduced cerebral glucose metabolism in anti-NMDA receptor encephalitis: a case report |
|  | High-dose Thiotepa, Busulfan, Cyclophosphamide, and Autologous Stem Cell Transplantation as Upfront Consolidation for Systemic Non-Hodgkin Lymphoma With Synchronous Central Nervous System Involvement |
|  | Methylprednisolone in combination with interferon beta-1a for relapsing-remitting multiple sclerosis (MECOMBIN study): a multicentre, double-blind, randomised, placebo-controlled, parallel-group trial |
|  | Vernet syndrome resulting from varicella zoster virus infection-a very rare clinical presentation of a common viral infection |
|  | Chronic lymphocytic inflammation with pontine perivascular enhancement responsive to steroids (CLIPPERS): A pediatric case report with six year follow-up |
|  | Susac syndrome with prominent dermatological findings and a prompt response to intravenous immunoglobulin, steroids, and rituximab: a case report |
|  | Juvenile polymyositis with unremitting pain and progressive loss of motor and bulbar function on a background of sickle cell disease |
|  | A thyroid storm patient with protracted disturbance of consciousness and reversible lesion in the splenium of corpus callosum: A case report |
|  | []An adult case of mumps-associated encephalitis/encephalopathy successfully treated with steroid pulse therapy] |
|  | [Effect of cerebrolysin on remyelination processes in multiple sclerosis patients in stage of relapse regression] |
|  | Late-onset paradoxical reactions 10 years after treatment for tuberculous meningitis in an HIV-negative patient: a case report |
|  | Cryptococcal meningitis accompanying lymphocytic inflammation predominantly in cerebral deep white matter: a possible manifestation of immune reconstitution inflammatory syndrome |
|  | Rheumatoid meningitis developed in patient with stable rheumatoid arthritis and myasthenia gravis-detailed analysis of intracranial inflammation using flow cytometry |
|  | [A case of anti-neurofascin 155 antibody-positive combined central and peripheral demyelination successfully treated with plasma exchange] |
|  | Visual loss due to optic nerve infarction and central retinal artery occlusion after spine surgery in the prone position: A case report |
|  | Successful treatment of central nervous system PTLD with rituximab and cranial radiotherapy |
|  | [A case of anti-cyclic citrullinated peptides antibody positive rheumatoid meningitis without arthritis at the onset of neurological symptoms] |
|  | Central nervous system relapse of diffuse large B-cell lymphoma in the rituximab era: results of the UK NCRI R-CHOP-14 versus 21 trial |
|  | Unilateral central retinal artery occlusion as the sole presenting sign of Susac syndrome in a young man: case report |
|  | Effects of addition of rituximab to chemotherapy on central nervous system events in patients with diffuse large B-cell lymphoma |
|  | [Herpes simplex encephalitis presenting as stroke-like symptoms with atypical MRI findings and lacking cerebrospinal fluid pleocytosis] |
|  | [Herpes simplex encephalitis without cerebrospinal fluid pleocytosis in a patient with bullous pemphigoid: a case report] |
|  | [Case of intravascular large B-cell lymphoma (IVLBCL) with central nervous system symptoms diagnosed by renal biopsy] |
|  | [A case of relapsing encephalitis positive for gamma aminobutyric acid (GABA)(A) receptor antibody associated with Type B3 thymoma] |
|  | Combined treatment of methylprednisolone pulse and memantine hydrochloride prompts recovery from neurological dysfunction and cerebral hypoperfusion in carbon monoxide poisoning: a case report |
|  | [Case report: a case of Epstein-Barr virus associated hemophagocytic syndrome with reversible leukoencephalopathy in the splenium of the corpus callosum] |
|  | [A case of myeloperoxidase anti-neutrophil cytoplasmic antibody (MPO-ANCA)-associated hypertrophic pachymeningitis presenting with multiple cranial nerve palsies and diabetes insipidus] |
|  | [Case of neuromyelitis optica spectrum disorder associated with central pontine and extrapontine myelinolysis preceded by syndrome of inappropriate antidiuretic hormone secretion] |
|  | Encephalopathic Susac's Syndrome associated with livedo racemosa in a young woman before the completion of family planning |
|  | [A case of Staphylococcus aureus meningitis associated with cryoglobulin-related renal failure and clinically mild encephalitis/encephalopathy with a reversible splenial lesion] |
|  | [A case of chronic lymphocytic inflammation with pontine perivascular enhancement responsive to steroids (CLIPPERS) with the largest lesion in the temporal lobe] |
|  | [A case of recurrent optic neuritis associated with cerebral and spinal cord lesions and autoantibodies against myelin oligodendrocyte glycoprotein relapsed after fingolimod therapy] |
|  | [A case of recurrent myelitis associated with anti-myelin oligodendrocyte glycoprotein antibody that developed only as localized short spinal cord lesions] |
|  | [Two cases of acute onset of focal cortical reflex myoclonus following acute aseptic meningoencephalitis with positive anti-glutamate receptor autoantibody] |
|  | [Chronic lymphocytic inflammation with pontine perivascular enhancement responsive to steroids (CLIPPERS) associated with swelling in the brainstem: a case report] |
|  | Focal idiopathic hypertrophic pachymeningitis: a case with two separate sites involved at a 5-year interval |
|  | Stevens Johnson syndrome--an adverse drug reaction occurred after uncomplicated removal of an intracerebral cavernous hemangioma |
|  | Advantage of 11C-methionine positron emission tomography for assessing IgG4-related central nervous system lesions |
|  | Recovery from an acute relapse is associated with changes in motor resting-state connectivity in multiple sclerosis |
|  | Unilateral transplantation of human primary fetal tissue in four patients with Huntington's disease: NEST-UK safety report ISRCTN no 36485475 |
|  | Vasculitis with superior ophthalmic vein thrombosis compatible with neuro-neutrophilic disease |
|  | Solitary cavernous sinus neurosarcoidosis mimicking neurosyphilis |
|  | Paraneoplastic ganglioradiculoneuritis in a cat with a plasma cell tumour |
|  | Teaching neuroimages: infant with glutaric aciduria type 1 presenting with infantile spasms and hypsarrhythmia |
|  | Parry-Romberg syndrome with ipsilateral hemipons involvement presenting as monoplegic ataxia |
|  | A Recurrent Case of Ischemic Stroke Caused by Vasospasm due to Giant Cell Arteritis |
|  | Speculating on Kleine-Levin Syndrome mechanisms |
|  | Cerebral Venous Thrombosis in Behçet's Disease Patients Compared to Other Causes of Cerebral Venous Thrombosis: a Retrospective Study |
|  | A case of anti-N-methyl-d-aspartate receptor encephalitis with multiple sclerosis-like demyelinated lesions |
|  | A Unique Case of Encephalopathy with an Elevated IgG-4 and Extremely High Interleukin-6 Level and Delayed Myelodysplastic Syndrome |
|  | Intravascular Lymphoma in the CNS: Options for Treatment |
|  | Weston-Hurst syndrome: a rare fulminant form of acute disseminated encephalomyelitis (ADEM) |
|  | A case of primary dural lymphoma: diffuse large B-cell type |
|  | [Progression of right internal carotid artery stenosis in ischemic stroke patient with autoimmune polyglandular syndrome: A case report] |
|  | The AAV-mediated and RNA-guided CRISPR/Cas9 system for gene therapy of DMD and BMD |
|  | Interstitial Keratitis, Vertigo, and Vasculitis: Typical Cogan's Syndrome |
|  | Bilateral optic neuritis in a patient with Behçet's disease who respond to therapeutic plasma exchange |
|  | Antineutrophil cytoplasmic antibody-negative pauci-immune glomerulonephritis with massive intestinal bleeding |
|  | Feline hyperaesthesia syndrome with self-trauma to the tail: retrospective study of seven cases and proposal for an integrated multidisciplinary diagnostic approach |
|  | Rapidly progressive dementia due to neurosarcoidosis |
|  | Cerebral neurocysticercosis mimicking or comorbid with episodic migraine? |
|  | Cabazitaxel: a novel drug for hormone-refractory prostate cancer |
|  | Combined use of 18 F-FDG PET and corticosteroid for diagnosis of deep-seated primary central nervous system lymphoma without histopathological confirmation |
|  | Primary biliary cirrhosis and hepatic sarcoidosis--a case report |
|  | A prospective study of dexamethasone therapy in refractory epileptic encephalopathy with continuous spike-and-wave during sleep |
|  | Co-existence of renovascular hypertension, polyarteritis nodosa, antiphospholipid syndrome and methylenetetrahydrofolate reductase mutation |
|  | Evidence-based guideline: treatment of parenchymal neurocysticercosis: report of the Guideline Development Subcommittee of the American Academy of Neurology |
|  | The role of magnetic resonance imaging and visual evoked potential in management of optic neuritis |
|  | ANCA-Negative Granulomatosis with Polyangiitis Presenting with Hypertrophic Cranial Pachymeningitis, Abducens Nerve Palsy, and Stenosis of the Internal Carotid Artery |
|  | Spontaneous resolution of Pneumocystis jirovecii pneumonia on high-resolution computed tomography in a patient with renal cell carcinoma |
|  | A case of membranous nephropathy as a manifestation of graft-versus-host disease |
|  | A rare case of Cytomegalovirus, Scedosporium apiospermum and Mycobacterium tuberculosis in a renal transplant recipient |
|  | EMP1, EMP 2, and EMP3 as novel therapeutic targets in human cancer |
|  | Treatment and comorbidities of multiple sclerosis in an employed population in Japan: analysis of health claims data |
|  | [A case of neurolymphomatosis that was diagnosed by acoustic nerve biopsy] |
|  | West Syndrome: A Review and Guide for Paediatricians |
|  | Infection-associated decrease of serum creatine kinase levels in Fukuyama congenital muscular dystrophy |
|  | Influenza-associated MOG antibody-positive longitudinally extensive transverse myelitis: a case report |
|  | [Clinical, epidemiological and etiological studies of adult aseptic meningitis: Report of 11 cases with varicella zoster virus meningitis] |
|  | Atypical acute retinal necrosis accompanied by Terson's syndrome: a case report |
|  | AQP4-IgG autoimmunity in Japan and Germany: Differences in clinical profiles and prognosis in seropositive neuromyelitis optica spectrum disorders |
| Budesonide | Inhaled budesonide protects against chronic asthma-induced neuroinflammation in mouse brain |
|  | Pharmacokinetics of Budesonide Administered with Surfactant in Premature Lambs: Implications for Neonatal Clinical Trials |
|  | Early Inhaled Budesonide for the Prevention of Bronchopulmonary Dysplasia |
|  | Safety of long-term high-volume sinonasal budesonide irrigations for chronic rhinosinusitis |
|  | Opportunity and challenges of nasal powders: Drug formulation and delivery |
|  | Safety analysis of long-term budesonide nasal irrigations in patients with chronic rhinosinusitis post endoscopic sinus surgery |
|  | The safety and efficacy of short-term budesonide delivered via mucosal atomization device for chronic rhinosinusitis without nasal polyposis |
|  | Safety of long-term intranasal budesonide delivered via the mucosal atomization device for chronic rhinosinusitis |
|  | Validated assay for the simultaneous determination of cortisol and budesonide in human plasma using ultra high performance liquid chromatography-tandem mass spectrometry |
|  | The protective effect of different airway humidification liquids to lung after tracheotomy in traumatic brain injury: The role of pulmonary surfactant protein-A (SP-A) |
|  | Impact of study design on the evaluation of inhaled and intranasal corticosteroids' effect on hypothalamic-pituitary-adrenal axis function, part I: general overview of HPA axis study design |
|  | Interventions for preventing high altitude illness: Part 1. Commonly-used classes of drugs |
|  | Chronic asthma results in cognitive dysfunction in immature mice |
|  | A 55-Year-Old Woman With Frequent Pulmonary Exacerbations and Endobronchial Lesions |
|  | Long-Term Cortisol Concentration in Scalp Hair of Asthmatic Children Using Inhaled Corticosteroids: A Case-Control Study |
|  | Chronic asthma-induced behavioral and hippocampal neuronal morphological changes are concurrent with BDNF, cofilin1 and Cdc42/RhoA alterations in immature mice |
|  | Itraconazole and inhaled fluticasone causing hypothalamic-pituitary-adrenal axis suppression in adults with cystic fibrosis |
|  | Effects of fastigial nucleus electrostimulation on airway inflammation and remodeling in an experimental rat model of asthma |
|  | Inhalation toxicity of soman vapor in non-anesthetized rats: a preliminary assessment of inhaled bronchodilator or steroid therapy |
| Meclofenamic acid | Carcinoma-astrocyte gap junctions promote brain metastasis by cGAMP transfer |
|  | m(6)A RNA Methylation Regulates the Self-Renewal and Tumorigenesis of Glioblastoma Stem Cells |
|  | Effects of meclofenamic acid on limbic epileptogenesis in mice kindling models |
|  | Gap Junction Blockers: An Overview of their Effects on Induced Seizures in Animal Models |
|  | Electrophysiological behavior of neonatal astrocytes in hippocampal stratum radiatum |
|  | Electrical synapses connect a network of gonadotropin releasing hormone neurons in a cichlid fish |
|  | Purinergic receptor- and gap junction-mediated intercellular signalling as a mechanism of heterosynaptic metaplasticity |
|  | Anatomical and functional gonadotrope networks in the teleost pituitary |
|  | Meclofenamate causes loss of cellular tethering and decoupling of functional networks in glioblastoma |
|  | The projective field of retinal bipolar cells and its modulation by visual context |
|  | Inhibition of striatal cholinergic interneuron activity by the Kv7 opener retigabine and the nonsteroidal anti-inflammatory drug diclofenac |
|  | Visual circuit development requires patterned activity mediated by retinal acetylcholine receptors |
|  | Opening of astrocytic mitochondrial ATP-sensitive potassium channels upregulates electrical coupling between hippocampal astrocytes in rat brain slices |
|  | Retinal gap junctions are involved in rhythmogenesis of neuronal activity at remote locations - Study on infra-slow oscillations in the rat olivary pretectal nucleus |
|  | Modifications of diflunisal and meclofenamate carboxyl groups affect their allosteric effects on GABAA receptor ligand binding |
| Nimesulide | Biomimetic synthesis of proline-derivative templated mesoporous silica for increasing the brain distribution of diazepam and improving the pharmacodynamics of nimesulide |
|  | Isomeric iodinated analogs of nimesulide: Synthesis, physicochemical characterization, cyclooxygenase-2 inhibitory activity, and transport across Caco-2 cells |
|  | Quantification of curcumin, demethoxycurcumin, and bisdemethoxycurcumin in rodent brain by UHPLC/ESI-Q-TOF-MS/MS after intra-nasal administration of curcuminoids loaded PNIPAM nanoparticles |
|  | Effects of selective and non-selective cyclooxygenase inhibition against neurological deficit and brain oedema following closed head injury in mice |
|  | Piroxicam confer neuroprotection in Cerebral Ischemia by inhibiting Cyclooxygenases, Acid- Sensing Ion Channel-1a and Aquaporin-4: an in silico comparison with Aspirin and Nimesulide |
|  | Nonsteroidal anti-inflammatory drugs modulate cellular glycosaminoglycan synthesis by affecting EGFR and PI3K signaling pathways |
|  | Influence of NSAIDs and methotrexate on CD73 expression and glioma cell growth |
|  | Cerebrovascular Injury After Serial Exposure to Chronic Stress and Abstinence from Methamphetamine Self-Administration |
|  | Cyclooxygenase-2 inhibitors differentially attenuate pentylenetetrazol-induced seizures and increase of pro- and anti-inflammatory cytokine levels in the cerebral cortex and hippocampus of mice |
|  | Reactive astrocyte COX2-PGE2 production inhibits oligodendrocyte maturation in neonatal white matter injury |
|  | Protective effect of losartan and ramipril against stress induced insulin resistance and related complications: Anti-inflammatory mechanisms |
|  | Prostaglandin Signaling Governs Spike Timing-Dependent Plasticity at Sensory Synapses onto Mouse Spinal Projection Neurons |
|  | The use of a gene expression signature and connectivity map to repurpose drugs for bipolar disorder |
|  | Motor learning in common marmosets: vestibulo-ocular reflex adaptation and its sensitivity to inhibitors of Purkinje cell long-term depression |
|  | A Critical Period in Purkinje Cell Development Is Mediated by Local Estradiol Synthesis, Disrupted by Inflammation, and Has Enduring Consequences Only for Males |
|  | Risk of ischemic stroke and the use of individual non-steroidal anti-inflammatory drugs: A multi-country European database study within the SOS Project |
| Celecoxib | Obsessive-Compulsive Disorder: Advances in Diagnosis and Treatment |
|  | Cyclooxygenases and the cardiovascular system |
|  | Celecoxib attenuates systemic lipopolysaccharide-induced brain inflammation and white matter injury in the neonatal rats |
|  | Celecoxib reduces brain dopaminergic neuronaldysfunction, and improves sensorimotor behavioral performance in neonatal rats exposed to systemic lipopolysaccharide |
|  | Inflammation and depression: a causal or coincidental link to the pathophysiology? |
|  | Celecoxib-induced gastrointestinal, liver and brain lesions in rats, counteraction by BPC 157 or L-arginine, aggravation by L-NAME |
|  | Destruction of vasculogenic mimicry channels by targeting epirubicin plus celecoxib liposomes in treatment of brain glioma |
|  | Aspirin and celecoxib may help to rectify a neurotransmission imbalance in bipolar disorder |
|  | In Vivo Brain Imaging, Biodistribution, and Radiation Dosimetry Estimation of [(11)C]Celecoxib, a COX-2 PET Ligand, in Nonhuman Primates |
|  | Celecoxib and omega-3 fatty acids alone and in combination with risperidone affect the behavior and brain biochemistry in amphetamine-induced model of schizophrenia |
|  | Simvastatin Augmentation for Patients With Early-Phase Schizophrenia-Spectrum Disorders: A Double-Blind, Randomized Placebo-Controlled Trial |
|  | Celecoxib-induced inhibition of neurogenesis in fetal frontal cortex is attenuated by curcumin via Wnt/β-catenin pathway |
|  | Gene expression profiling of brain endothelial cells after experimental subarachnoid haemorrhage |
|  | Identification of Celecoxib-Targeted Proteins Using Label-Free Thermal Proteome Profiling on Rat Hippocampus |
|  | Adjunctive celecoxib for schizophrenia: A meta-analysis of randomized, double-blind, placebo-controlled trials |
|  | Pharmacological modulation of brain activity in a preclinical model of osteoarthritis |
|  | Celecoxib Ameliorates Seizure Susceptibility in Autosomal Dominant Lateral Temporal Epilepsy |
|  | Celecoxib Decrease Seizures Susceptibility in a Rat Model of Inflammation by Inhibiting HMGB1 Translocation |
|  | New celecoxib multiparticulate systems to improve glioblastoma treatment |
|  | Celecoxib increases SMN and survival in a severe spinal muscular atrophy mouse model via p38 pathway activation |
|  | Premetastatic soil and prevention of breast cancer brain metastasis |
|  | Influence of Cyclooxygenase-2 Inhibitors on Kynurenic Acid Production in Rat Brain in Vitro |
|  | Sub-chronic celecoxib prevents soluble beta amyloid-induced depressive-like behaviour in rats |
|  | Celecoxib Prevents Cognitive Impairment and Neuroinflammation in Soluble Amyloid β-treated Rats |
|  | Celecoxib Alleviates Memory Deficits by Downregulation of COX-2 Expression and Upregulation of the BDNF-TrkB Signaling Pathway in a Diabetic Rat Model |
|  | Synthesis and characterization of celecoxib derivatives as possible anti-inflammatory, analgesic, antioxidant, anticancer and anti-HCV agents |
|  | Inflammation, Obsessive-Compulsive Disorder, and Related Disorders |
|  | Celecoxib With Neoadjuvant Chemotherapy for Breast Cancer Might Worsen Outcomes Differentially by COX-2 Expression and ER Status: Exploratory Analysis of the REMAGUS02 Trial |
|  | Synergistic effects of celecoxib and bupropion in a model of chronic inflammation-related depression in mice |
|  | Celecoxib suppresses the phosphorylation of STAT3 protein and can enhance the radiosensitivity of medulloblastoma-derived cancer stem-like cells |
|  | Celecoxib inhibits proliferation and survival of chronic myelogeous leukemia (CML) cells via AMPK-dependent regulation of β-catenin and mTORC1/2 |
|  | Cyclooxygenase 2 inhibitor celecoxib inhibits glutamate release by attenuating the PGE2/EP2 pathway in rat cerebral cortex endings |
|  | Nonsteroidal anti-inflammatory drugs diclofenac and celecoxib attenuates Wnt/β-catenin/Tcf signaling pathway in human glioblastoma cells |
|  | Efficacy of adjunctive celecoxib treatment for patients with major depressive disorder: a meta-analysis |
|  | Neoadjuvant irinotecan, cisplatin, and concurrent radiation therapy with celecoxib for patients with locally advanced esophageal cancer |
|  | Celecoxib attenuates depressive-like behavior associated with immunological liver injury in C57BL/6 mice through TNF-α and NF-κb dependent mechanisms |
|  | Simultaneous pulmonary administration of celecoxib and naringin using a nebulization-friendly nanoemulsion: A device-targeted delivery for treatment of lung cancer |
|  | Combined treatment with celecoxib and sevoflurane after global cerebral ischaemia has no additive neuroprotective effects in rats |
|  | Tocotrienols: The promising analogues of vitamin E for cancer therapeutics |
|  | Transport rankings of non-steroidal antiinflammatory drugs across blood-brain barrier in vitro models |
|  | Effects of celecoxib on hematoma and edema volumes in primary intracerebral hemorrhage: a multicenter randomized controlled trial |
|  | Dynamic regulation of P-glycoprotein in human brain capillaries |
|  | Vascular brain-derived neurotrophic factor pathway in rats with adjuvant-induced arthritis: Effect of anti-rheumatic drugs |
|  | The cytokine model of schizophrenia: emerging therapeutic strategies |
|  | Bipolar Disorder and Immune Dysfunction: Epidemiological Findings, Proposed Pathophysiology and Clinical Implications |
|  | COX2/PTGS2 Expression Is Predictive of Response to Neoadjuvant Celecoxib in HER2-negative Breast Cancer Patients |
|  | Effect of celecoxib and L-NAME on global ischemia-reperfusion injury in the rat hippocampus |
|  | Immunomodulatory effect of Celecoxib on HMGB1/TLR4 pathway in a recurrent seizures model in immature rats |
|  | COX-2 drives metastatic breast cells from brain lesions into the cerebrospinal fluid and systemic circulation |
|  | Combination treatment of celecoxib and ciprofloxacin attenuates live S. aureus induced oxidative damage and inflammation in murine microglia via regulation of cytokine balance |
|  | 2,5-Dimethyl Celecoxib Inhibits Proliferation and Cell Cycle and Induces Apoptosis in Glioblastoma by Suppressing CIP2A/PP2A/Akt Signaling Axis |
|  | Inflamed moods: a review of the interactions between inflammation and mood disorders |
|  | Staged anticonvulsant screening for chronic epilepsy |
|  | Are Non-steroidal Anti-Inflammatory Drugs Clinically Suitable for the Treatment of Symptoms in Depression-Associated Inflammation? |
|  | Glutamate-mediated upregulation of the multidrug resistance protein 2 in porcine and human brain capillaries |
|  | Analysis of Post-Traumatic Brain Injury Gene Expression Signature Reveals Tubulins, Nfe2l2, Nfkb, Cd44, and S100a4 as Treatment Targets |
|  | Efficacy of celecoxib add-on treatment for immuno-metabolic depression: Protocol of the INFLAMED double-blind placebo-controlled randomized controlled trial |
|  | Erythrocyte membrane-encapsulated celecoxib improves the cognitive decline of Alzheimer's disease by concurrently inducing neurogenesis and reducing apoptosis in APP/PS1 transgenic mice |
|  | Combination of an agonistic anti-CD40 monoclonal antibody and the COX-2 inhibitor celecoxib induces anti-glioma effects by promotion of type-1 immunity in myeloid cells and T-cells |
|  | A phase I study of sirolimus in combination with metronomic therapy (CHOAnome) in children with recurrent or refractory solid and brain tumors |
|  | Intrinsic Resistance to 5-Fluorouracil in a Brain Metastatic Variant of Human Breast Cancer Cell Line, MDA-MB-231BR |
|  | Neuroprotective potential of solanesol in a combined model of intracerebral and intraventricular hemorrhage in rats |
|  | Assessment of radioligands for PET imaging of cyclooxygenase-2 in an ischemic neuronal injury model |
|  | Acquired heterotopic ossification in hips and knees following encephalitis: case report and literature review |
|  | Blocking COX-2 induces apoptosis and inhibits cell proliferation via the Akt/survivin- and Akt/ID3 pathway in low-grade-glioma |
|  | Long-term outcome of the REMAGUS 02 trial, a multicenter randomised phase II trial in locally advanced breast cancer patients treated with neoadjuvant chemotherapy with or without celecoxib or trastuzumab according to HER2 status |
|  | PET measurement of cyclooxygenase-2 using a novel radioligand: upregulation in primate neuroinflammation and first-in-human study |
|  | COXIBs and 2,5-dimethylcelecoxib counteract the hyperactivated Wnt/β-catenin pathway and COX-2/PGE2/EP4 signaling in glioblastoma cells |
|  | Randomized phase II adjuvant factorial study of dose-dense temozolomide alone and in combination with isotretinoin, celecoxib, and/or thalidomide for glioblastoma |
|  | Evidence for new targets and synergistic effect of metronomic celecoxib/fluvastatin combination in pilocytic astrocytoma |
|  | Radiosynthesis and in vivo evaluation of [(11)C]MOV as a PET imaging agent for COX-2 |
|  | A multicenter randomized phase II study of sequential epirubicin/cyclophosphamide followed by docetaxel with or without celecoxib or trastuzumab according to HER2 status, as primary chemotherapy for localized invasive breast cancer patients |
|  | OSU-03012 and Viagra Treatment Inhibits the Activity of Multiple Chaperone Proteins and Disrupts the Blood-Brain Barrier: Implications for Anti-Cancer Therapies |
|  | Indomethacin induced gene regulation in the rat hippocampus |
|  | CUSP9* treatment protocol for recurrent glioblastoma: aprepitant, artesunate, auranofin, captopril, celecoxib, disulfiram, itraconazole, ritonavir, sertraline augmenting continuous low dose temozolomide |
|  | Radiation therapy and concurrent topotecan followed by maintenance triple anti-angiogenic therapy with thalidomide, etoposide, and celecoxib for pediatric diffuse intrinsic pontine glioma |
|  | Identification and modification of amyloid-independent phenotypes of APOE4 mice |
|  | The effect of some immunomodulatory and anti-inflammatory drugs on Li-pilocarpine-induced epileptic disorders in Wistar rats |
|  | Isomeric iodinated analogs of nimesulide: Synthesis, physicochemical characterization, cyclooxygenase-2 inhibitory activity, and transport across Caco-2 cells |
|  | Effects of adjunctive inflammatory modulation on IL-1β in treatment resistant bipolar depression |
|  | Drug Targets for Cardiovascular-Safe Anti-Inflammatory: In Silico Rational Drug Studies |
|  | Dysfunction of thermoregulation contributes to the generation of hyperthermia-induced seizures |
|  | Pharmacological characterization of intraplantar Complete Freund's Adjuvant-induced burrowing deficits |
|  | Effect of maternal immune activation on the kynurenine pathway in preadolescent rat offspring and on MK801-induced hyperlocomotion in adulthood: amelioration by COX-2 inhibition |
|  | Potent, orally available, selective COX-2 inhibitors based on 2-imidazoline core |
|  | Epinephrine promotes COX-2-dependent immune suppression in myeloid cells and cancer tissues |
|  | Wnt/β-catenin pathway regulates MGMT gene expression in cancer and inhibition of Wnt signalling prevents chemoresistance |
|  | Interactive involvement of hippocampal cAMP/PKA and cyclooxygenase-2 signaling pathways in spatial learning in the Morris water maze |
|  | Hyperalgesic and hypoalgesic mechanisms evoked by the acute administration of CCL5 in mice |
|  | Acceptable cardiac safety profile of neoadjuvant 5-fluorouracil, epirubicin, cyclophosphamide and celecoxib (FEC-C) for breast cancer: a subanalysis of biomarkers for cardiac injury |
|  | Drugs and Scaffold That Inhibit Cytochrome P450 27A1 In Vitro and In Vivo |
|  | The ER stress inducer DMC enhances TRAIL-induced apoptosis in glioblastoma |
|  | COX-2 is involved in ET-1-induced hypertrophy of neonatal rat cardiomyocytes: role of NFATc3 |
|  | Cyclooxygenase-2 Inhibition Limits Angiotensin II-Induced DNA Oxidation and Protein Nitration in Humans |
|  | COX inhibitors and bone: A safer impact on osteoblasts by NO-releasing NSAIDs |
|  | Cyclooxygenase-2 inhibitors differentially attenuate pentylenetetrazol-induced seizures and increase of pro- and anti-inflammatory cytokine levels in the cerebral cortex and hippocampus of mice |
|  | Sustained Complete Response to Metronomic Chemotherapy in a Child with Refractory Atypical Teratoid Rhabdoid Tumor: A Case Report |
|  | Induction of COX-2-PGE2 synthesis by activation of the MAPK/ERK pathway contributes to neuronal death triggered by TDP-43-depleted microglia |
|  | Regulated expression of PTPRJ by COX-2/PGE2 axis in endothelial cells |
|  | Nebulizable colloidal nanoparticles co-encapsulating a COX-2 inhibitor and a herbal compound for treatment of lung cancer |
|  | Efficacy of anti-inflammatory agents to improve symptoms in patients with schizophrenia: an update |
|  | Neuropathic and inflammatory antinociceptive effects and electrocortical changes produced by Salvia divinorum in rats |
|  | Investigations on 16-Arylideno Steroids as a New Class of Neuroprotective Agents for the Treatment of Alzheimer's and Parkinson's Diseases |
|  | Comparison of pharmacological and genetic inhibition of cyclooxygenase-2: effects on adult neurogenesis in the hippocampal dentate gyrus |
|  | Single circulating tumor cell detection and overall survival in nonmetastatic breast cancer |
|  | Vascular KCNQ (Kv7) potassium channels as common signaling intermediates and therapeutic targets in cerebral vasospasm |
|  | Cadmium-induced IL-6 and IL-8 expression and release from astrocytes are mediated by MAPK and NF-κB pathways |
|  | Metronomic Four-Drug Regimen Has Anti-tumor Activity in Pediatric Low-Grade Glioma; The Results of a Phase II Clinical Trial |
|  | Studies on 16,17-Pyrazoline Substituted Heterosteroids as Anti-Alzheimer and Anti-Parkinsonian Agents Using LPS Induced Neuroinflammation Models of Mice and Rats |
|  | Endocannabinoids, through opioids and prostaglandins, contribute to fever induced by key pyrogenic mediators |
|  | Clinical benefit in recurrent glioblastoma from adjuvant NovoTTF-100A and TCCC after temozolomide and bevacizumab failure: a preliminary observation |
|  | Plasma Exosomes and Improvements in Endothelial Function by Angiotensin 2 Type 1 Receptor or Cyclooxygenase 2 Blockade following Intermittent Hypoxia |
|  | Preclinical pharmacokinetics, tissue distribution and excretion studies of a novel anti-candidal agent-thiosemicarbazide derivative of isoniazid (TSC-INH) by validated UPLC-MS/MS assay |
|  | Avian influenza A H7N9 virus induces severe pneumonia in mice without prior adaptation and responds to a combination of zanamivir and COX-2 inhibitor |
|  | Allogeneic Natural Killer and Cytomegalovirus (CMV)-pp65 Pulsed Dendritic Cells Induced Complete Response Through 15 Months in a Patient with Recurrent Glioblastoma: A Case Study |
|  | Panax ginseng exerts antidepressant-like effects by suppressing neuroinflammatory response and upregulating nuclear factor erythroid 2 related factor 2 signaling in the amygdala |
|  | The inhibition of 2-arachidonoyl-glycerol (2-AG) biosynthesis, rather than enhancing striatal damage, protects striatal neurons from malonate-induced death: a potential role of cyclooxygenase-2-dependent metabolism of 2-AG |
| Etodolac | High-Throughput Screening for Identification of Blood-Brain Barrier Integrity Enhancers: A Drug Repurposing Opportunity to Rectify Vascular Amyloid Toxicity |
|  | Expression of tissue inhibitor of metalloproteinases and matrix metalloproteinases in the ischemic brain of photothrombosis model mice |
|  | Perioperative inhibition of β-adrenergic and COX2 signaling in a clinical trial in breast cancer patients improves tumor Ki-67 expression, serum cytokine levels, and PBMCs transcriptome |
|  | Reducing liver metastases of colon cancer in the context of extensive and minor surgeries through β-adrenoceptors blockade and COX2 inhibition |
|  | Stress impairs the efficacy of immune stimulation by CpG-C: Potential neuroendocrine mediating mechanisms and significance to tumor metastasis and the perioperative period |
| Fenbufen | Synthesis and Biological Evaluation of an (18)Fluorine-Labeled COX Inhibitor--[(18)F]Fluorooctyl Fenbufen Amide--For Imaging of Brain Tumors |
| Mesalamine | Post-Infectious Irritable Bowel Syndrome |
|  | Invasive Fungal Rhinosinusitis with Orbital Apex Syndrome Leading to Brain Abscess in a Patient with Ulcerative Colitis |
|  | Pre-clinical toxicity of a combination of berberine and 5-aminosalicylic acid in mice |
|  | Crohn's disease: is there a place for neurological screening? |
|  | Comparison between 5-aminosalicylic acid (5-ASA) and para-aminosalicylic acid (4-PAS) as potential protectors against Mn-induced neurotoxicity |
|  | Unusual Case of Cerebral Venous Thrombosis in Patient with Crohn's Disease |
|  | Addition of Berberine to 5-Aminosalicylic Acid for Treatment of Dextran Sulfate Sodium-Induced Chronic Colitis in C57BL/6 Mice |
|  | Histologically confirmed case of cerebral vasculitis associated with Crohn's disease--a case report |
|  | Pharmacokinetics in Wistar Rats of 5-[(4-Carboxybutanoyl)Amino]-2-Hydroxybenzoic Acid: A Novel Synthetic Derivative of 5-Aminosalicylic Acid (5-ASA) with Possible Anti-Inflammatory Activity |
| Nabumetone | Determining the Molecular Pathways Underlying the Protective Effect of Non-Steroidal Anti-Inflammatory Drugs for Alzheimer's Disease: A Bioinformatics Approach |
| Rofecoxib | Prostanoid receptor EP2 as a therapeutic target |
|  | Cyclooxygenase- and cytochrome P450-derived eicosanoids in stroke |
|  | Synergistical neuroprotection of rofecoxib and statins against malonic acid induced Huntington's disease like symptoms and related cognitive dysfunction in rats |
|  | Assessment of radioligands for PET imaging of cyclooxygenase-2 in an ischemic neuronal injury model |
|  | Combined therapy with COX-2 inhibitor and 20-HETE inhibitor reduces colon tumor growth and the adverse effects of ischemic stroke associated with COX-2 inhibition |
|  | In vitro screen of a small molecule inhibitor drug library identifies multiple compounds that synergize with oncolytic myxoma virus against human brain tumor-initiating cells |
|  | COXIBs and 2,5-dimethylcelecoxib counteract the hyperactivated Wnt/β-catenin pathway and COX-2/PGE2/EP4 signaling in glioblastoma cells |
|  | Nonsteroidal anti-inflammatory drug choice and adverse outcomes in clopidogrel users: A retrospective cohort study |
|  | Role of angiotensin-(1-7) in gastroprotection against stress-induced ulcerogenesis. The involvement of mas receptor, nitric oxide, prostaglandins, and sensory neuropeptides |
|  | Risk of ischemic stroke and the use of individual non-steroidal anti-inflammatory drugs: A multi-country European database study within the SOS Project |
| Sulfasalazine | Neurology of inflammatory bowel disease |
|  | Sulfasalazine maintains blood-brain barrier integrity and relieves lipopolysaccharide-induced inflammation in hCMEC/D3 cells |
|  | Quantification and Metabolite Identification of Sulfasalazine in Mouse Brain and Plasma Using Quadrupole-Time-of-Flight Mass Spectrometry |
|  | Convection-enhanced delivery of sulfasalazine prolongs survival in a glioma stem cell brain tumor model |
|  | Sulfasalazine treatment can cause a positive effect on LPS-induced endotoxic rats |
|  | Sulfasalazine impacts on ferroptotic cell death and alleviates the tumor microenvironment and glioma-induced brain edema |
|  | Sulfasalazine and temozolomide with radiation therapy for newly diagnosed glioblastoma |
|  | Characteristics of sulfasalazine-induced cytotoxicity in C6 rat glioma cells |
|  | Sulfasalazine augments a pro-inflammatory response in interleukin-1β-stimulated amniocytes and myocytes |
|  | Seizures and gliomas--towards a single therapeutic approach |
|  | Central-Variant Posterior Reversible Encephalopathy due to Sulfasalazine: A Case Report |
|  | Behavioural Effects of Using Sulfasalazine to Inhibit Glutamate Released by Cancer Cells: A Novel target for Cancer-Induced Depression |
|  | Use of cassette dosing approach to examine the effects of P-glycoprotein on the brain and cerebrospinal fluid concentrations in wild-type and P-glycoprotein knockout rats |
|  | Potential of sulfasalazine as a therapeutic sensitizer for CD44 splice variant 9-positive urogenital cancer |
|  | Combination Therapy with Sulfasalazine and Valproic Acid Promotes Human Glioblastoma Cell Death Through Imbalance of the Intracellular Oxidative Response |
|  | Glutamate Excitotoxicity Linked to Spermine Oxidase Overexpression |
|  | [Glutamate and malignant gliomas, from epilepsia to biological aggressiveness: therapeutic implications] |
|  | Cystine/glutamate antiporter blockage induces myelin degeneration |
|  | Effects of Sulphasalazine in Cerebral Ischemia Reperfusion Injury in Rat |
|  | PET Imaging with [(18)F]FSPG Evidences the Role of System xc(-) on Brain Inflammation Following Cerebral Ischemia in Rats |
|  | The role of system Xc(-) in methamphetamine-induced dopaminergic neurotoxicity in mice |
|  | Attenuated effects of Neu2000 on hypoxia-induced synaptic activities in a rat hippocampus |
|  | Transport of BMAA into Neurons and Astrocytes by System x(c) |
|  | In vitro modeling of HIV proviral activity in microglia |
|  | Requirement of NF-kappa B Activation in Different Mice Brain Areas during Long-Term Memory Consolidation in Two Contextual One-Trial Tasks with Opposing Valences |
|  | Inhibition of IKKβ Reduces Ethanol Consumption in C57BL/6J Mice |
|  | Reduction of Neuropathic and Inflammatory Pain through Inhibition of the Tetrahydrobiopterin Pathway |
|  | Behavioral assessment of acute inhibition of system xc (-) in rats |
|  | Temozolomide toxicity operates in a xCT/SLC7a11 dependent manner and is fostered by ferroptosis |
|  | A phase 1-2, prospective, double blind, randomized study of the safety and efficacy of Sulfasalazine for the treatment of progressing malignant gliomas: study protocol of [ISRCTN45828668] |
|  | Administration of Wasabia koreana Ameliorates Irritable Bowel Syndrome-Like Symptoms in a Zymosan-Induced Mouse Model |
|  | FGF-2 induces neuronal death through upregulation of system xc- |
|  | NFκB signaling is essential for the lipopolysaccharide-induced increase of type 2 deiodinase in tanycytes |
|  | Protein degradation by ubiquitin-proteasome system in formation and labilization of contextual conditioning memory |
|  | Dynamic (68)Ga-DOTATATE PET/MRI in the Diagnosis and Management of Intracranial Meningiomas |
|  | Increased Expression of System xc- in Glioblastoma Confers an Altered Metabolic State and Temozolomide Resistance |
|  | The EGF Receptor Promotes the Malignant Potential of Glioma by Regulating Amino Acid Transport System xc(-) |
|  | Opposite in vivo effects of agents that stimulate or inhibit the glutamate/cysteine exchanger system xc- on the inhibition of hippocampal LTP by Aß |
|  | Neuroprotection Promoted by Guanosine Depends on Glutamine Synthetase and Glutamate Transporters Activity in Hippocampal Slices Subjected to Oxygen/Glucose Deprivation |
|  | Experimental Reactivation of Pulmonary Mycobacterium avium Complex Infection in a Modified Cornell-Like Murine Model |
|  | Mn Inhibits GSH Synthesis via Downregulation of Neuronal EAAC1 and Astrocytic xCT to Cause Oxidative Damage in the Striatum of Mice |
|  | SLC7A11 expression is associated with seizures and predicts poor survival in patients with malignant glioma |
|  | High-Throughput Assay Development for Cystine-Glutamate Antiporter (xc-) Highlights Faster Cystine Uptake than Glutamate Release in Glioma Cells |
|  | Dietary polyacetylenes of the falcarinol type are inhibitors of breast cancer resistance protein (BCRP/ABCG2) |
|  | Calcineurin phosphatase as a negative regulator of fear memory in hippocampus: control on nuclear factor-κB signaling in consolidation and reconsolidation |
|  | Safety and Optimal Neuroprotection of neu2000 in acute Ischemic stroke with reCanalization: study protocol for a randomized, double-blinded, placebo-controlled, phase-II trial |
| Zomepirac | Characterization of aldo-keto reductase 1C subfamily members encoded in two rat genes (akr1c19 and RGD1564865). Relationship to 9-hydroxyprostaglandin dehydrogenase |
| Dexamethasone | Dexamethasone treatment for the acute respiratory distress syndrome: a multicentre, randomised controlled trial |
|  | Dexamethasone and supportive care with or without whole brain radiotherapy in treating patients with non-small cell lung cancer with brain metastases unsuitable for resection or stereotactic radiotherapy (QUARTZ): results from a phase 3, non-inferiority, randomised trial |
|  | Trial of Dexamethasone for Chronic Subdural Hematoma |
|  | Dexamethasone Administration and Mortality in Patients with Brain Abscess: A Systematic Review and Meta-Analysis |
|  | A Refill for the Brain Mineralocorticoid Receptor: The Benefit of Cortisol Add-On to Dexamethasone Therapy |
|  | Postnatal Corticosteroids to Prevent or Treat Bronchopulmonary Dysplasia |
|  | Dexamethasone and the brain at age 18 years: randomize the first baby--and follow-up |
|  | Brain Transcriptome Responses to Dexamethasone Depending on Dose and Sex Reveal Factors Contributing to Sex-Specific Vulnerability to Stress-Induced Disorders |
|  | Influence of Dexamethasone on O-(2-[(18)F]-Fluoroethyl)-L-Tyrosine Uptake in the Human Brain and Quantification of Tumor Uptake |
|  | Multiple Antenatal Dexamethasone Treatment Alters Brain Vessel Differentiation in Newborn Mouse Pups |
|  | Dexamethasone for the treatment of traumatic brain injured patients with brain contusions and pericontusional edema: Study protocol for a prospective, randomized and double blind trial |
|  | Hydrogel-mediated local delivery of dexamethasone reduces neuroinflammation after traumatic brain injury |
|  | Dexamethasone-related adrenal insufficiency in patients with brain and skull base tumours |
|  | Dexamethasone protects neonatal hypoxic-ischemic brain injury via L-PGDS-dependent PGD2-DP1-pERK signaling pathway |
|  | Advances in the Prevention and Treatment of High Altitude Illness |
|  | Dexamethasone inhibits brain apoptosis in mice with eosinophilic meningitis caused by Angiostrongylus cantonensis infection |
|  | Dexamethasone increases production of C-type natriuretic peptide in the sheep brain |
|  | Dexamethasone exacerbates cerebral edema and brain injury following lithium-pilocarpine induced status epilepticus |
|  | Dexamethasone reduces brain cell apoptosis and inhibits inflammatory response in rats with intracerebral hemorrhage |
|  | Association between postnatal dexamethasone for treatment of bronchopulmonary dysplasia and brain volumes at adolescence in infants born very preterm |
|  | Effects of progesterone vs. dexamethasone on brain oedema and inflammatory responses following experimental brain resection |
|  | Dexamethasone-induced acute excitotoxic cell death in the developing brain |
|  | Dexamethasone alleviates tumor-associated brain damage and angiogenesis |
|  | Development of L-carnosine functionalized iron oxide nanoparticles loaded with dexamethasone for simultaneous therapeutic potential of blood brain barrier crossing and ischemic stroke treatment |
|  | Dexamethasone Alters the Appetite Regulation via Induction of Hypothalamic Insulin Resistance in Rat Brain |
|  | Neonatal dexamethasone treatment exacerbates hypoxic-ischemic brain injury |
|  | Dexamethasone increased the survival rate in Plasmodium berghei-infected mice |
|  | Antenatal Dexamethasone Treatment Induces Sex-dependent Upregulation of NTPDase1/CD39 and Ecto-5'-nucleotidase/CD73 in the Rat Fetal Brain |
|  | Dexamethasone Attenuates the Enhanced Rewarding Effects of Cocaine Following Experimental Traumatic Brain Injury |
|  | Inhibition of proteasomal glucocorticoid receptor degradation restores dexamethasone-mediated stabilization of the blood-brain barrier after traumatic brain injury |
|  | Dexamethasone induces apoptosis of progenitor cells in the subventricular zone and dentate gyrus of developing rat brain |
|  | Tissue Lipidomic Alterations Induced by Prolonged Dexamethasone Treatment |
|  | Spinal cord brain-derived neurotrophic factor levels increase after dexamethasone treatment in male rats with chronic inflammation |
|  | Antenatal low-intensity pulsed ultrasound reduces neurobehavioral deficits and brain injury following dexamethasone-induced intrauterine growth restriction |
|  | Management der akuten Meningitis und Enzephalitis: ein klinischer Leitfaden |
|  | Dexamethasone-mediated oncogenicity in vitro and in an animal model of glioblastoma |
|  | Dexamethasone suppresses JMJD3 gene activation via a putative negative glucocorticoid response element and maintains integrity of tight junctions in brain microvascular endothelial cells |
|  | Dexamethasone for chronic subdural haematoma: a systematic review and meta-analysis |
|  | The Hypothalamic-Pituitary-Adrenal Axis and the Fetus |
|  | Neonatal Dexamethasone Treatment Exacerbates Hypoxia/Ischemia-Induced White Matter Injury |
|  | Dexamethasone potentiates in vitro blood-brain barrier recovery after primary blast injury by glucocorticoid receptor-mediated upregulation of ZO-1 tight junction protein |
|  | Dexamethasone-induced neuroprotection in hypoxic-ischemic brain injury in newborn rats is partly mediated via Akt activation |
|  | Effects of Neonatal Dexamethasone Exposure on Adult Neuropsychiatric Traits in Rats |
|  | Melatonin attenuates dexamethasone-induced spatial memory impairment and dexamethasone-induced reduction of synaptic protein expressions in the mouse brain |
|  | Combination therapy with melatonin and dexamethasone in a mouse model of traumatic brain injury |
|  | Neuroprotective effect of magnesium sulfate and dexamethasone on intrauterine ischemia in the fetal rat brain: ultrastructural evaluation |
|  | [Influence of dexamethasone on the expression of immediate early genes c-fos and c-jun in different regions of the neonatal brain] |
|  | Dexamethasone-Mediated Upregulation of Calreticulin Inhibits Primary Human Glioblastoma Dispersal Ex Vivo |
|  | Dexamethasone Chemotherapy Does Not Disrupt Orexin Signaling |
|  | Dexamethasone-induced leukocytosis is associated with poor survival in newly diagnosed glioblastoma |
|  | A Dexamethasone-regulated Gene Signature Is Prognostic for Poor Survival in Glioblastoma Patients |
|  | The relationship between corticosteroids and symptoms in patients with primary brain tumors: utility of the Dexamethasone Symptom Questionnaire-Chronic |
|  | NK1 receptor antagonists and dexamethasone as anticancer agents in vitro and in a model of brain tumours secondary to breast cancer |
|  | Dexamethasone retrodialysis attenuates microglial response to implanted probes in vivo |
|  | Intrathecal dexamethasone therapy for febrile infection-related epilepsy syndrome |
|  | Dexamethasone-Loaded, PEGylated, Vertically Aligned, Multiwalled Carbon Nanotubes for Potential Ischemic Stroke Intervention |
|  | Hypothalamic-pituitary-adrenal axis hyperactivity and brain differences in healthy women |
|  | Time-dependent effects of dexamethasone plasma concentrations on glucocorticoid receptor challenge tests |
|  | Use of Glucocorticoids for the Fetus and Preterm Infant |
|  | In utero exposure to dexamethasone causes a persistent and age-dependent exacerbation of the neurotoxic effects and glia activation induced by MDMA in dopaminergic brain regions of C57BL/6J mice |
|  | Short review of SEC, a potential dexamethasone-sparing regimen for glioblastoma: Spironolactone, ecallantide, clotrimazole |
|  | Antenatal dexamethasone before asphyxia promotes cystic neural injury in preterm fetal sheep by inducing hyperglycemia |
|  | Fetal hypoxia increases vulnerability of hypoxic-ischemic brain injury in neonatal rats: role of glucocorticoid receptors |
|  | Glucocorticoids for the prevention of cerebral edema in traumatic brain injury: mission (im)possible? |
|  | Dexamethasone-mediated inhibition of Glioblastoma neurosphere dispersal in an ex vivo organotypic neural assay |
|  | Does growth impairment underlie the adverse effects of dexamethasone on development of noradrenergic systems? |
|  | Hippo/YAP signaling pathway mitigates blood-brain barrier disruption after cerebral ischemia/reperfusion injury |
|  | Enhancing Continuous Online Microdialysis Using Dexamethasone: Measurement of Dynamic Neurometabolic Changes during Spreading Depolarization |
|  | Clinical practice: Acute high-altitude illnesses |
|  | Dexamethasone Inhibits Copper-Induced Alpha-Synuclein Aggregation by a Metallothionein-Dependent Mechanism |
|  | Efficacy and safety of modafinil versus dexamethasone in cancer-related fatigue: a prospective randomized controlled study |
|  | Dexamethasone suppresses the locomotor response of neonatal rats to novel environment |
|  | Effects of Dexamethasone in the Treatment of Recurrent Chronic Subdural Hematoma |
|  | Influence of blood-brain barrier permeability on O-(2-(18)F-fluoroethyl)-L-tyrosine uptake in rat gliomas |
|  | Pharmacological mitigation of tissue damage during brain microdialysis |
|  | Multimodal analysis of the effects of dexamethasone on high-altitude cerebral oedema: protocol for a pilot study |
|  | Glucocorticoids Protect Neonatal Rat Brain in Model of Hypoxic-Ischemic Encephalopathy (HIE) |
|  | Neonatal dexamethasone accelerates spreading depression in the rat, and antioxidant vitamins counteract this effect |
|  | The effects of dexamethasone on post-asphyxial cerebral oxygenation in the preterm fetal sheep |
|  | Effect of dexamethasone administered with magnesium sulfate on inflammation-mediated degradation of the blood-brain barrier using an in vitro model |
|  | Seizure and Fever |
|  | Anoxia ameliorates the dexamethasone-induced neurobehavioral alterations in the neonatal male rat pups |
|  | Dexamethasone facilitates fear extinction and safety discrimination in PTSD: A placebo-controlled, double-blind study |
|  | Prenatal dexamethasone augments the neurobehavioral teratology of chlorpyrifos: significance for maternal stress and preterm labor |
|  | Effects of dexamethasone on the Li-pilocarpine model of epilepsy: protection against hippocampal inflammation and astrogliosis |
|  | Development of a safe dexamethasone-eluting electrode array for cochlear implantation |
|  | Dexamethasone Treatment Leads to Enhanced Fear Extinction and Dynamic Fkbp5 Regulation in Amygdala |
|  | Endogenous Glucocorticoid Response to Single-Dose Dexamethasone for Croup in Children: A Pharmacodynamic Study |
|  | Antenatal dexamethasone after asphyxia increases neural injury in preterm fetal sheep |
|  | Communication from the periphery to the hypothalamus through the blood-brain barrier: An in vitro platform |
|  | Lymphomatoid Granulomatosis with Central Nervous System Involvement Successfully Treated with Cyclophosphamide, High-dose Cytarabine, Dexamethasone, Etoposide, and Rituximab (CHASER therapy) Followed by Brain Irradiation: A Case Study |
|  | A brain populated with space-occupying lesions: identifying the culprit |
|  | Dexamethasone-suppressed Salivary Cortisol and Pain Sensitivity in Female Twins |
|  | Effects of sustained release dexamethasone hydrogels in hearing preservation cochlear implantation |
|  | Effects of dexamethasone on quality of recovery following vaginal surgery: a randomized trial |
|  | Effect of intratympanic dexamethasone administration on cisplatin-induced ototoxicity in adult guinea pigs |
|  | Astroglial Plasticity Is Implicated in Hippocampal Remodelling in Adult Rats Exposed to Antenatal Dexamethasone |
|  | Dexamethasone PONV prophylaxis alters the hypothalamic-pituitary-adrenal axis after transsphenoidal pituitary surgery |
|  | A comparison of the sexually dimorphic dexamethasone transcriptome in mouse cerebral cortical and hypothalamic embryonic neural stem cells |
|  | Nebulized dexamethasone sodium phosphate in the treatment of horses with severe asthma |
|  | Post-dexamethasone cortisol, self-inflicted injury, and suicidal ideation among depressed adolescent girls |
|  | Dexamethasone diffusion across contact lenses is inhibited by Staphylococcus epidermidis biofilms in vitro |
|  | [Acute Mountain Sickness and High-Altitude Cerebral Edema] |
|  | Dexamethasone otoprotection in a multidose cisplatin ototoxicity mouse model |
|  | In vivo induction of P-glycoprotein expression at the mouse blood-brain barrier: an intracerebral microdialysis study |
|  | Effects of dexamethasone on intracochlear inflammation and residual hearing after cochleostomy: A comparison of administration routes |
|  | Neonatal Dexamethasone Treatment Suppresses Hippocampal Estrogen Receptor α Expression in Adolescent Female Rats |
|  | A prospective study of dexamethasone therapy in refractory epileptic encephalopathy with continuous spike-and-wave during sleep |
|  | Fatigue scores in patients with brain metastases receiving whole brain radiotherapy |
|  | Dexamethasone in outcome of patients with hepatitis B virus-related acute-on-chronic liver failure |
|  | The lateral habenula is a common target of cocaine and dexamethasone |
|  | Regression of Recurrent High-Grade Glioma with Temozolomide, Dexamethasone, and Levetiracetam: Case Report and Review of the Literature |
|  | Glucocorticoids and fetal programming part 1: Outcomes |
|  | Antagonistic interactions between dexamethasone and fluoxetine modulate morphodynamics and expression of cytokines in astrocytes |
|  | Dexamethasone-induced insulin resistance: kinetic modeling using novel PET radiopharmaceutical 6-deoxy-6-[(18)F]fluoro-D-glucose |
|  | Time dependent neuroprotection of dexamethasone in experimental focal cerebral ischemia: The involvement of NF-κB pathways |
|  | Statins prevent adverse effects of postnatal glucocorticoid therapy on the developing brain in rats |
|  | Cochlear and Vestibular Effects of Combined Intratympanic Gentamicin and Dexamethasone |
|  | Effects of dexamethasone and meloxicam on Borrelia burgdorferi-induced inflammation in glial and neuronal cells of the central nervous system |
|  | Late gestational exposure to dexamethasone and fetal programming of abnormal behavior in Wistar Kyoto rats |
|  | Brain microvascular endothelial-astrocyte cell responses following Japanese encephalitis virus infection in an in vitro human blood-brain barrier model |
|  | Dexamethasone eluting electrodes for cochlear implantation: Effect on residual hearing |
|  | Glucocorticoid receptor represses brain-derived neurotrophic factor expression in neuron-like cells |
|  | Toll-like receptor expression in the blood and brain of patients and a mouse model of Parkinson's disease |
|  | Single-shot bevacizumab for cerebral radiation injury |
|  | Kinetics of functionalised carbon nanotube distribution in mouse brain after systemic injection: Spatial to ultra-structural analyses |
|  | Prenatal dexamethasone, as used in preterm labor, worsens the impact of postnatal chlorpyrifos exposure on serotonergic pathways |
|  | Effects of intratympanic dexamethasone on noise-induced hearing loss: An experimental study |
|  | Melatonin pretreatment prevented the effect of dexamethasone negative alterations on behavior and hippocampal neurogenesis in the mouse brain |
|  | Dexamethasone exposure and memory function in adult survivors of childhood acute lymphoblastic leukemia: A report from the SJLIFE cohort |
|  | Role of Aspirin and Dexamethasone against Experimentally Induced Depression in Rats |
|  | l-DOPA as a small molecule surrogate to promote angiogenesis and prevent dexamethasone-induced ischemia |
|  | The Sustained-Exposure Dexamethasone Formulation OTO-104 Offers Effective Protection against Cisplatin-Induced Hearing Loss |
|  | Regulation of Multidrug Resistance P-Glycoprotein in the Developing Blood-Brain Barrier: Interplay between Glucocorticoids and Cytokines |
|  | Dexamethasone induced changes of neural activity in the auditory cortex of rats |
|  | Neuronal Fos-like immunoreactivity associated with dexamethasone-induced hypertension in rats and effects of glucagon-like peptide-2 |
|  | Efficacy of dexamethasone on penicillin-induced epileptiform activity in rats: an electrophysiological study |
|  | Dexamethasone-induced activation of heat shock response ameliorates seizure susceptibility and neuroinflammation in mouse models of Lafora disease |
|  | The Sustained-Exposure Dexamethasone Formulation OTO-104 Offers Effective Protection against Noise-Induced Hearing Loss |
|  | Dexamethasone induces heat shock response and slows down disease progression in mouse and fly models of Huntington's disease |
|  | Prenatal dexamethasone augments the sex-selective developmental neurotoxicity of chlorpyrifos: implications for vulnerability after pharmacotherapy for preterm labor |
|  | Characterization of persistent and recurrent Cushing's disease |
|  | Reply to letter: "Dexamethasone for chronic subdural haematoma: a systematic review and meta-analysis" |
|  | Expression and glucocorticoid-dependent regulation of the stress-inducible protein DRR1 in the mouse adult brain |
|  | Comment on 'Dexamethasone exerts profound immunologic interference on treatment efficacy for recurrent glioblastoma' |
|  | Dexamethasone loaded nanoparticles exert protective effects against Cisplatin-induced hearing loss by systemic administration |
|  | [Gliomas – What I Have to Know in ten Questions] |
|  | How to measure glucocorticoid receptor's sensitivity in patients with stress-related psychiatric disorders |
|  | Opportunity and challenges of nasal powders: Drug formulation and delivery |
|  | Corticosteroids compromise survival in glioblastoma |
|  | Dexamethasone enhances necrosis-like neuronal death in ischemic rat hippocampus involving μ-calpain activation |
|  | Linezolid and dexamethasone experience in a serious case of listeria rhombencephalitis |
|  | Purity and stability of the membrane-limited glucocorticoid receptor agonist dexamethasone-BSA |
|  | Pneumococcal meningitis: clinical-pathological correlations (MeninGene-Path) |
|  | Dexamethasone acts as a radiosensitizer in three astrocytoma cell lines via oxidative stress |
|  | Re-exposure to the hypobaric hypoxic brain injury of high altitude: plasma S100B levels and the possible effect of acclimatisation on blood-brain barrier dysfunction |
|  | A single high dose of dexamethasone increases GAP-43 and synaptophysin in the hippocampus of aged rats |
|  | Caffeine neuroprotects against dexamethasone-induced anxiety-like behaviour in the Zebrafish (Danio rerio) |
|  | Knockout of 5-lipoxygenase prevents dexamethasone-induced tau pathology in 3xTg mice |
|  | The effect of vitamin B6 on dexamethasone-induced depression in mice model of despair |
|  | Brain-specific homeobox factor as a target selector for glucocorticoid receptor in energy balance |
|  | Neuroimmune Response Mediated by Cytokines in Natural Scrapie after Chronic Dexamethasone Treatment |
|  | No effects of hydrocortisone and dexamethasone on pain sensitivity in healthy individuals |
|  | Combined dexamethasone suppression-corticotrophin-releasing hormone stimulation test in medication-free major depression and healthy volunteers |
|  | Inner ear delivery of dexamethasone using injectable silk-polyethylene glycol (PEG) hydrogel |
|  | Genetic variation in the glucocorticoid receptor and psychopathology after dexamethasone administration in cardiac surgery patients |
|  | Dexamethasone prevents motor deficits and neurovascular damage produced by shiga toxin 2 and lipopolysaccharide in the mouse striatum |
|  | Epac2a-knockout mice are resistant to dexamethasone-induced skeletal muscle atrophy and short-term cold stress |
|  | The Effect of Dexamethasone on Symptoms of Posttraumatic Stress Disorder and Depression After Cardiac Surgery and Intensive Care Admission: Longitudinal Follow-Up of a Randomized Controlled Trial |
|  | Cochlear Transcriptome Following Acoustic Trauma and Dexamethasone Administration Identified by a Combination of RNA-seq and DNA Microarray |
|  | Pathophysiology, clinical presentation, and treatment of coma and acute kidney injury complicating falciparum malaria |
|  | Dexamethasone's effect in the retrocochlear auditory centers of a noise-induced hearing loss mouse model |
|  | Chronic dexamethasone treatment results in hippocampal neurons injury due to activate NLRP1 inflammasome in vitro |
|  | A review of associated controversies surrounding glucocorticoid use in veterinary emergency and critical care |
|  | [Clinical utility of the liposteroid therapy: Potential effects on the macrophage activation] |
|  | Research Resource: The Dexamethasone Transcriptome in Hypothalamic Embryonic Neural Stem Cells |
|  | Dexamethasone induces different morphological changes in the dorsal and ventral hippocampus of rats |
|  | Prenatal exposure to dexamethasone in the mouse induces sex-specific differences in placental gene expression |
|  | Glia- and tissue-specific changes in the Kynurenine Pathway after treatment of mice with lipopolysaccharide and dexamethasone |
|  | Differential targeting of brain stress circuits with a selective glucocorticoid receptor modulator |
|  | The dexamethasone corticotropin releasing hormone test in healthy and depressed women with and without childhood adversity |
|  | Antenatal betamethasone compared with dexamethasone (betacode trial): a randomized controlled trial |
|  | Antiglucocorticoid and related treatments for psychosis |
|  | Maternal treatment with glucocorticoids modulates gap junction protein expression in the ovine fetal brain |
|  | A Propensity Score Analysis of the Impact of Dexamethasone Use on Delayed Cerebral Ischemia and Poor Functional Outcomes After Subarachnoid Hemorrhage |
|  | Development of the blood-brain barrier within the paraventricular nucleus of the hypothalamus: influence of fetal glucocorticoid excess |
|  | Effect of both local and systemically administered dexamethasone on long-term hearing and tissue response in a Guinea pig model of cochlear implantation |
|  | Low-dose dexamethasone treatment promotes the pro-survival signalling pathway in the adult rat prefrontal cortex |
|  | Expression of Rasd1 in mouse endocrine pituitary cells and its response to dexamethasone |
|  | Hypothalamic pituitary adrenal axis and prolactin abnormalities in suicidal behavior |
|  | Pharmacokinetics of dexamethasone and nefopam administered alone or in combination using a newly developed prefilled multi-drug injector in rats |
|  | Neural Correlates to the Increase in Maximal Force after Dexamethasone Administration |
|  | Exposure to dexamethasone reduces pituitary volume and gonadotropic cell number in rat fetuses |
|  | Dexamethasone administration to the neonatal rat results in neurological dysfunction at the juvenile stage even at low doses |
|  | HPA axis and aging in depression: systematic review and meta-analysis |
|  | The glucocorticoid analog dexamethasone alters the expression and the distribution of dopamine receptors and enkephalin within cortico-subcortical regions |
|  | Subcutaneous pulsatile glucocorticoid replacement therapy |
|  | Bevacizumab for the Treatment of Gammaknife Radiosurgery-Induced Brain Radiation Necrosis |
|  | Validating self-report and proxy reports of the Dexamethasone Symptom Questionnaire -Chronic for the evaluation of longer-term corticosteroid toxicity |
|  | Co-Therapy of Albendazole and Dexamethasone Reduces Pathological Changes in the Cerebral Parenchyma of Th-1 and Th-2 Dominant Mice Heavily Infected with Angiostrongylus cantonensis: Histopathological and RNA-seq Analyses |
|  | Melatonin attenuates dexamethasone toxicity-induced oxidative stress, calpain and caspase activation in human neuroblastoma SH-SY5Y cells |
|  | Dexamethasone exposure affects paraventricular nucleus and pituitary corticotrophs in female rat fetuses: An unbiased stereological and immunohistochemical study |
|  | Combined application of dexamethasone and hyperbaric oxygen therapy yields better efficacy for patients with delayed encephalopathy after acute carbon monoxide poisoning |
|  | Depressive-like phenotype induced by prenatal dexamethasone in mice is reversed by desipramine |
|  | Histological studies of neuroprotective effects of Curcuma longa Linn. on neuronal loss induced by dexamethasone treatment in the rat hippocampus |
|  | Adrenal steroidogenesis following prenatal dexamethasone exposure in the spiny mouse |
|  | Single neonatal dexamethasone administration has long-lasting outcome on depressive-like behaviour, Bdnf, Nt-3, p75ngfr and sorting receptors (SorCS1-3) stress reactive expression |
|  | A Randomized Controlled Trial Studying the Role of Dexamethasone in Scalp Nerve Blocks for Supratentorial Craniotomy |
|  | Antenatal dexamethasone exposure differentially affects distinct cortical neural progenitor cells and triggers long-term changes in murine cerebral architecture and behavior |
|  | Neurochemical control of rapid stress-induced changes in brain aromatase activity |
|  | Different corticosteroids and regimens for accelerating fetal lung maturation for women at risk of preterm birth |
|  | Effect of dexamethasone intravitreal implant on blood glucose, hypothalamic-pituitary-adrenal axis function and vascular endothelial growth factor serum levels in patients with diabetic macular oedema |
|  | Aseptic meningitis in relapsing polychondritis: a case report and literature review |
|  | Maternal dexamethasone exposure inhibits the gonadotropin-releasing hormone neuronal movement in the preoptic area of rat offspring |
|  | Dexamethasone and levetiracetam reduce hetero-cellular gap-junctional coupling between F98 glioma cells and glial cells in vitro |
|  | Dexamethasone Rescues an Acute High-Fat Diet-Induced Decrease in Human Growth Hormone Gene Expression in Male Partially Humanized CD-1 Mice |
|  | Prenatal exposure to dexamethasone disturbs sex-determining gene expression and fetal testosterone production in male embryos |
|  | Microdialysis in the rat striatum: effects of 24 h dexamethasone retrodialysis on evoked dopamine release and penetration injury |
|  | Dexamethasone rescues neurovascular unit integrity from cell damage caused by systemic administration of shiga toxin 2 and lipopolysaccharide in mice motor cortex |
|  | Inhibition of Hippocampal Regeneration by Adjuvant Dexamethasone in Experimental Infant Rat Pneumococcal Meningitis |
|  | Low-functional programming of the CREB/BDNF/TrkB pathway mediates cognitive impairment in male offspring after prenatal dexamethasone exposure |
|  | Dexamethasone Is One of the Factors Minimizing the Inner Ear Damage from Electrode Insertion in Cochlear Implantation |
|  | A Single Postnatal Dose of Dexamethasone Enhances Memory of Rat Pups Later in Life |
|  | Pro-inflammatory and anti-inflammatory compounds exert similar effects on P-glycoprotein in blood-brain barrier endothelial cells |
|  | Effects of Prenatal Dexamethasone on the Rat Pituitary Gland and Gonadotropic Cells in Female Offspring |
|  | Mold-templated inorganic-organic hybrid supraparticles for codelivery of drugs |
|  | Protective effects of maternal nutritional supplementation with lactoferrin on growth and brain metabolism |
|  | Upregulation of nucleoside triphosphate diphosphohydrolase-1 and ecto-5'-nucleotidase in rat hippocampus after repeated low-dose dexamethasone administration |
|  | The effect of amifostine on pathophysiological changes in vasogenic brain edema induced by an experimental cold injury model |
|  | Dexamethasone administration during definitive radiation and temozolomide renders a poor prognosis in a retrospective analysis of newly diagnosed glioblastoma patients |
|  | A single high dose of dexamethasone affects the phosphorylation state of glutamate AMPA receptors in the human limbic system |
|  | Pre-emptive coinfiltration of dexamethasone palmitate emulsion with ropivacaine for postoperative pain in patients undergoing major spine surgery: a study protocol for a prospective, randomised controlled, multicentre trial |
|  | Lung cancer: Best supportive care - a reasonable option for patients with brain metastases? |
|  | Fluvoxamine moderates reduced voluntary activity following chronic dexamethasone infusion in mice via recovery of BDNF signal cascades |
|  | Antenatal glucocorticoid treatment affects hippocampal development in mice |
|  | End-of-life care in pediatric neuro-oncology |
|  | What is the utility of postoperative antihelminthic therapy after resection for intraventricular neurocysticercosis? |
|  | Probing the association between dexamethasone-induced cortisol suppression and serotonin transporter availability among drug-free patients with major depressive disorder--a small-sample SPECT study with [¹²³I]ADAM |
|  | Effects of Late Gestational Fetal Exposure to Dexamethasone Administration on the Postnatal Hypothalamus-Pituitary-Adrenal Axis Response to Hypoglycemia in Pigs |
|  | The role of glucocorticoid receptors in dexamethasone-induced apoptosis of neuroprogenitor cells in the hippocampus of rat pups |
|  | Retinoids and glucocorticoids have opposite effects on actin cytoskeleton rearrangement in hippocampal HT22 cells |
|  | Multigenerational effects of fetal dexamethasone exposure on the hypothalamic-pituitary-adrenal axis of first- and second-generation female offspring |
|  | Inhibitory effect of matrine on blood-brain barrier disruption for the treatment of experimental autoimmune encephalomyelitis |
|  | A new strategy of CyberKnife treatment system based radiosurgery followed by early use of adjuvant bevacizumab treatment for brain metastasis with extensive cerebral edema |
|  | The role of inflammation on the functionality of intracortical microelectrodes |
|  | Rapid Translation of COVID-19 Preprint Data into Critical Care Practice |
|  | Attenuation of cisplatin ototoxicity by otoprotective effects of nanoencapsulated curcumin and dexamethasone in a guinea pig model |
|  | The role of H3K4me3 and H3K9/14ac in the induction by dexamethasone of Per1 and Sgk1, two glucocorticoid [corrected] early response genes that mediate the effects of acute stress in mammals |
|  | Acute symptomatic peri-lead edema 33 hours after deep brain stimulation surgery: a case report |
|  | Clostridium perfringens epsilon toxin induces permanent neuronal degeneration and behavioral changes |
|  | A fatal case of malignant neurocysticercosis |
|  | Prognostic effect of neutrophil-lymphocyte ratio during concurrrent chemo-radiotherapy period in glioblastoma patients who did not receive dexamethasone |
|  | Steroid responsive encephalopathy associated with autoimmune thyroiditis (SREAT) in childhood |
|  | A Case of Cerebral Fat Embolism |
|  | Dexamethasone rapidly induces a novel ras superfamily member-related gene in AtT-20 cells |
|  | Hyperammonemic encephalopathy in multiple myeloma |
|  | Glucocorticoids attenuate the central sympathoexcitatory actions of insulin |
|  | Antidepressants attenuate the dexamethasone-induced decrease in viability and proliferation of human neuroblastoma SH-SY5Y cells: a involvement of extracellular regulated kinase (ERK1/2) |
|  | LXR agonist rescued the deficit in the proliferation of the cerebellar granule cells induced by dexamethasone |
|  | Macroglossia associated with brainstem injury |
|  | Prenatal drug exposures sensitize noradrenergic circuits to subsequent disruption by chlorpyrifos |
|  | Neonatal amygdala lesions lead to increased activity of brain CRF systems and hypothalamic-pituitary-adrenal axis of juvenile rhesus monkeys |
|  | GR gene BclI polymorphysm changes the path, but not the level, of dexamethasone-induced cortisol suppression |
|  | Zika Virus Infection in Dexamethasone-immunosuppressed Mice Demonstrating Disseminated Infection with Multi-organ Involvement Including Orchitis Effectively Treated by Recombinant Type I Interferons |
|  | The associations between adolescent sleep, diurnal cortisol patterns and cortisol reactivity to dexamethasone suppression test |
|  | Rapid initial response of myeloma hyperammonemic encephalopathy to L-ornithine-L-aspartate and dexamethasone therapy |
|  | Glucocorticoids reset circadian clock in choroid plexus via period genes |
|  | Glucocorticoid Induces Incoordination between Glutamatergic and GABAergic Neurons in the Amygdala |
|  | Prenatal treatment of congenital adrenal hyperplasia: risks outweigh benefits |
|  | Management of hydrocephalus and subdural hygromas in pediatric patients after decompression of Chiari malformation type I: case series and review of the literature |
|  | 16-Substituted steroids alleviate LPS-induced neurodegenerative disorders in rats |
|  | Postnatal glucocorticoid-induced hypomyelination, gliosis, and neurologic deficits are dose-dependent, preparation-specific, and reversible |
|  | Protective effect of dexamethasone on fetal rat skin in experimental intrauterine ischaemia/reperfusion injury |
|  | Corticosteroids effect on caspase 3 expression in an in-vitro model of hypoxic brain cells |
|  | Clinically Mild Encephalitis/Encephalopathy With a Reversible Splenial Lesion Accompanied by Epstein-Barr Virus Hemophagocytic Lymphohistiocytosis: A Case Report and Review of the Literature |
|  | Infrared thermography is an effective, noninvasive measure of HPA activation |
|  | From epidemiology and neurodevelopment to antineoplasticity. Medroxyprogesterone reduces human glial tumor growth in vitro and C6 glioma in rat brain in vivo |
|  | Valproate recovers the inhibitory effect of dexamethasone on the proliferation of the adult dentate gyrus-derived neural precursor cells via GSK-3β and β-catenin pathway |
|  | Diurnal Cortisol Patterns and Dexamethasone Suppression Test Responses in Healthy Young Adults Born Preterm at Very Low Birth Weight |
|  | Anatomical and functional implications of corticotrophin-releasing hormone neurones in a septal nucleus of the avian brain: an emphasis on glial-neuronal interaction via V1a receptors in vitro |
|  | Different regulation of cortisol and corticosterone in the subterranean rodent Ctenomys talarum: Responses to dexamethasone, angiotensin II, potassium, and diet |
|  | Response to: Comment on 'Dexamethasone exerts profound immunologic interference on treatment efficacy for recurrent glioblastoma' |
|  | Molecular Integration of Incretin and Glucocorticoid Action Reverses Immunometabolic Dysfunction and Obesity |
|  | Short-term effect of ovariectomy on measures of insulin sensitivity and response to dexamethasone administration in horses |
|  | Combined early treatment in hemiplegic attacks related to CACNA1A encephalopathy with brain oedema: Blocking the cascade? |
|  | Marked differences in the effect of antiepileptic and cytostatic drugs on the functionality of P-glycoprotein in human and rat brain capillary endothelial cell lines |
|  | Metabolism of 2,2',3,3',6,6'-hexachlorobiphenyl (PCB 136) atropisomers in tissue slices from phenobarbital or dexamethasone-induced rats is sex-dependent |
|  | Retinoic acid increases glucocorticoid receptor phosphorylation via cyclin-dependent kinase 5 |
|  | Vamorolone, a dissociative steroidal compound, reduces pro-inflammatory cytokine expression in glioma cells and increases activity and survival in a murine model of cortical tumor |
|  | Interventions to Reduce Neurological Symptoms in Patients with GBM Receiving Radiotherapy: From Theory to Clinical Practice |
|  | Interventions for preventing high altitude illness: Part 1. Commonly-used classes of drugs |
|  | Adjuvant non-bacteriolytic and anti-inflammatory combination therapy in pneumococcal meningitis: an investigation in a mouse model |
|  | Risk Locus Identification Ties Alcohol Withdrawal Symptoms to SORCS2 |
|  | SGK1/FOXO3 Signaling in Hypothalamic POMC Neurons Mediates Glucocorticoid-Increased Adiposity |
|  | Impedance Changes and Fibrous Tissue Growth after Cochlear Implantation Are Correlated and Can Be Reduced Using a Dexamethasone Eluting Electrode |
|  | The effect of gabapentin premedication on postoperative nausea, vomiting, and pain in patients on preoperative dexamethasone undergoing craniotomy for intracranial tumors |
|  | HPA axis dysregulation in men with hypersexual disorder |
|  | Adrenal dysfunction in portal hypertensive rats with acute hemorrhage |
|  | Impairments in laterodorsal tegmentum to VTA projections underlie glucocorticoid-triggered reward deficits |
|  | Hypothalamic-pituitary-adrenal (HPA) axis suppression after treatment with glucocorticoid therapy for childhood acute lymphoblastic leukaemia |
|  | [Neuropsychiatric manifestations revealing cerebral subarachnoid hemorrhage caused by electrification accident about a case and review of literature] |
|  | Effect of a liposomal hyaluronic acid gel loaded with dexamethasone in a guinea pig model after manual or motorized cochlear implantation |
|  | [Hyperammonemic encephalopathy in multiple myeloma] |
|  | Role of the NMDA receptor in cognitive deficits, anxiety and depressive-like behavior in juvenile and adult mice after neonatal dexamethasone exposure |
|  | Reduction of Glucocorticoid Receptor Function in Chronic Fatigue Syndrome |
|  | Membrane-initiated nuclear trafficking of the glucocorticoid receptor in hypothalamic neurons |
|  | Transient contrast-induced encephalopathy after internal carotid artery embolisation prior to surgery for nasopharyngeal carcinoma |
|  | Glucocorticoid receptor in rat nucleus accumbens: Its roles in propofol addictions |
|  | Role of Ras, ERK, and Akt in glucocorticoid-induced differentiation of embryonic rat somatotropes in vitro |
|  | Successful Treatment of Refractory Langerhans Cell Histiocytosis of the Choroid Plexus in a Child With Pulse Dexamethasone and Lenalidomide |
|  | Glucocorticoid Effects on Cerebellar Development in a Chicken Embryo Model: Exploring Changes in PAX6 and Metalloproteinase-9 After Exposure to Dexamethasone |
|  | MMP9 mRNA is a potential diagnostic and treatment monitoring marker for PTSD: Evidence from mice and humans |
|  | Activation of the glucocorticoid receptor rapidly triggers calcium-dependent serotonin release in vitro |
|  | Absence of CMV viremia in high-grade glioma patients under low dosage glucocorticoid treatment |
|  | No differences between drug naive and drug experienced unipolar depressed patients in terms of neurobiological testing: a cross sectional study |
|  | In search of the HPA axis activity in unipolar depression patients with childhood trauma: Combined cortisol awakening response and dexamethasone suppression test |
|  | Beneficial effect of aspirin against interferon-α-2b-induced depressive behavior in Sprague Dawley rats |
|  | Leukoencephalopathy and long-term neurobehavioural, neurocognitive, and brain imaging outcomes in survivors of childhood acute lymphoblastic leukaemia treated with chemotherapy: a longitudinal analysis |
|  | Physical activity and hypothalamic-pituitary-adrenocortical axis function in adolescents |
|  | Comparative of transcranial magnetic stimulation and other treatments in experimental autoimmune encephalomyelitis |
|  | Dynamics of salivary cortisol in chronic kidney disease patients at stages 1 through 4 |
|  | cDNA sequence, ligand biding, and regulation of galanin/GMAP in mouse brain |
|  | Monitoring Dopamine Responses to Potassium Ion and Nomifensine by in Vivo Microdialysis with Online Liquid Chromatography at One-Minute Resolution |
|  | Glucocorticoids/glucocorticoid receptors effect on dopaminergic neurotransmitters in ADHD rats |
|  | The protective effect of adrenocorticotropic hormone treatment against noise-induced hearing loss |
|  | Characterization of Activation of the Hypothalamic-Pituitary-Adrenal Axis by the Herbicide Atrazine in the Female Rat |
|  | Efficacy of negative feedback in the HPA axis predicts recovery from acute challenges |
|  | Neurosarcoidosis: clinical characteristics, diagnosis, and treatment in eight Chinese patients |
|  | Predominant Neurologic Manifestations Seen in a Patient With a Biallelic Perforin1 Mutation (PRF1; p.R225W) |
|  | Ex vivo glucocorticoid receptor-mediated IL-10 response predicts the course of depression severity |
|  | Nongenomic Glucocorticoid Suppression of a Postsynaptic Potassium Current via Emergent Autocrine Endocannabinoid Signaling in Hypothalamic Neuroendocrine Cells following Chronic Dehydration |
|  | In Vivo Electrochemical Analysis of a PEDOT/MWCNT Neural Electrode Coating |
|  | Hippocampal mitogen-activated protein kinase phosphatase-1 regulates behavioral and systemic effects of chronic corticosterone administration |
|  | Further evidence for a membrane receptor that binds glucocorticoids in the rodent hypothalamus |
|  | Dexamethazone protects against Escherichia coli induced sickness behavior in rats |
|  | 3D RNA-seq: a powerful and flexible tool for rapid and accurate differential expression and alternative splicing analysis of RNA-seq data for biologists |
|  | High altitude illness in pilgrims after rapid ascent to 4380 M |
|  | Neural expression of a novel alternatively spliced and polyadenylated Gs alpha transcript |
|  | CRH/CRHR1 mediates prenatal synthetic glucocorticoid programming of depression-like behavior across 2 generations |
|  | Measurable outcomes for pediatric epileptic encephalopathy: a single-center experience with corticosteroid therapy |
|  | Inflammatory responses in the rat superior colliculus after eye enucleation |
|  | Enhanced steroid dosing reduces seizures during antiparasitic treatment for cysticercosis and early after |
|  | Biocompatibility and Therapeutic Effect of 3 Intra-Tympanic Drug Delivery Vehicles in Acute Acoustic Trauma |
|  | Disseminated aspergillosis in an HIV-positive cannabis user taking steroid treatment |
|  | Characterizing primary human microglia: A comparative study with myeloid subsets and culture models |
|  | Therapeutic efficacy of atypical antipsychotic drugs by targeting multiple stress-related metabolic pathways |
|  | Antidepressant-like effects of Xiaochaihutang in a neuroendocrine mouse model of anxiety/depression |
|  | Baylisascaris procyonis-Associated Meningoencephalitis in a Previously Healthy Adult, California, USA |
|  | Chronic glucocorticoid exposure activates BK-NLRP1 signal involving in hippocampal neuron damage |
|  | Childhood trauma and HPA axis functionality in offspring of bipolar parents |
|  | Diagnosis of brainstem involvement in NK/T cell lymphoma |
|  | Clinical application of DEX/CRH test and multi-channel NIRS in patients with depression |
|  | Social setting, social rank and HPA axis response in cynomolgus monkeys |
|  | Cochlear implant and inflammation reaction: Safety study of a new steroid-eluting electrode |
|  | Steroid-sparing effect of corticorelin acetate in peritumoral cerebral edema is associated with improvement in steroid-induced myopathy |
|  | Glucocorticoid receptor-mediated regulation of Rfrp (GnIH) and Gpr147 (GnIH-R) synthesis in immortalized hypothalamic neurons |
|  | Brainstem meningoencephalitis as a presentation of Behçet disease |
|  | Antidepressant responsiveness in adulthood is permanently impaired after neonatal destruction of the neurogenic pool |
|  | Relative adrenal insufficiency in mice deficient in 5α-reductase 1 |
|  | Extensive clinical experience: Hypothalamic-pituitary-adrenal axis recovery after adrenalectomy for corticotropin-independent cortisol excess |
|  | Neuroinflammation in acute hepatic encephalopathy rats: imaging and therapeutic effectiveness evaluation using (11)C-PK11195 and (18)F-DPA-714 micro-positron emission tomography |
|  | TNF-α blockade suppresses pericystic inflammation following anthelmintic treatment in porcine neurocysticercosis |
|  | Sex differences in the traumatic stress response: the role of adult gonadal hormones |
|  | Targeting metabolism with a ketogenic diet during the treatment of glioblastoma multiforme |
|  | Role of 3-Acetyl-11-Keto-Beta-Boswellic Acid in Counteracting LPS-Induced Neuroinflammation via Modulation of miRNA-155 |
|  | "It's Not a Tumor": A Rare Case of Symptomatic Cerebellar Developmental Venous Anomaly |
|  | Blunted glucocorticoid and mineralocorticoid sensitivity to stress in people with diabetes |
|  | Bevacizumab for symptomatic radiation-induced tumor enlargement in pediatric low grade gliomas |
|  | Combined pharmacological induction of Hsp70 suppresses prion protein neurotoxicity in Drosophila |
|  | HPA axis dysregulation, NR3C1 polymorphisms and glucocorticoid receptor isoforms imbalance in metabolic syndrome |
|  | Corticotropin-releasing hormone improves survival in pneumococcal pneumonia by reducing pulmonary inflammation |
|  | FKBP5 polymorphisms and hypothalamic-pituitary-adrenal axis negative feedback in major depression and obsessive-compulsive disorder |
|  | A Time Limit for Initiating Anti-Inflammatory Treatment for Improved Olfactory Function after Head Injury |
|  | Ring lesions in the brain: a harmless commensal? |
|  | Cortico-cortical projections in mouse visual cortex are functionally target specific |
|  | Maternal Licorice Consumption During Pregnancy and Pubertal, Cognitive, and Psychiatric Outcomes in Children |
|  | Total and free cortisol responses and their relation to outcomes after cardiopulmonary bypass in infants |
|  | Establishment of revised diagnostic cut-offs for adrenal laboratory investigation using the new Roche Diagnostics Elecsys(®) Cortisol II assay |
|  | Lithium protects against glucocorticoid induced neural progenitor cell apoptosis in the developing cerebellum |
|  | Herpes simplex virus encephalitis involving the right thalamus |
|  | HPA axis regulation and epigenetic programming of immune-related genes in chronically stressed and non-stressed mid-life women |
|  | Glutamate and GABA imbalance promotes neuronal apoptosis in hippocampus after stress |
|  | Retinoids and glucocorticoids target common genes in hippocampal HT22 cells |
|  | Sex differences in the traumatic stress response: PTSD symptoms in women recapitulated in female rats |
|  | Behavioral effects of glucocorticoids during the first exposures to the forced swim stress |
|  | Effects of antenatal steroid therapy on neurodevelopment in an IUGR mouse model |
|  | Hypothalamo-pituitary-adrenal axis activity evolves differentially in untreated versus treated multiple sclerosis |
|  | Depression-like phenotype by deletion of α7 nicotinic acetylcholine receptor: Role of BDNF-TrkB in nucleus accumbens |
|  | Miller-Fisher syndrome complicated by Bickerstaff brainstem encephalitis: A case report |
|  | The AMPK-mTOR signaling pathway is involved in regulation of food intake in the hypothalamus of stressed chickens |
|  | Chronic glucocorticoid treatment enhances lipogenic activity in visceral adipocytes of male Wistar rats |
|  | Effect of glucocorticoid-induced insulin resistance on follicle development and ovulation |
|  | Dopaminergic modulation of affective and social deficits induced by prenatal glucocorticoid exposure |
|  | Serum cortisol and adrenocorticotrophic hormone (ACTH) in infants receiving topical and subconjunctival corticosteroids following cataract surgery |
|  | Glucocorticoid use in patients with adrenal insufficiency following administration of the COVID-19 vaccine: a pituitary society statement |
|  | Sex-specific disruption of murine midbrain astrocytic and dopaminergic developmental trajectories following antenatal GC treatment |
|  | Activity of pomalidomide in patients with immunoglobulin light-chain amyloidosis |
|  | Delivery of Hypoxia-Inducible Heme Oxygenase-1 Gene for Site-Specific Gene Therapy in the Ischemic Stroke Animal Model |
|  | Timing of glucocorticoid administration determines severity of lipid metabolism and behavioral effects in rats |
|  | Facial diplegia as a clinical presentation of a ruptured intracranial dermoid cyst |
|  | Counteractive effects of antenatal glucocorticoid treatment on D1 receptor modulation of spatial working memory |
|  | Lenalidomide-Induced Ischemic Cerebrovascular Disease in Polyneuropathy, Organomegaly, Endocrinopathy, Monoclonal Gammopathy, and Skin Changes Syndrome |
|  | Concurrent radiotherapy and intrathecal methotrexate for treating leptomeningeal metastasis from solid tumors with adverse prognostic factors: A prospective and single-arm study |
|  | Suppression of TDO-mediated tryptophan catabolism in glioblastoma cells by a steroid-responsive FKBP52-dependent pathway |
|  | Increase of glucocorticoids is not required for the acquisition, but hinders the extinction, of lithium-induced conditioned taste aversion |
|  | Corticotropin-releasing hormone exerts direct effects on neuronal progenitor cells: implications for neuroprotection |
|  | Epigenetic modifications of Dexras 1 along the nNOS pathway in an animal model of multiple sclerosis |
|  | RETRACTED: Antenatal corticosteroid administration before elective caesarean section at term to prevent neonatal respiratory morbidity: a randomized controlled trial |
|  | Rasd1, a small G protein with a big role in the hypothalamic response to neuronal activation |
|  | All-trans retinoic acid-induced hypothalamus-pituitary-adrenal hyperactivity involves glucocorticoid receptor dysregulation |
|  | Distribution of Corticotrophin-Releasing Factor Type 1 Receptor-Like Immunoreactivity in the Rat Pituitary |
|  | Isolated optic nerve, chiasm, and tract involvement in Bing-Neel Syndrome |
|  | Adenosine A(2A) receptor regulation of microglia morphological remodeling-gender bias in physiology and in a model of chronic anxiety |
|  | Glucocorticoids Prevent Enterovirus 71 Capsid Protein VP1 Induced Calreticulin Surface Exposure by Alleviating Neuronal ER Stress |
|  | Acute stress causes rapid synaptic insertion of Ca2+ -permeable AMPA receptors to facilitate long-term potentiation in the hippocampus |
|  | Reversible splenial lesion of the corpus callosum associated with bacterial meningitis |
|  | Toxoplasma gondii infection in the peritoneal macrophages of rats treated with glucocorticoids |
|  | 'Not-so-minor' stroke: Lasting psychosocial consequences of anterior cingulate cortical ischemia in the rat |
|  | Resting state functional MRI connectivity predicts hypothalamus-pituitary-axis status in healthy males |
|  | Repeated unpredictable stress and social isolation induce chronic HPA axis dysfunction and persistent abnormal fear memory |
|  | Comprehensive Diagnosis and Surgical Management of Cushing Disease: Two-Dimensional Angiographic and Operative Video |
|  | Acute Stress Suppresses Synaptic Inhibition and Increases Anxiety via Endocannabinoid Release in the Basolateral Amygdala |
|  | Effects of tail docking and castration on stress responses in lambs and the influence of prenatal glucocorticoid treatment |
|  | Increased left ventricular mass in hypercortisolemic depressed patients: a hypothesis based on a case series |
|  | Allopregnanolone serum concentrations and diurnal cortisol secretion in women with premenstrual dysphoric disorder |
|  | Identifying the cellular targets of drug action in the central nervous system following corticosteroid therapy |
|  | Glucocorticoid exerts its non-genomic effect on IPSC by activation of a phospholipase C-dependent pathway in prefrontal cortex of rats |
|  | Corticotroph tumor progression during long-term therapy with osilodrostat in a patient with persistent Cushing's disease |
|  | Measurable Supratentorial White Matter Volume Changes in Patients with Diffuse Intrinsic Pontine Glioma Treated with an Anti-Vascular Endothelial Growth Factor Agent, Steroids, and Radiation |
|  | Mitochondrial Encephalomyopathy Lactic Acidosis and Stroke-Like Episodes (MELAS): A Case Report and Critical Reappraisal of Treatment Options |
|  | Anti-Apoptotic Protein Bcl-xL Expression in the Midbrain Raphe Region Is Sensitive to Stress and Glucocorticoids |
|  | [Contributions of cortisol suppression tests to understanding of psychiatric disorders: a narrative review of literature] |
|  | Sex-dependent programming effects of prenatal glucocorticoid treatment on the developing serotonin system and stress-related behaviors in adulthood |
|  | Involvement of sonic hedgehog and notch signaling in regenerative neurogenesis in adult zebrafish optic tectum after stab injury |
|  | Evaluation of patients with cardiac amyloidosis using echocardiography, ECG and right heart catheterization |
|  | Perinatal Risk and Protective Factors in the Development of Diffuse White Matter Abnormality on Term-Equivalent Age Magnetic Resonance Imaging in Infants Born Very Preterm |
|  | Differential immunoreactivity of glucocorticoid receptor and GABA in GABAergic afferents to parvocellular neurons in the paraventricular nucleus |
|  | Decline of hippocampal stress reactivity and neuronal ensemble coherence in a mouse model of depression |
|  | Glucocorticoids curtail stimuli-induced CREB phosphorylation in TRH neurons through interaction of the glucocorticoid receptor with the catalytic subunit of protein kinase A |
|  | The CB1 cannabinoid receptor mediates glucocorticoid-induced effects on behavioural and neuronal responses during lactation |
|  | Bilateral diffuse scleritis as a first manifestation of immunoglobulin G4-related sclerosing pachymeningitis |
|  | A simplified approach for efficient isolation of functional microglial cells: Application for modeling neuroinflammatory responses in vitro |
|  | An evolutionarily conserved N-terminal Sgk1 variant with enhanced stability and improved function |
|  | Pituitary corticotroph ontogeny and regulation in transgenic zebrafish |
|  | Chronic glucocorticoids exposure enhances neurodegeneration in the frontal cortex and hippocampus via NLRP-1 inflammasome activation in male mice |
|  | Enhanced Intracranial Microdialysis by Reduction of Traumatic Penetration Injury at the Probe Track |
|  | Porcine glucocorticoid receptor (NR3C1) gene: tissue-specificity of transcriptional strength and glucocorticoid responsiveness of alternative promoters |
|  | Suppressing the endocrine and autonomic stress systems does not impact the emotional stress experience after psychosocial stress |
|  | Vanishing glioblastoma after corticosteroid therapy: does this occurrence modify our surgical strategy? |
|  | Inhibition of the kynurenine pathway protects against reactive microglial-associated reductions in the complexity of primary cortical neurons |
|  | Studies on 16,17-Pyrazoline Substituted Heterosteroids as Anti-Alzheimer and Anti-Parkinsonian Agents Using LPS Induced Neuroinflammation Models of Mice and Rats |
|  | Response of the hypothalamic-pituitary-adrenal axis to stimulation tests before and after exercise training in old and young Standardbred mares |
|  | Concurrent progressive multifocal leukoencephalopathy and central nervous system infiltration by multiple myeloma: A case report |
|  | Glucocorticoid treatment of MCMV infected newborn mice attenuates CNS inflammation and limits deficits in cerebellar development |
|  | Effect of reproductive status on hypothalamic-pituitary-adrenal (HPA) activity and reactivity in male California mice (Peromyscus californicus) |
|  | The Effect of Systemic Steroid on Hearing Preservation After Cochlear Implantation via Round Window Approach: A Guinea Pig Model |
|  | Release of an ~55kDa fragment containing the actin-binding domain of β-spectrin by caspase-8 during FND-induced apoptosis depends on the presence of protein 4.1 |
|  | Poststress block of kappa opioid receptors rescues long-term potentiation of inhibitory synapses and prevents reinstatement of cocaine seeking |
|  | Expression of neuropeptide Y and agouti-related protein mRNA stimulated by glucocorticoids is attenuated via NF-κB p65 under ER stress in mouse hypothalamic cultures |
|  | Effects of nutritional stress during different developmental periods on song and the hypothalamic-pituitary-adrenal axis in zebra finches |
|  | Glucocorticoids promote a glioma stem cell-like phenotype and resistance to chemotherapy in human glioblastoma primary cells: Biological and prognostic significance |
|  | Peritumoral edema following Gamma Knife radiosurgery as the primary treatment for intraventricular meningiomas |
|  | Developmental programming of the HPA and HPG axes by early-life stress in male and female song sparrows |
|  | Assessment of tissue-specific cortisol activity with regard to degeneration of the suspensory ligaments in horses with pituitary pars intermedia dysfunction |
|  | Mechanism of aquaporin 4 (AQP 4) up-regulation in rat cerebral edema under hypobaric hypoxia and the preventative effect of puerarin |
|  | Maternal postpartum corticosterone and fluoxetine differentially affect adult male and female offspring on anxiety-like behavior, stress reactivity, and hippocampal neurogenesis |
|  | Effects of intraoperatively applied glucocorticoid hydrogels on residual hearing and foreign body reaction in a guinea pig model of cochlear implantation |
|  | Cortisol response patterns in depressed women and their healthy daughters at risk: Comparison with healthy women and their daughters |
|  | A case of meningococcal meningitis with multiple cerebellar microbleeds detected by susceptibility-weighted imaging |
|  | A cross species study of heterogeneity in fear extinction learning in relation to FKBP5 variation and expression: Implications for the acute treatment of posttraumatic stress disorder |
|  | Transcription factor CREB3L1 mediates cAMP and glucocorticoid regulation of arginine vasopressin gene transcription in the rat hypothalamus |
|  | Hindbrain catecholamine neurons control rapid switching of metabolic substrate use during glucoprivation in male rats |
|  | Sex differences in the ACTH and cortisol response to pharmacological probes are stressor-specific and occur regardless of alcohol dependence history |
|  | Maternal separation enhances conditioned fear and decreases the mRNA levels of the neurotensin receptor 1 gene with hypermethylation of this gene in the rat amygdala |
|  | Prenatal exposure to glucocorticoids affects body weight, serum leptin levels, and hypothalamic neuropeptide-Y expression in pre-pubertal female rat offspring |
|  | Spontaneous convexity subarachnoid haemorrhage: Clinical series of 3 patients with associated cerebral amyloid angiopathy |
|  | Cloning, expression and characterization of a novel human Ras-related protein that is regulated by glucocorticoid hormone |
|  | Functional effects of polymorphisms on glucocorticoid receptor modulation of human anxiogenic substance-P gene promoter activity in primary amygdala neurones |
|  | Treatment of children with acute lymphoblastic leukemia with risk group based intensification and omission of cranial irradiation: A Korean study of 295 patients |
|  | Regulated endocrine-specific protein-18: a short-lived novel glucocorticoid-regulated endocrine protein |
|  | The inflamed axis: the interaction between stress, hormones, and the expression of inflammatory-related genes within key structures comprising the hypothalamic-pituitary-adrenal axis |
|  | Endocrine and hematological responses of beef heifers divergently ranked for residual feed intake following a bovine corticotropin-releasing hormone challenge |
|  | Successful medical management of a domestic longhair cat with subdural intracranial empyema and multifocal pneumonia |
|  | The effects of birth timing and ambient temperature on the hypothalamic-pituitary-adrenal axis in 3-4 month old rhesus monkeys |
|  | Successful treatment of recurrent CNS disease post-bone marrow transplant in children with familial hemophagocytic lymphohistiocytosis |
|  | Glucocorticoids exacerbate cognitive deficits in TDP-25 transgenic mice via a glutathione-mediated mechanism: implications for aging, stress and TDP-43 proteinopathies |
|  | Feeding and adrenal entrainment stimuli are both necessary for normal circadian oscillation of peripheral clocks in mice housed under different photoperiods |
|  | Acute encephalopathy with unilateral cortical-subcortical lesions in two unrelated kindreds treated with glucocorticoids prenatally for congenital adrenal hyperplasia due to 21-hydroxylase deficiency: established facts and novel insight |
|  | Case report of intracranial Rosai-Dorfman disease |
|  | Escitalopram alters gene expression and HPA axis reactivity in rats following chronic overexpression of corticotropin-releasing factor from the central amygdala |
|  | Prenatally exposure to exogenous glucocorticoids and stress may affect the inner ear |
|  | Effects of pasireotide (SOM230) on protein turnover and p70S6 kinase-S6 ribosomal protein signaling pathway in rat skeletal muscle cells |
|  | Total Artificial Heart Implantation as a Bridge to Heart Transplantation in an Active Duty Service Member With Amyloid Cardiomyopathy |
|  | Expression of the glucocorticoid receptor from the 1A promoter correlates with T lymphocyte sensitivity to glucocorticoid-induced cell death |
|  | Tissue inhibitor of metalloproteinases-3 (TIMP-3) is an extracellular matrix-associated protein with a distinctive pattern of expression in mouse cells and tissues |
|  | Chronic lymphocytic inflammation with pontine perivascular enhancement responsive to steroids (CLIPPERS) with intracranial Epstein-Barr virus infection: A Case Report |
|  | [Case report: a case of Epstein-Barr virus associated hemophagocytic syndrome with reversible leukoencephalopathy in the splenium of the corpus callosum] |
|  | A Natural Mutation in Helix 5 of the Ligand Binding Domain of Glucocorticoid Receptor Enhances Receptor-Ligand Interaction |
|  | The promoter of the human monoamine oxidase A gene |
|  | Correlation between circulating corticosterone and protein carbonylation in the liver after short-term hypokinesia |
|  | Hierarchical glucocorticoid-endocannabinoid interplay regulates the activation of the nucleus accumbens by insulin |
|  | Glucocorticoid affects dendritic transport of BDNF-containing vesicles |
|  | Induction of Exaggerated Cytokine Production in Human Peripheral Blood Mononuclear Cells by a Recombinant SARS-CoV-2 Spike Glycoprotein S1 and Its Inhibition by Dexamethasone |
|  | Programming changes of hippocampal miR-134-5p/SOX2 signal mediate the susceptibility to depression in prenatal dexamethasone-exposed female offspring |
|  | The stress-inducible actin-interacting protein DRR1 shapes social behavior |
|  | Mouse corticotropin-releasing factor receptor type 2alpha gene: isolation, distribution, pharmacological characterization and regulation by stress and glucocorticoids |
|  | Glucocorticoids alter adrenomedullin receptor expression and secretion in endothelial-like cells and astrocytes |
|  | Local inner ear application of dexamethasone in cochlear implant models is safe for auditory neurons and increases the neuroprotective effect of chronic electrical stimulation |
|  | Chronic high-fat diet increases acute neuroendocrine stress response independently of prenatal dexamethasone treatment in male rats |
|  | Anna-Monika Award Lecture, DGPPN Kongress, 2013: the role of the hypothalamic-pituitary-adrenal (HPA) axis in the pathogenesis of psychotic major depression |
|  | Single-agent rituximab for primary CNS lymphoma during pregnancy as a bridge to definitive management |
|  | Glucocorticoid receptor mediated the propofol self-administration by dopamine D1 receptor in nucleus accumbens |
|  | FKBP5 and specific microRNAs via glucocorticoid receptor in the basolateral amygdala involved in the susceptibility to depressive disorder in early adolescent stressed rats |
|  | Central pontine myelinolysis during pregnancy: Pathogenesis, diagnosis and management |
|  | Cerebral vasculitis and lateral rectus palsy - two rare central nervous system complications of dengue fever: two case reports and review of the literature |
|  | Altitude headache |
|  | Corticotropin releasing hormone can selectively stimulate glucose uptake in corticotropinoma via glucose transporter 1 |
|  | Acute hemolysis in a patient with a newly diagnosed glioblastoma |
|  | Sequential cyclophosphamide-bortezomib-dexamethasone unmasks the harmful cardiac effect of dexamethasone in primary light-chain cardiac amyloidosis |
|  | Glucocorticoids, genes and brain function |
|  | The Paired Siglecs in Brain Tumours Therapy: The Immunomodulatory Effect of Dexamethasone and Temozolomide in Human Glioma In Vitro Model |
|  | Fluoxetine protects against OVA induced bronchial asthma and depression in rats |
|  | Fotemustine, teniposide and dexamethasone versus high-dose methotrexate plus cytarabine in newly diagnosed primary CNS lymphoma: a randomised phase 2 trial |
|  | Associations among hypothalamus-pituitary-adrenal axis function, novelty seeking, and retention in methadone maintenance therapy for heroin dependency |
|  | Hypothalamic-pituitary-adrenal axis dysfunction and illness progression in bipolar disorder |
|  | A Randomized, Multicenter, Open-Label, Blinded End Point, Phase 2, Feasibility, Efficacy, and Safety Trial of Preoperative Microvascular Protection in Patients Undergoing Major Abdominal Surgery |
|  | Ft1, a novel gene related to ubiquitin-conjugating enzymes, is deleted in the Fused toes mouse mutation |
|  | Basal glucocorticoid receptor activation induces proliferation and inhibits neuronal differentiation of human induced pluripotent stem cell-derived neuronal precursor cells |
|  | Posttraumatic Uncal Herniation in a Conscious Patient |
|  | Multiple brain parenchymal neurocysticercosis with extraocular muscle cysticercosis affecting levator palpebral superioris and superior rectus complex: an unusual association |
|  | Fight INflammation to Improve outcome after aneurysmal Subarachnoid HEmorRhage (FINISHER) trial: Study protocol for a randomized controlled trial |
|  | Differential effects of β-arrestin1 and β-arrestin2 on somatostatin receptors in murine AtT-20 corticotroph tumor cells |
|  | Dexamethasone improves heat stroke-induced multiorgan dysfunction and damage in rats |
|  | Autoimmune hypophysitis presenting with intracranial multi-organ involvement: three case reports and review of the literature |
|  | Endogenous opioids regulate glucocorticoid-dependent stress-coping strategies in mice |
|  | Probiotics reduce risk-taking behavior in the Elevated Plus Maze in the Flinders Sensitive Line rat model of depression |
|  | Fasting regulates EGR1 and protects from glucose- and dexamethasone-dependent sensitization to chemotherapy |
|  | Dexamethasone decreases the autotaxin-lysophosphatidate-inflammatory axis in adipose tissue: implications for the metabolic syndrome and breast cancer |
|  | [New treatments for cluster headache] |
|  | Spatial learning and memory deficits induced by prenatal glucocorticoid exposure depend on hippocampal CRHR1 and CXCL5 signaling in rats |
|  | Brain metastasis in a patient with multiple malignancies |
|  | GR/NF-κB signaling pathway regulates hippocampal inflammatory responses in diabetic rats with chronic unpredictable mild stress |
|  | Dexamethasone Downregulates Endothelin Receptors and Reduces Endothelin-Induced Production of Matrix Metalloproteinases in Cultured Rat Astrocytes |
|  | Alterations in hypothalamic-pituitary-adrenal function immediately after resection of adrenal adenomas in patients with Cushing's syndrome and others with incidentalomas and subclinical hypercortisolism |
|  | Acute hydrocephalus caused by intraspinal neurocysticercosis: case report |
|  | Glucocorticoids and the Brain after Critical Illness |
|  | Homer1 mediates acute stress-induced cognitive deficits in the dorsal hippocampus |
|  | Effects of dexamethasone and cox inhibitors on intracranial pressure and cerebral perfusion in the lipopolysaccharide treated rats with hyperammonemia |
|  | Maternal dexamethasone exposure during pregnancy in rats disrupts gonadotropin-releasing hormone neuronal development in the offspring |
|  | Delayed restoration of maximum speech discrimination scores in patients with idiopathic sudden sensorineural hearing loss |
|  | Childhood trauma dependent anxious depression sensitizes HPA axis function |
|  | Protein kinase Mζ is involved in the modulatory effect of fluoxetine on hippocampal neurogenesis in vitro |
|  | TNF-α regulates natriuretic peptides and aquaporins in human bronchial epithelial cells BEAS-2B |
|  | Molecular mechanism regulating 24-hour rhythm of dopamine D3 receptor expression in mouse ventral striatum |
|  | Safety and efficacy of thalidomide in patients with POEMS syndrome: a multicentre, randomised, double-blind, placebo-controlled trial |
|  | Interfering with the CCL2-glycosaminoglycan axis as a potential approach to modulate neuroinflammation |
|  | Metastatic Intracerebral Plasmacytoma Treated with Radiation and Thalidomide, Dexamethasone with Cyclophosphamide Chemotherapy |
|  | Hypothalamic evanescent lesion by diffuse large B-cell lymphoma |
|  | Glucocorticoid-mediated Period2 induction delays the phase of circadian rhythm |
|  | Case of a healthy infant born following antenatal enterovirus myocarditis and hydrops |
|  | Unbiased Metabolite Profiling of Schizophrenia Fibroblasts under Stressful Perturbations Reveals Dysregulation of Plasmalogens and Phosphatidylcholines |
|  | Lifetime stress accelerates epigenetic aging in an urban, African American cohort: relevance of glucocorticoid signaling |
|  | Pyogenic brain abscesses treated with antibiotics in a patient with hemophagocytic lymphohistiocytosis on HLH-94 protocol |
|  | Small molecule epigenetic screen identifies novel EZH2 and HDAC inhibitors that target glioblastoma brain tumor-initiating cells |
|  | Brain damage and congenital cataract due to autogenously fecal peritonitis in pregnant Wistar rats |
|  | Chromatin organization as an indicator of glucocorticoid induced natural killer cell dysfunction |
|  | Neurologic deterioration in a child undergoing treatment for tuberculosis meningitis |
|  | Postnatal high-fat diet leads to spatial deficit, obesity, and central and peripheral inflammation in prenatal dexamethasone adult offspring rats |
|  | Standardized Citrus unshiu peel extract ameliorates dexamethasone-induced neurotoxicity and depressive-like behaviors in mice |
|  | Dexamethasone exerts profound immunologic interference on treatment efficacy for recurrent glioblastoma |
|  | Hypothalamo-pituitary-adrenal axis, glucose metabolism and TNF-α in narcolepsy |
|  | Expression and regulation of neuromedin B in pituitary corticotrophs of male melanocortin 2 receptor-deficient mice |
|  | C-reactive protein, pre- and postdexamethasone cortisol levels in post-traumatic stress disorder |
|  | Dexamethasone-Mediated Activation of Fibronectin Matrix Assembly Reduces Dispersal of Primary Human Glioblastoma Cells |
|  | Sensorineural deafness following routine transurethral resection of the prostate |
|  | Mice selected for extremes in stress reactivity reveal key endophenotypes of major depression: a translational approach |
|  | Association of ABCB1 polymorphisms with the antiemetic efficacy of granisetron plus dexamethasone in breast cancer patients |
|  | Switching from astrocytic neuroprotection to neurodegeneration by cytokine stimulation |
|  | [Serotonin transporter gene and stress reactivity in unipolar depression. Role of the HPA system as endophenotype of the SLC6A4 gene] |
|  | Gonadotropin-releasing hormone agonist in premenopausal women does not alter hypothalamic-pituitary-adrenal axis response to corticotropin-releasing hormone |
|  | Ginsenoside Rg1 protects against neuronal degeneration induced by chronic dexamethasone treatment by inhibiting NLRP-1 inflammasomes in mice |
|  | Impact of glycemia on survival of glioblastoma patients treated with radiation and temozolomide |
|  | Corticosteroids for managing tuberculous meningitis |
|  | Cell number and neuropil alterations in subregions of the anterior hippocampus in a female monkey model of depression |
|  | Conditional Deletion of Hsd11b2 in the Brain Causes Salt Appetite and Hypertension |
|  | Role of adenosine receptors in resveratrol-induced intraocular pressure lowering in rats with steroid-induced ocular hypertension |
|  | Combination of Palonosetron, Aprepitant, and Dexamethasone Effectively Controls Chemotherapy-induced Nausea and Vomiting in Patients Treated with Concomitant Temozolomide and Radiotherapy: Results of a Prospective Study |
|  | Intrinsic responses to Borna disease virus infection of the central nervous system |
|  | Subclinical Cushing's syndrome: current concepts and trends |
|  | Combined treatment of rituximab, idarubicin, dexamethasone, cytarabine, methotrexate with radiotherapy for primary central nervous system lymphoma |
|  | Changes in cardiac biomarkers with bortezomib treatment in patients with advanced cardiac amyloidosis |
|  | Treatment of secondary central nervous system lymphoma with intrathecal rituximab, high-dose methotrexate, and R-DHAP followed by autologous stem cell transplantation: results of the HOVON 80 phase 2 study |
|  | Intrathecal Isoniazid for Refractory Tuberculous Meningitis with Cerebral Infarction |
|  | Saturated high-fat feeding independent of obesity alters hypothalamus-pituitary-adrenal axis function but not anxiety-like behaviour |
|  | Hemostatic nanoparticles increase survival, mitigate neuropathology and alleviate anxiety in a rodent blast trauma model |
|  | Monocytes from depressed patients display an altered pattern of response to endotoxin challenge |
|  | Central nervous system stem cell transplantation for children with neuronal ceroid lipofuscinosis |
|  | Preclinical Modeling of Surgery and Steroid Therapy for Glioblastoma Reveals Changes in Immunophenotype that are Associated with Tumor Growth and Outcome |
|  | Associations between cognitive performance and cortisol reaction to the DEX/CRH test in patients recovered from depression |
|  | The obesity-associated transcription factor ETV5 modulates circulating glucocorticoids |
|  | Regression of a glioblastoma multiforme: spontaneous versus a potential antineoplastic effect of dexamethasone and levetiracetam |
|  | Caveolin-1 regulates genomic action of the glucocorticoid receptor in neural stem cells |
|  | A novel non genomic glucocorticoid signaling mediated by a membrane palmitoylated glucocorticoid receptor cross talks with GnRH in gonadotrope cells |
|  | Effect of acamprosate on magnetic resonance spectroscopy measures of central glutamate in detoxified alcohol-dependent individuals: a randomized controlled experimental medicine study |
|  | Brain tuberculoma, an unusual cause of stroke in a child with trisomy 21: a case report |
|  | The Cortisol and ACTH Response to Dex/CRH Testing in Women With and Without Perimenopausal Depression |
|  | The impact of peri-operative dexamethasone administration on the normal hypothalamic pituitary adrenal response to major surgical procedures |
|  | Treating Anthrax-Induced Meningitis in Rabbits |
|  | Glucocorticoids attenuate interleukin-6-induced c-Fos and Egr1 expression and impair neuritogenesis in PC12 cells |
|  | Dysregulation of vitamin D metabolism in the brain and myocardium of rats following prolonged exposure to dexamethasone |
|  | Hypothalamic-pituitary-adrenal axis in lethal canine Staphylococcus aureus pneumonia |
|  | Immunoglobulin light chain amyloidosis: 2014 update on diagnosis, prognosis, and treatment |
|  | Salivary cortisol levels and the 2-year course of depressive and anxiety disorders |
|  | Adherence to antiemetic guidelines in patients with malignant glioma: a quality improvement project to translate evidence into practice |
|  | Dexamethasone rapidly increases GABA release in the dorsal motor nucleus of the vagus via retrograde messenger-mediated enhancement of TRPV1 activity |
|  | The effect of dexamethasone/cell-penetrating peptide nanoparticles on gene delivery for inner ear therapy |
|  | Prophylactic and therapeutic functions of drug combinations against noise-induced hearing loss |
|  | Intractable pneumococcal meningoencephalitis associated with a TNF-α antagonist |
|  | Surgically induced SMART syndrome: case report and review of the literature |
|  | Osmotic therapies added to antibiotics for acute bacterial meningitis |
|  | Concurrent asymptomatic inflammatory aneurysm and ischemic stroke due to cysticercal arteritis |
|  | Genetic variation in FKBP5 associated with the extent of stress hormone dysregulation in major depression |
|  | Cognitive impairment and cortisol levels in first-episode schizophrenia patients |
|  | Corticosteroid-endocannabinoid loop supports decrease of fear-conditioned response in rats |
|  | Comparison of neutrophil to lymphocyte ratio and prognostic nutritional index with other clinical and molecular biomarkers for prediction of glioblastoma multiforme outcome |
|  | A corticosteroid-induced gene expressing an "IsK-like" K+ channel activity in Xenopus oocytes |
|  | Relations between cochlear histopathology and hearing loss in experimental cochlear implantation |
|  | Long-Term Programming of CD8 T Cell Immunity by Perinatal Exposure to Glucocorticoids |
|  | Post-dexamethasone serum copeptin corresponds to HPA axis responsiveness in human obesity |
|  | The influence of FKBP5 genotype on expression of FKBP5 and other glucocorticoid-regulated genes, dependent on trauma exposure |
|  | Prevention of trauma-induced cochlear fibrosis using intracochlear application of anti-inflammatory and antiproliferative drugs |
|  | Delayed effects of brain irradiation--part 1: adrenocortical axis dysfunction and hippocampal damage in an adult rat model |
|  | Glucocorticoids regulate the metabolic hormone FGF21 in a feed-forward loop |
|  | Anti-edematous, anti-inflammatory and neuroprotective effect of etanercept in acute stage in experimental head injury |
|  | Cloning of two adenosine receptor subtypes from mouse bone marrow-derived mast cells |
|  | Diplopia and variable ptosis as the sole initial findings in a case of orbital plasmacytoma and multiple myeloma |
|  | Dexamethasone downregulated the expression of CSF 14-3-3β protein in mice with eosinophilic meningitis caused by Angiostrongylus cantonensis infection |
|  | Glucocorticoid inhibition of leptin- and lipopolysaccharide-induced interleukin-6 production in obesity |
|  | Pemetrexed-induced eyelid edema in lung cancer |
|  | Stress and neurodevelopmental processes in the emergence of psychosis |
|  | Methotrexate-cytarabine-dexamethasone combination chemotherapy with or without rituximab in patients with primary central nervous system lymphoma |
|  | Time matters - acute stress response and glucocorticoid sensitivity in early multiple sclerosis |
|  | Prevention of behavioral deficits in rats exposed to folate receptor antibodies: implication in autism |
|  | Dexamethasone suppresses neurosteroid biosynthesis via downregulation of steroidogenic enzyme gene expression in human glioma GI-1 cells |
|  | Recurrent dysphasia due to nivolumab-induced encephalopathy with presence of Hu autoantibody |
|  | Enhanced immune-modulatory effects of thalidomide and dexamethasone co-treatment on T cell subsets |
|  | Characteristics and outcome of patients with primary CNS lymphoma in a "real-life" setting compared to a clinical trial |
|  | Systemic Immunomodulatory Effects of Combinatorial Treatment of Thalidomide and Dexamethasone on T Cells and Other Immune Cells |
|  | Light chain amyloidosis 2012: a new era |
|  | Allele-specific FKBP5 DNA demethylation mediates gene-childhood trauma interactions |
|  | Nephroblastoma overexpressed gene (NOV) codes for a growth factor that induces protein tyrosine phosphorylation |
|  | Preoperative steroids triggering diabetic ketoacidosis in the neurosurgical patient |
|  | Anti-apoptotic and antioxidant effects of low dose gamma irradiation against diabetes-induced brain injury in rats |
|  | A new dexamethasone-induced gene of the leucine zipper family protects T lymphocytes from TCR/CD3-activated cell death |
|  | Long-term survival of diffuse large B cell lymphoma of the trigeminal region extending to the Meckel's cave treated by CHASER therapy: case report |
|  | Bacillus cereus bacteremia and multiple brain abscesses during acute lymphoblastic leukemia induction therapy |
|  | Phase II trial of hypofractionated IMRT with temozolomide for patients with newly diagnosed glioblastoma multiforme |
|  | Bradykinin B2 receptor in the adrenal medulla of male rats and mice: glucocorticoid-dependent increase with immobilization stress |
|  | Diffuse large B cell lymphomas relapsing in the CNS lack oncogenic MYD88 and CD79B mutations |
|  | Efficacy and safety of baricitinib for the treatment of hospitalised adults with COVID-19 (COV-BARRIER): a randomised, double-blind, parallel-group, placebo-controlled phase 3 trial |
|  | Early relapses in patients with primary CNS lymphoma treated with methotrexate-based chemotherapy without consolidating whole brain irradiation |
|  | Herpes simplex virus-1 encephalitis induced by chemoradiotherapy and steroids in an esophageal cancer patient: a case report |
|  | Characterization of the structure and regulation of two novel isoforms of serum- and glucocorticoid-induced protein kinase |
|  | Title: role of matrix metalloproteinase -9 in progression of tuberculous meningitis: a pilot study in patients at different stages of the disease |
|  | Zebrafish is a predictive model for identifying compounds that protect against brain toxicity in severe acute organophosphorus intoxication |
|  | Severe cerebral edema following nivolumab treatment for pediatric glioblastoma: case report |
|  | Urtica dioica extract attenuates depressive like behavior and associative memory dysfunction in dexamethasone induced diabetic mice |
|  | The effect of imipramine, ketamine, and zinc in the mouse model of depression |
|  | Subchronic glucocorticoid receptor inhibition rescues early episodic memory and synaptic plasticity deficits in a mouse model of Alzheimer's disease |
|  | The influence of Hatha yoga as an add-on treatment in major depression on hypothalamic-pituitary-adrenal-axis activity: a randomized trial |
|  | 59-Year-Old Man With Fatigue, Weight Loss, and Hepatomegaly |
|  | Distribution of bovine herpesvirus type 1 in the nervous system of experimentally infected calves |
|  | Tumoral Bing-Neel Syndrome presenting as a cerebellar mass |
|  | A new member of the tumor necrosis factor/nerve growth factor receptor family inhibits T cell receptor-induced apoptosis |
|  | Circulating insulin-like growth factor I modulates mood and is a biomarker of vulnerability to stress: from mouse to man |
|  | Inhibition of SUR1 decreases the vascular permeability of cerebral metastases |
|  | The contribution of postnatal steroid administration to early brain damage in preterm babies with bronchopulmonary dysplasia |
|  | Repeated intranasal oxytocin administration in early life dysregulates the HPA axis and alters social behavior |
|  | Regulatable interleukin-12 gene therapy in patients with recurrent high-grade glioma: Results of a phase 1 trial |
|  | COVID-19-induced toxic epidermal necrolysis |
|  | Diurnal variation of hypothalamic function and chronic subthalamic nucleus stimulation in Parkinson's disease |
|  | Bmal1 interference impairs hormone synthesis and promotes apoptosis in porcine granulosa cells |
|  | Comparing the Efficacy of DeVIC Therapy and High-dose Methotrexate Monotherapy with Whole-brain Radiation Therapy for Newly-diagnosed Primary Central Nervous System Lymphoma: A Single Institution Study |
|  | Cytochrome p450 mRNA expression in the rodent brain: species-, sex-, and region-dependent differences |
|  | Cognitive Dysfunction After Cranial Radiation for a Brain Tumor |
|  | Brain-derived neurotrophic factor signaling rewrites the glucocorticoid transcriptome via glucocorticoid receptor phosphorylation |
|  | Molecular forms of C-type natriuretic peptide in cerebrospinal fluid and plasma reflect differential processing in brain and pituitary tissues |
|  | Sex Hormone Depletion Augments Glucocorticoid Induction of Tau Hyperphosphorylation in Male Rat Brain |
|  | Hydrocortisone treatment for bronchopulmonary dysplasia and brain volumes in preterm infants |
|  | Population variation in neuroendocrine activity is associated with behavioral inhibition and hemispheric brain structure in young rhesus monkeys |
|  | Suppressing Interferon-γ Stimulates Microglial Responses and Repair of Microbleeds in the Diabetic Brain |
|  | Implanting mechanics of PEG/DEX coated flexible neural probe: impacts of fabricating methods |
|  | Electroconvulsive therapy's mechanism of action: neuroendocrine hypotheses |
|  | Pharmacological targeting of secondary brain damage following ischemic or hemorrhagic stroke, traumatic brain injury, and bacterial meningitis - a systematic review and meta-analysis |
|  | Super-resolution structural analysis of dendritic spines using three-dimensional structured illumination microscopy in cleared mouse brain slices |
|  | Using genome-wide CRISPR library screening with library resistant DCK to find new sources of Ara-C drug resistance in AML |
|  | Brain injury and altered brain growth in preterm infants: predictors and prognosis |
|  | Phase I dose escalation trial of vandetanib with fractionated radiosurgery in patients with recurrent malignant gliomas |
|  | Third Trimester Brain Growth in Preterm Infants Compared With In Utero Healthy Fetuses |
|  | Endoscopic transsphenoidal pituitary surgery: a good and safe primary treatment option for Cushing's disease, even in case of macroadenomas or invasive adenomas |
|  | Bovine herpesvirus-5 infection in a rabbit experimental model: immunohistochemical study of the cellular response in the CNS |
|  | Antidepressant effects of TrkB ligands on depression-like behavior and dendritic changes in mice after inflammation |
|  | Isolation and molecular characterisation of Halicephalobus gingivalis in the brain of a horse in Piedmont, Italy |
|  | Phosphodiesterase-1b deletion confers depression-like behavioral resistance separate from stress-related effects in mice |
|  | Ghrelin augments murine T-cell proliferation by activation of the phosphatidylinositol-3-kinase, extracellular signal-regulated kinase and protein kinase C signaling pathways |
|  | Glucocorticoid regulation of vasopressin V1a receptors in rat forebrain |
|  | Toxoplasma gondii 70 kDa heat shock protein: systemic detection is associated with the death of the parasites by the immune response and its increased expression in the brain is associated with parasite replication |
|  | Duration of mechanical ventilation is more critical for brain growth than postnatal hydrocortisone in extremely preterm infants |
|  | The possible anti-apoptotic and antioxidant effects of acetyl l-carnitine as an add-on therapy on a relapsing-remitting model of experimental autoimmune encephalomyelitis in rats |
|  | ADAM9 promotes lung cancer metastases to brain by a plasminogen activator-based pathway |
|  | Multimodal nanoprobes to target cerebrovascular amyloid in Alzheimer's disease brain |
|  | Lapatinib with whole brain radiotherapy in patients with brain metastases from breast and non-small cell lung cancer: a phase II study of the Hellenic Cooperative Oncology Group (HeCOG) |
|  | Two hit induced acute lung injury impairs cognitive function in mice: A potential model to study cross talk between lung and brain |
|  | Low-dose bevacizumab as an effective pre-treatment for peri-tumoral brain edema prior to CyberKnife radiosurgery: A case report |
|  | Dietary administration of cumin-derived cuminaldehyde induce neuroprotective and learning and memory enhancement effects to aging mice |
|  | Preventing inflammation inhibits biopsy-mediated changes in tumor cell behavior |
|  | The Protective Effects and the Involved Mechanisms of Tanshinone IIA on Sepsis-Induced Brain Damage in Mice |
|  | Trigonelline mitigates lipopolysaccharide-induced learning and memory impairment in the rat due to its anti-oxidative and anti-inflammatory effect |
|  | Pharmacokinetics of Budesonide Administered with Surfactant in Premature Lambs: Implications for Neonatal Clinical Trials |
|  | Obstructive sleep apnea syndrome causes a pseudo-Cushing's state in Japanese obese patients with type 2 diabetes mellitus |
|  | Microarray Expression Data Identify DCC as a Candidate Gene for Early Meningioma Progression |
|  | Ocular surface injury induces inflammation in the brain: in vivo and ex vivo evidence of a corneal-trigeminal axis |
|  | Salivary Cortisol and Regional Brain Volumes Among Veterans With and Without Posttraumatic Stress Disorder |
|  | MCL1 gene co-expression module stratifies multiple myeloma and predicts response to proteasome inhibitor-based therapy |
|  | Microglia may compensate for dopaminergic neuron loss in experimental Parkinsonism through selective elimination of glutamatergic synapses from the subthalamic nucleus |
|  | Anti-N-methyl-D-aspartate receptor-mediated encephalitis in infants and toddlers: case report and review of the literature |
|  | Manifestation of Intracranial Lesions at High Altitude: Case Report and Review of the Literature |
|  | High-altitude cerebral oedema mimicking stroke |
|  | Treatment with the NK1 antagonist emend reduces blood brain barrier dysfunction and edema formation in an experimental model of brain tumors |
|  | The properties and utility of the CORE measure of melancholia |
|  | CART (cocaine- and amphetamine-regulated transcript) peptide specific binding sites in PC12 cells have characteristics of CART peptide receptors |
|  | High-dose glucocorticoid aggravates TBI-associated corticosteroid insufficiency by inducing hypothalamic neuronal apoptosis |
|  | HPA Axis Genes, and Their Interaction with Childhood Maltreatment, are Related to Cortisol Levels and Stress-Related Phenotypes |
|  | Immunoendocrine dysbalance during uncontrolled T. cruzi infection is associated with the acquisition of a Th-1-like phenotype by Foxp3(+) T cells |
|  | Microglia in normal appearing white matter of multiple sclerosis are alerted but immunosuppressed |
|  | [Adrenocorticotropic-secreting pheochromocytoma] |
|  | Intranasal delivery of dexamethasone efficiently controls LPS-induced murine neuroinflammation |
|  | [Study on intervention action of total flavonoids from Ilex Pubescens Radix on animal models of cerebral ischemic tolerance with blood stasis] |
|  | Synthesis and Biological Evaluation of Derivatives of Indoline as Highly Potent Antioxidant and Anti-inflammatory Agents |
|  | Interactions between inflammatory mediators and corticosteroids regulate transcription of genes within the Kynurenine Pathway in the mouse hippocampus |
|  | Risk-averse personalities have a systemically potentiated neuroendocrine stress axis: A multilevel experiment in Parus major |
|  | Thyroid hormone regulation of gene expression in primary cerebrocortical cells: role of thyroid hormone receptor subtypes and interactions with retinoic acid and glucocorticoids |
|  | cDNA cloning of a novel CYP3A from rat brain |
|  | Transcriptional signatures of steroid hormones in the striatal neurons and astrocytes |
|  | Symptomatic, non-infectious, non-hemorrhagic edema after subthalamic nucleus deep brain stimulation surgery for Parkinson's disease |
|  | A 43-year-old woman presenting with subacute, bilateral, sequential facial nerve palsies, then developing pseudotumour cerebri |
|  | Hypothalamic-pituitary-adrenal axis regulation and organization in urban and rural song sparrows |
|  | Glucocorticoids modify effects of TGF-β1 on multidrug resistance in the fetal blood-brain barrier |
|  | Rabbit Model of Human Gliomas: Implications for Intra-Arterial Drug Delivery |
|  | Prenatal xenobiotic exposure and intrauterine hypothalamus-pituitary-adrenal axis programming alteration |
|  | carboxypeptidase E-ΔN, a neuroprotein transiently expressed during development protects embryonic neurons against glutamate neurotoxicity |
|  | Anti-inflammatory substances can influence some glial cell types but not others |
|  | Pharmacology of acute mountain sickness: old drugs and newer thinking |
|  | Rapid Nongenomic Glucocorticoid Actions in Male Mouse Hypothalamic Neuroendocrine Cells Are Dependent on the Nuclear Glucocorticoid Receptor |
|  | Magnetic resonance imaging characteristics and treatment aspects of ventricular tuberculosis in adult patients |
|  | Effects of moderate treadmill exercise and fluoxetine on behavioural and cognitive deficits, hypothalamic-pituitary-adrenal axis dysfunction and alternations in hippocampal BDNF and mRNA expression of apoptosis - related proteins in a rat model of post-traumatic stress disorder |
|  | Bevacizumab for acute neurologic deterioration in patients with glioblastoma |
|  | Sex differences in the association between obsessive-compulsive symptom dimensions and diurnal cortisol patterns |
|  | Acute transient cognitive dysfunction and acute brain injury induced by systemic inflammation occur by dissociable IL-1-dependent mechanisms |
|  | P-glycoprotein ABCB1: a major player in drug handling by mammals |
|  | Central nervous system involvement with multiple myeloma: long term survival can be achieved with radiation, intrathecal chemotherapy, and immunomodulatory agents |
|  | Adult glucocorticoid exposure leads to transcriptional and DNA methylation changes in nuclear steroid receptors in the hippocampus and kidney of mouse male offspring |
|  | Do depressed patients without activation of the hypothalamus-pituitary-adrenal (HPA) system have metabolic disturbances? |
|  | Frequent occurrence of therapeutically reversible CMV-associated encephalopathy during radiotherapy of the brain |
|  | PTEN in prefrontal cortex is essential in regulating depression-like behaviors in mice |
|  | Early-life stress changes expression of GnRH and kisspeptin genes and DNA methylation of GnRH3 promoter in the adult zebrafish brain |
|  | Subchronic glucocorticoids, glutathione depletion and a postpartum model elevate monoamine oxidase a activity in the prefrontal cortex of rats |
|  | Gamma-delta T-cell lymphoma of skin, eye and brain presenting with visual loss |
|  | Hypothalamic-pituitary-adrenal axis function and exposure to stress factors and cannabis use in recent-onset psychosis |
|  | In vitro porcine blood-brain barrier model for permeability studies: pCEL-X software pKa(FLUX) method for aqueous boundary layer correction and detailed data analysis |
|  | Dominance rank causally affects personality and glucocorticoid regulation in female rhesus macaques |
|  | Phase shifts in circadian peripheral clocks caused by exercise are dependent on the feeding schedule in PER2::LUC mice |
|  | Protective effects of puerarin on acute lung and cerebrum injury induced by hypobaric hypoxia via the regulation of aquaporin (AQP) via NF-κB signaling pathway |
|  | [Antihistaminik kullanımı ile tetiklenen tekrarlayıcı baş ağrısı ve reversible serebral vazokonstriksiyon sendromu] |
|  | Nrf2 activation: a potential strategy for the prevention of acute mountain sickness |
|  | Stress impairs response to antidepressants via HPA axis and immune system activation |
|  | Acute measles encephalitis in partially vaccinated adults |
|  | Enhancement of stress resilience through histone deacetylase 6-mediated regulation of glucocorticoid receptor chaperone dynamics |
|  | Neonatal glucocorticoid treatment increased depression-like behaviour in adult rats |
|  | Environmental enrichment modulates HPA axis reprogramming in adult male rats exposed to early adolescent stress |
|  | Hypothalamic-pituitary-adrenal axis hyperactivity is associated with decreased brain-derived neurotrophic factor in female suicide attempters |
|  | Molecular cloning of a mineralocorticoid (type I) receptor complementary DNA from rat hippocampus |
|  | Severe invasive Listeria monocytogenes rhombencephalitis mimicking facial neuritis in a healthy middle-aged man: a case report and literature review |
|  | Prior parental experience attenuates hormonal stress responses and alters hippocampal glucocorticoid receptors in biparental rock doves |
|  | ATAT1 is essential for regulation of homeostasis-retaining cellular responses in corticotrophs along hypothalamic-pituitary-adrenal axis |
|  | Disrupting hypothalamic glucocorticoid receptors causes HPA axis hyperactivity and excess adiposity |
|  | The HPA axis in bipolar disorder: Systematic review and meta-analysis |
|  | Combined effect of non-bacteriolytic antibiotic and inhibition of matrix metalloproteinases prevents brain injury and preserves learning, memory and hearing function in experimental paediatric pneumococcal meningitis |
|  | Delayed cerebral ischaemic injury following apparent recovery from Streptococcus pneumoniae meningitis |
|  | Non-pain-related CRF1 activation in the amygdala facilitates synaptic transmission and pain responses |
|  | Dysregulated diurnal cortisol pattern is associated with glucocorticoid resistance in women with major depressive disorder |
|  | High glucocorticoid levels during gestation activate the inflammasome in hippocampal oligodendrocytes of the offspring |
|  | Trauma exposure relates to heightened stress, altered amygdala morphology and deficient extinction learning: Implications for psychopathology |
|  | An integrated approach to understand biological stress system dysregulation across depressive and anxiety disorders |
|  | Dexmedetomidine protects against glucocorticoid induced progenitor cell apoptosis in neonatal mouse cerebellum |
|  | Predictors of remission in depression to individual and combined treatments (PReDICT): study protocol for a randomized controlled trial |
|  | Anti-Inflammatory Modulation of Microglia via CD163-Targeted Glucocorticoids Protects Dopaminergic Neurons in the 6-OHDA Parkinson's Disease Model |
|  | Relationship between iNOS expression and apoptosis in cerebral tissue, and the effect of sini injection in endotoxin shock rats |
|  | HPA axis reactivity to pharmacologic and psychological stressors in euthymic women with histories of postpartum versus major depression |
|  | Carboxypeptidase E protects hippocampal neurons during stress in male mice by up-regulating prosurvival BCL2 protein expression |
|  | Neuroactive Steroid (3α,5α)3-hydroxypregnan-20-one (3α,5α-THP) and Pro-inflammatory Cytokine MCP-1 Levels in Hippocampus CA1 are Correlated with Voluntary Ethanol Consumption in Cynomolgus Monkey |
|  | Temporal dynamics of the HPA axis linked to exploratory behavior in a wild European songbird (Parus major) |
|  | Chemoimmunotherapy with methotrexate, cytarabine, thiotepa, and rituximab (MATRix regimen) in patients with primary CNS lymphoma: results of the first randomisation of the International Extranodal Lymphoma Study Group-32 (IELSG32) phase 2 trial |
|  | The influence of combined oral contraceptives containing drospirenone on hypothalamic-pituitary-adrenocortical axis activity and glucocorticoid receptor expression and function in women with polycystic ovary syndrome |
|  | Effect of the common functional FKBP5 variant (rs1360780) on the hypothalamic-pituitary-adrenal axis and peripheral blood gene expression |
|  | Prenatal SSRI alters the hormonal and behavioral responses to stress in female mice: Possible role for glucocorticoid resistance |
|  | Validated assay for the simultaneous determination of cortisol and budesonide in human plasma using ultra high performance liquid chromatography-tandem mass spectrometry |
|  | Maternal exercise increases but concurrent maternal fluoxetine prevents the increase in hippocampal neurogenesis of adult offspring |
|  | Evidence for adverse effect of perinatal glucocorticoid use on the developing brain |
|  | Gamma Ventral Capsulotomy in Intractable Obsessive-Compulsive Disorder |
|  | High Sensitivity of the Circadian Clock in the Hippocampal Dentate Gyrus to Glucocorticoid- and GSK3-Beta-Dependent Signals |
|  | Hypothalamic-pituitary-adrenal axis activity in the comorbidity between obsessive-compulsive disorder and major depression |
|  | Norethisterone enanthate-induced cerebral venous sinus thrombosis (CVST) |
|  | Mood Disorder in Systemic Lupus Erythematosus Induced by Antiribosomal P Protein Antibodies Associated with Decreased Serum and Brain Tryptophan |
|  | Obstructive sleep apnoea syndrome is associated with relative hypocortisolemia and decreased hypothalamo-pituitary-adrenal axis response to 1 and 250μg ACTH and glucagon stimulation tests |
|  | Generation and characterization of gsuα:EGFP transgenic zebrafish for evaluating endocrine-disrupting effects |
|  | Roles of glucocorticoids in human parturition: a controversial fact? |
|  | Hyper- and hypocortisolism in bipolar disorder - A beneficial influence of lithium on the HPA-axis? |
|  | Sex differences in the outcome of juvenile social isolation on HPA axis function in rats |
|  | Increase in seizure susceptibility in sepsis like condition explained by spiking cytokines and altered adhesion molecules level with impaired blood brain barrier integrity in experimental model of rats treated with lipopolysaccharides |
|  | Diurnal fluctuations in HPA and neuropeptide Y-ergic systems underlie differences in vulnerability to traumatic stress responses at different zeitgeber times |
|  | Hypothalamic-pituitary-adrenal axis measures and cognitive abilities in early psychosis: Are there sex differences? |
|  | The role of childhood trauma, HPA axis reactivity and FKBP5 genotype on cognition in healthy individuals |
|  | Severe, multimodal stress exposure induces PTSD-like characteristics in a mouse model of single prolonged stress |
|  | Prenatal fluoxetine modifies the behavioral and hormonal responses to stress in male mice: role for glucocorticoid insensitivity |
|  | Dexras1, a small GTPase, is required for glutamate-NMDA neurotoxicity |
|  | Metabotropic glutamate receptor-mediated signaling dampens the HPA axis response to restraint stress |
|  | Hypothalamic-pituitary-adrenal axis activity and cognition in major depression: The role of remission status |
|  | HPA axis regulation, survival, and reproduction in free-living sparrows: Functional relationships or developmental correlations? |
|  | The interaction of BDNF Val66Met, PTSD, and child abuse on psychophysiological reactivity and HPA axis function in a sample of Gulf War Veterans |
|  | Hydrocephalus in tuberculous meningitis: Incidence, its predictive factors and impact on the prognosis |
|  | Mechanisms of hormonal regulation of the peripheral circadian clock in the colon |
|  | Effect of dietary carbohydrates and time of year on ACTH and cortisol concentrations in adult and aged horses |
|  | Corticotropin-releasing hormone and the hypothalamic-pituitary-adrenal axis in psychiatric disease |
|  | Nucleotide sequence of the gene coding for ovine corticotropin-releasing factor and regulation of its mRNA levels by glucocorticoids |
|  | Escitalopram and neuroendocrine response in healthy first-degree relatives to depressed patients--a randomized placebo-controlled trial |
|  | Synergistic effects of celecoxib and bupropion in a model of chronic inflammation-related depression in mice |
|  | Quantitative trait Loci influencing abdominal fat deposition and functional variability of the HPA axis in the rat |
|  | Antioxidant Treatment Induces Hyperactivation of the HPA Axis by Upregulating ACTH Receptor in the Adrenal and Downregulating Glucocorticoid Receptors in the Pituitary |
|  | Increasing adult hippocampal neurogenesis in mice after exposure to unpredictable chronic mild stress may counteract some of the effects of stress |
|  | Evaluation of the diagnostic criteria for Cushing's disease in Japan |
|  | Functioning of the hypothalamic-pituitary-adrenal and growth hormone axes in frequently unexplained disorders: results of a population study |
|  | Clinical correlates of hypothalamic-pituitary-adrenal axis measures in individuals at risk for psychosis and with first-episode psychosis |
|  | HPA axis in psychotic major depression and schizophrenia spectrum disorders: Cortisol, clinical symptomatology, and cognition |
|  | Evidence for increased immune mobilization in First Episode Psychosis compared with the prodromal stage in males |
|  | The predictive value of cortisol levels on 2-year course of depression in older persons |
|  | Impact on cortisol and antidepressant efficacy of quetiapine and escitalopram in depression |
|  | Increased plasma levels of circulating cell-free mitochondrial DNA in suicide attempters: associations with HPA-axis hyperactivity |
|  | Effects of aging on hypothalamic-pituitary-adrenal (HPA) axis activity and reactivity in virgin male and female California mice (Peromyscus californicus) |
|  | Comparison of hair follicle histology between horses with pituitary pars intermedia dysfunction and excessive hair growth and normal aged horses |
|  | Utility and Safety of Intrathecal Methotrexate Treatment in Severe Anti-N-methyl-D-aspartate Receptor Encephalitis: A Pilot Study |
|  | Hypothalamic-Pituitary-Adrenocortical Dysfunction in Elderly, Male Marathon Runners: Feedback Sensitivity, Stress Response, and Effects on Verbal Memory |
|  | Loneliness in older adults is associated with diminished cortisol output |
|  | Epigenetic modifications and glucocorticoid sensitivity in Myalgic Encephalomyelitis/Chronic Fatigue Syndrome (ME/CFS) |
|  | Mechanisms underlying the increased plasma ACTH levels in chronic psychosocially stressed male mice |
|  | Hypothalamic-pituitary-adrenal axis activity, personality traits, and BCL1 and N363S polymorphisms of the glucocorticoid receptor gene in metabolically obese normal-weight women |
|  | Increased cortisol awakening response was associated with time to recurrence of major depressive disorder |
|  | Methylation of HPA axis related genes in men with hypersexual disorder |
|  | A novel point mutation of the human glucocorticoid receptor gene causes primary generalized glucocorticoid resistance through impaired interaction with the LXXLL motif of the p160 coactivators: dissociation of the transactivating and transreppressive activities |
|  | A prospective study of the hypothalamic-pituitary-adrenal axis in children with acute lymphoblastic leukemia receiving chemotherapy |
|  | Negative surgical exploration in patients with Cushing's disease: benefit of two-thirds gland resection on remission rate and a review of the literature |
|  | Nitric Oxide Synthase inhibition counteracts the stress-induced DNA methyltransferase 3b expression in the hippocampus of rats |
|  | Ablation of hippocampal neurogenesis in mice impairs the response to stress during the dark cycle |
|  | MT-III, a brain-specific member of the metallothionein gene family |
|  | Covariation among glucocorticoid regulatory elements varies seasonally in house sparrows |
|  | The effects of early trauma and the FKBP5 gene on PTSD and the HPA axis in a clinical sample of Gulf War veterans |
|  | A rare case of ectopic ACTH syndrome originating from malignant renal paraganglioma |
|  | Pathophysiology of Glucocorticoid Signaling |
|  | Chronic itch impairs mood and HPA axis function in mice: modulation by CRFR1 antagonist |
|  | New types of localization methods for adrenocorticotropic hormone-dependent Cushing's syndrome |
|  | miR-449a contributes to glucocorticoid-induced CRF-R1 downregulation in the pituitary during stress |
|  | The medical perspective on burnout |
|  | Reduced hypothalamic-pituitary-adrenal axis activity in chronic multi-site musculoskeletal pain: partly masked by depressive and anxiety disorders |
|  | Predictability of hypoadrenalism occurrence and duration after adrenalectomy for ACTH-independent hypercortisolism |
|  | Interference in ACTH immunoassay negatively impacts the management of subclinical hypercortisolism |
|  | Attention-deficit/hyperactivity disorder symptoms and stress-related biomarkers |
|  | A naturally hypersensitive glucocorticoid receptor elicits a compensatory reduction of hypothalamus-pituitary-adrenal axis activity early in ontogeny |
|  | Hypothalamus-pituitary-adrenal axis involves in anti-viral ability through regulation of immune response in piglets infected by highly pathogenic porcine reproductive and respiratory syndrome virus |
|  | Hypothalamic-pituitary-adrenal axis activity is associated with the prevalence of chronic kidney disease in diabetic patients |
|  | Dysregulated physiological stress systems and accelerated cellular aging |
|  | Comparative Neuroprotective Effects of Dexamethasone and Minocycline during Hepatic Encephalopathy |
|  | Subjective insomnia symptoms and sleep duration are not related to hypothalamic-pituitary-adrenal axis activity in older adults |
|  | Subtherapeutic voriconazole concentrations associated with concomitant dexamethasone: case report and review of the literature |
|  | The evaluation of the effects of steroid treatment on the tumor and peritumoral edema by DWI and MR spectroscopy in brain tumors |
|  | Depression-like behavior is dependent on age in male SAMP8 mice |
|  | Corticosteroid sensitivity in gliomatosis cerebri delays diagnosis |
|  | Treadmill exercise ameliorates ischemia-induced brain edema while suppressing Na⁺/H⁺ exchanger 1 expression |
|  | Use of dexamethasone in patients with high-grade glioma: a clinical practice guideline |
|  | Relationship of cortisol levels and genetic polymorphisms to antidepressant response to placebo and fluoxetine in patients with major depressive disorder: a prospective study |
|  | Collaboration in the presence of cerebral edema: The complications of steroids |
|  | Effects of escitalopram/quetiapine combination therapy versus escitalopram monotherapy on hypothalamic-pituitary-adrenal-axis activity in relation to antidepressant effectiveness |
|  | Methods for Assessing Serpins as Neuroprotective Therapeutics |
|  | Strongyloides stercoralis Hyperinfection Syndrome Presenting as Severe, Recurrent Gastrointestinal Bleeding, Leading to a Diagnosis of Cushing Disease |
|  | Brain Abscesses Caused by Nocardia paucivorans in a Multiple Myeloma Patient Treated with Lenalidomide and Dexamethasone: a Case Report and Review of Literature |
|  | Glucocorticoid receptor activation selectively influence performance of Wistar rats in Y-maze |
|  | Adenosine A(2A) Receptor Blockade Modulates Glucocorticoid-Induced Morphological Alterations in Axons, But Not in Dendrites, of Hippocampal Neurons |
|  | A randomised double blind placebo controlled phase 2 trial of adjunctive aspirin for tuberculous meningitis in HIV-uninfected adults |
|  | Atorvastatin combined with low-dose dexamethasone for vascular endothelial cell dysfunction induced by chronic subdural hematoma |
|  | Context modulates outcome of perinatal glucocorticoid action in the brain |
|  | Traumatic brain injury as vulnerability factor for the development of psychological disorders in a prospective single-event trauma traffic accident sample |
|  | Short-Term and Long-Term Effects of Dexamethasone on Cognitive Dysfunction Induced by Sevoflurane in Adult Rats |
|  | Severe Radiation Necrosis Successfully Treated With Bevacizumab in an Infant with Low-Grade Glioma and Tumor-Associated Intractable Trigeminal Neuralgia |
|  | Tissue-specific changes in molecular clocks during the transition from pregnancy to lactation in mice |
|  | Metabolic changes in serum steroids for diagnosing and subtyping Cushing's syndrome |
|  | Can progesterone be a better alternative to dexamethasone for use in routine brain surgery? |
|  | Prospective, randomized, double-blind, placebo-controlled phase IIa clinical trial on the effects of an estrogen-progestin combination as add-on to inpatient psychotherapy in adult female patients suffering from anorexia nervosa |
|  | Granisetron versus ondansetron for post-operative nausea and vomiting prophylaxis in elective craniotomies for brain tumors: A randomized controlled double-blind study |
|  | Detection of Pneumocystis in the nasal swabs of immune-suppressed rats by use of PCR and microscopy |
|  | Allergic Asthma-Induced Cognitive Impairment is Alleviated by Dexamethasone |
|  | Treatment and protective effects of metalloproteinase inhibitors alone and in combination with N-Acetyl cysteine plus vitamin E in rats exposed to aflatoxin B(1) |
|  | Cerebral sparganosis in mainland Chinese patients |
|  | The effects of antiepileptic drugs on the growth of glioblastoma cell lines |
|  | Dexamethasone therapy prevents delayed neuropsychiatric sequelae after carbon monoxide poisoning: a prospective registry-based study |
|  | Relative hypocortisolism is associated with obesity and the metabolic syndrome in recurrent affective disorders |
|  | Are steroids a beneficial adjunctive therapy in the immunosuppressed patient with herpes simplex virus encephalitis? |
|  | The correlation between perceived social support, cortisol and brain derived neurotrophic factor levels in healthy women |
|  | Self-resolving prepontine cyst |
|  | Current advances in understanding and managing secondary brain metastasis |
|  | Management of bacterial central nervous system infections |
|  | Successful treatment of IgG4-related hypertrophic pachymeningitis with induction rituximab and dexamethasone followed by maintenance rituximab |
|  | Development of Neurological Mouse Model for Toxoplasmosis Using Toxoplasma gondii Isolated from Chicken in Kenya |
|  | Adjuvant hypofractionated partial-brain radiation therapy for pediatric Ewing sarcoma brain metastases: case report |
|  | Corticosteroid use endpoints in neuro-oncology: Response Assessment in Neuro-Oncology Working Group |
|  | Utilization of multidisciplinary surgical techniques to successfully manage 20 brain abscesses in a child |
|  | GLT-1 Upregulation as a Potential Therapeutic Target for Ischemic Brain Injury |
|  | Embryonic Exposure to Dexamethasone Affects Nonneuronal Cells in the Adult Paraventricular Nucleus of the Hypothalamus |
|  | Immunoglobulin light chain amyloidosis: 2013 update on diagnosis, prognosis, and treatment |
|  | Response assessment of NovoTTF-100A versus best physician's choice chemotherapy in recurrent glioblastoma |
|  | Bevacizumab for radiation necrosis following radiotherapy of brain metastatic disease: a systematic review & meta-analysis |
|  | Immunoglobulin light chain amyloidosis: 2016 update on diagnosis, prognosis, and treatment |
|  | Early life environmental and pharmacological stressors result in persistent dysregulations of the serotonergic system |
|  | Proptosis and hemiplegia as an initial manifestation of multiple myeloma |
|  | Efficacy of perineural dexamethasone with ropivacaine in thoracic paravertebral block for postoperative analgesia in elective thoracotomy: a randomized, double-blind, placebo-controlled trial |
|  | Inhibition of DAMP signaling as an effective adjunctive treatment strategy in pneumococcal meningitis |
|  | Association of Isocitrate Dehydrogenase (IDH) Status With Edema to Tumor Ratio and Its Correlation With Immune Infiltration in Glioblastoma |
|  | Brain abscess after transanal hemorrhoidal dearterialization: a case report |
|  | Fatal case of amoebic encephalitis masquerading as herpes |
|  | Physiological challenges for intracortical electrodes |
|  | Depression-related behavioural and neuroendocrine changes in the Spontaneously Diabetic Torii (SDT) fatty rat, an animal model of type 2 diabetes mellitus |
|  | Characterization of macrophages from schizophrenia patients |
|  | Laparoscopic surgery for primary ovarian and retroperitoneal hydatid disease: A case report |
|  | An Overview of Alternating Electric Fields Therapy (NovoTTF Therapy) for the Treatment of Malignant Glioma |
|  | Maternal Dexamethasone Exposure Alters Synaptic Inputs to Gonadotropin-Releasing Hormone Neurons in the Early Postnatal Rat |
|  | Perturbational Profiling of Metabolites in Patient Fibroblasts Implicates α-Aminoadipate as a Potential Biomarker for Bipolar Disorder |
|  | Novel and preclinical treatment strategies in pneumococcal meningitis |
|  | Clinical benefit in recurrent glioblastoma from adjuvant NovoTTF-100A and TCCC after temozolomide and bevacizumab failure: a preliminary observation |
|  | Multimodal Treatment of Skull Base Inflammatory Pseudotumor: Case Report |
|  | Systemic progesterone for modulating electrocautery-induced secondary brain injury |
|  | Rapid remission of symptomatic brain metastases in melanoma by programmed-death-receptor-1 inhibition |
|  | The olfactory mucosa, first actor of olfactory detection, is sensitive to glucocorticoid hormone |
|  | Impaired Spinal Glucocorticoid Receptor Signaling Contributes to the Attenuating Effect of Depression on Mechanical Allodynia and Thermal Hyperalgesia in Rats with Neuropathic Pain |
|  | Hyperosmolar and methotrexate therapy avoiding surgery in the acute presentation of primary central nervous system lymphoma |
|  | Dandy-Walker Syndrome with Giant Cell Lesions and Cherubism |
|  | Cerebral syphilitic gumma presenting with intracranial gumma and pathologic vertebrae fractures |
|  | Rat preprocarboxypeptidase H. Cloning, characterization, and sequence of the cDNA and regulation of the mRNA by corticotropin-releasing factor |
|  | Wilderness Medical Society practice guidelines for the prevention and treatment of acute altitude illness: 2014 update |
|  | Infantile Tubercular Meningitis With Brain Infarct |
|  | Hippocampal nuclear factor kappa B accounts for stress-induced anxiety behaviors via enhancing neuronal nitric oxide synthase (nNOS)-carboxy-terminal PDZ ligand of nNOS-Dexras1 coupling |
|  | Update on community-acquired bacterial meningitis: guidance and challenges |
|  | Outcomes by Cardiac Stage in Patients With Newly Diagnosed AL Amyloidosis: Phase 3 ANDROMEDA Trial |
|  | Launching Hsp70 neuroprotection: two drugs better than one |
|  | Isolated secondary CNS relapse in a case of stage I diffuse large B-cell lymphoma |
|  | Possible role of aprepitant for intractable nausea and vomiting following whole brain radiotherapy-a case report |
|  | Widening the differential for brain masses in human immunodeficiency virus-positive patients: syphilitic cerebral gummata |
|  | Severe paradoxical reaction in tuberculous meningitis |
|  | Steroids for delayed cerebral edema after traumatic brain injury |
|  | Multiple cerebral gliomas mimicking central nervous system inflammatory demyelinating diseases: A rare case with review of literature |
|  | Neuroendocrine Assessment of Dopaminergic Function during Antidepressant Treatment in Major Depressed Patients |
|  | Characterization of GABAergic marker expression in the chronic unpredictable stress model of depression |
|  | Evidence for Immune Activation and Resistance to Glucocorticoids Following Childhood Maltreatment in Adolescents Without Psychopathology |
|  | Diffuse primary leptomeningeal melanocytosis in a patient receiving a novel cancer cell vaccine for prostate cancer |
|  | Pathology of Experimental Encephalitozoon cuniculi Infection in Immunocompetent and Immunosuppressed Mice in Iraq |
|  | Laparoscopic gynaecological surgery in the context of maintaining normal intracranial pressure |
|  | Expression Profiling of the MAP Kinase Phosphatase Family Reveals a Role for DUSP1 in the Glioblastoma Stem Cell Niche |
|  | Anti-inflammatory drugs decrease infection of brain endothelial cells with EHV-1 in vitro |
|  | The in vivo therapeutic efficacy of the oncolytic adenovirus Delta24-RGD is mediated by tumor-specific immunity |
|  | The Value of Anlotinib in the Treatment of Intractable Brain Edema: Two Case Reports |
|  | Parkinsonism and other movement disorders in 23 cases of neurocysticercosis |
|  | Intracerebral laser interstitial thermal therapy followed by tumor resection to minimize cerebral edema |
|  | Neutrophil-lymphocyte ratio dynamics during concurrent chemo-radiotherapy for glioblastoma is an independent predictor for overall survival |
|  | ACR Appropriateness Criteria® pre-irradiation evaluation and management of brain metastases |
|  | Stereotactic Radiosurgery: Treatment of Brain Metastasis Without Interruption of Systemic Therapy |
|  | Characteristics of particulate and non-particulate corticosteroids. Indications for their use in chronic pain treatments |
|  | Early postnatal handling alters glucocorticoid receptor concentrations in selected brain regions |
|  | A case of water intoxication with prolonged hyponatremia caused by excessive water drinking and secondary SIADH |
|  | Molecular patterns of neurodevelopmental preconditioning: a study of the effects of antenatal steroid therapy in a protein-restriction mouse model |
|  | Magnetic Resonance-Guided Laser-Induced Thermal Therapy for the Treatment of Progressive Enhancing Inflammatory Reactions Following Stereotactic Radiosurgery, or PEIRs, for Metastatic Brain Disease |
|  | The Fluoropen: a simple low-cost device to detect intraoperative fluorescein fluorescence in stereotactic needle biopsy of brain tumors |
|  | Intratympanic corticosteroids in Ménière's disease: A mini-review |
|  | Clinically significant CMV (re)activation during or after radiotherapy/chemotherapy of the brain : Correlation with neurological deterioration and improvement upon antiviral treatment |
|  | Prevention of high altitude illness |
|  | The Glucocorticoid Receptor Is a Critical Regulator of HIV Latency in Human Microglial Cells |
|  | The Anti-inflammatory Effect of the Tricyclic Antidepressant Clomipramine and Its High Penetration in the Brain Might Be Useful to Prevent the Psychiatric Consequences of SARS-CoV-2 Infection |
|  | RANBP2 mutation in an Indian child with recurrent acute necrotizing encephalopathy |
|  | Early-onset symptomatic radiation necrosis after stereotactic radiosurgery in the setting of COVID-19 infection |
|  | Regulatory T cell subsets in patients with medulloblastoma at diagnosis and during standard irradiation and chemotherapy (PBTC N-11) |
|  | The CRF1 Antagonist Verucerfont in Anxious Alcohol-Dependent Women: Translation of Neuroendocrine, But not of Anti-Craving Effects |
|  | Differential diagnosis of a vanishing brain space occupying lesion in a child |
|  | Acute bacterial meningitis |
|  | Treatment strategies for central nervous system infections: an update |
|  | Diffuse glioblastoma resembling acute hemorrhagic leukoencephalitis |
|  | Multiple Disruptions of Glial-Neuronal Networks in Epileptogenesis That Follows Prolonged Febrile Seizures |
|  | Incidence of pseudoprogression in low-grade gliomas treated with radiotherapy |
|  | A phase 0 analysis of ixazomib in patients with glioblastoma |
|  | Tumor microtubes convey resistance to surgical lesions and chemotherapy in gliomas |
|  | A case report of pediatric calciphylaxis-a rare and potentially fatal under diagnosed condition |
|  | Post-treatment sequelae and management of primary laryngeal NK/T-cell lymphoma: A case report |
|  | Immunoglobulin light chain amyloidosis: 2018 Update on diagnosis, prognosis, and treatment |
|  | Chapter for antenatal steroids - Treatment drift for a potent therapy with unknown long-term safety seminars in fetal and neonatal medicine |
|  | Bevacizumab Treatment of Radiation-Induced Brain Necrosis: A Systematic Review |
|  | A Case of Unusual Presentation of Contrast-induced Encephalopathy after Cerebral Angiography Using Iodixanol |
|  | Glucocorticoids promote neural progenitor cell proliferation derived from human induced pluripotent stem cells |
|  | [Tumour form of neurocysticercosis in a patient with prostate carcinoma] |
|  | A 44-year-old man with eye, kidney, and brain dysfunction |
|  | Cerebral amyloid angiopathy-related inflammation with epilepsy mimicking a presentation of brain tumor: A case report and review of the literature |
|  | Fulminant bilateral acute retinal necrosis syndrome associated with viral encephalitis: A case report |
|  | Outcome of cancer-related seizures in patients treated with lacosamide |
|  | Pediatric intracerebral histiocytic sarcoma with rhabdoid features: Case report and literature review |
|  | Long-term survival in a patient with progressive multifocal leukoencephalopathy after therapy with rituximab, fludarabine and cyclophosphamide for chronic lymphocytic leukemia |
|  | Reliability of Magnetic Resonance Spectroscopy and Positron Emission Tomography Computed Tomography in Differentiating Metastatic Brain Tumor Recurrence from Radiation Necrosis |
|  | Glucocorticoid regulates parkin expression in mouse frontal cortex: implications in schizophrenia |
|  | Orbital apex cyst: a rare cause of compressive optic neuropathy post-functional endoscopic sinus surgery |
|  | Pulmonary Pneumatocele in a Pneumonia Patient Infected with Extended-Spectrum β-Lactamase Producing Proteus mirabilis |
|  | Structure and expression of the mouse prealbumin gene |
|  | A Case Report of Listeria monocytogenes Abscesses Presenting as Cortically Predominant Ring-Enhancing Lesions |
|  | Neurocysticercosis in a 23-year-old Chinese man |
|  | Downregulated RASD1 and upregulated miR-375 are involved in protective effects of calycosin on cerebral ischemia/reperfusion rats |
|  | Effects of glucocorticoid treatment given in early or late gestation on growth and development in sheep |
|  | Preventing High Altitude Cerebral Edema in Rats with Repurposed Anti-Angiogenesis Pharmacotherapy |
|  | Cognitive outcome of very-low-birthweight infants at 6 years of age |
|  | The macrophage marker translocator protein (TSPO) is down-regulated on pro-inflammatory 'M1' human macrophages |
|  | Pharmacokinetics and toxicity of intrathecal liposomal cytarabine in children and adolescents following age-adapted dosing |
|  | Culture Negative Listeria monocytogenes Meningitis Resulting in Hydrocephalus and Severe Neurological Sequelae in a Previously Healthy Immunocompetent Man with Penicillin Allergy |
|  | 75 years of neurosurgery at the University of Minnesota |
|  | A Case of Congenital Brainstem Oligodendroglioma: Pathology Findings and Review of the Literature |
|  | Septo-optic dysplasia/de Morsier's syndrome |
|  | Early central nervous system relapse of monomorphic epitheliotropic intestinal T-cell lymphoma after cord blood transplantation |
|  | The Safety of Bevacizumab Administered Shortly after Laser Interstitial Thermal Therapy in Glioblastoma: A Case Series |
|  | Sensitivity and specificity of intrathecal fluorescein and white light excitation for detecting intraoperative cerebrospinal fluid leak in endoscopic skull base surgery: a prospective study |
|  | The effect of some immunomodulatory and anti-inflammatory drugs on Li-pilocarpine-induced epileptic disorders in Wistar rats |
|  | Identification and differentiation of PDX1 β-cell progenitors within the human pancreatic epithelium |
|  | Effect of Embelin Against Lipopolysaccharide-induced Sickness Behaviour in Mice |
|  | Cloning and nucleotide sequence of a complementary DNA encoding Xenopus laevis metallothionein: mRNA accumulation in response to heavy metals |
|  | Acute cerebellitis in adults: a case report and review of the literature |
|  | Investigations on 16-Arylideno Steroids as a New Class of Neuroprotective Agents for the Treatment of Alzheimer's and Parkinson's Diseases |
|  | Radiation therapy combined with intracerebral administration of carboplatin for the treatment of brain tumors |
|  | Beneficial Effects of Maprotiline in a Murine Model of Colitis in Normal and Reserpinised Depressed Rats |
|  | Rare Clinical Presentation of Tuberculous Meningitis: A Case Report |
|  | Intractable hiccups due to herpetic esophagitis in an immunocompromised patient |
|  | COVID-19 Related Acute Hemorrhagic Necrotizing Encephalitis: A Report of Two Cases and Literature Review |
|  | Biomarkers of Cardiac Stress and Cytokine Release Syndrome in COVID-19: A Review |
|  | Quantitative analysis of TNF-α, IL-4, and IL-10 expression, nitric oxide response, and apoptosis in Encephalitozoon cuniculi-infected rabbits |
|  | Preoperative statin use is not associated with improvement in survival after glioblastoma surgery |
|  | Intervention action of total flavonoids from root of Ilex pubescens in cerebral ischemic tolerance with blood stasis |
|  | A case series of primary central nervous system posttransplantation lymphoproliferative disorder: imaging and clinical characteristics |
|  | Chronic myelogenous leukemia presenting with central nervous system infiltration, successfully treated with central nervous system-directed chemotherapy followed by allogeneic stem cell transplantation |
|  | Identification of a Dexamethasone Mediated Radioprotection Mechanism Reveals New Therapeutic Vulnerabilities in Glioblastoma |
|  | Evidence-based guideline: treatment of parenchymal neurocysticercosis: report of the Guideline Development Subcommittee of the American Academy of Neurology |
|  | Pig's ear: Streptococcus suis Meningitis and its associated inner ear implications |
|  | Diarrhea-associated pneumococcal meningitis with complicating hydrocephalus in a child in a resource-limited setting |
|  | N-type calcium channel antibody-mediated autoimmune encephalitis: An unlikely cause of a common presentation |
|  | Differential neuroendocrine and immune responses to acute psychosocial stress in women with type 1 bipolar disorder |
|  | Optimization of IL13Rα2-Targeted Chimeric Antigen Receptor T Cells for Improved Anti-tumor Efficacy against Glioblastoma |
|  | Galectin-1 accelerates high-fat diet-induced obesity by activation of peroxisome proliferator-activated receptor gamma (PPARγ) in mice |
|  | Diffuse traumatic brain injury affects chronic corticosterone function in the rat |
|  | Nivolumab-induced organizing pneumonia in a melanoma patient |
|  | The search for ectopic ACTH production in a 9-year-old boy |
|  | Hydroxycitric acid does not promote inflammation or liver toxicity |
|  | Renal intravascular large B-cell lymphoma predominantly localized in peritubular capillaries |
|  | Clinical presentation and outcome of toxoplasma encephalitis in HIV-infected patients from Zaria, Northern Nigeria: a case series of 9 patients |
|  | Cysticidal Efficacy of Combined Treatment With Praziquantel and Albendazole for Parenchymal Brain Cysticercosis |
|  | Resilience to stress and sex-specific remodeling of microglia and neuronal morphology in a rat model of anxiety and anhedonia |
|  | Phase II study of rituximab given in conjunction with standard chemotherapy in primary central nervous system lymphoma (PCNSL): a trial of the ECOG-ACRIN cancer research group (E1F05) |
|  | PET imaging of disease progression and treatment effects in the experimental autoimmune encephalomyelitis rat model |
|  | Five-fraction stereotactic radiosurgery (SRS) for single inoperable high-risk non-small cell lung cancer (NSCLC) brain metastases |
|  | Secondary stabbing headache associated with intracranial tumors, aneurysms, and arteriovenous malformation: An alarming warning sign |
|  | Profile of Antiemetic Activity of Netupitant Alone or in Combination with Palonosetron and Dexamethasone in Ferrets and Suncus murinus (House Musk Shrew) |
|  | Paciente inmunocompetente con criptococosis cerebral: reporte de un caso |
|  | Development of fine motor skills in preterm infants |
|  | Isolation of a novel ras-recision gene that is induced by hydrogen peroxide from a mouse osteoblastic cell line, MC3T3-E1 |
|  | RP105, a novel B cell surface molecule implicated in B cell activation, is a member of the leucine-rich repeat protein family |
|  | Bilateral optic disc edema as a presentation of an obstructing spinal plasmacytoma |
|  | CYP3C1, the first member of a new cytochrome P450 subfamily found in zebrafish (Danio rerio) |
|  | First-in-Human Trial of EphA2-Redirected CAR T-Cells in Patients With Recurrent Glioblastoma: A Preliminary Report of Three Cases at the Starting Dose |
|  | Right orbital edema masquerading a hematologic malignancy |
|  | Early posttraumatic autonomic and endocrine markers to predict posttraumatic stress symptoms after a preventive intervention with oxytocin |
|  | Cloning and production of antisera to human placental 11 beta-hydroxysteroid dehydrogenase type 2 |
|  | Modulation of MMP-2 and -9 secretion by cytokines, inducers and inhibitors in human melanoma A-2058 cells |
|  | Fulminant diffuse cerebral toxoplasmosis as the first manifestation of HIV infection |
|  | Generalized morphoea in the setting of combined immune checkpoint inhibitor therapy for metastatic melanoma: A case report |
|  | Mass cytometry detects H3.3K27M-specific vaccine responses in diffuse midline glioma |
|  | Severe motor aphasia after reinfusion of cryopreserved autologous stem cells after myeloablative conditioning |
|  | Encephalopathy Induced by Preventive Administration of Acyclovir in a Man with Symptomatic Multiple Myeloma and Renal Dysfunction |
|  | Glucocorticoid resistance syndrome caused by a novel NR3C1 point mutation |
|  | A variant of the neuronal amino acid transporter SLC6A15 is associated with ACTH and cortisol responses and cognitive performance in unipolar depression |
|  | The role of blinatumomab in patients with relapsed/refractory acute lymphoblastic leukemia |
|  | Immunomodulatory Factors Galectin-9 and Interferon-Gamma Synergize to Induce Expression of Rate-Limiting Enzymes of the Kynurenine Pathway in the Mouse Hippocampus |
|  | Characterization of membrane receptor binding activity for cortisol in the liver and kidney of the euryhaline teleost, Mozambique tilapia (Oreochromis mossambicus) |
|  | Modulation of MMP-2 and MMP-9 secretion by cytokines, inducers and inhibitors in human glioblastoma T-98G cells |
|  | Natural product HTP screening for antibacterial (E.coli 0157:H7) and anti-inflammatory agents in (LPS from E. coli O111:B4) activated macrophages and microglial cells; focus on sepsis |
|  | Carbon nanotubes for delivery of small molecule drugs |
|  | High activity of the stress promoter contributes to susceptibility to stress in the tree shrew |
|  | A Phase I Study of Autologous Dendritic Cell Vaccine Pulsed with Allogeneic Stem-like Cell Line Lysate in Patients with Newly Diagnosed or Recurrent Glioblastoma |
|  | [Intraoperative fluorescence diagnostics in surgery of intracranial meningiomas: analysis of 101 cases] |
|  | Antigen-specific immune responses and clinical outcome after vaccination with glioma-associated antigen peptides and polyinosinic-polycytidylic acid stabilized by lysine and carboxymethylcellulose in children with newly diagnosed malignant brainstem and nonbrainstem gliomas |
|  | Efficacy and safety of HD-MTX based systemic chemotherapy regimens: retrospective study of induction therapy for primary central nervous system lymphoma in Chinese |
|  | Ectopic adrenocorticotropic hormone syndrome in a case of duodenal neuroendocrine tumor presenting with liver metastasis |
|  | Cytochrome P450 genetic polymorphism in neonatal drug metabolism: role and practical consequences towards a new drug culture in neonatology |
|  | Treatment of Tuberculous Meningitis and Its Complications in Adults |
|  | Short-term exercise combined with Acipimox administration induces an increase in plasma ACTH and its subsequent fall in the recovery phase in bulimic women |
|  | Membrane-bound glucocorticoid receptors on distinct nociceptive neurons as potential targets for pain control through rapid non-genomic effects |
|  | Neuroendocrine carcinoma of the thyroid causing adrenocorticotrophic hormone-dependent Cushing's syndrome |
|  | Neurotrophic factor mediated neuronal differentiation of human cord blood mesenchymal stem cells and their applicability to assess the developmental neurotoxicity |
|  | A Bayesian analysis of the association between Leukotriene A4 Hydrolase genotype and survival in tuberculous meningitis |
|  | Complex regulation of neutrophil-derived MMP-9 secretion in central nervous system tuberculosis |
|  | Ischaemic stroke and the recanalization drug tissue plasminogen activator interfere with antibacterial phagocyte function |
|  | Management of Cushing syndrome in children and adolescents: experience of a single tertiary centre |
|  | Interaction of Norepinephrine and Glucocorticoids Modulate Inhibition of Principle Cells of Layer II Medial Entorhinal Cortex in Male Mice |
|  | Japanese POEMS syndrome with Thalidomide (J-POST) Trial: study protocol for a phase II/III multicentre, randomised, double-blind, placebo-controlled trial |
|  | Cloning of a novel interleukin (IL)-20-like gene in rainbow trout Oncorhynchus mykiss gives an insight into the evolution of the IL-10 family |
|  | Anti-Ma2-associated limbic encephalitis with coexisting chronic inflammatory demyelinating polyneuropathy in a patient with non-Hodgkin lymphoma: A case report |
|  | A Phase II single-arm trial of palonosetron for the prevention of acute and delayed chemotherapy-induced nausea and vomiting in malignant glioma patients receiving multidose irinotecan in combination with bevacizumab |
|  | The Impact of Resistance Exercise on Muscle Mass in Glioblastoma in Survivors (RESIST): Protocol for a Randomized Controlled Trial |
|  | Timosaponin B-III exhibits antidepressive activity in a mouse model of postpartum depression by the regulation of inflammatory cytokines, BNDF signaling and synaptic plasticity |
|  | The hypnotic bromovalerylurea ameliorates 6-hydroxydopamine-induced dopaminergic neuron loss while suppressing expression of interferon regulatory factors by microglia |
|  | CAPON-nNOS coupling can serve as a target for developing new anxiolytics |
|  | Canadian Headache Society systematic review and recommendations on the treatment of migraine pain in emergency settings |
|  | G0/G1 switch gene 2 has a critical role in adipocyte differentiation |
|  | Cerebral tuberculosis in a patient with systemic lupus erythematosus following cyclophosphamide treatment: a case report |
|  | DMBT1 promotes basal and meconium-induced nitric oxide production in human lung epithelial cells in vitro |
|  | An Optimised Human Cell Culture Model for Alveolar Epithelial Transport |
|  | Carbon dioxide: the cause of devastating stroke without hemodynamic compromise during laparoscopic nephrectomy with injury of the inferior vena cava: A case report |
|  | A case of ectopic ACTH syndrome due to DDAVP-sensitive but V1b receptor-negative bronchial typical carcinoid with lymphatic metastasis and plasma ProGRP elevation |
|  | Neural stem cell-mediated enzyme/prodrug therapy for glioma: preclinical studies |
|  | Effect of San'ao decoction with scorpio and bombyx batryticatus on CVA mice model via airway inflammation and regulation of TRPA1/TRPV1/TRPV5 channels |
|  | Ovarian hormones, but not fluoxetine, impart resilience within a chronic unpredictable stress model in middle-aged female rats |
|  | Molecular characterization of a new gene, CEAL1, encoding for a carcinoembryonic antigen-like protein with a highly conserved domain of eukaryotic translation initiation factors |
|  | Physiological Functions of the COPI Complex in Higher Plants |
|  | Experimental Reactivation of Pulmonary Mycobacterium avium Complex Infection in a Modified Cornell-Like Murine Model |
|  | In vivo bioimaging with tissue-specific transcription factor activated luciferase reporters |
|  | Epigenetic and genetic variation at SKA2 predict suicidal behavior and post-traumatic stress disorder |
|  | Colchicine and high-intensity rosuvastatin in the treatment of non-critically ill patients hospitalised with COVID-19: a randomised clinical trial |
|  | Neurocognitive outcomes decades after treatment for childhood acute lymphoblastic leukemia: a report from the St Jude lifetime cohort study |
|  | Mesenchymal stem cells prevent restraint stress-induced lymphocyte depletion via interleukin-4 |
|  | Involuntary swimming exercise in pregnant rats disturbs ERK1/2 signaling in embryonic neurons through increased cortisol in the amniotic fluid |
|  | The incidence of postoperative respiratory complications: A retrospective analysis of cuffed vs uncuffed tracheal tubes in children 0-7 years of age |
|  | Neuroprotective effect of minocycline in a rat model of branch retinal vein occlusion |
|  | Precision therapeutic targets for COVID-19 |
|  | Antioxidant Compound, Oxyresveratrol, Inhibits APP Production through the AMPK/ULK1/mTOR-Mediated Autophagy Pathway in Mouse Cortical Astrocytes |
|  | LLY-2707, a novel nonsteroidal glucocorticoid antagonist that reduces atypical antipsychotic-associated weight gain in rats |
|  | Dexras1 a unique ras-GTPase interacts with NMDA receptor activity and provides a novel dissociation between anxiety, working memory and sensory gating |
|  | Long-term outcomes of patients with active melanoma brain metastases treated with combination nivolumab plus ipilimumab (CheckMate 204): final results of an open-label, multicentre, phase 2 study |
|  | Childhood Maltreatment and Its Interaction with Hypothalamic-Pituitary-Adrenal Axis Activity and the Remission Status of Major Depression: Effects on Functionality and Quality of Life |
|  | High-dose chemotherapy and autologous stem cell transplant compared with conventional chemotherapy for consolidation in newly diagnosed primary CNS lymphoma--a randomized phase III trial (MATRix) |
|  | Desipramine decreases expression of human and murine indoleamine-2,3-dioxygenases |
|  | Mutations in six nephrosis genes delineate a pathogenic pathway amenable to treatment |
|  | 36th International Symposium on Intensive Care and Emergency Medicine : Brussels, Belgium. 15-18 March 2016 |
| Prednisone | Effect of Different Corticosteroid Dosing Regimens on Clinical Outcomes in Boys With Duchenne Muscular Dystrophy: A Randomized Clinical Trial |
|  | Clinical course, therapeutic responses and outcomes in relapsing MOG antibody-associated demyelination |
|  | Safety and efficacy of prednisone versus placebo in short-term prevention of episodic cluster headache: a multicentre, double-blind, randomised controlled trial |
|  | Impact of Baseline Steroids on Efficacy of Programmed Cell Death-1 and Programmed Death-Ligand 1 Blockade in Patients With Non-Small-Cell Lung Cancer |
|  | Radiotherapy or Autologous Stem-Cell Transplantation for Primary CNS Lymphoma in Patients 60 Years of Age and Younger: Results of the Intergroup ANOCEF-GOELAMS Randomized Phase II PRECIS Study |
|  | A brain mass in a patient with Behcet's disease: a case report |
|  | Case 259 |
|  | Muscle disease |
|  | Primary Angiitis of the Central Nervous System |
|  | A tearfully painful darkness |
|  | The influence of foetal prednisone exposure on the cortisol levels in the offspring |
|  | Hand-grenade splinter-induced hypopituitarism |
|  | Hypothalamus-pituitary-adrenal axis function in patients with rheumatoid arthritis treated with nighttime-release prednisone |
|  | Listeria Monocytogenes Brain Abscess in Crohn's Disease Treated with Adalimumab |
|  | Usefulness of brain SPECT imaging in the study of recurrent hypersomnia: Kleine-Levin syndrome |
|  | Circadian rhythms in rheumatology--a glucocorticoid perspective |
|  | Photo Quiz: An 85-Year-Old Man with Brain Abscess |
|  | Prednisone in Uric Acid lowering in Symptomatic Heart Failure Patients With Hyperuricemia (PUSH-PATH) study |
|  | What's in a name? |
|  | Lymphomatoid granulomatosis of the brain: A case report |
|  | Cabazitaxel: a novel drug for hormone-refractory prostate cancer |
|  | Cerebellar ataxia as a primary manifestation of neuropsychiatric systemic lupus erythematosus |
|  | Disseminated nocardiosis with cerebral and subcutaneous lesions on low-dose prednisone |
|  | Isolated IgG4-related hypertrophic pachymeningitis |
|  | Central Nervous System Prophylaxis Strategies in Diffuse Large B Cell Lymphoma |
|  | A review of associated controversies surrounding glucocorticoid use in veterinary emergency and critical care |
|  | The value of glucocorticoid co-therapy in different rheumatic diseases--positive and adverse effects |
|  | Prednisone alleviates demyelination through regulation of the NLRP3 inflammasome in a C57BL/6 mouse model of cuprizone-induced demyelination |
|  | Bevacizumab Monotherapy Reduces Radiation-induced Brain Necrosis in Nasopharyngeal Carcinoma Patients: A Randomized Controlled Trial |
|  | Answer to July 2018 Photo Quiz |
|  | Spontaneous methicillin-resistant Staphylococcus aureus (MRSA) meningitis |
|  | A Case Report of Neurosarcoidosis Presenting as a Lymphoma Mimic |
|  | Survival time following hospital discharge in dogs with palliatively treated primary brain tumors |
|  | Immunoglobulin light chain amyloidosis: 2016 update on diagnosis, prognosis, and treatment |
|  | Vertigo as a predominant manifestation of neurosarcoidosis |
|  | Tocilizumab reverses cerebral vasculopathy in a patient with homozygous SAMHD1 mutation |
|  | Pseudotumoral lesion: pathology and follow-up |
|  | Immunoglobulin light chain amyloidosis: 2013 update on diagnosis, prognosis, and treatment |
|  | Strategies to prevent the neuropsychiatric side-effects of corticosteroids: a case report and review of the literature |
|  | Post-chemotherapy cognitive impairment in patients with B-cell non-Hodgkin lymphoma: a first comprehensive approach to determine cognitive impairments after treatment with rituximab, cyclophosphamide, doxorubicin, vincristine and prednisone or rituximab and bendamustine |
|  | Progressive multifocal leukoencephalopathy in patients with a hematological malignancy: review of therapeutic options |
|  | Microscopic Polyangiitis with Spinal Cord Involvement: A Case Report and Review of the Literature |
|  | Immunoglobulin light chain amyloidosis: 2014 update on diagnosis, prognosis, and treatment |
|  | Neurosarcoidosis: two case reports with multiple cranial nerve involvement and review of the literature |
|  | Mefloquine improved progressive multifocal leukoencephalopathy in a patient with immunoglobulin A nephropathy |
|  | A case of pembrolizumab-induced localized organizing pneumonia |
|  | Chemotherapy-associated Posterior Reversible Encephalopathy Syndrome: A Case Report and Review of the Literature |
|  | Primary Cutaneous Aggressive Epidermotropic CD8+ T-cell Lymphoma with Brain Involvement and Mimicking Dermatomyositis |
|  | Postpneumococcal Moyamoya syndrome case report and review of the postinfective cases |
|  | Cerebellar ataxia and myeloradiculopathy associated with AP3B2 antibody: a case report and literature review |
|  | NEUROSARCOIDOSIS MASQUERADING AS A CENTRAL NERVOUS SYSTEM TUMOR |
|  | Severe Intracranial Involvement in Giant Cell Arteritis: 5 Cases and Literature Review |
|  | Unihemispheric central nervous system vasculitis |
|  | Penetrating Orbital Injury From a Needlefish |
|  | Lymphocytic Hypophysitis Successfully Treated with Stereotactic Radiosurgery: Case Report and Review of the Literature |
|  | Neuroscientific basis of corticosteroid-induced changes in human cognitive and emotional processing: implications for affective illness |
|  | Iatrogenic Hypothalamus Injury After Resection of Subependymoma Within the Left Brain Ventricle |
|  | Association of Biomarkers with Serious Cardiac Adverse Events during Abiraterone Acetate Treatment in Castration Resistant Prostate Cancer |
|  | Neurocysticercosis presenting as focal hydrocephalus |
|  | Case 232: Amyloid β-related Angiitis |
|  | Predominantly periventricular necrotizing encephalitis due to toxoplasmosis: two unusual cases and review of literature |
|  | Altered mental status: what is the diagnosis? |
|  | A Medication Combination for the Treatment of Central Poststroke Pain via the Adjuvant Use of Prednisone With Gabapentin: A Case Report |
|  | Intrathecal methotrexate prophylaxis and central nervous system relapse in patients with diffuse large B-cell lymphoma following rituximab plus cyclophosphamide, doxorubicin, vincristine and prednisone |
|  | A Subgaleal Hematoma That Turned Out to Be a B-Cell Lymphoma |
|  | Mantle cell lymphoma: a great masquerader |
|  | Paroxysmal dysarthria-ataxia in remitting-relapsing Bickerstaff's-like encephalitis |
|  | Progressive Multifocal Leukoencephalopathy Following Treatment with Rituximab in an HIV-Negative Patient with Non-Hodgkin Lymphoma. A Case Report and Literature Review |
|  | Tinnitus as a presenting symptom of neurosarcoidosis with ocular involvement |
|  | Rapidly progressive dementia due to neurosarcoidosis |
|  | Therapeutic benefits of ACTH and levetiracetam in STXBP1 encephalopathy with a de novo mutation: A case report and literature review |
|  | Liver transplantation for a giant mesenchymal hamartoma of the liver in an adult: Case report and review of the literature |
|  | Drug-induced thrombocytopenia secondary to natalizumab treatment |
|  | Impact of Baseline Corticosteroids on Immunotherapy Efficacy in Patients With Advanced Melanoma |
|  | Hypothalamic-pituitary-adrenal (HPA) axis suppression after treatment with glucocorticoid therapy for childhood acute lymphoblastic leukaemia |
|  | Hidden in Plain Sight: An Elusive Cause of Cardiogenic Shock |
|  | Orbital T-cell lymphoma in youngest recorded patient - early diagnosis, management, and successful outcome: a case report and review of the literature |
|  | [Low signal intensity lesions on brain susceptibility-weighted MRI in a patient with intravascular large B-cell lymphoma] |
|  | Hashimoto Encephalopathy in Pediatrics: Report of 3 Cases |
|  | Lymphomatoid granulomatosis involving the central nervous system: A case report and review of the literature |
|  | Priapism as the initial symptom of primary penile lymphoma: A case report |
|  | Susac syndrome: diverse clinical findings and treatment |
|  | Renal intravascular large B-cell lymphoma predominantly localized in peritubular capillaries |
|  | Sjogren's syndrome with trigeminal neuropathy: motor involvement |
|  | Neurosarcoidosis: clinical characteristics, diagnosis, and treatment in eight Chinese patients |
|  | Magnetic resonance imaging findings in giant cell arteritis |
|  | Pseudotumour cerebri in children: Aetiology, clinical features, and progression |
|  | Paraneoplastic cerebellar degeneration associated with cerebellar hypermetabolism: Case report |
|  | Cognitive impairment in Brazilian patients with Behçet's disease occurs independently of neurologic manifestation |
|  | Primary Central Nervous System Vasculitis With Optic Nerve Involvement |
|  | Allergic fungal rhinosinusitis presenting with intracranial spread along large sphenoidal emissary foramen |
|  | Treatment of initial parenchymal central nervous system involvement in systemic aggressive B-cell lymphoma |
|  | Internal carotid artery floating thrombus in relapsing polychondritis |
|  | Primary dural diffuse large B cell lymphoma mimicking parafalcine meningioma |
|  | Lower motor neuron facial palsy after ventriculoperitoneal shunt surgery |
|  | Neuro-Behçet's disease presenting with tumour-like lesions and responding to rituximab |
|  | Alzheimer's Disease and Intranasal Fluticasone Propionate in the FDA MedWatch Adverse Events Database |
|  | Tumoral Bing-Neel Syndrome presenting as a cerebellar mass |
|  | Testicular non-Hodgkin's lymphoma presenting in a young adult |
|  | Clinical and MRI improvement in a case of progressive multifocal leukoencephalopathy |
|  | Lupus vasculopathy: Diagnostic, pathogenetic and therapeutic considerations |
|  | Musical hallucinations: a rare and atypical presentation of anti-IgLON5 disease responsive to immunosuppressive therapy |
|  | Adrenal Insufficiency Caused by Chronic Corticosteroid Use, Identified through Medication Therapy Management |
|  | Early-onset of multiple sclerosis in a 5-year-old girl |
|  | IL-1β inhibition in autoimmune inner ear disease: can you hear me now? |
|  | Hypophysitis Outcome and Factors Predicting Responsiveness to Glucocorticoid Therapy: A Prospective and Double-Arm Study |
|  | Absence of 11-keto reduction of cortisone and 11-ketotestosterone in the model organism zebrafish |
|  | A mimic's imitator: a cavitary pneumonia in a myasthenic patient with history of tuberculosis |
|  | Incomplete Vogt-Koyanagi-Harada in a 14-year-Old African American female with bilateral disc edema |
|  | Cranial Nerve-VI Palsy as the Main Clinical Manifestation of Neurosarcoidosis |
|  | Bell's Palsy after second dose of Pfizer COVID-19 vaccination in a patient with history of recurrent Bell's palsy |
|  | Abnormality in hippocampal signal intensity predicts atrophy in patients with systemic lupus erythematosus |
|  | Jacobian integration method increases the statistical power to measure gray matter atrophy in multiple sclerosis |
|  | Bilateral Striatal Necrosis after Sydenham's Chorea in a 7-Year-Old Boy: A 2-Year Follow-Up |
|  | Pearls & Oy-sters: The critical role of histopathology in diagnosing cancer-associated necrotizing CNS vasculitis |
|  | Third nerve palsy as the initial manifestation of giant cell arteritis |
|  | Long-term immunosuppression for CNS mouse xenotransplantation: Effects on nigrostriatal neurodegeneration and neuroprotective carotid body cell therapy |
|  | Dexamethasone exposure and memory function in adult survivors of childhood acute lymphoblastic leukemia: A report from the SJLIFE cohort |
|  | Posterior reversible encephalopathy syndrome (PRES) complicating newly-diagnosed diffuse large B-cell lymphoma |
|  | Biopsy proven pituitary sarcoidosis presenting as a possible adenoma |
|  | Early Use of Systemic Corticosteroids in Patients with Advanced NSCLC Treated with Nivolumab |
|  | Neospora caninum and Ehrlichia canis co-infection in a dog with meningoencephalitis |
|  | Central diabetes insipidus as the presenting symptom of granulomatosis with polyangiitis |
|  | Angiotensin II receptor blockers, steroids and radiotherapy in glioblastoma-a randomised multicentre trial (ASTER trial). An ANOCEF study |
|  | A 47-Year-Old Man With Progressive Mental Deterioration During Ventilator Management of Asthma in the ICU |
|  | A prospective study of the hypothalamic-pituitary-adrenal axis in children with acute lymphoblastic leukemia receiving chemotherapy |
|  | Transient adrenal insufficiency in diffuse large B cell lymphoma patients after chemotherapy with short-course, high-dose corticosteroids |
|  | Clinical course of choroidal neovascular membrane in West Nile virus chorioretinitis: a case report |
|  | Ipilimumab-induced Adenohypophysitis and Orbital Apex Syndrome: Importance of Early Diagnosis and Management |
|  | Headaches as a presenting symptom of linear morphea en coup de sabre |
|  | Does infectious fever relieve autistic behavior by releasing glutamine from skeletal muscles as provisional fuel? |
|  | [Adult multi-organ Langerhans cell histiocytosis with fatal outcome] |
|  | [Optic neuritis in juvenile idiopathic arthritis patient] |
|  | Myoclonic status epilepticus as a presentation of caspr2 antibody-associated autoimmune encephalitis |
|  | Primary pituitary diffuse large B-cell lymphoma with somatotroph hyperplasia and acromegaly: case report |
|  | The effect of prednisolone on symptom severity in schizophrenia: A placebo-controlled, randomized controlled trial |
|  | Association between physical activity and immunogenicity of an inactivated virus vaccine against SARS-CoV-2 in patients with autoimmune rheumatic diseases |
|  | Once-daily treatment with beclomethasone dipropionate nasal aerosol does not affect hypothalamic-pituitary-adrenal axis function |
|  | You-Gui pills promote nerve regeneration by regulating netrin1, DCC and Rho family GTPases RhoA, Racl, Cdc42 in C57BL/6 mice with experimental autoimmune encephalomyelitis |
|  | Bilateral thalamic and mesencephalic infarctions with hypopituitarism as long-term complications postradiotherapy: A case report |
|  | A case of primary dural lymphoma: diffuse large B-cell type |
|  | Predominant cerebral cytokine release syndrome in CD19-directed chimeric antigen receptor-modified T cell therapy |
|  | Pineal gland hypermetabolic involvement without central nervous system symptoms in a pediatric patient with primary nodular sclerosis subtype classical Hodgkin Lymphoma |
|  | Effects of albendazole treatment on neurocysticercosis: a randomised controlled trial |
|  | 18-month outcomes of heterologous bilateral hand transplantation in a child: a case report |
|  | [Childhood-onset systemic polyarteritis nodosa and systemic lupus erythematosus: an overlap syndrome?] |
|  | Bilateral optic perineuritis as the presenting feature of giant cell arteritis |
|  | Positron Emission Tomography/Computerized Tomography in Newly Diagnosed Patients with Giant Cell Arteritis Who Are Taking Glucocorticoids |
|  | Spontaneous convexity subarachnoid haemorrhage: Clinical series of 3 patients with associated cerebral amyloid angiopathy |
|  | Small lymphocytic lymphoma presenting as a paraneoplastic syndrome with acute central nervous system demyelination |
|  | Fatal cryptococcal meningitis in a juvenile lupus erythematosus patient |
|  | The First Hand Allotransplantation in Taiwan: A Report at 9 Months |
|  | Cluster-like headache and a cystic hypothalamic tumour as first presentation of sarcoidosis |
|  | Eculizumab in the Treatment of Aquaporin-4 Seronegative Neuromyelitis Optica Spectrum Disorder: A Case Report |
|  | Human herpes virus 8-unrelated primary effusion lymphoma-like lymphoma presenting with cardiac tamponade: A case report |
|  | Lymphoid organs of neonatal and adult mice preferentially produce active glucocorticoids from metabolites, not precursors |
|  | Isolated dysphagia unmasking bulbar neurosarcoidosis and pulmonary sarcoidosis |
|  | Abiraterone acetate, exemestane or the combination in postmenopausal patients with estrogen receptor-positive metastatic breast cancer |
|  | Tacrolimus-induced encephalopathy and polyneuropathy in a renal transplant recipient |
|  | Plasma interferon-alpha is associated with double-positivity for autoantibodies but is not a predictor of remission in early rheumatoid arthritis-a spin-off study of the NORD-STAR randomized clinical trial |
|  | Limited Sampling Strategy for Estimating Mycophenolic Acid Exposure on Day 7 Post-Transplant for Two Mycophenolate Mofetil Formulations Derived From 20 Chinese Renal Transplant Recipients |
|  | Impact of immunosuppressive treatment on the cardiovascular system in patients after hand transplantation |
|  | Serial auditory-evoked potentials in the diagnosis and monitoring of a child with Landau-Kleffner syndrome |
|  | Seropositive neuromyelitis optica: a pediatric case report and 6-year follow-up |
|  | Human parvovirus B19-associated early postoperative acquired pure red cell aplasia in simultaneous pancreas-kidney transplantation: A case report |
|  | Deficiency of Adenosine Deaminase 2 in Adult Siblings: Many Years of a Misdiagnosed Disease With Severe Consequences |
|  | Treatment of recurrent posttransplant lymphoproliferative disorder of the central nervous system with high-dose methotrexate |
|  | Resolution of neuropsychological and FDG-PET abnormalities in a patient with neuropsychiatric systemic lupus erythematosus |
|  | Zika virus epidemic in Brazil. I. Fatal disease in adults: Clinical and laboratorial aspects |
|  | Effect of Bushen Yisui Capsule () on oligodendrocyte lineage genes 1 and 2 in mice with experimental autoimmune encephalomyelitis |
|  | [Paradoxical reaction to antituberculosis treatment in an immunocompetent girl with pulmonary tuberculosis] |
|  | Mediastinal mass and pericardial tamponade in a renal transplant recipient: A rare case of nocardia infection |
|  | Central nervous system relapse in patients with diffuse large B cell lymphoma: analysis of the risk factors and proposal of a new prognostic model |
|  | Atypical haemolytic uremic syndrome (aHUS) and membranoproliferative glomerulonephritis (MPGN), different diseases or a spectrum of complement-mediated glomerular diseases? |
|  | Usefulness of 2-[18F]-fluoro-2-deoxy-D-glucose-positron emission tomography/computed tomography for staging and evaluation of treatment response in IgG4-related disease: a retrospective multicenter study |
|  | Unusual case of Hashimoto's encephalopathy and pseudo-obstruction in a patient with undiagnosed hypothyroidism: a case report |
|  | Acute cerebral infarction in watershed distribution in a patient with hypereosinophilic syndrome without cardiac lesion |
|  | Anti-HMGCR myopathy overlaps with dermatomyositis-like rash: a distinct subtype of idiopathic inflammatory myopathy |
|  | Isolated central nervous system relapse in a patient with systemic diffuse large B-cell lymphoma: Diagnostic and treatment challenges |
|  | A case of stiff dog syndrome associated with anti-glutamic acid decarboxylase antibodies |
|  | Posterior reversible encephalopathy syndrome in a patient with Richter's syndrome on combination DA-R-EPOCH chemotherapy regimen: a case report |
|  | Lack of effect on adult and adolescent hypothalamic-pituitary-adrenal axis function with use of fluticasone furoate nasal spray |
|  | Pattern and Outcome of Acute Disseminated Encephalomyelitis (ADEM) in Children: Experience in a Tertiary Center, Upper Egypt |
|  | Zuo-Gui and You-Gui pills, two traditional Chinese herbal formulas, downregulated the expression of NogoA, NgR, and RhoA in rats with experimental autoimmune encephalomyelitis |
|  | Posterior reversible encephalopathy syndrome after renal transplant: a simple solution for a complicated patient |
|  | Long-term complete remission in a patient with intravascular large B-cell lymphoma with central nervous system involvement |
|  | Clearance of cerebrospinal fluid JCV DNA with mirtazapine in a patient with progressive multifocal leukoencephalopathy and sarcoidosis |
|  | [C-ANCA positive necrotising scleritis and multiple sclerosis compatible with ocular Wegener: treatment with rituximab] |
|  | Unexpected diagnosis of metastatic breast carcinoma in an endomyocardial biopsy done for cardiac allograft rejection evaluation |
|  | Central nervous system relapse of diffuse large B-cell lymphoma in the rituximab era: results of the UK NCRI R-CHOP-14 versus 21 trial |
|  | A 3-Year Observational Study of Patients with Progressive Systemic Sclerosis Treated with an Intensified B Lymphocyte Depletion Protocol: Clinical and Immunological Response |
|  | Biomarker Associations with Efficacy of Abiraterone Acetate and Exemestane in Postmenopausal Patients with Estrogen Receptor-Positive Metastatic Breast Cancer |
|  | Jisuikang, a Chinese herbal formula, increases neurotrophic factor expression and promotes the recovery of neurological function after spinal cord injury |
|  | Progressive multifocal leukoencephalopathy during treatment with rituximab and CHOP chemotherapy in a patient with a diffuse large B-cell lymphoma |
|  | An mTOR-inhibitor-based protocol and calcineurin inhibitor (CNI)-free treatment in kidney transplant recipients from donors after cardiac death: good renal function, but high incidence of conversion to CNI |
|  | Ivermectin treatment of Loa loa hyper-microfilaraemic baboons (Papio anubis): Assessment of microfilarial load reduction, haematological and biochemical parameters and histopathological changes following treatment |
|  | Seizure syndrome as a first manifestation of solitary tumor-like mass lesion of PACNS: Two case reports |
|  | Rapid Multifocal Neurologic Decline in an Immunocompromised Patient |
|  | Chorea as the presenting feature of acute rheumatic fever in childhood; case reports from a low-prevalence European setting |
|  | A Phase I Study of the AKT Inhibitor MK-2206 in Combination with Hormonal Therapy in Postmenopausal Women with Estrogen Receptor-Positive Metastatic Breast Cancer |
|  | Left thigh phlegmon caused by Nocardia farcinica identified by 16S rRNA sequencing in a patient with leprosy: a case report |
|  | The Diving Bell and the Butterfly Revisited: A Fatal Case of Locked-in Syndrome in a Man With Epstein-Barr Virus-Positive Diffuse Large B-Cell Lymphoma, Not Otherwise Specified |
|  | Primary Psychiatric Disorder Masking the Diagnosis of Neuropsychiatric Lupus in a Patient with Altered Mental Status: A Case Report |
|  | Hand-Schüller-Christian disease associated with symptomatic cavum septi pellucidi cyst in an adult with a 10-year follow-up |
|  | Fingolimod in active multiple sclerosis: an impressive decrease in Gd-enhancing lesions |
|  | Advantage of 11C-methionine positron emission tomography for assessing IgG4-related central nervous system lesions |
|  | Multifocal primary bone lymphoma: durable complete remission after R-CHOP chemotherapy |
| Diacerein | Peroxynitrite activates NLRP3 inflammasome and contributes to hemorrhagic transformation and poor outcome in ischemic stroke with hyperglycemia |
| Dexibuprofen | Mechanism of brain targeting by dexibuprofen prodrugs modified with ethanolamine-related structures |
|  | New potential strategies for Alzheimer's disease prevention: pegylated biodegradable dexibuprofen nanospheres administration to APPswe/PS1dE9 |
|  | Dexibuprofen prevents neurodegeneration and cognitive decline in APPswe/PS1dE9 through multiple signaling pathways |
| Tenidap | Tenidap, an agonist of the inwardly rectifying K+ channel Kir2.3, delays the onset of cortical epileptiform activity in a model of chronic temporal lobe epilepsy |
| Bromfenac | Topical Treatment With Bromfenac Reduces Retinal Gliosis and Inflammation After Optic Nerve Crush |
|  | The Effects of Two Non-Steroidal Anti-Inflammatory Drugs, Bromfenac 0.1% and Ketorolac 0.45%, on Cataract Surgery |
| Valdecoxib | Prostanoid receptor EP2 as a therapeutic target |
|  | Intratumoral COX-2 inhibition enhances GM-CSF immunotherapy against established mouse GL261 brain tumors |
|  | A standardized and reproducible protocol for serum-free monolayer culturing of primary paediatric brain tumours to be utilized for therapeutic assays |
|  | Radiosynthesis and in vivo evaluation of [(11)C]MOV as a PET imaging agent for COX-2 |
|  | COXIBs and 2,5-dimethylcelecoxib counteract the hyperactivated Wnt/β-catenin pathway and COX-2/PGE2/EP4 signaling in glioblastoma cells |
|  | Nonsteroidal anti-inflammatory drug choice and adverse outcomes in clopidogrel users: A retrospective cohort study |
|  | The use of a gene expression signature and connectivity map to repurpose drugs for bipolar disorder |
|  | Modulation of LOX and COX pathways via inhibition of amyloidogenesis contributes to mitoprotection against β-amyloid oligomer-induced toxicity in an animal model of Alzheimer's disease in rats |
| Parecoxib | Parecoxib inhibits glioblastoma cell proliferation, migration and invasion by upregulating miRNA-29c |
|  | Pretreatment of parecoxib attenuates hepatic ischemia/reperfusion injury in rats |
|  | Influence of Cyclooxygenase-2 Inhibitors on Kynurenic Acid Production in Rat Brain in Vitro |
|  | Parecoxib suppresses CHOP and Foxo1 nuclear translocation, but increases GRP78 levels in a rat model of focal ischemia |
|  | Protective effects of parecoxib on rat primary astrocytes from oxidative stress induced by hydrogen peroxide |
|  | Chronic administration of parecoxib exerts anxiolytic-like and memory enhancing effects and modulates synaptophysin expression in mice |
|  | The efficacy of parecoxib on systemic inflammatory response associated with cardiopulmonary bypass during cardiac surgery |
|  | Intratumoral COX-2 inhibition enhances GM-CSF immunotherapy against established mouse GL261 brain tumors |
|  | Effect of parecoxib sodium pretreatment combined with dexmedetomidine on early postoperative cognitive dysfunction in elderly patients after shoulder arthroscopy: A randomized double blinded controlled trial |
|  | Effectiveness of a Mindfulness Meditation App Based on an Electroencephalography-Based Brain-Computer Interface in Radiofrequency Catheter Ablation for Patients With Atrial Fibrillation: Pilot Randomized Controlled Trial |
|  | Importance of kynurenine 3-monooxygenase for spontaneous firing and pharmacological responses of midbrain dopamine neurons: Relevance for schizophrenia |
| Etoricoxib | No effect of the cyclooxygenase-2 inhibitor etoricoxib on pre-emptive and post-operative analgesia in visceral surgery: results of a randomized controlled trial |
|  | COXIBs and 2,5-dimethylcelecoxib counteract the hyperactivated Wnt/β-catenin pathway and COX-2/PGE2/EP4 signaling in glioblastoma cells |
|  | Cyclooxygenase-2 inhibitors differentially attenuate pentylenetetrazol-induced seizures and increase of pro- and anti-inflammatory cytokine levels in the cerebral cortex and hippocampus of mice |
|  | Cox-2 Plays a Vital Role in the Impaired Anxiety Like Behavior in Colchicine Induced Rat Model of Alzheimer Disease |
|  | Role of cox-2 mediated neuroinflammation on the neurodegeneration and cognitive impairments in colchicine induced rat model of Alzheimer's Disease |
| Lumiracoxib | Detection of Cyclooxygenase-2-Derived Oxygenation Products of the Endogenous Cannabinoid 2-Arachidonoylglycerol in Mouse Brain |
| Meloxicam | Meloxicam Improves Cognitive Impairment of Diabetic Rats through COX2-PGE2-EPs-cAMP/pPKA Pathway |
|  | Feline hyperaesthesia syndrome with self-trauma to the tail: retrospective study of seven cases and proposal for an integrated multidisciplinary diagnostic approach |
|  | Unfolded protein response to global ischemia following 48 h of reperfusion in the rat brain: the effect of age and meloxicam |
|  | Investigation of Absorption Routes of Meloxicam and Its Salt Form from Intranasal Delivery Systems |
|  | Cyclooxygenase-2 inhibitor prevents radiation-enhanced infiltration of F98 glioma cells in brain of Fischer rat |
|  | Co-nanoencapsulated meloxicam and curcumin improves cognitive impairment induced by amyloid-beta through modulation of cyclooxygenase-2 in mice |
|  | Age and meloxicam modify the response of the glutamate vesicular transporters (VGLUTs) after transient global cerebral ischemia in the rat brain |
|  | Effects of dexamethasone and meloxicam on Borrelia burgdorferi-induced inflammation in glial and neuronal cells of the central nervous system |
|  | Meloxicam blocks neuroinflammation, but not depressive-like behaviors, in HIV-1 transgenic female rats |
|  | Effects of selective and non-selective cyclooxygenase inhibition against neurological deficit and brain oedema following closed head injury in mice |
|  | Bicuculline Reverts the Neuroprotective Effects of Meloxicam in an Oxygen and Glucose Deprivation (OGD) Model of Organotypic Hippocampal Slice Cultures |
|  | Meloxicam-loaded nanocapsules as an alternative to improve memory decline in an Alzheimer's disease model in mice: involvement of Na(+), K(+)-ATPase |
|  | A comparison study of lipid and polymeric nanoparticles in the nasal delivery of meloxicam: Formulation, characterization, and in vitro evaluation |
|  | Transport rankings of non-steroidal antiinflammatory drugs across blood-brain barrier in vitro models |
|  | Hippocampal neuronal metal ion imbalance related oxidative stress in a rat model of chronic aluminum exposure and neuroprotection of meloxicam |
|  | Inflammatory mechanisms contribute to microembolism-induced anxiety-like and depressive-like behaviors |
|  | Cyclooxygenase-2 Signalling Pathway in the Cortex is Involved in the Pathophysiological Mechanisms in the Rat Model of Depression |
|  | Influence of NSAIDs and methotrexate on CD73 expression and glioma cell growth |
|  | The roles of prostaglandin E2 and D2 in lipopolysaccharide-mediated changes in sleep |
|  | Natural antioxidant flavonoids in formalin-induced mice paw inflammation; inhibition of mitochondrial sorbitol dehydrogenase activity |
|  | Real-time application of the Rat Grimace Scale as a welfare refinement in laboratory rats |
|  | Inflammation and vascular remodeling in the ventral hippocampus contributes to vulnerability to stress |
|  | Trial-unique, delayed nonmatching-to-location (TUNL) touchscreen testing for mice: sensitivity to dorsal hippocampal dysfunction |
|  | Diagnostic imaging of peripheral vestibular disease in a Chinese goose (Anser cygnoides) |
|  | Amyloid-β peptide absence in short term effects on kinase activity of energy metabolism in mice hippocampus and cerebral cortex |
|  | cAMP/PKA-CREB-BDNF signaling pathway in hippocampus mediates cyclooxygenase 2-induced learning/memory deficits of rats subjected to chronic unpredictable mild stress |
|  | Psychometric assessment of the Rat Grimace Scale and development of an analgesic intervention score |
| Tenoxicam | Transport rankings of non-steroidal antiinflammatory drugs across blood-brain barrier in vitro models |
| Lornoxicam | The effects of lornoxicam on brain edema and blood brain barrier following diffuse traumatic brain injury in rats |
|  | The effects of lornoxicam on neuroprotection following diffuse traumatic brain injury in rats |
|  | Antinociceptive tolerance to NSAIDs microinjected into dorsal hippocampus |
|  | Antinociceptive tolerance to NSAIDs in the anterior cingulate cortex is mediated via endogenous opioid mechanism |
| Loxoprofen | Lamotrigine for intractable migraine-like headaches in Sturge-Weber syndrome |
|  | Cloning and characterization of four rabbit aldo-keto reductases featuring broad substrate specificity for xenobiotic and endogenous carbonyl compounds: relationship with multiple forms of drug ketone reductases |
| Amphotericin B | Pharmacokinetics of antifungal drugs: practical implications for optimized treatment of patients |
|  | Neuroinfections caused by fungi |
|  | Study of amphotericin B magnetic liposomes for brain targeting |
|  | Carbon nanotubes for delivery of small molecule drugs |
|  | In vitro and in vivo evidence for amphotericin B as a P-glycoprotein substrate on the blood-brain barrier |
|  | Amphotericin B induces glial cell line-derived neurotrophic factor in the rat brain |
|  | Amphotericin B transfer to CSF following intravenous administration of liposomal amphotericin B |
|  | Intracavitary amphotericin B in the treatment of intracranial aspergillosis |
|  | Cryptococcal meningitis |
|  | Cryptococcose disséminée chez une greffée rénale de longue date |
|  | Sustained intrathecal delivery of amphotericin B using an injectable and biodegradable thermogel |
|  | Experimental Models of Short Courses of Liposomal Amphotericin B for Induction Therapy for Cryptococcal Meningitis |
|  | Prion diseases: immunotargets and therapy |
|  | Novel oral amphotericin B formulation (iCo-010) remains highly effective against murine systemic candidiasis following exposure to tropical temperature |
|  | Pharmacodynamics of liposomal amphotericin B and flucytosine for cryptococcal meningoencephalitis: safe and effective regimens for immunocompromised patients |
|  | Efficacy of liposomal amphotericin B and posaconazole in intratracheal models of murine mucormycosis |
|  | Polysorbate Surfactants as Drug Carriers: Tween 20-Amphotericin B Conjugates as Anti-Fungal and Anti-Leishmanial Agents |
|  | Systemic cryptococcosis in an immune-competent child |
|  | Fungal Infections of the Central Nervous System in Small Animals: Clinical Features, Diagnosis, and Management |
|  | A randomized open label trial of tamoxifen combined with amphotericin B and fluconazole for cryptococcal meningitis |
|  | High-dose fluconazole in combination with amphotericin B is more efficient than monotherapy in murine model of cryptococcosis |
|  | The novel fungal CYP51 inhibitor VT-1598 is efficacious alone and in combination with liposomal amphotericin B in a murine model of cryptococcal meningitis |
|  | Fusarium brain abscess: case report and literature review |
|  | Cerebral protothecosis mimicking high-grade glioma |
|  | Scedosporium aurantiacum brain abscess after near-drowning in a survivor of a tsunami in Japan |
|  | Paracoccidioidomycosis simulating brain tumor |
|  | Use of external ventriculostomy and intrathecal anti-fungal treatment in cerebral mucormycotic abscess |
|  | Rhinocerebral Mucormycosis and COVID-19 Pneumonia |
|  | Systemic paracoccidioidomycosis involving breast |
|  | Histoplasmosis brain abscesses in an immunocompetent adult. A case report and literature review |
|  | Activation of cultured astrocytes by amphotericin B: stimulation of NO and cytokines production and changes in neurotrophic factors production |
|  | In Vitro Efficacy of Ebselen and BAY 11-7082 Against Naegleria fowleri |
|  | MRI monitoring of monocytes to detect immune stimulating treatment response in brain tumor |
|  | Ventriculitis due to infection with Rhizopus arrhizus |
|  | Efficacy of an abbreviated induction regimen of amphotericin B deoxycholate for cryptococcal meningoencephalitis: 3 days of therapy is equivalent to 14 days |
|  | Brain-Eating Amoebae: Silver Nanoparticle Conjugation Enhanced Efficacy of Anti-Amoebic Drugs against Naegleria fowleri |
|  | The battle for the brain: Brain tumor-initiating cells vs. microglia/macrophages |
|  | Identification of cysteine protease inhibitors as new drug leads against Naegleria fowleri |
|  | Efficacy of an oral and tropically stable lipid-based formulation of Amphotericin B (iCo-010) in an experimental mouse model of systemic candidiasis |
|  | Healthy Brain-pituitary Slices for Electrophysiological Investigations of Pituitary Cells in Teleost Fish |
|  | In Vitro and In Vivo Evaluation of the Antifungal Activity of APX001A/APX001 against Candida auris |
|  | Multimodal Treatment of Skull Base Inflammatory Pseudotumor: Case Report |
|  | Phellinus species: An emerging cause of refractory fungal infections in patients with X-linked chronic granulomatous disease |
|  | Candida cerebral abscesses: a case report and review of the literature |
|  | Chronic orbital and calvarial fungal infection with Apophysomyces variabilis in an immunocompetent patient |
|  | Antibiotics armed neutrophils as a potential therapy for brain fungal infection caused by chemotherapy-induced neutropenia |
|  | Meningitis Caused by Candida Dubliniensis in a Patient with Cirrhosis: A Case Report and Review of the Literature |
|  | Chronic rhinocerebral mucormycosis: a rare case report and review of the literature |
|  | Phaeohyphomycosis caused by Cladophialophora bantiana |
|  | Pharmacokinetics/pharmacodynamic correlations of fluconazole in murine model of cryptococcosis |
|  | Naegleria fowleri after 50 years: is it a neglected pathogen? |
|  | Isolated cerebral mucormycosis caused by Rhizomucor pusillus |
|  | Trichosporon asahii infection presenting as chronic meningo-ventriculitis and intra ventricular fungal ball: a case report and literature review |
|  | Fatal cryptococcal meningitis in an AIDS patient complicated with immune reconstitution syndrome refractory to prolonged amphotericin B treatment |
|  | Efficacy of Prosopilosidine from Prosopis glandulosa var. glandulosa against Cryptococcus neoformans Infection in a Murine Model |
|  | Psychosis Due to Disseminated Cryptococcal Infection and Delirium in an Immunocompetent Patient: A Case Report and Review of the Literature |
|  | Fatal case of amoebic encephalitis masquerading as herpes |
|  | Disseminated Hormographiella aspergillata infection with involvement of the lung, brain, and small intestine following allogeneic hematopoietic stem cell transplantation: case report and literature review |
|  | Fungal brain abscess caused by "Black Mold" (Cladophialophora bantiana) - A case report of successful treatment with an emphasis on how fungal brain abscess may be different from bacterial brain abscess |
|  | Evaluation of Mucorales DNA load in cerebrospinal fluid in a patient with possible cerebral mucormycosis treated with intravenous liposomal amphotericin B |
|  | Sinusitis and frontal brain abscess in a diabetic patient caused by the basidiomycete Schizophyllum commune: case report and review of the literature |
|  | In vitro and in vivo antifungal activities of T-2307, a novel arylamidine, against Cryptococcus gattii: an emerging fungal pathogen |
|  | Combined Orofacial Aspergillosis and Mucormycosis: Fatal Complication of a Recurrent Paediatric Glioma-Case Report and Review of Literature |
|  | Therapeutic activation of macrophages and microglia to suppress brain tumor-initiating cells |
|  | Immune Reconstitution Inflammatory Syndrome in Patients with AIDS and Disseminated Coccidioidomycosis: A Case Series and Review of the Literature |
|  | The Anti-helminthic Compound Mebendazole Has Multiple Antifungal Effects against Cryptococcus neoformans |
|  | An Unusual Presentation of Nocardiosis in an Allogeneic Transplant Recipient |
|  | Acute meningitis caused by Cladosporium sphaerospermum |
|  | Derivation of Leptomeninges Explant Cultures from Postmortem Human Brain Donors |
|  | The Repurposing of Anti-Psychotic Drugs, Quetiapine and Olanzapine, as Anti-Cryptococcus Drugs |
|  | Perplexity of a fungus in the sinus |
|  | Long term outcome of medical and surgical co-management of craniospinal aspergillosis in an immunocompromised patient |
|  | Comparative Pathogenicity of Lomentospora prolificans (Scedosporium prolificans) Isolates from Mexican Patients |
|  | Primary cutaneous aspergillosis caused by Aspergillus tamarii in a premature infant with extremely low birthweight: A case report with short review |
|  | In vivo whole-cell recording with high success rate in anaesthetized and awake mammalian brains |
|  | Successful Treatment of Rhino-Orbital-Cerebral Mucormycosis in a Child With Leukemia |
|  | Fungal meningitis in an immunocompetent patient |
|  | Cryptococcosis in an immunocompetent pregnant woman |
|  | Successful Control of a Co-Infection Caused by Candida albicans and Pseudomonas aeruginosa in Keratitis |
|  | Brain abscess caused by Cladophialophora bantiana in China |
|  | Melanization and morphological effects on antifungal susceptibility of Penicillium marneffei |
|  | Bradykinin-induced chemotaxis of human gliomas requires the activation of KCa3.1 and ClC-3 |
|  | The Frequency, Antifungal Susceptibility and Enzymatic Profiles of Candida Species Isolated from Neutropenic Patients |
|  | Visceral leishmaniasis masquerading as drug-induced pancytopenia in myasthenia gravis |
|  | Trehalose as quantitative biomarker for in vivo diagnosis and treatment follow-up in cryptococcomas |
|  | Balamuthia mandrillaris from soil samples |
|  | Antifungal therapy and management of complications of cryptococcosis due to Cryptococcus gattii |
|  | Intracranial blastomycotic abscess mimicking malignant brain neoplasm: Successful treatment with voriconazole and surgery |
|  | Rhino-Orbital-Cerebral Mucormycosis: A Fatal Complication of Uncontrolled Diabetes Mellitus |
|  | Successful treatment of primary cerebral mucormycosis: Role of microbiologist |
|  | Basic research: Antifungal treatment for glioma? |
|  | Central nervous system histoplasmosis: Multicenter retrospective study on clinical features, diagnostic approach and outcome of treatment |
|  | Central Nervous System Histoplasmosis in Acquired Immunodeficiency Syndrome |
|  | Neurovirulence of Cryptococcus neoformans determined by time course of capsule accumulation and total volume of capsule in the brain |
|  | Paciente inmunocompetente con criptococosis cerebral: reporte de un caso |
|  | Hemichorea in a patient with HIV-associated central nervous system histoplasmosis |
|  | Cerebral candidiasis in a 4-year-old boy after intestinal surgery |
|  | Isolated cerebral aspergillosis in immunocompetent patients |
|  | Disseminated mucormycosis (DM) after pneumonectomy: a case report |
|  | Cryptococcal meningitis post autologous stem cell transplantation |
|  | Cerebral phaeohyphomycosis due to Rhinocladiella mackenziei (formerly Ramichloridium mackenziei): a taxonomic update and review of the literature |
|  | Double Vision and Gait Ataxia in an Immunocompetent 9-Year-Old Girl With Intracranial Phaeohyphomycosis |
|  | Estrogen receptor antagonists are anti-cryptococcal agents that directly bind EF hand proteins and synergize with fluconazole in vivo |
|  | The surgical management of rhinocerebral mucormycosis |
|  | Fatal disseminated aspergillosis in an immunocompetent patient with COVID-19 due to Aspergillus ochraceus |
|  | Puerperal brain cryptococcoma in an HIV-negative woman successfully treated with fluconazole: a case report |
|  | Direct Invasion of the Optic Nerves, Chiasm, and Tracts by Cryptococcus neoformans in an Immunocompetent Host |
|  | Randomized, phase 1/2, double-blind pioglitazone repositioning trial combined with antifungals for the treatment of cryptococcal meningitis - PIO study |
|  | Multimodal Treatment of Rhinocerebral Mucormycosis in a Pediatric Patient With Relapsed Pre-B Acute Lymphoblastic Leukemia |
|  | Progressive disseminated histoplasmosis in an immunocompetent adult: A case report |
|  | Atypical Presentation of Pediatric Systemic Lupus Erythematosus Complicated by Cryptococcal Meningitis |
|  | Multiple Brain Abscesses Due to Aspergillus Fumigatus in a Patient With Liver Cirrhosis: A Case Report |
|  | Diagnosing Ring-Enhancing Lesions in the Brain of a Patient With AIDS Without Brain Biopsy: A Case of Central Nervous System Histoplasmoma |
|  | Progressive disseminated histoplasmosis presenting with cachexia and hypercalcemia |
|  | Gut microbiota depletion affects nutritional and behavioral responses to activity-based anorexia model in a sex-dependent manner |
|  | Rhinocerebral Mucormycosis with Orosinusal Involvement: Diagnostic and Surgical Treatment Guidelines |
|  | [Neonatal renal candidiasis: a case report] |
|  | Successful isavuconazole salvage therapy in a patient with invasive mucormycosis |
|  | A case of central nervous system infection due to Cladophialophora bantiana |
|  | Respiratory Tract Infection Caused by Fonsecaea monophora After Kidney Transplantation |
|  | [Multiple facial nodules revealing disseminated cryptococcosis in an immunocompetent patient] |
|  | Candida Meningitis After Transsphenoidal Surgery: A Single-Institution Case Series and Literature Review |
|  | Stem cell transplantation for acute myeloid leukemia with pulmonary and cerebral mucormycosis |
|  | Cerebral aspergillosis in a patient with leprosy and diabetes: a case report |
|  | Cerebral Scedosporium apiospermum infection presenting with intestinal manifestations |
|  | A case report: primary amoebic meningoencephalitis in a young Zambian adult |
|  | Management of Presumed Endogenous Fungal Endophthalmitis in a Child With Acute Lymphoblastic Leukemia |
|  | Multicentric Cryptococcosis in a Congo African Grey Parrot ( Psittacus erithacus erithacus) |
|  | Central nervous system paracoccidioidomycosis in an AIDS patient: case report |
|  | Fatal cerebral phaeohyphomycosis in an immunocompetent individual due to Thielavia subthermophila |
|  | A tip from the nose: rhinocerebral mucormycosis in a patient with alcoholic liver cirrhosis and cocaine abuse, an uncommon association |
|  | Invasive lung infection by Scedosporium apiospermum in an immunocompetent individual |
|  | Hyponatremia as the Initial Presentation of Cryptococcal Meningitis After Liver Transplantation |
|  | Fatal cryptococcal meningitis in a juvenile lupus erythematosus patient |
|  | Phaeohyphomycosis fungal infections in solid organ transplant recipients: clinical presentation, pathology, and treatment |
|  | A case of cerebral pheohyphomycosis in an immunocompetent patient: emphasis on intraoperative findings |
|  | [A case of histoplasmosis with chronic basilar meningitis diagnosed relatively early] |
|  | A 30-year delayed presentation of disseminated histoplasmosis in a heart transplant recipient: diagnostic challenges in a non-endemic area |
|  | Posaconazole pharmacokinetics in a 2-year-old boy with rhino-cerebral-orbital zygomycosis |
|  | Comparisons of presentations and outcomes of cryptococcal meningitis between patients with and without hepatitis B virus infection |
|  | Cerebral Phaeohyphomycosis due to Cladophialophora bantiana - A Case Report and Review of Literature from India |
|  | Rapid identification of Aspergillus terreus from bronchoalveolar lavage fluid by PCR and electrospray ionization with mass spectrometry |
|  | Hiding in plain sight: a case of chronic disseminated histoplasmosis with central nervous system involvement |
|  | Successful treatment of cerebral aspergillosis: case report of a patient with T-cell large granular lymphocytic leukemia (T-LGL) |
|  | Histoplasmosis with Deep CNS Involvement: Case Presentation with Discussion and Literature Review |
|  | Rhino-orbitocerebral mucormycosis in a patient with idiopathic crescentic glomerulonephritis |
|  | Epidemiological aspects and clinical outcome of patients with Rhinocerebral zygomycosis: a survey in a referral hospital in Iran |
|  | Cerebral aspergillosis with multiple enhancing nodules in the right cerebral hemisphere in the immune-competent patient |
|  | Neuro-oncology: antifungal treatment for glioma? |
|  | [Cryptococcal meningitis in a patient with a ventriculoperitoneal shunt and monitoring for pulmonary sarcoidosis] |
|  | Isolated central nervous system histoplasmosis presenting with ischemic pontine stroke and meningitis in an immune-competent patient |
|  | Skull base osteomyelitis with secondary cavernous sinus thrombosis: a rare presentation of an animate foreign body in the ear |
|  | Pneumonia caused by Talaromyces marneffei in an epidermal growth factor receptor (EGFR) mutation-positive advanced lung adenocarcinoma patient: a case report |
|  | [Fungal keratitis caused by Scedosporium apiospermum: first report from Turkey] |
| Rifaximin | Rifaximin-α reduces gut-derived inflammation and mucin degradation in cirrhosis and encephalopathy: RIFSYS randomised controlled trial |
|  | Small Intestinal Bacterial Overgrowth and Irritable Bowel Syndrome: A Bridge between Functional Organic Dichotomy |
|  | Effects of Alcohol on the Brain in Cirrhosis: Beyond Hepatic Encephalopathy |
|  | The liver-brain axis in liver failure: neuroinflammation and encephalopathy |
|  | Rifaximin for the treatment of irritable bowel syndrome - a drug safety evaluation |
|  | Enhancement of functional connectivity, working memory and inhibitory control on multi-modal brain MR imaging with Rifaximin in Cirrhosis: implications for the gut-liver-brain axis |
|  | Branched-chain amino acids for people with hepatic encephalopathy |
|  | Hepatic encephalopathy |
|  | Fecal Microbial Transplant Capsules Are Safe in Hepatic Encephalopathy: A Phase 1, Randomized, Placebo-Controlled Trial |
|  | Effects of Rifaximin on Central Responses to Social Stress-a Pilot Experiment |
|  | Modulation of the metabiome by rifaximin in patients with cirrhosis and minimal hepatic encephalopathy |
|  | [Hepatic encephalopathy] |
|  | Antibiotics for the treatment of hepatic encephalopathy |
|  | Gut : liver : brain axis: the microbial challenge in the hepatic encephalopathy |
|  | Miniaturized Microbiological Method to Determine the Potency of Rifaximin in Tablets |
|  | The role of microbiota in hepatic encephalopathy |
|  | Management of covert hepatic encephalopathy |
|  | Is irritable bowel syndrome an infectious disease? |
|  | Rifaximin has no effect on hemodynamics in decompensated cirrhosis: A randomized, double-blind, placebo-controlled trial |
|  | Minimal hepatic encephalopathy: A review |
|  | Effects of the antibiotic rifaximin on cortical functional connectivity are mediated through insular cortex |
|  | Disaccharides in the treatment of hepatic encephalopathy |
|  | Treatment of hyperammonemia in liver failure |
|  | Hepatic encephalopathy: how to test and treat |
|  | Mechanistic insight, diagnosis, and treatment of ammonia-induced hepatic encephalopathy |
|  | New treatment options for irritable bowel syndrome with predominant diarrhea |
|  | The burden of minimal hepatic encephalopathy: from diagnosis to therapeutic strategies |
|  | New treatments and therapeutic targets for IBS and other functional bowel disorders |
|  | Rifaximin is an efficacious treatment for the Parkinsonian phenotype of hepatic encephalopathy |
|  | Modulation of the gut microbiota: a focus on treatments for irritable bowel syndrome |
|  | Gut Microbiota Modulation and Fecal Transplantation: An Overview on Innovative Strategies for Hepatic Encephalopathy Treatment |
|  | Randomised clinical trial: rifaximin improves health-related quality of life in cirrhotic patients with hepatic encephalopathy - a double-blind placebo-controlled study |
|  | Design of the Prospective Real-world Outcomes Study of hepatic encephalopathy Patients' Experience on Rifaximin-α (PROSPER): an observational study among 550 patients |
|  | A model of blood-ammonia homeostasis based on a quantitative analysis of nitrogen metabolism in the multiple organs involved in the production, catabolism, and excretion of ammonia in humans |
|  | Evolving concepts: the negative effect of minimal hepatic encephalopathy and role for prophylaxis in patients with cirrhosis |
|  | Development of an experimental rat model of hyperammonemic encephalopathy and evaluation of the effects of rifaximin |
|  | Symptom Severity Following Rifaximin and the Probiotic VSL#3 in Patients with Chronic Pelvic Pain Syndrome (Due to Inflammatory Prostatitis) Plus Irritable Bowel Syndrome |
|  | Reversal of Low-Grade Cerebral Edema After Lactulose/Rifaximin Therapy in Patients with Cirrhosis and Minimal Hepatic Encephalopathy |
|  | Hyperammonaemic encephalopathy following an uncomplicated surgery |
|  | Psychiatric Manifestations of Hyperammonemic Encephalopathy Following Roux-en-Y Gastric Bypass |
|  | Hepatic encephalopathy: cause and possible management with botanicals |
|  | Propranolol-induced hallucinations mimicking encephalopathy in a patient with liver cirrhosis |
|  | Oral administration of trace element magnesium significantly improving the cognition and locomotion in hepatic encephalopathy rats |
|  | Sex is associated with differences in gut microbial composition and function in hepatic encephalopathy |
|  | Efficacy and Safety of Ornithine Phenylacetate for Treating Overt Hepatic Encephalopathy in a Randomized Trial |
|  | Posterior reversible encephalopathy syndrome in alcoholic hepatitis: Hepatic encephalopathy a common theme |
|  | Disabling portosystemic encephalopathy in a non-cirrhotic patient: Successful endovascular treatment of a giant inferior mesenteric-caval shunt via the left internal iliac vein |
| Ridauran | Scavenging of H2O2 by mouse brain mitochondria |
|  | Repurposing auranofin for treatment of Experimental Cerebral Toxoplasmosis |
|  | A conceptually new treatment approach for relapsed glioblastoma: coordinated undermining of survival paths with nine repurposed drugs (CUSP9) by the International Initiative for Accelerated Improvement of Glioblastoma Care |
|  | TDP-43 self-interaction is modulated by redox-active compounds Auranofin, Chelerythrine and Riluzole |
|  | Gold drug auranofin could reduce neuroinflammation by inhibiting microglia cytotoxic secretions and primed respiratory burst |
|  | The antioxidant 2,6-di-tert-butylphenol moiety attenuates the pro-oxidant properties of the auranofin analogue |
|  | Methylglyoxal-Induced Protection Response and Toxicity: Role of Glutathione Reductase and Thioredoxin Systems |
|  | GluR6-FasL-Trx2 mediates denitrosylation and activation of procaspase-3 in cerebral ischemia/reperfusion in rats |
|  | TSC2 Deficiency Unmasks a Novel Necrosis Pathway That Is Suppressed by the RIP1/RIP3/MLKL Signaling Cascade |
|  | Novel protective properties of auranofin: inhibition of human astrocyte cytotoxic secretions and direct neuroprotection |
|  | Neuroprotection of ischemic preconditioning is mediated by thioredoxin 2 in the hippocampal CA1 region following a subsequent transient cerebral ischemia |
|  | Differentiating between apparent and actual rates of H2O2 metabolism by isolated rat muscle mitochondria to test a simple model of mitochondria as regulators of H2O2 concentration |
|  | Differences in Reperfusion-Induced Mitochondrial Oxidative Stress and Cell Death Between Hippocampal CA1 and CA3 Subfields Are Due to the Mitochondrial Thioredoxin System |
|  | CUSP9* treatment protocol for recurrent glioblastoma: aprepitant, artesunate, auranofin, captopril, celecoxib, disulfiram, itraconazole, ritonavir, sertraline augmenting continuous low dose temozolomide |
| Fidaxomicin | Time-kill kinetics of cadazolid and comparator antibacterial agents against different ribotypes of Clostridium difficile |
| Betamethasone | Effects of intra-amniotic lipopolysaccharide and maternal betamethasone on brain inflammation in fetal sheep |
|  | Calcipotriol/Betamethasone Dipropionate Foam: A Review in Plaque Psoriasis |
|  | The effects of betamethasone on allopregnanolone concentrations and brain development in preterm fetal sheep |
|  | The Hypothalamic-Pituitary-Adrenal Axis and the Fetus |
|  | Multiple Antenatal Dexamethasone Treatment Alters Brain Vessel Differentiation in Newborn Mouse Pups |
|  | Fetal immune response to chorioamnionitis |
|  | Sex-dependent expression of brain medullary MAP and PI3 kinases in adult sheep with antenatal betamethasone exposure |
|  | Use of Glucocorticoids for the Fetus and Preterm Infant |
|  | Inflammatory Pseudotumor of the Brain Parenchyma with IgG4 Hypergammaglobulinemia |
|  | Olanzapine and Betamethasone Are Effective for the Treatment of Nausea and Vomiting due to Metastatic Brain Tumors of Rectal Cancer |
|  | Glucocorticoids and fetal programming part 1: Outcomes |
|  | Development of somatosensory-evoked potentials in foetal sheep: effects of betamethasone |
|  | Antenatal betamethasone compared with dexamethasone (betacode trial): a randomized controlled trial |
|  | Prenatal betamethasone does not affect glutamatergic or GABAergic neurogenesis in preterm newborns |
|  | Efficacy and safety of fixed-dose combination calcipotriol/betamethasone dipropionate foam for the treatment of psoriasis |
|  | Fetal betamethasone exposure attenuates angiotensin-(1-7)-Mas receptor expression in the dorsal medulla of adult sheep |
|  | Roles of glucocorticoids in human parturition: a controversial fact? |
|  | Different corticosteroids and regimens for accelerating fetal lung maturation for women at risk of preterm birth |
|  | Effects of a single course of prenatal betamethasone on dendritic development in dentate gyrus granular neurons and on spatial memory in rat offspring |
|  | Do Prenatal Corticosteroids Affect Brain Maturation of the Premature Infant? An Electroencephalography Study |
|  | Estradiol does not affect spasms in the betamethasone-NMDA rat model of infantile spasms |
|  | The effect of intra-articular injection of betamethasone acetate/betamethasone sodium phosphate at the knee joint on the hypothalamic-pituitary-adrenal axis: a case-controlled study |
|  | Antenatal betamethasone exposure is associated with lower ANG-(1-7) and increased ACE in the CSF of adult sheep |
|  | The effects of perinatal steroid therapy on growth factor levels during different stages of the developing brain |
|  | Basic mechanisms of catastrophic epilepsy -- overview from animal models |
|  | Antenatal glucocorticoid treatment is associated with diurnal cortisol regulation in term-born children |
|  | Early efficacy and safety data with fixed-dose combination calcipotriol/betamethasone dipropionate foam attributed to mechanism of absorption and steroid potency |
|  | A Novel Aerosol Foam Formulation of Calcipotriol and Betamethasone Has No Impact on HPA Axis and Calcium Homeostasis in Patients With Extensive Psoriasis Vulgaris |
|  | Pathogenesis and new candidate treatments for infantile spasms and early life epileptic encephalopathies: A view from preclinical studies |
|  | Long-term impact of intrauterine neuroinflammation and treatment with magnesium sulphate and betamethasone: Sex-specific differences in a preterm labor murine model |
|  | Effect of calcipotriene plus betamethasone dipropionate topical suspension on the hypothalamic-pituitary-adrenal axis and calcium homeostasis in subjects with extensive psoriasis vulgaris: an open, non-controlled, 8-week trial |
|  | Safety and efficacy of calcipotriene plus betamethasone dipropionate topical suspension in the treatment of extensive scalp psoriasis in adolescents ages 12 to 17 years |
|  | Enhanced long-term potentiation in mature rats in a model of epileptic spasms with betamethasone-priming and postnatal N-methyl-D-aspartate administration |
|  | ACTH vs steroids for the treatment of acute gout in hospitalized patients: a randomized, open label, comparative study |
|  | Clobetasol promotes remyelination in a mouse model of neuromyelitis optica |
|  | A rat model of ataxia-telangiectasia: evidence for a neurodegenerative phenotype |
|  | Postnatal glucocorticoid-induced hypomyelination, gliosis, and neurologic deficits are dose-dependent, preparation-specific, and reversible |
|  | Characteristics of particulate and non-particulate corticosteroids. Indications for their use in chronic pain treatments |
|  | Glucocorticoid programming of the fetal male hippocampal epigenome |
|  | Drug-based modulation of endogenous stem cells promotes functional remyelination in vivo |
|  | Steroids in aminoglycoside-containing ear drops: do they reduce cochlear toxicity? |
|  | Altered expression of KCC2 in GABAergic interneuron contributes prenatal stress-induced epileptic spasms in infant rat |
|  | Serum cortisol and adrenocorticotrophic hormone (ACTH) in infants receiving topical and subconjunctival corticosteroids following cataract surgery |
|  | Correlation between auditory brainstem response and hearing prognosis in idiopathic sudden sensorineural hearing loss patients |
|  | PSO-LONG: Design of a Novel, 12-Month Clinical Trial of Topical, Proactive Maintenance with Twice-Weekly Cal/BD Foam in Psoriasis |
|  | Effects of glucocorticoid treatment given in early or late gestation on growth and development in sheep |
|  | Reduction of experimental cerebral malaria and its related proinflammatory responses by the novel liposome-based β-methasone nanodrug |
|  | Targeted delivery of glucocorticoids to macrophages in a mouse model of multiple sclerosis using inorganic-organic hybrid nanoparticles |
|  | Glucocorticoids promote neural progenitor cell proliferation derived from human induced pluripotent stem cells |
|  | Enhanced activity of an angiotensin-(1-7) neuropeptidase in glucocorticoid-induced fetal programming |
|  | Revealing the influence of glucocorticoid treatment on the excretion of anabolic-androgenic steroids in horses through in vitro digestive simulations and an in vivo case study |
|  | Steroid treatment in Ataxia-Telangiectasia induces alterations of functional magnetic resonance imaging during prono-supination task |
|  | Overexpression of vascular endothelial growth factor in the germinal matrix induces neurovascular proteases and intraventricular hemorrhage |
|  | Acute Tetraparesis with Respiratory Failure after Steroid Administration in a Patient with a Dural Arteriovenous Fistula at the Craniocervical Junction |
|  | Cushing's Syndrome in a 6-month-old Boy: A Rare Side-effect due to Inadequate use of Topical Corticosteroids |
|  | Humanized mice, a new model to study the influence of drug treatment on neonatal sepsis |
|  | Everolimus improves memory and learning while worsening depressive- and anxiety-like behavior in an animal model of depression |
|  | Chapter for antenatal steroids - Treatment drift for a potent therapy with unknown long-term safety seminars in fetal and neonatal medicine |
|  | Effects of antenatal synthetic glucocorticoid on glucocorticoid receptor binding, DNA methylation, and genome-wide mRNA levels in the fetal male hippocampus |
|  | Anti-MOG antibody-positive ADEM following infectious mononucleosis due to a primary EBV infection: a case report |
|  | Glucocorticosteroids in nano-sterically stabilized liposomes are efficacious for elimination of the acute symptoms of experimental cerebral malaria |
|  | Bleomycin induced flagellate erythema in a patient with thalamic mixed germ cell tumour: Report of a rare adverse effect |
|  | [Chronic lymphocytic inflammation with pontine perivascular enhancement responsive to steroids (CLIPPERS) associated with swelling in the brainstem: a case report] |
| Cromolyn | Evaluation of Fluorinated Cromolyn Derivatives as Potential Therapeutics for Alzheimer's Disease |
|  | Cromolyn Reduces Levels of the Alzheimer's Disease-Associated Amyloid β-Protein by Promoting Microglial Phagocytosis |
|  | Suppression of Brain Mast Cells Degranulation Inhibits Microglial Activation and Central Nervous System Inflammation |
|  | Activated brain mast cells contribute to postoperative cognitive dysfunction by evoking microglia activation and neuronal apoptosis |
|  | Cerebral mast cells contribute to postoperative cognitive dysfunction by promoting blood brain barrier disruption |
|  | Contribution of mast cells to injury mechanisms in a mouse model of pediatric traumatic brain injury |
|  | Responses of dural mast cells in concussive and blast models of mild traumatic brain injury in mice: Potential implications for post-traumatic headache |
|  | Sodium cromoglycate reduces short- and long-term consequences of status epilepticus in rats |
|  | Thalamic mast cell activity is associated with sign-tracking behavior in rats |
|  | Cerebral Mast Cells Participate In Postoperative Cognitive Dysfunction by Promoting Astrocyte Activation |
|  | Mast cells in the paraventricular nucleus participate in visceral hypersensitivity induced by neonatal maternal separation |
|  | Effect of hyperthermia on histamine blood level and convulsive behavior in infant rats |
|  | Stress induces more serious barrier dysfunction in follicle-associated epithelium than villus epithelium involving mast cells and protease-activated receptor-2 |
|  | Mast cells mediate early neutrophil recruitment and exhibit anti-inflammatory properties via the formyl peptide receptor 2/lipoxin A(4) receptor |
| Beclomethasone | Structures of the glucocorticoid-bound adhesion receptor GPR97-G(o) complex |
|  | Safety of intranasal corticosteroids |
|  | Opportunity and challenges of nasal powders: Drug formulation and delivery |
|  | High-Throughput Screening for Identification of Blood-Brain Barrier Integrity Enhancers: A Drug Repurposing Opportunity to Rectify Vascular Amyloid Toxicity |
|  | Parenteral nanosuspensions: a brief review from solubility enhancement to more novel and specific applications |
|  | Once-daily treatment with beclomethasone dipropionate nasal aerosol does not affect hypothalamic-pituitary-adrenal axis function |
|  | No hypothalamic-pituitary-adrenal function effect with beclomethasone dipropionate nasal aerosol, based on 24-hour serum cortisol in pediatric allergic rhinitis |
|  | Impact of study design on the evaluation of inhaled and intranasal corticosteroids' effect on hypothalamic-pituitary-adrenal axis function, part I: general overview of HPA axis study design |
|  | Metabolic and reproductive effects of relatively low concentrations of beclomethasone dipropionate, a synthetic glucocorticoid, on fathead minnows |
|  | Different corticosteroids and regimens for accelerating fetal lung maturation for women at risk of preterm birth |
|  | Hypothalamic-pituitary-adrenal axis suppression by inhaled or nasal corticosteroids in HIV-infected patients |
|  | Eye-specific retinogeniculate segregation proceeds normally following disruption of patterned spontaneous retinal activity |
|  | Age-related changes in tonic activation of presynaptic versus extrasynaptic γ-amniobutyric acid type B receptors in rat medial prefrontal cortex |
| Triamcinolone | INTRAVITREAL TRIAMCINOLONE IN SUSAC SYNDROME |
|  | Hypothalamo-pituitary-adrenal axis after a single epidural triamcinolone injection |
|  | Reduction in the free radical status and clinical benefit of repeated intrathecal triamcinolone acetonide application in patients with progressive multiple sclerosis |
|  | Effect of intranasal triamcinolone acetonide on basal hypothalamic-pituitary-adrenal axis function in children with allergic rhinitis |
|  | Reduced Fragmentation of IGFBP-2 and IGFBP-3 as a Potential Mechanism for Decreased Ratio of IGF-II to IGFBPs in Cerebrospinal Fluid in Response to Repeated Intrathecal Administration of Triamcinolone Acetonide in Patients With Multiple Sclerosis |
|  | Impact of study design on the evaluation of inhaled and intranasal corticosteroids' effect on hypothalamic-pituitary-adrenal axis function, part I: general overview of HPA axis study design |
|  | A prospective observational study evaluating hypothalamic-pituitary-adrenal axis alteration and efficacy of intramuscular triamcinolone acetonide for steroid-responsive dermatologic disease |
|  | Vibration Anesthesia for Pain Reduction During Intralesional Steroid Injection for Keloid Treatment |
|  | Characteristics of particulate and non-particulate corticosteroids. Indications for their use in chronic pain treatments |
|  | Safety and efficacy of intralesional steroid injection for aggressive fibromatosis |
|  | Transient facial nerve palsy after occipital nerve block: a case report |
|  | Randomized controlled trial of steroid-soaked absorbable calcium alginate nasal packing following endoscopic sinus surgery |
|  | Episcleral drug film for better-targeted ocular drug delivery and controlled release using multilayered poly-ε-caprolactone (PCL) |
|  | Serous retinal detachment and cystoid macular edema in a patient with Wyburn-Mason syndrome |
|  | Twice-daily application of topical corticosteroids with a squirt system for patients with steroid-responsive olfactory impairment |
|  | Effects of intraoperatively applied glucocorticoid hydrogels on residual hearing and foreign body reaction in a guinea pig model of cochlear implantation |
| Rifamycin | Brucellosis |
|  | Rifaximin-α reduces gut-derived inflammation and mucin degradation in cirrhosis and encephalopathy: RIFSYS randomised controlled trial |
|  | RIFAMPICIN: an antibiotic with brain protective function |
|  | Tuberculous meningitis |
|  | Therapeutic role of rifampicin in Alzheimer's disease |
|  | A Clinical Study of Miliary Brain Tuberculomas in China |
|  | Treatment of hyperammonemia in liver failure |
|  | Hepatic encephalopathy: how to test and treat |
|  | New treatment options for irritable bowel syndrome with predominant diarrhea |
|  | Modulation of the gut microbiota: a focus on treatments for irritable bowel syndrome |
|  | Rifaximin for the treatment of irritable bowel syndrome - a drug safety evaluation |
|  | A Woman With Confusion and Blurred Vision |
|  | Encephalopathy in an adult with cat-scratch disease |
|  | Spontaneous methicillin-resistant Staphylococcus aureus (MRSA) meningitis |
|  | Pregnane X receptor upregulates ABC-transporter Abcg2 and Abcb1 at the blood-brain barrier |
|  | Evolving concepts: the negative effect of minimal hepatic encephalopathy and role for prophylaxis in patients with cirrhosis |
|  | Development and Validation of an In-Cell Western for Quantifying P-Glycoprotein Expression in Human Brain Microvascular Endothelial (hCMEC/D3) Cells |
|  | Modulation of P-glycoprotein at the Human Blood-Brain Barrier by Quinidine or Rifampin Treatment: A Positron Emission Tomography Imaging Study |
|  | Xpert MTB/RIF Ultra: the long-awaited game changer for tuberculous meningitis? |
|  | Enhancement of functional connectivity, working memory and inhibitory control on multi-modal brain MR imaging with Rifaximin in Cirrhosis: implications for the gut-liver-brain axis |
|  | Donepezil loaded PLGA-b-PEG nanoparticles: their ability to induce destabilization of amyloid fibrils and to cross blood brain barrier in vitro |
|  | Miniaturized Microbiological Method to Determine the Potency of Rifaximin in Tablets |
|  | Psychiatric Manifestations of Hyperammonemic Encephalopathy Following Roux-en-Y Gastric Bypass |
|  | Deep gray matter involvement in neurobrucellosis |
|  | Effects of the antibiotic rifaximin on cortical functional connectivity are mediated through insular cortex |
|  | Effects of Rifaximin on Central Responses to Social Stress-a Pilot Experiment |
|  | Rifampicin attenuates experimental autoimmune encephalomyelitis by inhibiting pathogenic Th17 cells responses |
|  | MicroRNA hsa-miR-25-3p suppresses the expression and drug induction of CYP2B6 in human hepatocytes |
|  | Propranolol-induced hallucinations mimicking encephalopathy in a patient with liver cirrhosis |
|  | Sex is associated with differences in gut microbial composition and function in hepatic encephalopathy |
|  | The role of Xpert MTB/RIF in diagnosing pulmonary tuberculosis in post-mortem tissues |
|  | Rifampicin is a candidate preventive medicine against amyloid-β and tau oligomers |
|  | Modulation of the metabiome by rifaximin in patients with cirrhosis and minimal hepatic encephalopathy |
|  | Rifampicin Suppresses Amyloid-β Accumulation Through Enhancing Autophagy in the Hippocampus of a Lipopolysaccharide-Induced Mouse Model of Cognitive Decline |
|  | Sigma-1 receptor knockout increases α-synuclein aggregation and phosphorylation with loss of dopaminergic neurons in substantia nigra |
|  | Posterior reversible encephalopathy syndrome in alcoholic hepatitis: Hepatic encephalopathy a common theme |
|  | Optimized In Vitro Antibiotic Susceptibility Testing Method for Small-Colony Variant Staphylococcus aureus |
|  | Rifampicin pre-treatment inhibits the toxicity of rotenone-induced PC12 cells by enhancing sumoylation modification of α-synuclein |
|  | Rifampicin improves neuronal apoptosis in LPS-stimulated co‑cultured BV2 cells through inhibition of the TLR-4 pathway |
|  | Rifaximin is an efficacious treatment for the Parkinsonian phenotype of hepatic encephalopathy |
|  | Development of an experimental rat model of hyperammonemic encephalopathy and evaluation of the effects of rifaximin |
|  | Pharmacological induction of heat shock proteins ameliorates toxicity of mutant PKCγ in spinocerebellar ataxia type 14 |
|  | Management of paradoxical response in pediatric tubercular meningitis with methylprednisolone |
|  | Rifaximin has no effect on hemodynamics in decompensated cirrhosis: A randomized, double-blind, placebo-controlled trial |
|  | Rifampicin ameliorates lipopolysaccharide-induced cognitive and motor impairments via inhibition of the TLR4/MyD88/NF-κB signaling pathway in mice |
|  | Development of a stretch-induced neurotrauma model for medium-throughput screening in vitro: identification of rifampicin as a neuroprotectant |
|  | Isoniazid- and streptomycin-resistant miliary tuberculosis complicated by intracranial tuberculoma in a Japanese infant |
|  | Studies on the safety and the tissue distribution of inhaled high-dose amorphous and crystalline rifampicin in a rat model |
|  | Mechanisms of P-gp inhibition and effects on membrane fluidity of a new rifampicin derivative, 1,8-dibenzoyl-rifampicin |
|  | Multidrug resistance protein 1 reduces the aggregation of mutant huntingtin in neuronal cells derived from the Huntington's disease R6/2 model |
|  | Rifampicin ameliorates lithium-pilocarpine-induced seizures, consequent hippocampal damage and memory deficit in rats: Impact on oxidative, inflammatory and apoptotic machineries |
|  | Shunt-related intracranial abscess caused by Staphylococcus lugdunensis in a hydranencephalic patient |
|  | Orally delivered water soluble Coenzyme Q10 (Ubisol-Q10) blocks on-going neurodegeneration in rats exposed to paraquat: potential for therapeutic application in Parkinson's disease |
|  | Severe hyponatremia and MRI point to diagnosis of tuberculous meningitis in the Southwest USA |
|  | Cloning of cytochrome P450 3A137 complementary DNA in silver carp and expression induction by ionic liquid |
|  | Has the introduction of antibiotic-impregnated external ventricular drain catheters changed the nature of the microorganisms cultured in patients with drain-related infection? A single neurosurgical centre's experience |
|  | Symptom Severity Following Rifaximin and the Probiotic VSL#3 in Patients with Chronic Pelvic Pain Syndrome (Due to Inflammatory Prostatitis) Plus Irritable Bowel Syndrome |
|  | Intracranial abscess due to Mycobacterium avium complex in an immunocompetent host: a case report |
|  | Development of novel rifampicin-derived P-glycoprotein activators/inducers. synthesis, in silico analysis and application in the RBE4 cell model, using paraquat as substrate |
|  | Randomised clinical trial: rifaximin improves health-related quality of life in cirrhotic patients with hepatic encephalopathy - a double-blind placebo-controlled study |
|  | In vitro characterization of axitinib interactions with human efflux and hepatic uptake transporters: implications for disposition and drug interactions |
|  | Rothia mucilaginosa, rarely isolated pathogen as an etiological factor of infection of soft tissues in young, healthy woman |
|  | Silver-impregnated, antibiotic-impregnated or non-impregnated ventriculoperitoneal shunts to prevent shunt infection: the BASICS three-arm RCT |
| Papers related to AD | |
| Selected anti-inflammatory drugs | |

**Supplementary Table 2**

| Antiinflammatory Drugs | Paper Names |
| --- | --- |
| Flurbiprofen | Nanoparticulate flurbiprofen reduces amyloid-β42 generation in an in vitro blood-brain barrier model |
|  | Role of serum albumin as a nanoparticulate carrier for nose-to-brain delivery of R-flurbiprofen: implications for the treatment of Alzheimer's disease |
|  | R-flurbiprofen improves tau, but not Aß pathology in a triple transgenic model of Alzheimer's disease |
|  | Nasal NSAIDs for Alzheimer's Disease |
|  | Design, synthesis and evaluation of 4'-OH-flurbiprofen-chalcone hybrids as potential multifunctional agents for Alzheimer's disease treatment |
|  | Intranasal delivery of nanoparticle encapsulated tarenflurbil: A potential brain targeting strategy for Alzheimer's disease |
|  | Design, synthesis and evaluation of tacrine-flurbiprofen-nitrate trihybrids as novel anti-Alzheimer's disease agents |
|  | New flurbiprofen derivatives: synthesis, membrane affinity and evaluation of in vitro effect on β-amyloid levels |
|  | Combination therapy in a transgenic model of Alzheimer's disease |
|  | Aging Enables Ca2+ Overload and Apoptosis Induced by Amyloid-β Oligomers in Rat Hippocampal Neurons: Neuroprotection by Non-Steroidal Anti-Inflammatory Drugs and R-Flurbiprofen in Aging Neurons |
|  | Multi-target action of the novel anti-Alzheimer compound CHF5074: in vivo study of long term treatment in Tg2576 mice |
| Piroxicam | Determining the Molecular Pathways Underlying the Protective Effect of Non-Steroidal Anti-Inflammatory Drugs for Alzheimer's Disease: A Bioinformatics Approach |
| Ibuprofen | The effects of aging, housing and ibuprofen treatment on brain neurochemistry in a triple transgene Alzheimer's disease mouse model using magnetic resonance spectroscopy and imaging |
|  | Ibuprofen inhibits the synaptic failure induced by the amyloid-β peptide in hippocampal neurons |
|  | Nasal NSAIDs for Alzheimer's Disease |
|  | ROS-responsive and multifunctional anti-Alzheimer prodrugs: Tacrine-ibuprofen hybrids via a phenyl boronate linker |
|  | Ibuprofen and lipoic acid conjugate neuroprotective activity is mediated by Ngb/Akt intracellular signaling pathway in Alzheimer's disease rat model |
|  | New potential strategies for Alzheimer's disease prevention: pegylated biodegradable dexibuprofen nanospheres administration to APPswe/PS1dE9 |
|  | Identification and modification of amyloid-independent phenotypes of APOE4 mice |
|  | Cyclooxygenase inhibition targets neurons to prevent early behavioural decline in Alzheimer's disease model mice |
|  | Alzheimer's Disease and Intranasal Fluticasone Propionate in the FDA MedWatch Adverse Events Database |
|  | R-flurbiprofen improves tau, but not Aß pathology in a triple transgenic model of Alzheimer's disease |
|  | Deficiency in either COX-1 or COX-2 genes does not affect amyloid beta protein burden in amyloid precursor protein transgenic mice |
| Hydrocortisone | Hypercortisolemia and glucocorticoid receptor-signaling insufficiency in Alzheimer's disease initiation and development |
|  | Associations between Sleep, Cortisol Regulation, and Diet: Possible Implications for the Risk of Alzheimer Disease |
|  | Effects of naproxen on immune responses in a colchicine-induced rat model of Alzheimer's disease |
|  | Sex differences in Alzheimer's disease risk: are we looking at the wrong hormones? |
|  | Increased Total Urinary Cortisol (tUC) and Serum Brain-derived Neurotrophic Factor (BDNF) Ratio in Alzheimer Disease (AD)-affected Patients |
|  | Age Effects on Cognitive and Physiological Parameters in Familial Caregivers of Alzheimer's Disease Patients |
|  | Naringin ameliorates memory deficits and exerts neuroprotective effects in a mouse model of Alzheimer's disease by regulating multiple metabolic pathways |
|  | Anterior hypopituitarism in a patient with amyloidosis secondary to Crohn's disease: a case report |
| Prednisolone | [A case of inflammatory cerebral amyloid angiopathy with white matter lesions appearing after brain biopsy] |
| Celecoxib | Sub-chronic celecoxib prevents soluble beta amyloid-induced depressive-like behaviour in rats |
|  | Celecoxib Prevents Cognitive Impairment and Neuroinflammation in Soluble Amyloid β-treated Rats |
|  | Erythrocyte membrane-encapsulated celecoxib improves the cognitive decline of Alzheimer's disease by concurrently inducing neurogenesis and reducing apoptosis in APP/PS1 transgenic mice |
|  | Identification and modification of amyloid-independent phenotypes of APOE4 mice |
|  | Investigations on 16-Arylideno Steroids as a New Class of Neuroprotective Agents for the Treatment of Alzheimer's and Parkinson's Diseases |
|  | Studies on 16,17-Pyrazoline Substituted Heterosteroids as Anti-Alzheimer and Anti-Parkinsonian Agents Using LPS Induced Neuroinflammation Models of Mice and Rats |
| Etodolac | High-Throughput Screening for Identification of Blood-Brain Barrier Integrity Enhancers: A Drug Repurposing Opportunity to Rectify Vascular Amyloid Toxicity |
| Nabumetone | Determining the Molecular Pathways Underlying the Protective Effect of Non-Steroidal Anti-Inflammatory Drugs for Alzheimer's Disease: A Bioinformatics Approach |
| Dexamethasone | Knockout of 5-lipoxygenase prevents dexamethasone-induced tau pathology in 3xTg mice |
|  | Activity of pomalidomide in patients with immunoglobulin light-chain amyloidosis |
|  | Evaluation of patients with cardiac amyloidosis using echocardiography, ECG and right heart catheterization |
|  | Studies on 16,17-Pyrazoline Substituted Heterosteroids as Anti-Alzheimer and Anti-Parkinsonian Agents Using LPS Induced Neuroinflammation Models of Mice and Rats |
|  | Spontaneous convexity subarachnoid haemorrhage: Clinical series of 3 patients with associated cerebral amyloid angiopathy |
|  | Total Artificial Heart Implantation as a Bridge to Heart Transplantation in an Active Duty Service Member With Amyloid Cardiomyopathy |
|  | Sequential cyclophosphamide-bortezomib-dexamethasone unmasks the harmful cardiac effect of dexamethasone in primary light-chain cardiac amyloidosis |
|  | Light chain amyloidosis 2012: a new era |
|  | Subchronic glucocorticoid receptor inhibition rescues early episodic memory and synaptic plasticity deficits in a mouse model of Alzheimer's disease |
|  | Sex Hormone Depletion Augments Glucocorticoid Induction of Tau Hyperphosphorylation in Male Rat Brain |
|  | Multimodal nanoprobes to target cerebrovascular amyloid in Alzheimer's disease brain |
|  | Immunoglobulin light chain amyloidosis: 2013 update on diagnosis, prognosis, and treatment |
|  | Immunoglobulin light chain amyloidosis: 2016 update on diagnosis, prognosis, and treatment |
|  | Outcomes by Cardiac Stage in Patients With Newly Diagnosed AL Amyloidosis: Phase 3 ANDROMEDA Trial |
|  | Immunoglobulin light chain amyloidosis: 2018 Update on diagnosis, prognosis, and treatment |
|  | Cerebral amyloid angiopathy-related inflammation with epilepsy mimicking a presentation of brain tumor: A case report and review of the literature |
|  | Investigations on 16-Arylideno Steroids as a New Class of Neuroprotective Agents for the Treatment of Alzheimer's and Parkinson's Diseases |
| Prednisone | Immunoglobulin light chain amyloidosis: 2016 update on diagnosis, prognosis, and treatment |
|  | Immunoglobulin light chain amyloidosis: 2013 update on diagnosis, prognosis, and treatment |
|  | Immunoglobulin light chain amyloidosis: 2014 update on diagnosis, prognosis, and treatment |
|  | Case 232: Amyloid β-related Angiitis |
|  | Alzheimer's Disease and Intranasal Fluticasone Propionate in the FDA MedWatch Adverse Events Database |
|  | Spontaneous convexity subarachnoid haemorrhage: Clinical series of 3 patients with associated cerebral amyloid angiopathy |
| Dexibuprofen | New potential strategies for Alzheimer's disease prevention: pegylated biodegradable dexibuprofen nanospheres administration to APPswe/PS1dE9 |
| Valdecoxib | Modulation of LOX and COX pathways via inhibition of amyloidogenesis contributes to mitoprotection against β-amyloid oligomer-induced toxicity in an animal model of Alzheimer's disease in rats |
| Etoricoxib | Cox-2 Plays a Vital Role in the Impaired Anxiety Like Behavior in Colchicine Induced Rat Model of Alzheimer Disease |
|  | Role of cox-2 mediated neuroinflammation on the neurodegeneration and cognitive impairments in colchicine induced rat model of Alzheimer's Disease |
| Meloxicam | Co-nanoencapsulated meloxicam and curcumin improves cognitive impairment induced by amyloid-beta through modulation of cyclooxygenase-2 in mice |
|  | Meloxicam-loaded nanocapsules as an alternative to improve memory decline in an Alzheimer's disease model in mice: involvement of Na(+), K(+)-ATPase |
|  | Amyloid-β peptide absence in short term effects on kinase activity of energy metabolism in mice hippocampus and cerebral cortex |
| Cromolyn | Evaluation of Fluorinated Cromolyn Derivatives as Potential Therapeutics for Alzheimer's Disease |
|  | Cromolyn Reduces Levels of the Alzheimer's Disease-Associated Amyloid β-Protein by Promoting Microglial Phagocytosis |
| Beclomethasone | High-Throughput Screening for Identification of Blood-Brain Barrier Integrity Enhancers: A Drug Repurposing Opportunity to Rectify Vascular Amyloid Toxicity |
| Rifamycin | Therapeutic role of rifampicin in Alzheimer's disease |
|  | Donepezil loaded PLGA-b-PEG nanoparticles: their ability to induce destabilization of amyloid fibrils and to cross blood brain barrier in vitro |
|  | Rifampicin is a candidate preventive medicine against amyloid-β and tau oligomers |
|  | Rifampicin Suppresses Amyloid-β Accumulation Through Enhancing Autophagy in the Hippocampus of a Lipopolysaccharide-Induced Mouse Model of Cognitive Decline |
|  |  |
| literature evidence of both AD and amyloid |  |
| literarture evidence of amyloid |  |
| literature evidence of AD |  |

**Supplementary Table 3**

| **Receptor** | **Ligands** | **BFE** | Interacting Residues | **H-bond** | | **van der Waals** | **C-H Bond** | | **Alkyl** | | **Pi-Alkyl** | | **Pi-Donor H-bond** | | **Pi-Sigma** | | **Pi-Pi Stacked** | | **Pi-Pi T-shaped** | | **Pi-Sulfur** | | **Pi-Lone Pair** | | **Halogen (F)** | | **Attractive Charge** | |
| --- | --- | --- | --- | --- | --- | --- | --- | --- | --- | --- | --- | --- | --- | --- | --- | --- | --- | --- | --- | --- | --- | --- | --- | --- | --- | --- | --- | --- |
|  |  |  |  | **Residue** | **No. of bonds** | **Residue** | **Residue** | **No. of bonds** | **Residue** | **No. of bonds** | **Residue** | **No. of bonds** | **Residue** | **No. of bonds** | **Residue** | **No. of bonds** | **Residue** | **No. of bonds** | **Residue** | **No. of bonds** | **Residue** | **No. of bonds** | **Residue** | **No. of bonds** | **Residue** | **No. of bonds** | **Residue** | **No. of bonds** |
| WT | Prednisone | -6.65 | VAL18,PHE19,PHE20,ALA21,GLU22,VAL24,ALA30,GLY33,LEU34 | GLU22 | 2 | ALA21 | VAL18 | **1** | VAL24 | 1 | PHE19 | 1 |  |  | PHE19 | 1 |  |  |  |  |  |  |  |  |  |  |  |  |
|  |  |  |  |  |  | GLY33 |  |  | ALA30 | 1 |  |  |  |  |  |  |  |  |  |  |  |  |  |  |  |  |  |  |
|  |  |  |  |  |  |  |  |  | LEU34 | 1 |  |  |  |  |  |  |  |  |  |  |  |  |  |  |  |  |  |  |
|  | Valdecoxib | -6.41 | VAL18,PHE19,PHE20,ALA21,GLU22,VAL24,ALA30,GLY33,LEU34, VAL36,GLY37 | PHE2O | 1 | GLY29 | ALA3O | **1** | ALA21 | 1 | PHE19 | 2 |  |  |  |  |  |  |  |  |  |  |  |  |  |  |  |  |
|  |  |  |  | GLU22 | 1 | GLY33 |  |  | VAL24 | 2 |  |  |  |  |  |  |  |  |  |  |  |  |  |  |  |  |  |  |
|  |  |  |  | LEU34 | 1 | GLY37 |  |  |  |  |  |  |  |  |  |  |  |  |  |  |  |  |  |  |  |  |  |  |
|  | Celecoxib | -6.33 | VAL18,PHE19,PHE20,ALA21,GLU22,VAL24,GLY33,LEU34,VAL36,GLY37 | VAL18 | 1 | GLU22 | GLY33 | 1 | ALA21 | 2 |  |  |  |  |  |  | PHE19 | 2 |  |  |  |  |  |  | GLY33 | 1 |  |  |
|  |  |  |  |  | 1 | LEU34 |  |  | VAL24 | 2 |  |  |  |  |  |  |  |  |  |  |  |  |  |  | VAL36 | 1 |  |  |
|  |  |  |  | GLY37 | 1 |  |  |  |  |  |  |  |  |  |  |  |  |  |  |  |  |  |  |  |  |  |  |  |
|  | Etoricoxib | -6.15 | SER8,GLY9,TYR10,GLU11,VAL12,HIS13 | GLY9 | 1 | SER8 | SER8 | 1 | VAL12 | 1 |  |  |  |  |  |  |  |  | HIS13 | 1 | TYR10 | 1 |  |  |  |  |  |  |
|  |  |  |  |  |  | HIS13 | HIS13 | 1 | HIS13 | 1 |  |  |  |  |  |  |  |  |  |  |  |  |  |  |  |  |  |  |
|  | Meloxicam | -6.13 | VAL18,PHE19,PHE20,ALA21,GLU22,VAL24,GLY33,LEU34 | PHE20 | 2 | PHE19 |  |  | VAL18 | 1 |  |  |  |  |  |  |  |  |  |  |  |  |  |  |  |  |  |  |
|  |  |  |  | GLU22 | 1 | ALA21 |  |  | VAL24 | 1 |  |  |  |  |  |  |  |  |  |  |  |  |  |  |  |  |  |  |
|  |  |  |  |  |  | GLY33 |  |  | LEU34 | 1 |  |  |  |  |  |  |  |  |  |  |  |  |  |  |  |  |  |  |
|  | Piroxicam | -6.09 | VAL18,PHE19,PHE20,ALA21,GLU22,VAL24,LEU34 | PHE20 | 1 |  |  |  |  |  |  |  |  |  |  |  |  |  |  |  |  |  |  |  |  |  |  |  |
|  |  |  |  | GLU22 | 1 |  |  |  |  |  |  |  |  |  |  |  |  |  |  |  |  |  |  |  |  |  |  |  |
|  |  |  |  |  |  |  |  |  |  |  |  |  |  |  |  |  |  |  |  |  |  |  |  |  |  |  |  |  |
|  | Hydrocortisone | -5.7 | GLN15,LYS16,LEU17,VAL18,PHE19,GLY37,VAL40,ILE41 | GLN15 | 1 |  |  |  |  |  |  |  |  |  |  |  |  |  |  |  |  |  |  |  |  |  |  |  |
|  |  |  |  | LYS16 | 1 |  |  |  |  |  |  |  |  |  |  |  |  |  |  |  |  |  |  |  |  |  |  |  |
|  |  |  |  |  |  |  |  |  |  |  |  |  |  |  |  |  |  |  |  |  |  |  |  |  |  |  |  |  |
|  | Prednisolone | -5.62 | GLN15,LYS16,LEU17,VAL18,PHE19,VAL40,ILE41 |  | 1 |  |  |  |  |  |  |  |  |  |  |  |  |  |  |  |  |  |  |  |  |  |  |  |
|  | Dexamethasone | -5.36 | LEU 17,VAL18,PHE19, ,GLY37,VAL40, ILE 41 |  |  |  |  |  |  |  |  |  |  |  |  |  |  |  |  |  |  |  |  |  |  |  |  |  |
|  | Beclomethasone | -5.24 | PHE19,PHE20,GLU22,VAL24,ALA30,,GLY33,LEU34,VAL36,GLY37,VAL40 |  |  |  |  |  |  |  |  |  |  |  |  |  |  |  |  |  |  |  |  |  |  |  |  |  |
|  | Rifamycin | -5.16 | HIS13,HIS14,GLN15,LYS16,VAL18,PHE20,ALA21,GLU22 | LYS16 | 1 |  |  |  |  |  |  |  |  |  |  |  |  |  |  |  |  |  |  |  |  |  |  |  |
|  | Nabumetone | -5.07 | VAL18,PHE19,PHE20,ALA21,GLU22,VAL24,ALA30,GLY33,LEU34 | PHE20 | 1 |  |  |  |  |  |  |  |  |  |  |  |  |  |  |  |  |  |  |  |  |  |  |  |
|  | Flurbiprofen | -4.94 | HIS13,HIS14,GLN15,LYS16,VAL18 |  | 1 |  |  |  |  |  |  |  |  |  |  |  |  |  |  |  |  |  |  |  |  |  |  |  |
|  | Ibuprofen | -4.21 | HIS13,HIS14,GLN15,LYS16,VAL18,PHE20 |  |  |  |  |  |  |  |  |  |  |  |  |  |  |  |  |  |  |  |  |  |  |  |  |  |
|  | Dexibuprofen | -4.09 | HIS13,HIS14,GLN15,LYS16,VAL18,PHE20 | LYS16 | 2 |  |  |  |  |  |  |  |  |  |  |  |  |  |  |  |  |  |  |  |  |  |  |  |
|  | Cromolyn | -3.62 | HIS 13, GLN 15, LYS 16, VAL18,PHE20 |  |  |  |  |  |  |  |  |  |  |  |  |  |  |  |  |  |  |  |  |  |  |  |  |  |
|  | Etodolac | -3.34 | GLN15,LYS16,LEU17,VAL18,PHE19 |  |  |  |  |  |  |  |  |  |  |  |  |  |  |  |  |  |  |  |  |  |  |  |  |  |
| A2V | Hydrocortisone | -6.43 | VAL18,PHE19,PHE20,ALA21,GLU22,VAL24,ALA30,GLY33,LEU34 | VAL18 | 1 | PHE19 | PHE20 | 1 | VAL24 | 2 |  |  |  |  |  |  |  |  |  |  |  |  |  |  |  |  |  |  |
|  |  |  |  | PHE20 | 1 | ALA21 | GLU22 | 1 | ALA30 | 1 |  |  |  |  |  |  |  |  |  |  |  |  |  |  |  |  |  |  |
|  |  |  |  | GLU22 | 1 | GLY33 |  |  | LEU34 | 1 |  |  |  |  |  |  |  |  |  |  |  |  |  |  |  |  |  |  |
|  | Meloxicam | -6.41 | VAL18,PHE19,PHE20,ALA21,GLU22,VAL24,LEU34 | PHE20 | 1 | ALA21 | PHE19 | 1 |  |  |  |  |  |  |  |  |  |  |  |  |  |  |  |  |  |  | GLU22 | 1 |
|  |  |  |  | GLU22 | 2 |  |  |  |  |  |  |  |  |  |  |  |  |  |  |  |  |  |  |  |  |  |  |  |
|  | Valdecoxib | -6.37 | VAL18, PHE19, PHE20, ALA21, GLU22, VAL24, GLY33, LEU34, VAL36, GLY37 | PHE20 | 1 | VAL18 |  |  | ALA21 | 1 |  |  |  |  |  |  |  |  | PHE22 | 2 |  |  | GLY33 | 1 |  |  |  |  |
|  |  |  |  | GLU22 | 1 | LEU34 |  |  | VAL24 | 2 |  |  |  |  |  |  |  |  |  |  |  |  |  |  |  |  |  |  |
|  |  |  |  |  |  | GLY37 |  |  | VAL36 | 1 |  |  |  |  |  |  |  |  |  |  |  |  |  |  |  |  |  |  |
|  | Prednisolone | -6.35 | VAL18,PHE19,PHE20,ALA21,GLU22,VAL24,ALA30,GLY33,LEU34 | PHE20 | 1 |  | VAL18 | 1 |  |  |  |  |  |  | PHE19 | 1 |  |  |  |  |  |  |  |  |  |  |  |  |
|  |  |  |  | GLU22 | 2 |  |  |  |  |  |  |  |  |  |  |  |  |  |  |  |  |  |  |  |  |  |  |  |
|  | Etoricoxib | -6.29 | VAL18,PHE19,PHE20,ALA21,GLU22,VAL24,ALA30,GLY33,LEU34,VAL36,GLY37 |  |  |  |  |  |  |  |  |  |  |  |  |  |  |  | PHE19 | 1 |  |  | PHE20 | 1 |  |  |  |  |
|  | Celecoxib | -6.22 | VAL18,PHE19,PHE20,ALA21,GLU22,VAL24,GLY33,LEU34,VAL36,GLY37 | VAL18 | 1 |  |  |  |  |  |  |  |  |  |  |  |  |  |  |  |  |  |  |  |  |  |  |  |
|  |  |  |  | PHE20 | 1 |  |  |  |  |  |  |  |  |  |  |  |  |  |  |  |  |  |  |  |  |  |  |  |
|  | Rifamycin | -6.03 | LYS16,LEU17,PHE19,ALA21,ILE41 | LEU17 | 1 |  |  |  |  |  |  |  |  |  |  |  |  |  |  |  |  |  |  |  |  |  |  |  |
|  |  |  |  | PHE19 | 1 |  |  |  |  |  |  |  |  |  |  |  |  |  |  |  |  |  |  |  |  |  |  |  |
|  |  |  |  | ALA21 | 1 |  |  |  |  |  |  |  |  |  |  |  |  |  |  |  |  |  |  |  |  |  |  |  |
|  | Piroxicam | -5.97 | VAL18,PHE19,PHE20,ALA21,GLU22,VAL24,ALA30,GLY33,LEU34 | PHE20 | 1 |  |  |  |  |  |  |  |  |  |  |  |  |  |  |  |  |  |  |  |  |  |  |  |
|  |  |  |  | GLU22 | 1 |  |  |  |  |  |  |  |  |  |  |  |  |  |  |  |  |  |  |  |  |  |  |  |
|  |  |  |  |  |  |  |  |  |  |  |  |  |  |  |  |  |  |  |  |  |  |  |  |  |  |  |  |  |
|  | Prednisone | -5.94 | GLN15,LYS16,LEU17,VAL18,PHE19,VAL40,ILE41 | LYS16 | 1 |  |  |  |  |  |  |  |  |  |  |  |  |  |  |  |  |  |  |  |  |  |  |  |
|  |  |  |  | VAL18 | 2 |  |  |  |  |  |  |  |  |  |  |  |  |  |  |  |  |  |  |  |  |  |  |  |
|  | Dexamethasone | -5.71 |  |  |  |  |  |  |  |  |  |  |  |  |  |  |  |  |  |  |  |  |  |  |  |  |  |  |
|  | Nabumetone | -5.37 | VAL18,PHE19,PHE20,ALA21,GLU22,VAL24,LEU34 | PHE20 | 1 |  |  |  |  |  |  |  |  |  |  |  |  |  |  |  |  |  |  |  |  |  |  |  |
|  |  |  |  |  |  |  |  |  |  |  |  |  |  |  |  |  |  |  |  |  |  |  |  |  |  |  |  |  |
|  | Beclomethasone | -5.32 | PHE19,PHE20,ALA21,GLU22,VAL24,ALA30,GLY33,LEU34,VAL36,GLY37,VAL40 |  |  |  |  |  |  |  |  |  |  |  |  |  |  |  |  |  |  |  |  |  |  |  |  |  |
|  | Flurbiprofen | -4.89 | HIS13,HIS14,GLN15,LYS16,VAL18,PHE20 |  |  |  |  |  |  |  |  |  |  |  |  |  |  |  |  |  |  |  |  |  |  |  |  |  |
|  | Cromolyn | -4.67 |  |  |  |  |  |  |  |  |  |  |  |  |  |  |  |  |  |  |  |  |  |  |  |  |  |  |
|  | Etodolac | -4.62 | VAL18,PHE19,PHE20,ALA21,GLU22,VAL24,GLY33,LEU34 |  |  |  |  |  |  |  |  |  |  |  |  |  |  |  |  |  |  |  |  |  |  |  |  |  |
|  | Dexibuprofen | -4.14 | HIS13,HIS14,GLN15,LYS16,VAL18,PHE20 |  |  |  |  |  |  |  |  |  |  |  |  |  |  |  |  |  |  |  |  |  |  |  |  |  |
|  | Ibuprofen | -4.08 |  |  |  |  |  |  |  |  |  |  |  |  |  |  |  |  |  |  |  |  |  |  |  |  |  |  |
| E22del | Prednisone | -6.68 | LEU17, VAL18, PHE19, PHE20, LEU33, MET34 | LEU17 | 1 |  |  |  |  |  |  |  |  |  |  |  |  |  |  |  |  |  |  |  |  |  |  |  |
|  |  |  |  | PHE19 | 1 |  |  |  |  |  |  |  |  |  |  |  |  |  |  |  |  |  |  |  |  |  |  |  |
|  |  |  |  | LEU17 | 1 |  |  |  |  |  |  |  |  |  |  |  |  |  |  |  |  |  |  |  |  |  |  |  |
|  | Celecoxib | -6.52 | LYS16, LEU17, VAL18, PHE19, PHE20, ALA21, VAL23, ASN26, LYS 27, ILE30, ILE31 | PHE19 | 1 |  |  |  |  |  |  |  |  |  |  |  |  |  |  |  |  |  |  |  |  |  |  |  |
|  |  |  |  | ALA21 | 1 |  |  |  |  |  |  |  |  |  |  |  |  |  |  |  |  |  |  |  |  |  |  |  |
|  |  |  |  | ASN26 | 1 |  |  |  |  |  |  |  |  |  |  |  |  |  |  |  |  |  |  |  |  |  |  |  |
|  | Beclomethasone | -6.11 | ASP7, SER8, GLU11, VAL12, HIS14,GLN15, LYS16,LEU17 |  |  |  |  |  |  |  |  |  |  |  |  |  |  |  |  |  |  |  |  |  |  |  |  |  |
|  | Etoricoxib | -6.11 | LEU17, VAL18, PHE19, PHE20, ALA21, ASN26, ALA29, ILE30, LEU33 |  |  |  |  |  |  |  |  |  |  |  |  |  |  |  |  |  |  |  |  |  |  |  |  |  |
|  | Valdecoxib | -5.99 | ASP1, ALA2, SER8, TYR10, GLU11, VAL12, HIS13 |  |  |  |  |  |  |  |  |  |  |  |  |  |  |  |  |  |  |  |  |  |  |  |  |  |
|  | Hydrocortisone | -5.78 | HIS14,GLN15, LYS16,LEU17, VAL18, PHE19, | GLN15 | 2 |  |  |  |  |  |  |  |  |  |  |  |  |  |  |  |  |  |  |  |  |  |  |  |
|  |  |  |  | LEU17 | 1 |  |  |  |  |  |  |  |  |  |  |  |  |  |  |  |  |  |  |  |  |  |  |  |
|  | Piroxicam | -5.71 | LEU17,VAL18,PHE19,PHE20,ALA21,ASN26,ALA29,ILE30,LEU33 |  |  |  |  |  |  |  |  |  |  |  |  |  |  |  |  |  |  |  |  |  |  |  |  |  |
|  | Meloxicam | -5.67 | LEU17, VAL18, PHE19, PHE20, ALA21, ASN26, ALA29, ILE30, LEU33 | VAL18 | 1 |  |  |  |  |  |  |  |  |  |  |  |  |  |  |  |  |  |  |  |  |  |  |  |
|  |  |  |  | PHE20 | 2 |  |  |  |  |  |  |  |  |  |  |  |  |  |  |  |  |  |  |  |  |  |  |  |
|  | Prednisolone | -5.61 | LEU17,VAL18,PHE19,PHE20,LEU33 |  |  |  |  |  |  |  |  |  |  |  |  |  |  |  |  |  |  |  |  |  |  |  |  |  |
|  | Rifamycin | -5.54 | LEU17,VAL18,PHE19,PHE20,LEU33,MET34,VAL35,GLY36,GLY37 |  |  |  |  |  |  |  |  |  |  |  |  |  |  |  |  |  |  |  |  |  |  |  |  |  |
|  | Dexamethasone | -5.42 | PHE20, ALA21, VAL 23, ASN 26, ALA 29, LEU33, GLY37, VAL38, VAL39, ALA41 |  |  |  |  |  |  |  |  |  |  |  |  |  |  |  |  |  |  |  |  |  |  |  |  |  |
|  | Nambumetone | -4.91 | GLY28, ALA29, GLY32, LEU33, GLY36, GLY37, VAL38, VAL39, ILE40, ALA41 |  |  |  |  |  |  |  |  |  |  |  |  |  |  |  |  |  |  |  |  |  |  |  |  |  |
|  | Etodolac | -4.65 | VAL18, PHE19, PHE20, ALA21,VAL23, SER25, ASN26, LYS27, ILE30 |  | 3 |  |  |  |  |  |  |  |  |  |  |  |  |  |  |  |  |  |  |  |  |  |  |  |
|  | Flurbiprofen | -4.53 | LYS16, LEU17, VAL18, PHE19 |  |  |  |  |  |  |  |  |  |  |  |  |  |  |  |  |  |  |  |  |  |  |  |  |  |
|  | Ibuprofen | -4.52 | VAL18,PHE19,PHE20, ALA21, VAL23, ASN26, ILE30 |  |  |  |  |  |  |  |  |  |  |  |  |  |  |  |  |  |  |  |  |  |  |  |  |  |
|  | Cromolyn | -4.43 | PHE19, PHE20, ALA21, VAL 23, GLY 24, SER 25, ASN 26, LYS 27, ILE 30 |  |  |  |  |  |  |  |  |  |  |  |  |  |  |  |  |  |  |  |  |  |  |  |  |  |
|  | Dexibuprofen | -4.17 | VAL18, PHE19, PHE20, ALA21,VAL23, GLY24, ASN26, ILE30 | 2 |  |  |  |  |  |  |  |  |  |  |  |  |  |  |  |  |  |  |  |  |  |  |  |  |
|  |  |  |  |  |  |  |  |  |  |  |  |  |  |  |  |  |  |  |  |  |  |  |  |  |  |  |  |  |
| Top five result | | | | | | | | | | | | | | | | | | | | | | | | | | | | |

**Supplementary Table 4**

| **Anti-inflammatory drug** | **Flurbiprofen** | | **Piroxicam** | | **Ibuprofen** | | **Hydrocortisone** | | **Prednisolone** | | **Celecoxib** | | **Etodolac** | | **Nabumetone** | | **Dexamethasone** | | **Prednisone** | | **Dexibuprofen** | | **Valdecoxib** | | **Etoricoxib** | | **Meloxicam** | | **Cromolyn** | | **Beclomethasone** | | **Rifamycin** | |
| --- | --- | --- | --- | --- | --- | --- | --- | --- | --- | --- | --- | --- | --- | --- | --- | --- | --- | --- | --- | --- | --- | --- | --- | --- | --- | --- | --- | --- | --- | --- | --- | --- | --- | --- |
| Model | Result | Probability | Result | Probability | Result | Probability | Result | Probability | Result | Probability | Result | Probability | Result | Probability | Result | Probability | Result | Probability | Result | Probability | Result | Probability | Result | Probability | Result | Probability | Result | Probability | Result | Probability | Result | Probability | Result | Probability |
| Absorption |  |  |  |  |  |  |  |  |  |  |  |  |  |  |  |  |  |  |  |  |  |  |  |  |  |  |  |  |  |  |  |  |  |  |
| Blood-Brain Barrier | BBB+ | 0.9824 | BBB- | 0.9659 | BBB+ | 0.9619 | BBB+ | 0.9383 | BBB+ | 0.9383 | BBB+ | 0.9713 | BBB+ | 0.9065 | BBB+ | 0.9401 | BBB+ | 0.9781 | BBB+ | 0.9383 | BBB+ | 0.9619 | BBB+ | 0.939 | BBB+ | 0.9399 | BBB- | 0.9811 | BBB+ | 0.5988 | BBB+ | 0.9644 | BBB- | 0.9659 |
| Human Intestinal Absorption | HIA+ | 1 | HIA+ | 0.9898 | HIA+ | 0.9927 | HIA+ | 0.9918 | HIA+ | 0.9918 | HIA+ | 1 | HIA+ | 0.9971 | HIA+ | 1 | HIA+ | 0.992 | HIA+ | 0.9918 | HIA+ | 0.9927 | HIA+ | 1 | HIA+ | 0.9776 | HIA+ | 0.9156 | HIA+ | 0.6657 | HIA+ | 0.992 | HIA+ | 0.6688 |
| AMES Toxicity | Non AMES toxic | 0.9659 | Non AMES toxic | 0.8767 | Non AMES toxic | 0.9894 | Non AMES toxic | 0.9132 | Non AMES toxic | 0.9132 | Non AMES toxic | 0.7185 | Non AMES toxic | 0.9132 | Non AMES toxic | 0.8942 | Non AMES toxic | 0.9132 | Non AMES toxic | 0.9132 | Non AMES toxic | 0.9894 | Non AMES toxic | 0.828 | Non AMES toxic | 0.7952 | Non AMES toxic | 0.8576 | Non AMES toxic | 0.9132 | Non AMES toxic | 0.9041 | Non AMES toxic | 0.5952 |
| Acute Oral Toxicity | II | 0.7385 | II | 0.7206 | III | 0.8084 | III | 0.7997 | III | 0.7997 | III | 0.6499 | II | 0.7385 | III | 0.8517 | III | 0.8328 | III | 0.7997 | III | 0.8084 | III | 0.575 | III | 0.576 | II | 0.6971 | III | 0.6735 | III | 0.7795 | III | 0.7013 |
| Carcinogenicity (Three-class) | Non-required | 0.5755 | Non-required | 0.6998 | Non-required | 0.7313 | Non-required | 0.736 | Non-required | 0.736 | Non-required | 0.7022 | Non-required | 0.694 | Non-required | 0.5692 | Non-required | 0.7364 | Non-required | 0.736 | Non-required | 0.7313 | Non-required | 0.567 | Non-required | 0.6563 | Non-required | 0.6941 | Non-required | 0.5173 | Non-required | 0.7246 | Danger | 0.4575 |
| **ADMET Predicted Profile --- Regression** |  |  |  |  |  |  |  |  |  |  |  |  |  |  |  |  |  |  |  |  |  |  |  |  |  |  |  |  |  |  |  |  |  |  |
| **Model** | **Value** | **Unit** | **Value** | **Unit** | **Value** | **Unit** | **Value** | **Unit** | **Value** | **Unit** | **Value** | **Unit** | **Value** | **Unit** | **Value** | **Unit** | **Value** | **Unit** | **Value** | **Unit** | **Value** | **Unit** | **Value** | **Unit** | **Value** | **Unit** | **Value** | **Unit** | **Value** | **Unit** | **Value** | **Unit** | **Value** | **Unit** |
| Absorption |  |  |  |  |  |  |  |  |  |  |  |  |  |  |  |  |  |  |  |  |  |  |  |  |  |  |  |  |  |  |  |  |  |  |
| Aqueous solubility | -4.4277 | LogS | -4.0977 | LogS | -3.9041 | LogS | -3.0321 | LogS | -3.0321 | LogS | -3.1816 | LogS | -3.7184 | LogS | -4.607 | LogS | -3.7026 | LogS | -3.032 | LogS | -3.9041 | LogS | -3.2339 | LogS | -4.0142 | LogS | -3.7597 | LogS | -2.7244 | LogS | -3.7801 | LogS | -3.7567 | LogS |
| Caco-2 Permeability | 1.5669 | LogPapp, cm/s | 1.3568 | LogPapp, cm/s | 1.7486 | LogPapp, cm/s | 0.8616 | LogPapp, cm/s | 0.8616 | LogPapp, cm/s | 1.0149 | LogPapp, cm/s | 0.9653 | LogPapp, cm/s | 1.8214 | LogPapp, cm/s | 1.0834 | LogPapp, cm/s | 0.8616 | LogPapp, cm/s | 1.7486 | LogPapp, cm/s | 0.9541 | LogPapp, cm/s | 1.5935 | LogPapp, cm/s | 1.2073 | LogPapp, cm/s | -0.1585 | LogPapp, cm/s | 1.0479 | LogPapp, cm/s | 0.4455 | LogPapp, cm/s |
| Toxicity |  |  |  |  |  |  |  |  |  |  |  |  |  |  |  |  |  |  |  |  |  |  |  |  |  |  |  |  |  |  |  |  |  |  |
| Rat Acute Toxicity | 3.1121 | LD50, mol/kg | 3.1545 | LD50, mol/kg | 2.3092 | LD50, mol/kg | 1.8914 | LD50, mol/kg | 1.8914 | LD50, mol/kg | 2.3719 | LD50, mol/kg | 3.4536 | LD50, mol/kg | 1.7384 | LD50, mol/kg | 2.1482 | LD50, mol/kg | 1.8914 | LD50, mol/kg | 2.3092 | LD50, mol/kg | 2.068 | LD50, mol/kg | 2.41 | LD50, mol/kg | 3.4619 | LD50, mol/kg | 2.5373 | LD50, mol/kg | 1.984 | LD50, mol/kg | 2.5438 | LD50, mol/kg |
| Fish Toxicity | 0.8577 | pLC50, mg/L | 1.7284 | pLC50, mg/L | 1.3122 | pLC50, mg/L | 1.0228 | pLC50, mg/L | 1.0228 | pLC50, mg/L | 1.5816 | pLC50, mg/L | 1.3755 | pLC50, mg/L | 0.0311 | pLC50, mg/L | 0.8318 | pLC50, mg/L | 1.0228 | pLC50, mg/L | 1.3122 | pLC50, mg/L | 1.8183 | pLC50, mg/L | 1.5245 | pLC50, mg/L | 1.6914 | pLC50, mg/L | 0.8703 | pLC50, mg/L | 0.766 | pLC50, mg/L | 0.4611 | pLC50, mg/L |
| Tetrahymena Pyriformis Toxicity | 0.9401 | pIGC50, ug/L | 0.5383 | pIGC50, ug/L | 1.3858 | pIGC50, ug/L | 0.9935 | pIGC50, ug/L | 0.9935 | pIGC50, ug/L | 0.5421 | pIGC50, ug/L | 0.7547 | pIGC50, ug/L | 1.1867 | pIGC50, ug/L | 1.0232 | pIGC50, ug/L | 0.9935 | pIGC50, ug/L | 1.3858 | pIGC50, ug/L | 0.4425 | pIGC50, ug/L | 0.816 | pIGC50, ug/L | 0.5386 | pIGC50, ug/L | 0.5327 | pIGC50, ug/L | 1.0762 | pIGC50, ug/L | 0.7331 | pIGC50, ug/L |

**Supplementary Table 5**

| COACH RESULT | | | | | |
| --- | --- | --- | --- | --- | --- |
| Rank | C-score | Cluster size | PDB Hit | Lig Name | Consensus Binding Residues |
| 1 | 0.15 | 297 | 3lyaA | RHE | 26,27,28,29 |
| 2 | 0.12 | 227 | 4l6va | CLA | 26,27,29,30,32,33,36 |
| 3 | 0.11 | 206 | 3bz1X | CLA | 34,35,38,39 |
| 4 | 0.09 | 179 | 1rxqD | SER | 31,32,34,35 |
| 5 | 0.08 | 153 | 2xqcD | Nuc.Acid | 32,33,36,37 |
| 6 | 0.05 | 95 | 4pj0I | CLA | 33,34,36,37,40,41 |
| 7 | 0.04 | 82 | 2akaA | PEPTIDE | 38,39,40,41 |
| 8 | 0 | 1 | 1kdgB | MAN | 23,24 |
| 9 | 0 | 1 | 3bamA | MN | 1,19 |
| 10 | 0 | 4 | 4ou7A | Nuc.Acid | 20,21,33,36,37,40 |
| S-Site RESULT | | | | | |
| Rank | C-score | Cluster size | Templates | Ligands | Predicted binding site residues |
| 1 | 0.28 | 2 | List | CLA(2) | 33,34,37,41 |
| 2 | 0.19 | 1 | List | MN(1) | 1,19 |
| TM-Site RESULT | | | | | |
| Rank | C-scorea | Cluster size | Rep Template | Ligands | Predicted binding site residues |
| 1 | 0.36 | 89 | 1e7gA_BS06_MYR | MG(42),CA(27),ZN(20),.. | 26,27,29,30 |
| 2 | 0.34 | 59 | 4l6va_BS38_CLA | CA(21),NUC(20),ZN(18),.. | 26,27,29,30,32,33,36 |
| 3 | 0.33 | 63 | 4az7A_BS02_LOG | CLA(29),CA(20),MG(14),.. | 34,35,38,39 |
| 4 | 0.32 | 34 | 3h0qB_BS02_B37 | CLA(13),MG(11),III(10),.. | 30,31,32,35 |
| 5 | 0.3 | 51 | 2xqcD_BS02_NUC | BCL(22),CLA(17),ZN(12),.. | 32,33,36,37 |

**Supplementary Table 6**
